# Proteomic Changes of Tissue-Tolerable Plasma Treated Airway Epithelial Cells and Their Relation to Wound Healing

**DOI:** 10.1155/2015/506059

**Published:** 2015-10-11

**Authors:** Derik Lendeckel, Christine Eymann, Philipp Emicke, Georg Daeschlein, Katrin Darm, Serena O'Neil, Achim G. Beule, Thomas von Woedtke, Uwe Völker, Klaus-Dieter Weltmann, Michael Jünger, Werner Hosemann, Christian Scharf

**Affiliations:** ^1^Department of Otorhinolaryngology, Head and Neck Surgery, University Medicine Greifswald, 17475 Greifswald, Germany; ^2^Department of Dermatology, University Medicine Greifswald, 17475 Greifswald, Germany; ^3^Leibniz Institute for Plasma Science and Technology (INP), 17489 Greifswald, Germany; ^4^Interfaculty Institute of Genetics and Functional Genomics, University of Greifswald, 17475 Greifswald, Germany

## Abstract

*Background*. The worldwide increasing number of patients suffering from nonhealing wounds requires the development of new safe strategies for wound repair. Recent studies suggest the possibility of nonthermal (cold) plasma application for the acceleration of wound closure. *Methods*. An *in vitro* wound healing model with upper airway S9 epithelial cells was established to determine the macroscopically optimal dosage of tissue-tolerable plasma (TTP) for wound regeneration, while a 2D-difference gel electrophoresis (2D-DIGE) approach was used to quantify the proteomic changes in a hypothesis-free manner and to evaluate the balance of beneficial and adverse effects due to TTP application. *Results*. Plasma doses from 30 s up to 360 s were tested in relation to wound closure after 24 h, 48 h, 72 h, 96 h, and 120 h, in which lower doses (30, 60, and 120 s) resulted in dose-dependent improved wound healing rate compared to untreated cells. Thereby, the 120 s dose caused significantly the best wound healing properties after 96 and 120 h. The proteome analysis combined with IPA revealed that a lot of affected stress adaptation responses are linked to oxidative stress response emphasizing oxidative stress as a possible key event in the regeneration process of epithelial cells as well as in the adaptation to plasma exposure. Further cellular and molecular functions like proliferation and apoptosis were significantly up- or downregulated by all TTP treatments but mostly by the 120 s dose. *Conclusions*. For the first time, we were able to show plasma effects on cellular adaptation of upper airway epithelial S9 cells improving wound healing. This is of particular interest for plasma application, for example, in the surgery field of otorhinolaryngology or internal medicine.

## 1. Background

Wound healing is an important necessity for survival. A worldwide increasing number of patients suffering from nonhealing and chronic infected wounds require the development of new strategies for wound repair. One of these promising strategies is the application of nonthermal plasma.

Plasma is defined as the fourth state of matter and is generated by almost completely or partly ionisation of gas. Naturally occurring thermal plasmas are found, for example, as lightning in thunderstorms and in the magnetosphere of astronomical objects. Plasma can also be created artificially and is commonly used in the industrial sector [1–4]. But only recently, possible applications of artificial plasma in diverse medical fields have begun to be investigated.

Besides thermal artificial plasma, which has become an innovative tool in, for example, microsurgery for the ablation and cauterisation of biological tissue [5–8], it is also possible to create low-temperature artificial plasmas (“nonthermal” or “cold”).

Due to many observed beneficial effects, the therapeutic potential of cold plasma has emerged as a promising new option in several clinical settings. In contrast to the thermal plasma equivalent, cold plasma mediated effects have been proposed to include modulation of the immune system [9–11], arrest of tumour proliferation [12–16], and improvement of wound healing [17–20].

Thus, the application of cold plasma to the skin is of particular importance in dermatology, where conventional therapies of chronic wounds are mostly insufficient. It has been shown that positive effects of nonthermal plasma on wound healing clearly result from its antiseptic properties [18, 21–24]. But also the increase of cell proliferation and migration as significant effects of particular low plasma doses might be also responsible for the support of wound healing [24].

Clinical studies have focused on wounds of the epidermis like venous ulcers [18, 21, 25] or skin graft donor sites [20, 26], whereas molecular research has predominantly been performed on common bacteria of the skin [23, 27, 28] or keratinocytes [29, 30].

Recent studies and clinical trials have demonstrated the efficacy of tissue-tolerable plasma (TTP) in wound healing [13, 17]. It may have the potential to improve wound healing in all clinical settings with healing in secondary intention. However, understanding of the underlying molecular mechanisms is limited owing to the complexity of both the plasma, which consists of charged particles, reactive oxygen species (ROS), and several types of radiation (VUV, UVA, and UVB) [28], and the tissue or cell. Available studies on cellular mechanisms are limited to targeted molecular research based on key mediators and their hypothetical downstream effects [19].

To our knowledge, only two global transcriptomic studies have been performed to analyse the entirety of the molecular mechanisms in human epithelial cell lines influenced by indirect plasma treatment [31, 32]. These recent transcriptomic analyses revealed an influence on several biological pathways, such as oxidative stress response, repair, and inflammation signalling. Oxidative stress might be a result of the existence of reactive oxygen or nitrogen species (ROS, RNS) such as ozone, superoxide, peroxide, and hydroxyl radicals which are generated by different plasma sources [14]. However, no gel-based proteomic studies have been reported to date.

It is interesting to note that some recent clinical trials and molecular research have already revealed dose-dependent cellular effects in response to cold plasma treatment [29, 31, 33]. These studies indicate that increasing plasma doses could heighten the proportion of cells which are forced to undergo apoptosis, leading consequently to a progressive negation of positive effects. The optimal dosage of tissue-tolerable plasma (TTP) treatment based on evaluation of molecular results has not been ascertained yet. Therefore, the aim of the present study was to identify the effects of varying TTP doses on molecular pathways in upper airway epithelial S9 cells at several time points. An* in vitro* wound healing model was established to determine the macroscopically optimal dosage of TTP treatment for wound regeneration, while a 2D-difference gel electrophoresis (2D-DIGE) approach was used to quantify the proteomic changes in a hypothesis-free manner and to evaluate the balance of beneficial and adverse effects due to TTP application. Human S9 upper airway epithelial cells serve as a model for mucosa cells of the throat in a broader sense and plasma application could be of particular interest, for example, for the field of surgery in otorhinolaryngology.

## 2. Methods

### 2.1. Cultivation of S9 Epithelial Cells

Upper airway S9 epithelial cells [34] were incubated with 10 mL standard cell cultivation medium MEM (Promocell) in a cell culture plate at atmospheric conditions (at 37°C, 5% CO_2_, and >85% humidity) and were grown up to an approximate cell density of 1 × 10^7^ cells/cm^2^. Cell counting was performed with CASY (Roche Life Sciences). Then subcultivation was performed by removing the cell cultivation medium, washing cells with 5.0 mL PBS, and overlaying them with 1.0 mL trypsin-solution (PAN Biotech, 10 ng/mL) for 5 to 15 minutes to detach cells from the surface [35, 36]. Then, 3 mL standard cell cultivation medium was added, and cells were repeatedly pipetted, divided into aliquots, and transferred into new cell culture plates, each containing 9 mL fresh cell cultivation medium with a subcultivation ratio of  1 : 4, which corresponded to approximately 1 × 10^7^ viable cells per cell culture plate. These subcultures were incubated as described above until 95% confluency of cells was reached. Substitution of medium against fresh medium was performed after 60 h.

### 2.2. Plasma Treatment of S9 Epithelial Cells

For plasma treatment the kINPen08, an atmospheric pressure plasma jet developed by the INP in Greifswald, was used ([24]; Figure S1-A). To generate the plasma, argon gas was used and a high-frequency voltage of 2–6 kVpp, 1.1 MHz, was applied to the electrode. At the tip of the plasma jet, temperatures measured ranged between 37°C and 42°C. Different plasma doses which varied between 30 s and 360 s were applied to cell cultures. For plasma application, the cell cultures covered with 1 mL standard cell medium were moved in a grid pattern underneath the visible tip of the fixed plasma jet for the requested time (supplementary Figure S1 (B-D) in the Supplementary Material available online at http://dx.doi.org/10.1155/2015/506059). After plasma treatment, untreated and treated cell cultures were incubated for several time periods enabling assessment of wound healing and proteome analyses (incubation for 24 h, 48 h, and 72 h before cell harvesting).

### 2.3. *In Vitro* Wound Model

**Figure 1:**
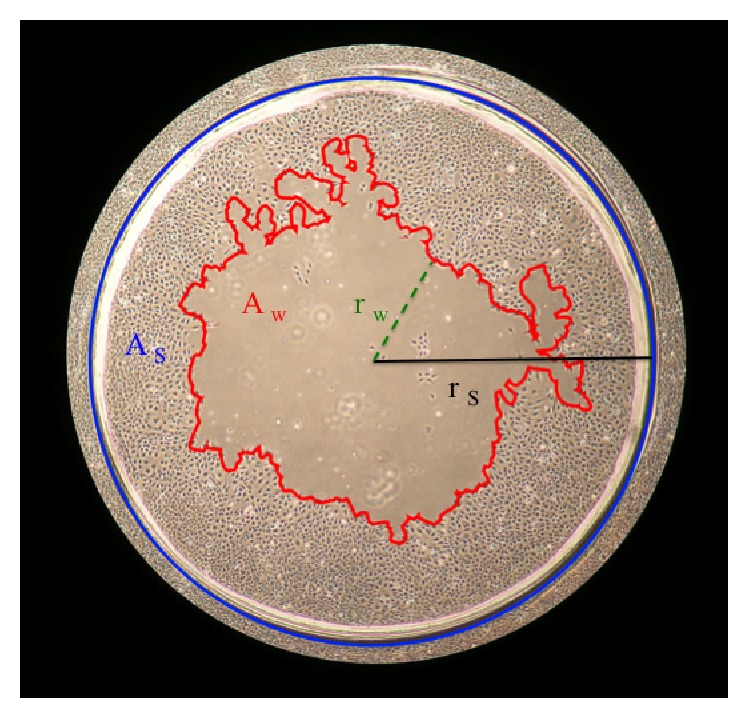
Schematic description of wound measurement. The blue line marks the initially punched wound border. The red line defines the wound border of the current moment. *r*_*w*_ = radius of the minimal diameter of the wound; *r*_*s*_ = radius of punched wound; *A*_*w*_ = wound area related to the defined punched area *A*_*s*_.

In order to study effects of plasma treatment on wound healing, we used cell cultures of S9 upper airway epithelial cells in a cell culture plate of 10 cm diameter. Cell cultures were treated according to an established wound model, previously described by Beule et al. and Roth et al. [35, 36]. In confluent cell cultures 21 circular wounds per plate were created using a 4 mm sterile biopsy punch (pfm AG, Cologne, Germany) (Figure S1-C). All wounds were documented photographically and subsequently treated with the kINPen08, following a grid pattern for a duration of 30 s, 60 s, 120 s, 240 s, and 360 s (Figure S1-D). Untreated wounds (controls) were covered with a light-proof lid for the same duration. Wound areas were documented at a 4-fold magnification on an inverted microscope (Nikon Eclipse) 24 h, 48 h, 72 h, 90 h, and 120 h after appropriate incubation. The size of six wounds was measured on the photographs by using an area-calculating tool of Photoshop CS5 (Adobe, San Jose, Calif., USA) (Figure 1). The average of each condition was calculated. Each of the different treatment groups was tested against the untreated control group. Effects were considered statistically significant for* p* < 0.05 using ANOVA. Additionally, the nonparametric Mann-Whitney* U* test was used to compare two groups (96 h, 120 h values,* p* < 0.05). Sphericity of wounds was assumed.

### 2.4. Sample Preparation for Proteomic Analyses

For cell harvesting, the medium was removed and cells were washed with PBS. After PBS removal, cells were incubated with 1.8 mL sample buffer (8 M urea, 2 M thiourea) and detached with a cell scraper (Greiner BioOne). For disruption, cells were initially shock-frozen in liquid nitrogen and then defrosted in a thermomixer (Eppendorf, Hamburg, Germany) at 1400 rpm at 30°C for 10 minutes. After five freeze and thaw cycles, samples were centrifuged to remove cell debris. Supernatants were transferred into new tubes and stored at −70°C prior to further processing. Protein concentrations were estimated using a Bradford assay (Bio-Rad, Munich, Germany) as previously described [37].

### 2.5. Two-Dimensional Difference in Gel Electrophoresis (2D-DIGE)

Proteome analysis was performed for two separate experimental series of four conditions (control, 30, 60, and 120 s of plasma treatment) and three time points (24, 48, and 72 h). Protein lysates were labelled with Cy-dyes (Cy3 or Cy5) according to the manufacturer's instructions (GE Healthcare, Munich, Germany). In order to reduce intergel variations an internal standard pool consisting of 50 *µ*g aliquots of all samples was generated and labelled with the Cy2-dye. 50 *µ*g protein of each sample or internal standard was labelled with 400 pmol of corresponding dye on ice in the dark for 30 min. Reaction was quenched with 10 mM L-lysine for 10 min under the same conditions. From all samples, four technical replicates labelled either with Cy3-dye or with Cy5-dye were analysed. For a two-dimensional-difference gel electrophoresis (2D-DIGE) approach two labelled samples (Cy3 and Cy5, each 50 *µ*g) were mixed with the internal standard (Cy2, 50 *µ*g) in rehydration buffer [38] and applied to IPG strips (pH 4–7, 24 cm, GE Healthcare, Munich, Germany) by in-gel rehydration. After rehydration overnight, isoelectric focusing (IEF) was carried out as described earlier [38]. Subsequently, the second dimension (SDS-PAGE) was carried out using low-fluorescent glass plates (GE Healthcare) in the PROTEAN plus Dodeca Cell system (Bio-Rad, Munich, Germany). After 2D-PAGE, the Cy2 (internal standard), Cy3, and Cy5 labelled proteins in each gel were visualized by using a Typhoon 9400 laser scanner (GE Healthcare) at 100 microns by using the following excitation/emission wavelengths: 488/520 nm (Cy2), 532/670 nm (Cy3), and 633/670 nm (Cy5). The resulting images (3 per gel) were processed with dedicated software as described below. Dose- and time-resolved proteome analyses (dose-dependent: control versus 30 s, 60 s, and 120 s of TTP treatment, time-dependent: the 24 h sample of each dose versus 48 h, 72 h) were performed for controls and samples treated with TTP.

### 2.6. Statistical Analyses

Analyses of the scanned gel images and data analyses were performed with the software package Delta2D (Version 4.2, Decodon GmbH, Greifswald, Germany). After aligning all image sets in position by using the internal standard (Cy2), all samples of a set were merged in order to create a fused image (2D proteome map) in union fusion mode [39]. Spot detection and editing were done with the Delta2D software on the union fused image and then spots and spot labels were transferred onto all other images included in the analyses. TMeV software (implemented in Delta2D) was used for statistical analyses and graphical display of expression profiles. Acquired spot expression values were automatically normalized, first by correcting differences between intensities of all gel images and then by calibrating spot volumes (Cy3 or Cy5) to the internal standard (Cy2). In order to identify differences in spot intensity, the values of TTP treated samples were divided by the corresponding baseline values of the untreated control. Stringent selection criteria were employed in order to reduce false positive results and changes were only considered significant when the following three criteria were fulfilled: (1) the change of the intensity ratio had to exceed a factor of 1.5, (2) the* p* value of the corresponding analysis of variance test had to be lower than 0.05 (one-way ANOVA), and (3) raw values of each spot had to exceed 0.3, avoiding calculation of ratios of spots close to background intensities. After TMeV analyses, principle component analysis (PCA) [40] was performed to validate reproducibility of experimental conditions and identify possible outliers. Each subset of sample (4 technical replicates per group) was defined as point in an* n*-dimensional Cartesian coordinate system, described by 1582 vectors, which represent the amount of expression values of every detected protein spot. Those samples were transferred into 2-dimensional space by focusing on the principal components with the largest variances. After removal of an obvious outlier in two groups (60 s/24 h and 120 s/24 h), caused by a hardware failure of the scanning device, regulated spots were identified with MALDI-TOF-MS as described below.

### 2.7. Preparative 2D Gel Electrophoresis and Sample Preparation for Mass Spectrometry

Preparative two-dimensional gel electrophoresis was performed as previously described [38]. Briefly, 450 *µ*g of protein was pooled from treated and untreated samples of each condition (75 *µ*g each) and added to the rehydration buffer. The resulting 2D-PAGE gels were stained with colloidal Coomassie brilliant blue according to the manufacturer's instructions (GE Healthcare). Digital documentation of the gel images was performed by a transmission light scan. Gel image analyses were performed with the Delta2D software package (Decodon GmbH) as described above. Spots of interest were processed for identification as described by Eymann et al. [41]. Briefly, spots were excised manually with a 2 mm picking head and transferred into 96-well microplates, which were loaded with 100 *µ*L of Lichrosolv water in each well. Tryptic digestion was performed automatically in an Ettan Spot Handling Workstation (Amersham Biosciences) as well as the subsequent spotting of peptide solution onto MALDI targets. For peptide extraction, gel pieces were covered with 60 *µ*L 50% v/v ACN/0.1% w/v TFA and incubated for 30 min at 37°C. Supernatants containing peptides were transferred into new microtiter plates. Peptide extraction was performed again with 40 *µ*L of the same solution. Joined supernatants were now completely dried at 40°C for 220 min. Peptides were dissolved in 2.2 *µ*L of 0.5% w/v TFA/50% v/v ACN and 0.7 *µ*L of each solution was spotted directly onto MALDI targets. Then, this sample solution was mixed with 0.4 *µ*L of matrix solution by aspirating five times. After drying for 10–15 min, samples were measured using the MALDI-TOF-TOF instrument.

### 2.8. Matrix-Assisted Laser Desorption-Ionization Time-of-Flight Mass Spectrometry (MALDI-TOF-MS)

MALDI-TOF-MS measurements were performed with a Proteome-Analyzer 4800 (Applied Biosystems, Foster City, CA, USA). Reflector mode was used in order to record the spectra in a mass range from 900 to 3700 Da with a mass focus to 2000 Da. Twenty-five subspectra with 100 shots per subspectrum were accumulated for one main spectrum using a random search pattern. The standard peptide search tolerance was set to 50 ppm. The peak lists were created and searched automatically by using GPS-Explorer software package (Applied Biosystems, Foster City, CA, USA). These peak lists were compared to a UniProt-SwissProt database (Rel. 51.5 restricted to human taxonomy) by the MASCOT search engine (Version 2.1). Positive identifications had to reach the following specifications: sequence coverage of at least 30% and a MOWSE-score of at least 49. Proteins and peptides, which failed to meet the 30% sequence coverage requirement, were reanalysed with more accurate MALDI-TOF-MS. The MALDI-TOF-MS/MS analyses were used for the five strongest peaks of the previous MS-spectrum. Here, 20 subspectra with 125 shots per subspectrum were accumulated using a random search pattern. The same tools were used for peak list interpretation. Results reaching a MOWSE-score of at least 49 in reflector mode (MALDI-TOF-MS) and being confirmed by subsequent measurement of the strongest peaks (MS/MS) were regarded as positively identified proteins. The confirmation by subsequent measurements (MS/MS) was particularly useful for protein identification in spots, which contained multiple proteins. Protein identifications and statistically relevant data were combined via unique spot-IDs using the MSRepo database software (Decodon, Greifswald, Germany). Categorization of the identified proteins was achieved by using the PANTHER Classification System (http://www.PANTHERdb.org/) [42].

### 2.9. Network and Protein Functional Analyses Using Specialized Software

Expression profiles of statistically significant changing spots were exported. TMeV data were combined with identification lists from mass spectrometry and then imported into Ingenuity Pathway Analysis (Ingenuity Systems). Using this program, it was possible to create protein networks and pathways, which contained the proteins displaying changes in intensity and thereby placing individual protein data into physiological context.

## 3. Results

### 3.1. Dose-Dependent Effects of Plasma Treatment on Wound Healing of Upper Airway S9 Epithelial Cells

**Figure 2:**
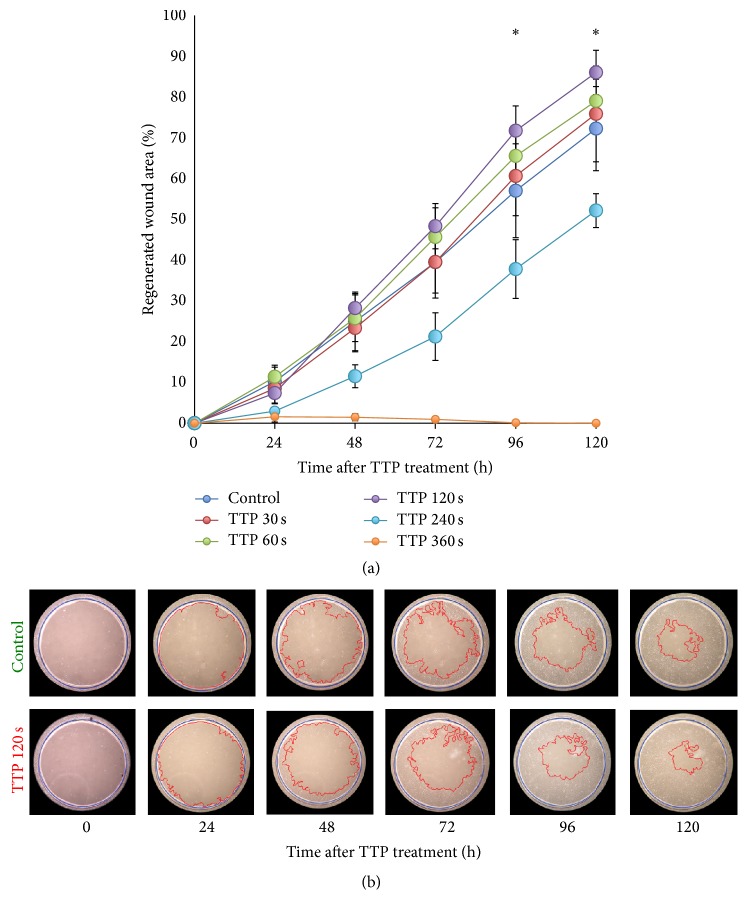
Time course of wound healing processes of S9 epithelial cells. (a) The regenerated wound area was calculated by taking the average of six representative wounds per culture plate/dose and time point. Significant differences (see ^*∗*^) were observed after 96 h and 120 h between 120 s plasma treated and untreated control samples. Plasma application for 30 s and 60 s provided no significant effect on wound healing, ^*∗*^ *p* < 0.05 (Student's *t*-test, *n* = 6). (b) Cell cultures of plasma treated and untreated samples were cut in order to create circular artificial wounds. The outer blue line represents the primary wound border. The red line represents the current healing border after 24 h, 48 h, 72 h, 96 h, and 120 h. For every time point, the effect of 120 s TTP treatment on wound healing is shown in comparison to untreated samples “control.”

The effect of various plasma doses on the rate of wound healing of S9 airway epithelial cells was examined over 120 hours in an* in vitro* wound model. In comparison to the control cell cultures, the application of low plasma doses (30, 60, and 120 s) led to successive accelerated wound regeneration after 96 h. Higher doses either resulted in significantly lower regenerated wound area (240 s) or even blocked the ability for regeneration and to close the wound area (360 s) (Figure 2(a)). The 120 s dose provided the most intense stimulation of wound healing after 48 h and at subsequent time points. Statistical analysis ensured a significantly increased wound healing rate after 96 h and 120 h (*p* < 0.05). No significance could be determined for cells which were treated for 30 and 60 s, although there is a trend for increasing wound healing potential over 120 h (Figure 2(a)). The difference in the wound healing area between control cells and 120 s of plasma treated cells is clearly visible as the regenerated wound area comprised approximately 72% of the initial wound area after 96 h and 86% after 120 h, whereas control samples provided 57% and 72% for the same time points (Figure 2(b)). The application of low plasma doses (≤120 s), in the following termed tissue-tolerable plasma (TTP), seems to improve cell proliferation and migration, thereby increasing the rate of wound healing of S9 airway epithelial cells. Higher doses seem to repress or stop cell proliferation/migration and could have therefore cell damaging or lethal effects.

### 3.2. The Protein Expression Is Influenced by Tissue-Tolerable Plasma (TTP) Doses as Revealed by 2D-DIGE and MALDI-MS Analysis

**Figure 3:**
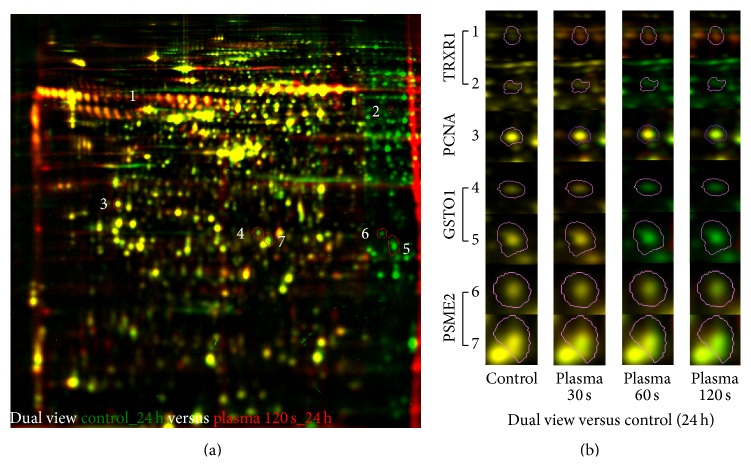
Dual channel images of whole proteome separated by 2D-DIGE (a) or selected protein spots as sections from 2D-DIGE gels (b). (a) Dual channel image of false coloured separated proteins of untreated (green) and 120 s TTP treated (red) S9 cells after 24 h. Protein spots with increased amounts induced by TTP exposure appear as red or orange spots, and those with decreased amounts as green spots. Spots, which are expressed with the same intensity on both gels, appear in yellow. Selected spots are numbered (1–7) and enlarged in (b). (b) Sections of selected protein spots 1–7 under different dose conditions underlining the phenomenon of multiple spots of several proteins. TRXR1 = thioredoxin-reductase 1; PCNA = proliferating cell nuclear antigen; GSTO1 = glutathione S-transferase omega 1; PSME2 = proteasome activator complex subunit 2.

To analyse possible changes in protein pattern caused by the TTP treatment, a 2D-DIGE approach comprising four conditions (untreated control, 30, 60, and 120 s TTP) and three time points after the TTP application (24 h, 48 h, and 72 h) was used. These time points should be most importantly for the evaluation of molecular changes on proteomic level if the doubling time of cells is 36 h and the macroscopic effects should happen after respective proteomic changes. Across the 72 resulting 2D-DIGE gel images 2094 different protein spots were clearly detected on the fusion gel and analysed using Delta2D statistical software tool for differential protein expression. The average abundances of spots were quantified and 1582 protein spots passed the one-way ANOVA test and could be determined as significantly regulated (supplementary Table S1). Those with relative changes in abundance greater than 1.5 times between control and plasma samples (up and down) at 95% confidence level (*p* ≤ 0.05) and identification by MALDI-MS were further considered (778 protein spots) (supplementary Table S2). In total, 553 unique proteins could be assigned to all samples with many proteins represented in multiple spots. Alterations in protein amounts between untreated and treated samples could be also demonstrated in dual view mode by Delta2D software (Decodon) as shown for one example in Figure 3(a). In several spots the same protein was identified by MALDI-MS, probably due to posttranslational modification whose relevance was not further examined in this analysis (Figure 3(b)). Further bioinformatical and statistical analyses of the identified regulated proteins of S9 epithelial cells highlighted the proteins of interest that are affected by TTP.

### 3.3. Principal Component Analysis (PCA) Uncovers Significant Treatment Effects

**Figure 4:**
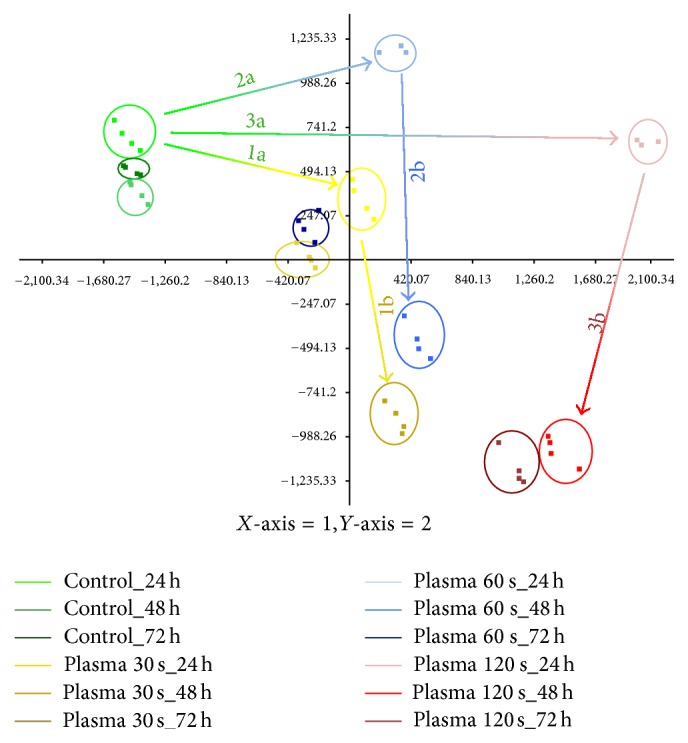
Principal component analyses (PCA) of proteomes of untreated and TTP treated S9 cells. The first and second principal components for untreated and TTP treated (30 s, 60 s, and 120 s) S9 epithelial cells after different points of time (24 h, 48 h, and 72 h) are shown. Each group consists of four technical replicates, except for the 24 h time point of 60 s and 120 s TTP treatment (only 3). The distances of shifts in space are illustrated by arrows. Arrows 1a, 2a, and 3a display the deviation of untreated samples and different treated samples after 24 h. Arrows 1b, 2b, and 3b represent the shift of treated samples after 24 h compared to their corresponding 48 h time point.

PCA was used to determine the variation within the proteomes and the significant effects of TTP treatment on the S9 epithelial cells proteome. The first and second principal components represented 47.7% of data variance (PC1: 33.3%, PC2: 14.4%) and distinguished all analysed samples in a very impressive way (Figure 4). After removing two outliers, it could be observed that all four technical replicates were tightly grouped, thus supporting the experimental reproducibility.

The proteomes of S9 cells which had undergone TTP treatment showed diverse distribution patterns depending on treatment dose and time point. The cellular proteomes of the control groups are closely clustered on the upper left side, with limited movement across the various time points. In contrast, 120 s TTP treated S9 cells are positioned on the right side of the coordinate system with long shifts among untreated and treated 24 h samples (arrow 3a) and between different time points (24 h versus 48 h, arrow 3b). The samples treated for 30 and 60 s are grouped between the others, as their proteomes differed in fact from those of untreated samples, but did not reach the distance of 120 s versus control samples. A comparison of the longest spatial distances between untreated and different treated samples after 24 h (arrows 1a, 2a, and 3a) or between 24 h and 48 h of each similar TTP treatment (1b, 2b, and 3b) clearly shows that the directions of the spatial shifts are similar, respectively, but the degree of shifts varied between the different doses of TTP (1a, 2a, and 3a) and time points (1b, 2b, and 3b) applied.

The PCA highlights that the highest dose of TTP treatment (120 s) after 24 h resulted in the greatest shifts between untreated and treated samples demonstrating the most significant effect on the proteome of S9 airway epithelial cells under these conditions.

### 3.4. TTP-Dose- and Time-Dependent Effects on the S9 Cell Proteome

**Figure 5:**
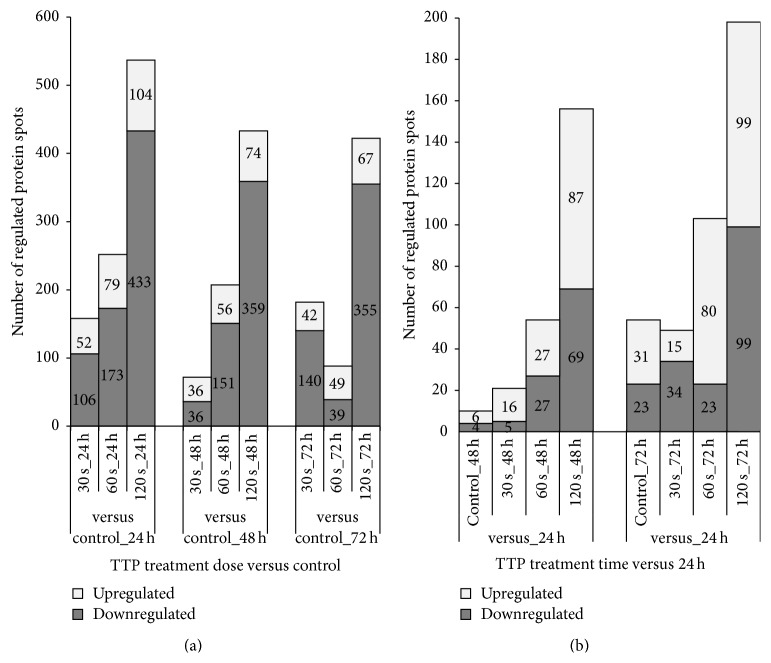
Number of TTP-dose- and time-dependent up- and downregulated protein spots in S9 epithelial cells. (a) In the TTP-dose-dependent approach, protein spots of all TTP doses (30 s, 60 s, and 120 s) were compared with untreated samples (control) after each time point (24 h, 48 h, and 72 h). The number of significantly up- and downregulated protein spots (±1.5-fold,* p* ≤ 0.05) is shown. Note that a TTP-dose-dependent increase of the number of regulated protein spots could be observed for each time with the greatest effect after 24 h. (b) In the time-dependent approach, protein spots of each of 48 h and 72 h time points were compared to the corresponding 24 h time points. The number of significantly up- and downregulated protein spots (±1.5-fold,* p* ≤ 0.05) is shown.

*TTP-dose-dependent effects*: The TTP-dose-dependent alterations in S9 cell protein expression with respect to up- and downregulated protein spots were examined and are shown in Figure 5(a). A total of 723 protein spots satisfied specific criteria (*p* < 0.05; fold change cut-off ±1.5; MS-identification) and could therefore be regarded as significantly regulated during at least one TTP-dose-time condition compared to untreated control samples. They correspond to 369 unique identified proteins.

As shown in Figure 5(a), the amount of up- and downregulated protein spots increased with raising TTP doses at each time point (one exception: 60 s/72 h). This dose-dependent correlation is developed most strongly after 24 h of TTP treatment. Thus, the strongest effect of regulation could be observed for 120 s TTP treatment after 24 h with 537 regulated protein spots (433 down- and 104 upregulated). This observed distinct dose-dependent effect of TTP on S9 airway epithelial cells supports the results of PCA analysis (see above). Among the top 10 of upregulated proteins after 120 s TTP treatment at 24 h are, for instance, GRP78 (Ca2+-binding, chaperone function, 5.7-fold), DCTN1 (axonal transport of vesicles and organelles, 4.4-fold), 2AAB (serine/threonine protein phosphatase, 4.2-fold), and the 4-fold induced DP13B protein which is involved in the regulation of cell proliferation.

*Time-dependent effects*: To analyse time-dependent alterations in S9 cell protein expression with respect to up- and downregulated protein spots, the proteomes of a specified dose of TTP treatment after 48 and 72 h were compared with those after 24 hours. A total of 323 protein spots fulfilled all mentioned criteria which could be allocated to 182 unique proteins. As shown in Figure 5(b), there is a time-dependent effect under all conditions, in untreated cells as well as in TTP treated samples. But whereas only a few proteins had changed their abundance time-dependently in untreated and 30 s TTP treated cells, significantly more proteins showed time-dependent alterations after the 60 and 120 s TTP treatment (Figure 5(b)). Hence, the highest TTP dose (120 s) resulted in the highest number of time-dependently regulated protein spots after 72 h.

A comparison of these time-dependently regulated protein spots (120 s, 72 h versus 120 s, 24 h) with dose-dependently regulated protein spots (120 s, 24 h) revealed a large overlay of protein spots that are repressed time-dependently after 72 h and induced by 120 s TTP dose after 24 h. Hence, from the 104 induced protein spots after 24 h, 57 showed a reduced protein level after 72 h (e.g., DP13B). Contrarily, from 433 repressed protein spots after 24 h, 97 showed an increased protein level after 72 h (e.g., PRDX3). This behaviour pointed out that a certain effect of recreation from TTP treatment has to exist.

**Figure 6:**
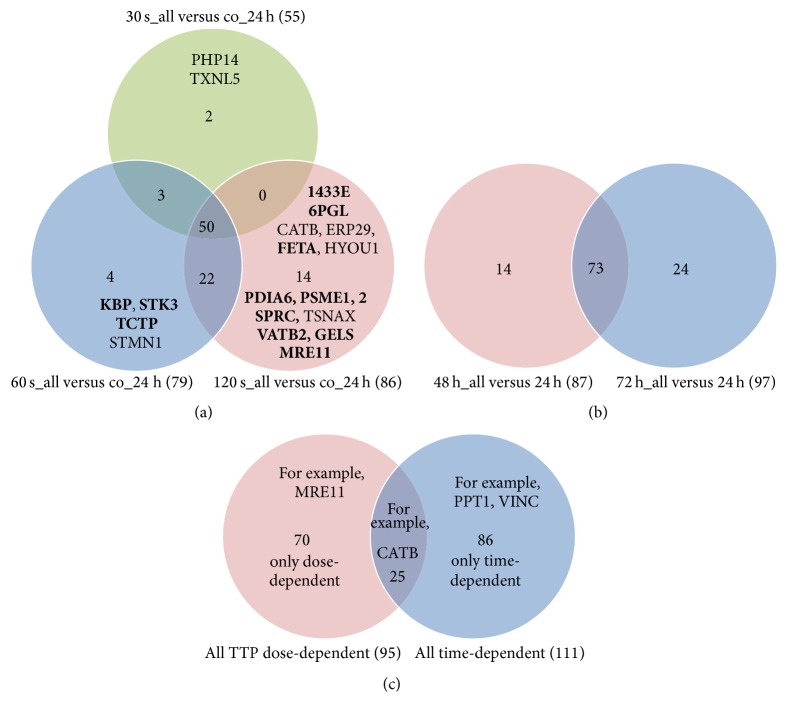
Comparison of upregulated proteins (TTP-dose and time-dependent) by VENN diagrams. (a) VENN diagram of all TTP-dose-dependently upregulated single proteins visualizing the overlapping results between upregulated proteins found in 30 s, 60 s, or 120 s TTP treated cells versus untreated cells (co). Proteins given in bold are only TTP-dose-dependently induced, and those given in light are induced also time-dependently. (b) VENN diagram of all time-dependently upregulated single proteins visualizing the overlapping results between upregulated proteins found in 48 h or 72 h incubated (TTP treated) cells versus 24 h incubated (TTP treated) cells. (c) VENN diagram of all TTP-dose- and time-dependently induced proteins indicating a fraction of proteins that are induced by both TTP dose and time span, as well as proteins that are solely regulated only by tissue-tolerable plasma or only over time.

*Comparison of TTP-dose- and time-dependently induced unique proteins*: A possible overlap of unique proteins regulated by more than one TTP dose or time point was not considered in Figure 5. To exemplify that there are really distinct TTP-dose- and time-specific protein alterations, we compared the upregulated unique proteins by means of VENN diagrams. Providing that mainly the function of upregulated proteins contributes to the observed macroscopic effect, we wanted to answer the following questions: (1) How many (and which) proteins are only induced by a special TTP dose, particularly by 120 s? (2) How many proteins are only induced by one time span? (3) How many proteins are only induced over time without any impact of TTP treatment? Figure 6(a) demonstrates that there is a TTP-dose-specific induction of proteins. Two proteins are only induced by the 30 s dose, four proteins are only upregulated in response to 60 s TTP, and fourteen proteins showed increased amounts only after the 120 s TTP treatment (e.g., PSME1 and 2, proteasome activator complex). The comparison of time-dependently induced proteins revealed that fourteen proteins are only induced after 48 h and twenty-four proteins are only upregulated later (72 h) (Figure 6(b)). To exclude the time-dependent effect, a comparison of TTP-dose-dependent induced proteins and time-dependently upregulated proteins was made (Figure 6(c)). Seventy proteins are solely induced by the TTP treatment and twenty-five proteins showed both increased amounts by the impact of tissue-tolerable plasma and by time period of 48 h or 72 h after the TTP treatment (e.g., CATB, catalase).

### 3.5. PANTHER Analysis of TTP-Dose-Dependently Regulated Proteins

**Figure 7:**
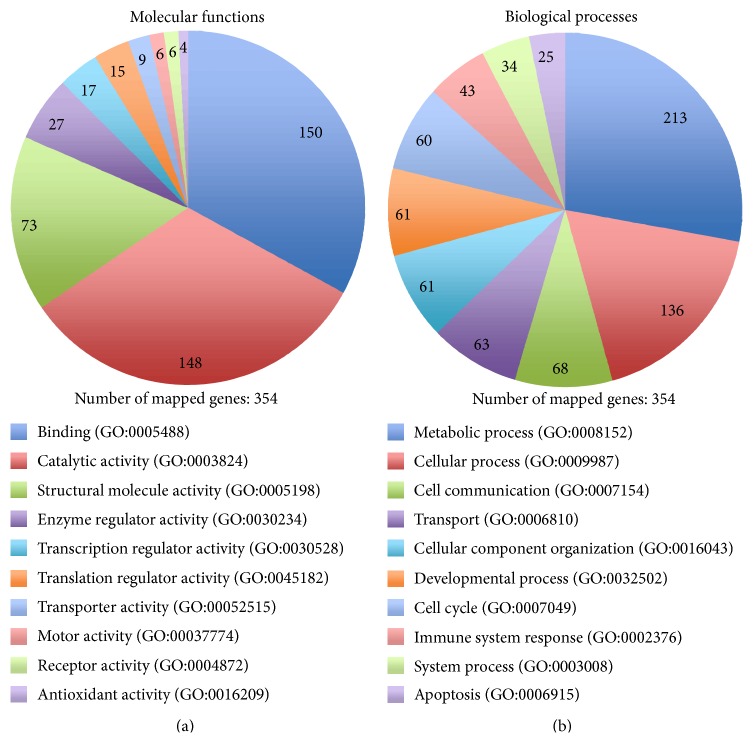
Pie charts showing assignments of regulated proteins of the dose-dependent approach to molecular functions (a) and biological processes (b). The charts were established by using PANTHER gene ontology. The number of genes that could be allocated to the corresponding function or process is marked. For the 369 significant regulated proteins (*p* < 0.05) 354 hits with allocations could be found. The standardized GO term is listed in brackets behind the function or process.

The gene ontology (GO) terms associated with the TTP-dose-dependently regulated proteins were examined using PANTHER. For 354 of the 369 identified proteins, 412 hits for molecular functions and 747 hits for biological processes could be found. Binding, catalytic, and structural molecule activity appear to be the most affected molecular functions (Figure 7(a)), while metabolic and cellular processes are the most influenced biological processes (Figure 7(b)). Consequently, PANTHER analyses revealed that observed dose-dependent proteome alterations applied mainly proteins which are involved in common cellular processes of binding and catalytic activity. The time-dependently affected proteins mainly function in protein expression and cell balance (data not shown).

### 3.6. Cellular Adaptation due to Different Doses of TTP Treatment as Revealed by IPA (Top Tox List and Top Molecular and Cellular Function List)

Pathways and networks, where TTP-dose-dependently regulated proteins are involved, were further analysed using Ingenuity Pathway Analysis (IPA) to determine the precise affected cellular pathways in response to TTP treatment. A ranking of the most determining stress factors “Top Tox List” and the most affected molecular functions “top molecular and cellular function” was established.

The “Top Tox List” (Table 1(a)) provides an overview of the adjustment of cell balance, displaying the five most affected stress response mechanisms for each condition, respectively.

At first sight, there is a significant change of influenced toxicity responses between 24 h after TTP treatment and the 48 h and 72 h time points, independently of applied doses, as several response mechanisms on one hand are strongly regulated after 24 h but not after 48 h and 72 h (e.g., “mitochondrial dysfunction,” “renal necrosis/cell death,” and “aryl hydrocarbon receptor signalling”) and on the other hand, other response mechanisms are strongly regulated after 48 h and 72 h but not after 24 h (e.g., “mechanisms of gene regulation by peroxisome proliferators via PPAR*α*,” “oxidative stress,” and “cell cycle : G2/M DNA checkpoint regulation”). “Nrf2-mediated oxidative stress” seems to be affected independently of TTP dose and time point but ranked first after 24 h.

The major part of toxicity responses is obviously linked to oxidative stress if “Nrf2-mediated oxidative stress” is the most affected response after 24 h for all TTP doses (rank 1) and “oxidative stress” seems to be the most affected response after 48 h and 72 h (ranks 1 and 2). Apart from these oxidative stress responses, “Decreases permeability transition of mitochondria and mitochondrial membrane”, which is affected by low TTP doses (30 and/or 60 s) at any time is a further marker for the presence of oxidative stress, as an increase of mitochondrial membrane permeability is an important initial step for apoptosis triggered by reactive oxygen species (ROS) [43]. The same is true for “increases transmembrane potential of mitochondria,” which is affected to rank four or five after 60 and 120 s TTP treatment [44, 45]. Additionally, the 30 s TTP dose regulated the “proteinuria-induced oxidative stress response.”

Besides oxidative stress responses, the regulation of “aryl hydrocarbon receptor signalling” at 24 h by 60 and 120 s doses indicated that cells furthermore had to prepare for xenobiotic and carcinogen detoxification due to higher doses of TTP treatment [46].

Proteins involved in the categories “mechanisms of gene regulation by peroxisome proliferators via PPAR*α*,” “PPAR*α*/RXR*α* activation,” and “PXR/RXR activation” were regulated by not exact referable TTP doses. Peroxisome proliferator-activated receptors (PPARs) have been discussed in context of initial wound healing phases [47].

In general, the analyses give a global overview of the major mediators of TTP treatment effects on cell balance. Oxidative stress is thereby one of the most determining factors.

The “top cellular and molecular function table” (Table 1(b)) provides an overview of cell adaption mechanisms, displaying the five most affected cellular pathways for each parameter. “Posttranslational modification,” “protein folding,” and “cell death” are the most regulated cellular functions, showing an increasing number of up- or downregulated proteins with increasing doses of TTP which was shown before in Figure 5(a).

The “cellular growth and proliferation” was mainly affected by the 120 s dose, whereas no regulation could be observed in response to the 30 s application. The sporadic affected expression of “DNA replication, recombination, and repair” indicated that TTP could also cause DNA damage.

### 3.7. Oxidative Stress Response and UV Damage Repair Proteins

**Figure 8:**
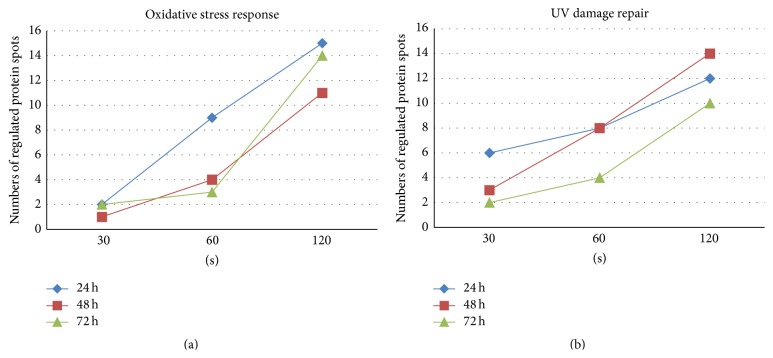
Progressive increase of the number of regulated protein spots involved in oxidative stress response and UV damage repair. Number of identified regulated protein spots involved in oxidative stress response (focus on detoxification of reactive oxygen species) (a) or UV damage repair (b) is displayed for different TTP doses after several time points. Note that increasing TTP doses progressively influences both adaptation pathways.

The oxidative stress response is one of the most determining toxicity factors. Besides reactive oxygen or nitrogen species (ROS or RNS), plasma contains also a certain fraction of UV radiation which could also induce the oxidative stress response as well as DNA damage. The sporadic affected expression of “DNA replication, recombination, and repair” could be one indication for DNA damage caused by TTP treatment. Therefore, a detailed view on single regulated proteins participating in oxidative stress response (focus on detoxification of reactive oxygen species) and UV damage repair was accomplished in order to support the IPA results. As shown in Figure 8 (for the whole protein list see supplementary Table S3), there is a TTP-dose-dependent regulation of both pathways with the highest number of regulated protein spots observed after the 120 s dose of TTP treatment during all time points. The largest TTP-dose-dependent regulating effect with respect to the number of affected protein spots could be found for oxidative stress response proteins after 24 h indicating an early, direct consequence of TTP treatment (Figure 8(a)). The impact of TTP on the regulation of UV damage repair proteins seems to be more lasting if the number of regulated protein spots was increased further after 48 h (Figure 8(b)).

As shown in supplementary Table S3, even a moderate dose of TTP (30 s) induced the expression of only two early oxidative stress response proteins, thioredoxin-reductase (TXRX1) and thioredoxin domain-containing protein 5 (TXND5). The 60 and 120 s TTP treatment significantly induced in a similar manner the expression of thioredoxin (THIO) itself. Additionally, reactive oxygen species- (ROS-) detoxification mechanisms seem to be affected, since superoxide dismutase (SODC), glutathione S-transferase (GSTO1), and peroxiredoxin (PRDX3, 2) were observed to be decreased in expression by 60 and 120 s TTP treatment although they are needed for detoxification of increased free radicals.

**Figure 9:**
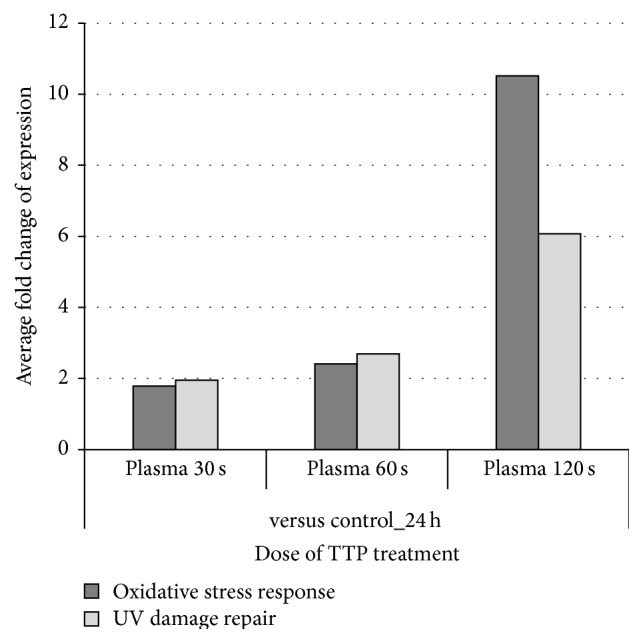
Effect of TTP treatment on the average fold change of protein spots involved in oxidative stress response and UV damage repair. The influence of different doses of TTP on oxidative stress response (focus on detoxification of reactive oxygen species) and UV damage repair is shown as average fold changes of all selected protein spots regardless of the fact that the single ratios have positive or negative values. For single ratios and their calculation see supplemental Table S3.

The UV excision repair protein RD23 homolog B (RD23B) showed a TTP-dose-dependent increasing induction after 24 h. An additional repair protein, the DNA double-strand break repair protein MRE11, was upregulated only by the 120 s dose (Table S3). As shown above, MRE11 was solely upregulated by the impact of TTP, not over time (see Figure 6(c)). In summary, this detailed analysis revealed a strong dose-dependent increase of the number of regulated oxidative stress proteins after 24 h indicating a direct impact on molecular adaptation mechanisms. The impact of UV radiation on cellular pathways could be observed as more lasting compared to oxidative stress. Furthermore, a strong dose-dependent increase of average fold changes is shown for both adaptation pathways in Figure 9.

### 3.8. Proteins Associated with Cell Death/Apoptosis and Cell Proliferation

“Cell death” and “cellular growth and proliferation” were under the top of molecular and cellular functions affected by TTP. In order to get a closer view on single regulated proteins participating in these pathways, a more detailed analysis of the subgroups “antiapoptotic factors,” “cell death/apoptotic factors,” and “cell proliferation/cell division” was accomplished and should support the present IPA results.

**Figure 10:**
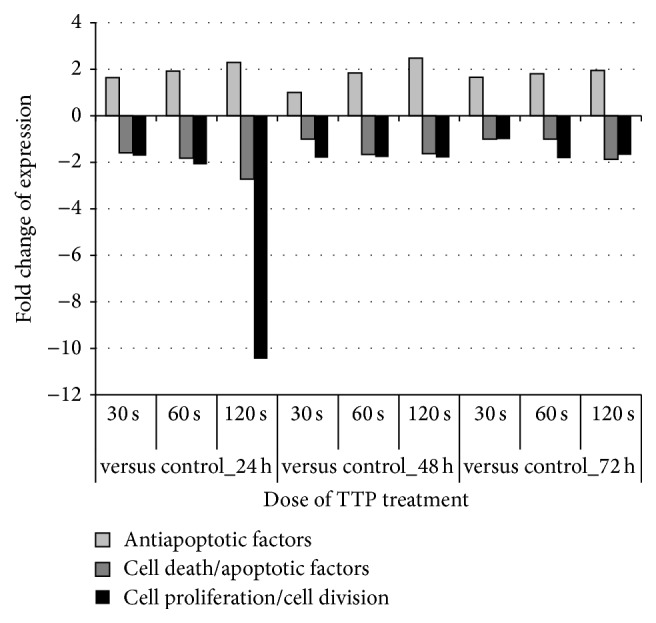
Effects of TTP treatment on cell death and cell proliferation pathways of S9 epithelial cells. The influence of different TTP doses on cell death/apoptosis and cell proliferation pathways is shown as average fold changes of all selected protein spots taking into account the fact that single ratios have positive or negative values. For single ratios and their calculation see supplemental Table S4.

An increasing number of cell death/apoptotic factors and protein spots functioning in cell proliferation/cell division showed reduced protein levels in S9 cells correlating with increasing TTP doses at any time point. The average fold changes of respective normalized values of each condition are shown in Figure 10. For the whole protein list see supplementary Table S4. There is a clear TTP-dose-dependent suppression after 24 h if the reduction of protein amount was highest after 120 s TTP treatment at this time point. In particular, the amount of one protein, the proliferation-associated protein 2G4 (PA2G4), was strongly decreased leading to an average expression value of about 10 for the whole group of “cell proliferation/cell division” proteins. If this effect could be observed only after 24 h, then it should be more directly linked to the given TTP stimulus.

In contrast, protein levels of antiapoptotic factors are increased up to 2.5-fold in a dose-dependent manner during all time points indicating lasting impact of TTP on regulation of respective proteins.

Taking both observations into account, it can be assumed that TTP treatment has regulating effects on cell death and apoptosis mechanisms, thus leading to longer cell survivability.

## 4. Discussion

Many recent papers have highlighted the promising potential of plasma treatment as a new therapeutic option for chronic infected wound care. In several* in vitro* studies, different types of wound relating cells in monolayer, such as keratinocytes, fibroblasts, breast epithelial, and endothelial cells, were used to analyse the different effects of plasma application [19, 22, 24, 25]. Despite these* in vitro* studies, the fundamental nature of the interaction between plasma and human cells is to a large extent unknown. But a detailed knowledge of these interactions is essential for the evaluation of plasma effects in relation to wound healing and the establishment of new therapeutic plasma tools. In this study we analysed for the first time plasma effects on cellular adaptation of upper airway epithelial S9 cells. The standardized wound model employed in this study simulates* in vitro* wound healing in secondary intention, for example, after surgery of the paranasal sinus, the larynx, or the trachea. As a consequence, results obtained here have a direct clinical impact for the medical application of cold plasma in internal medicine (e.g., during bronchoscopy), thoracic surgery, and otorhinolaryngology.

Furthermore, molecular changes due to plasma treatment were studied for the first time at the proteome level in order to complement the known macroscopic effects observed in the wound model. First, tissue-tolerable plasma (TTP) doses were determined in an* in vitro* wound healing model with S9 epithelial cells. Plasma doses from 30 s up to 360 s were tested in relation to wound regeneration after 24 h, 48 h, 72 h, 96 h, and 120 h, in which lower doses (30, 60, and 120 s) resulted in dose-dependent improved wound healing rate compared to untreated cells. Thereby, the 120 s dose caused significantly the best wound healing properties after 96 and 120 h. Because higher doses (240, 360 s) resulted in lowered or even blocked regeneration, only the lower plasma doses were termed as tissue-tolerable plasma (TTP) and used for the investigation of proteome alterations in the following. Thus, the proteomes of 30, 60, and 120 s TTP treated S9 cells were analysed and compared with untreated cells. Assuming that proteome alterations in the following experiments and molecular adaptation should happen before the development of the observed macroscopic effect, the analysis focused on cells harvested after 24, 48, and 72 h.

In accordance with the PCA, the detailed statistical proteome analysis highlights that the highest dose of TTP treatment (120 s) after 24 h resulted in the greatest proteome alterations between untreated and treated samples demonstrating the most significant effect on the proteome of S9 airway epithelial cells under these conditions. If the 120 s dose caused the best wound healing properties and the most significant changes at the proteome level, then the question arises if these molecular changes could be the reason for the observed macroscopic effect. Which cellular and molecular mechanisms could be responsible for the improved wound healing properties?

As shown by IPA, various toxic responses or cellular and molecular functions are affected by TTP treatment in terms of up- or downregulation of their associated proteins. Undoubtedly, the most toxicity responses are linked to oxidative stress response emphasizing oxidative stress as a possible key event in the regeneration process of epithelial cells as well as in the adaptation to plasma exposure. Oxidative stress might occur as a direct outcome of the existence of reactive oxygen or nitrogen species (ROS, RNS) which are detectable in the gas phase over the cells as well as in the culture medium after the argon plasma treatment. These species can also enter the cells possibly by diffusion or can induce new species within the cells [24]. It might be that the impact of ROS or RNS and the initiation of oxidative stress response are a necessary prerequisite for initiation of wound healing.

The significant influence of several oxidative stress response pathways is further supported by our single protein analyses, which revealed partial progressive regulation of downstream proteins involved in oxidative stress response, particularly in the detoxification of reactive oxygen species. The upregulation of thioredoxin-reductase and thioredoxin itself might be a clear evidence for the already existing defence against oxidative damage or at least for the signalling of molecules like hydrogen peroxide or nitric oxide. Even though the amounts of further detoxification enzymes like superoxide dismutase and peroxiredoxin were decreased relatively, an involvement of these enzymes in the active defence against oxidative stress can be assumed. We suppose that the oxidative stress elucidated by our TTP treatment is dosed as much as required for initiation of wound healing but low enough to prevent profound cell damage. The induction of oxidative stress due to plasma treatment has also been described for other cell types, both human [31, 48–50] and animal cells [51, 52]. In contrast to many other studies (e.g., 50), our proteomic approach was designed not to detect ROS or RNS but to detect many downstream regulated proteins, providing a new perspective on plasma treatment effects. The pivotal role of oxidative stress in the wound healing process can be regarded as independently of organism or cell type, as revealed by both* in vitro* and* in vivo* settings [53–55].

The wound healing process of acute wounds is an intricate interplay between several cell types involving various forms of intracellular signalling and classical wound healing phases like haemostasis, inflammation, and new-tissue formation. The last one includes, for example, keratinocytes migrating and proliferating into the wound [56]. Apoptosis of immune cells may be the major key to end inflammation and to initiate the healing [57]. Our proteomic study analysing only a single epithelial cell type could demonstrate the induction of antiapoptotic factors as well as the repression of proteins involved in apoptosis indicating that apoptosis seems to be inhibited by our TTP treatment in S9 cells. However, TTP treatment caused contrary effects on cells, because it inhibits apoptotic mechanisms on one hand and cell proliferation on the other hand. Although the majority of protein hits associated with cell proliferation and cell division showed reduced protein levels mainly 24 h after the 120 s TTP treatment (e.g., PA2G4), we cannot exclude a certain proliferation and migration activity leading to the observed wound regeneration after 96 and 120 h. In accordance with this assumption, the proteomic approach revealed that the degree of repression of these proteins is highest after 24 h but is clearly reduced after 48 and 72 h, indicated by a decreased number of affected proteins and decreased repression factors. Furthermore, the DP13B (APPL2) protein, involved in regulation of cell proliferation, belongs to the top 10 of upregulated proteins in response to 120 s plasma dose.

The apparently concurrent repression of apoptosis and cell proliferation might be an indication for the coexistence of both successful and failed cell adaptation in response to TTP treatment. Here, we have to underline that our proteomic approach could only detect the sum of all cell events of each culture plate including intact as well as injured cells representing all molecular mechanisms that are responsible for the final concentration of each protein. It might be that there are some cells, immediately bordered on the wound, which activate the cell proliferation/cell division at least to a certain extent.

Positive effects of plasma treatment on wound healing have also been shown several times in many studies [17, 18, 21, 25]. However, the positive effects were mostly ascribed to a decrease of bacterial load in chronic wounds [18, 21]. Only recently, different studies focusing on epithelial cells demonstrated that certain plasma doses increased cell proliferation in treated cells but also activated apoptotic pathways [24, 29–31]. In another very recently published study with keratinocytes, a significant role of apoptosis after plasma treatment was excluded [50]. Further studies showed that plasma can support wound healing not only by its antiseptic effects but also by the stimulation of proliferation and migration of wound relating skin cells or by its proangiogenic effect (reviewed in [24]).

In the current proteome study we analysed for the first time a possible correlation between detected proteome alterations, due to treatment with three different TTP doses, and wound healing process without an involvement of infection.

To some extent, these first detected proteomic effects in S9 epithelial cells are comparable with the sole transcriptomic studies carried out with indirectly plasma treated HaCaT keratinocyte cells [31, 32, 50]. Similar biological pathways, such as oxidative stress response, repair, cell, death, and proliferation, have been found as effected by both approaches. Both proteomic and transcriptomic studies revealed that, for the present, plasma treatment has to be considered as double-edged sword yet. For example, our results indicate that TTP treatment is probably not just affecting molecular mechanisms during the time of application or rather over one period of cell division but has in fact lasting impacts on protein expression and cell balance. A more detailed time-resolved view including also shorter times immediately after the application of TTP should apparently demonstrate that (1) the TTP dose affects the degree of molecular response and that (2) posttranslational modifications of proteins may appear as a consequence of oxidative stress (Emicke et al., in preparation).

We were able to show that the effects of TTP treatment on cells are highly dependent on the dose applied. The* in vitro* wound model revealed an increasing wound healing rate with increasing doses of TTP treatment. Our proteome analyses uncovered a TTP-dose-dependent increase of the number of regulated proteins functioning in different toxicity responses and biological pathways. Particularly after 24 h, the repression rate of proteins associated with apoptosis and cell proliferation is increased. This highlights the need of finding the optimal treatment dose to provide a balance between cell proliferation and death before it could be safely and routinely introduced in clinical use. In the current experimental setup, the 120 s dose of TTP treatment may be the optimal dose, if apoptotic pathways do not at least counteract with cell proliferation. Precaution should be used, as long term effects were not analysed so far and therefore the risk of cumulative cell damage could not be evaluated surely. In recent clinical trials, a dose of 120 s cold plasma treatment was set as standard, since beneficial macroscopic effects could be observed [21, 26, 48, 58–60]. Both studies reported an enhanced wound healing rate on skin graft donor sites or chronic ulcers without adverse effects for patients; however, long term effects were not evaluated. Lademann et al. [61] and Kramer et al. [33] focused on the suitability and risk assessment of plasma on human skin. Both concluded that plasma treatment entails no risk for humans. Nevertheless, our current study showed at proteome level that cell damaging effects of TTP treatment on airway epithelial cells should not be disregarded. Future studies should combine clinical trials with a molecular approach in order to achieve a comprehensive understanding of tissue-tolerable plasma.

The possible cell damaging effects of TTP treatment not only may be a result of oxidative stress, but also may be attributed to UV damage. Although no hints were obtained from top toxicity lists and associated molecular functions and biological processes, single protein analyses showed a TTP-dose-dependent regulation of UV damage repair mechanisms for all time points (e.g., upregulation of RD23B and MRE11). This could be an underestimated source of long term cell damage due to TTP treatment. The possible role of UV radiation due to plasma treatment on cells was recently discussed. Irradiation of cell cultures can lead to severe DNA damage and apoptosis, but moderate doses are able to activate UV damage response and trigger adaptation pathways which lead to enhanced cell proliferation [62, 63]. Stoffels [63] reasoned that cells appear to be fairly resistant against UV radiation, if activated response mechanisms are sufficient to cope with the imposed challenges. The possible UV damage caused by plasma treatment is still persisting as a critical and highly discussed point for plasma researchers. The current study indicates that UV damage is probably subordinate to oxidative stress regarding the effects of TTP treatment, at least under the investigated conditions. But long term research should be performed in order to evaluate the enduring effects of both UV damage and oxidative stress. Additionally, the more detailed time resolved view in the accompanying study of Emicke et al. (in preparation) should highlight the role of NRF2 in oxidative stress response and the identification of Nrf2 targets if “Nrf2 mediated oxidative stress” was found as a top of toxicity responses by our proteomic approach.

Last but not least, one limitation of this study has to be taken into consideration. Cell cultures were manually treated with TTP from the plasma jet “kINPen” by following a grid pattern for the required time (30 s, 60 s, and 120 s). In order to achieve equalization of treatment time for all cell cultures, six time checkpoints were set along the grid pattern. Nevertheless, absolute exactness of the manual procedure cannot be guaranteed. Now, a new plasma source generation has been developed by the INP Greifswald, which is able to treat cell cultures automatically for a specified time, following a defined pattern. For future studies, the automated application would be recommended to avoid any variation in application of treatment. Furthermore, it is necessary to note that the results gathered from cell culture analyses with only one single cell type cannot simply be transferred into interacting physiology or extrapolated for other cell types or complex organs. For example, the S9 airway epithelial cells may be more resistant to TTP treatment and may show different adaptation mechanisms than other human cells or even animal cells. In the future, the aim of comprehensive molecular research should be to combine* in vitro* and* in vivo* macroscopic and molecular analysis [64]. However, this first proteomic analysis indicates dose-dependent cellular adaptation in response to TTP treatment and therefore supports the results of the* in vitro* wound model.

## 5. Conclusion

This proteomic study provides an overall insight to cell adaptation pathways of S9 epithelial cells induced by TTP treatment. At the proteome level, oxidative stress could be shown to be the most affected response of TTP treatment and the possible key mediator for initiation of wound healing. Although this study gives reason to assume that oxidative stress is the greatest challenge for cells to cope with, the role of radiation should not be disregarded. Therefore, further analyses have to be performed in order to receive more detailed knowledge. Furthermore, results of an* in vitro* wound model highlight the dose-dependent wound healing accelerating effects of TTP treatment. Treatment of cells for 30 s and 60 s showed marginal beneficial effects on cell regeneration, whereas 120 s improved the wound healing rate by nearly 15% compared to untreated cells. Additionally, analyses on protein level supported the wound model, as alterations in protein expression profiles increased with the applied dose and cell proliferation pathways were TTP-dose-dependently regulated. However, higher doses of TTP seem to be also a more potent cause for cellular imbalance, leading to increased repression rate of proteins functioning in cell death/apoptosis and cell proliferation. Hence,* in vivo* wound models should be performed in order to evaluate the toxicity of TTP treatment for animal or human physiology. This study confirms that proteome analyses are a powerful tool to achieve molecular understanding about well-known macroscopic TTP treatment effects and should be implemented in clinical trials to learn about strengths and weaknesses of plasma in human medicine.

## Supplementary Material

Supplementary Tables:
- Table S-1: Register of all 1582 significantly regulated protein spots, including ratios and p-values
- Table S-2: Register of all 778 identified protein spots including ratios used for further analyses.
- Table S-3: Register of selected protein spots functioning in oxidative stress response (focus on detoxification of reactive oxygen species) and UV damage repair including ratios.
- Table S-4: Register of selected protein spots functioning in cell death and cell proliferation.
Supplementary Figure:
- Figure S-1: Supporting information about the non-thermal plasma source and the technical procedure

## Figures and Tables

**Table 1a:** (a) The “Top Tox List” displays the response mechanisms, which are most affected by several stress factors emerging due to TTP treatment. The top five response mechanisms are listed for every dose (30 s, 60 s, and 120 s) after every time point (24 h, 48 h, and 72 h). Numbers (1–5) represent the ranking of the response mechanisms for every parameter

Top Tox List	24 h			48 h			72 h		
	30 s	60 s	120 s	30 s	60 s	120 s	30 s	60 s	120 s
Nrf2-mediated oxidative stress	1	1	1		4	3	3		3
Decreases permeability transition of mitochondrial membrane	2			1			5	1	
Mitochondrial dysfunction	3								
Response to proteinuria-induced oxidative stress in renal proximal tubulus cells	4			2					
Increases bradycardia	5			5					
Renal necrosis/cell death		2	3						
Aryl hydrocarbon receptor signaling		3	2						
PPAR*α*/RXR*α* activation		4			3			4	
Increases transmembrane potential of mitochondrial membrane		5	5			4			5
Xenobiotic metabolism signaling			4						
Mechanisms of gene regulation by peroxisome proliferators via PPAR*α*				3	1	2	4		
Increases renal proliferation				4					
Oxidative stress					2	1	1	2	2
PXR/RXR activation					5			3	
Cell cycle: G2/M DNA checkpoint regulation						5			1
TR/RXR activation							2	5	
Acute renal failure panel									4

**Table 1b:** (b) The chart displays the most influenced molecular and cellular functions after 30 s, 60 s, and 120 s of TTP treatment and incubation time of 24 h, 48 h, and 72 h. Numbers represent the ranking of the top five cellular functions for every parameter

Top molecular and cellular functions	24 h			48 h			72 h		
	30 s	60 s	120 s	30 s	60 s	120 s	30 s	60 s	120 s
Posttranslational modification	1	1	1	1	1	2	1	5	1
Protein folding	2	3	2	2	3	4	3	4	3
Cell death	3	2	3		4	1	2	1	2
Cellular assembly and organization	4			4			4	2	
Cellular movement	5			3			5		
DNA replication, recombination, and repair		4							5
Cellular growth and proliferation		5	4			3			4
Cellular compromise			5		2	5			
Cellular function and maintenance				5	3			3	

## Abbreviations

TTP: Tissue-tolerable plasma
ROS: Reactive oxygen species
RNS: Reactive nitrogen species
2D-DIGE: Two-dimensional-difference gel electrophoresis
Plasma dose: Treatment time.

## References

[1] Treutler C. P. O. (2005). Industrial use of plasma-deposited coatings for components of automotive fuel injection systems. *Surface and Coatings Technology*.

[2] Chae J.-O. (2003). Non-thermal plasma for diesel exhaust treatment. *Journal of Electrostatics*.

[3] Morent R., de Geyter N., Verschuren J., de Clerck K., Kiekens P., Leys C. (2008). Non-thermal plasma treatment of textiles. *Surface and Coatings Technology*.

[4] Moreau E. (2007). Airflow control by non-thermal plasma actuators. *Journal of Physics D: Applied Physics*.

[5] Canard J. M., Védrenne B. (2001). Clinical application of argon plasma coagulation in gastrointestinal endoscopy: has the time come to replace the laser?. *Endoscopy*.

[6] Pereira-Lima J. C., Busnello J. V., Saul C. (2000). High power setting argon plasma coagulation for the eradication of Barrett's esophagus. *The American Journal of Gastroenterology*.

[7] Raiser J., Zenker M. (2006). Argon plasma coagulation for open surgical and endoscopic applications: state of the art. *Journal of Physics D: Applied Physics*.

[8] Brand C. U., Blum A., Schlegel A., Farin G., Garbe C. (1998). Application of argon plasma coagulation in skin surgery. *Dermatology*.

[9] Shi X.-M., Zhang G.-J., Yuan Y.-K., Ma Y., Xu G.-M., Yang Y. (2008). Effects of low-temperature atmospheric air plasmas on the activity and function of human lymphocytes. *Plasma Processes and Polymers*.

[10] Haertel B., Volkmann F., von Woedtke T., Lindequist U. (2012). Differential sensitivity of lymphocyte subpopulations to non-thermal atmospheric-pressure plasma. *Immunobiology*.

[11] Bundscherer L., Wende K., Ottmüller K. (2013). Impact of non-thermal plasma treatment on MAPK signaling pathways of human immune cell lines. *Immunobiology*.

[12] Kim D., Gweon B., Kim D. B., Choe W., Shin J. H., Lim C., Goh J. (2009). A feasibility study for the cancer therapy using cold plasma. *13th International Conference on Biomedical Engineering*.

[13] Partecke L. I., Evert K., Haugk J. (2012). Tissue tolerable plasma (TTP) induces apoptosis in pancreatic cancer cells in vitro and in vivo. *BMC Cancer*.

[14] Kim C.-H., Bahn J. H., Lee S.-H. (2010). Induction of cell growth arrest by atmospheric non-thermal plasma in colorectal cancer cells. *Journal of Biotechnology*.

[15] Fridman G., Shereshevsky A., Jost M. M. (2007). Floating electrode dielectric barrier discharge plasma in air promoting apoptotic behavior in Melanoma skin cancer cell lines. *Plasma Chemistry and Plasma Processing*.

[16] Vandamme M., Robert E., Lerondel S. (2012). ROS implication in a new antitumor strategy based on non-thermal plasma. *International Journal of Cancer*.

[17] Bender C. P., Hübner N., Weltmann K., Scharf C., Kramer A., Machala Z., Hensel K., Akishev Y. (2012). Tissue tolerable plasma and polihexanide: are synergistic effects possible to promote healing of chronic wounds? In vivo and in vitro results. *Plasma for Bio-Decontamination, Medicine and Food Security*.

[18] Isbary G., Morfill G., Schmidt H. U. (2010). A first prospective randomized controlled trial to decrease bacterial load using cold atmospheric argon plasma on chronic wounds in patients. *British Journal of Dermatology*.

[19] Arndt S., Unger P., Wacker E. (2013). Cold atmospheric plasma (CAP) changes gene expression of key molecules of the wound healing machinery and improves wound healing in vitro and in vivo. *PLoS ONE*.

[20] Nuutila K. (2013). *Gene expression profiling of human skin donor site wound healing to guide novel regenerative therapies [Ph.D. thesis]*.

[21] Isbary G., Heinlin J., Shimizu T. (2012). Successful and safe use of 2 min cold atmospheric argon plasma in chronic wounds: results of a randomized controlled trial. *The British Journal of Dermatology*.

[22] Heinlin J., Isbary G., Stolz W. (2011). Plasma applications in medicine with a special focus on dermatology. *Journal of the European Academy of Dermatology and Venereology*.

[23] Wiegand C., Beier O., Horn K. (2013). Antimicrobial impact of cold atmospheric pressure plasma on medical critical yeasts and bacteria cultures. *Skin Pharmacology and Physiology*.

[24] Haertel B., von Woedtke T., Weltmann K.-D., Lindequist U. (2014). Non-thermal atmospheric-pressure plasma possible application in wound healing. *Biomolecules & Therapeutics*.

[25] Isbary G., Stolz W., Shimizu T. (2013). Cold atmospheric argon plasma treatment may accelerate wound healing in chronic wounds: results of an open retrospective randomized controlled study in vivo. *Clinical Plasma Medicine*.

[26] Heinlin J., Zimmermann J. L., Zeman F. (2013). Randomized placebo-controlled human pilot study of cold atmospheric argon plasma on skin graft donor sites. *Wound Repair and Regeneration*.

[27] Dobrynin D., Fridman G., Friedman G., Fridman A. (2009). Physical and biological mechanisms of direct plasma interaction with living tissue. *New Journal of Physics*.

[28] Schneider S., Lackmann J.-W., Narberhaus F., Bandow J. E., Denis B., Benedikt J. (2011). Separation of VUV/UV photons and reactive particles in the effluent of a He/O_2_ atmospheric pressure plasma jet. *Journal of Physics D: Applied Physics*.

[29] Haertel B., Hähnel M., Blackert S., Wende K., von Woedtke T., Lindequist U. (2012). Surface molecules on HaCaT keratinocytes after interaction with non-thermal atmospheric pressure plasma. *Cell Biology International*.

[30] Blackert S., Haertel B., Wende K., von Woedtke T., Lindequist U. (2013). Influence of non-thermal atmospheric pressure plasma on cellular structures and processes in human keratinocytes (HaCaT). *Journal of Dermatological Science*.

[31] Schmidt A., Wende K., Bekeschus S. (2013). Non-thermal plasma treatment is associated with changes in transcriptome of human epithelial skin cells. *Free Radical Research*.

[32] Schmidt A., von Woedtke T., Weltmann K.-D., Masur K. (2013). Identification of the molecular basis of non-thermal plasma-induced changes in human keratinocytes. *Plasma Medicine*.

[33] Kramer A., Lademann J., Bender C. (2013). Suitability of tissue tolerable plasmas (TTP) for the management of chronic wounds. *Clinical Plasma Medicine*.

[34] Zeitlin P. L., Lu L., Rhim J. (1991). A cystic fibrosis bronchial epithelial cell line: immortalization by adeno-12-SV40 infection. *American Journal of Respiratory Cell and Molecular Biology*.

[35] Beule A. G., Athanasiadis T., Field J., Hosemann W., Wormald P.-J., Tan L. W. (2010). Effects of simulated bleeding in an in vitro nasal fibroblast wound healing model. *American Journal of Rhinology & Allergy*.

[36] Roth C., Beule A. G., Kramer A., Hosemann W., Kohlmann T., Scharf C. (2010). Response analysis of stimulating efficacy of polihexanide in an in vitro wound model with respiratory ciliary epithelial cells. *Skin Pharmacology and Physiology*.

[37] Bradford M. M. (1976). A rapid and sensitive method for the quantitation of microgram quantities of protein utilizing the principle of protein-dye binding. *Analytical Biochemistry*.

[38] Thiele T., Steil L., Gebhard S. (2007). Profiling of alterations in platelet proteins during storage of platelet concentrates. *Transfusion*.

[39] Luhn S., Berth M., Hecker M., Bernhard J. (2003). Using standard positions and image fusion to create proteome maps from collections of two-dimensional gel electrophoresis images. *Proteomics*.

[40] Dunteman G. H. (1989). *Principal Components Analysis*.

[41] Eymann C., Dreisbach A., Albrecht D. (2004). A comprehensive proteome map of growing *Bacillus subtilis* cells. *Proteomics*.

[42] Thomas P. D., Kejariwal A., Campbell M. J. (2003). PANTHER: a browsable database of gene products organized by biological function, using curated protein family and subfamily classification. *Nucleic Acids Research*.

[43] Skulachev V. P. (1996). Why are mitochondria involved in apoptosis? Permeability transition pores and apoptosis as selective mechanisms to eliminate superoxide-producing mitochondria and cell. *FEBS Letters*.

[44] Tsujimoto Y., Shimizu S. (2007). Role of the mitochondrial membrane permeability transition in cell death. *Apoptosis*.

[45] Ly J. D., Grubb D. R., Lawen A. (2003). The mitochondrial membrane potential (*δψ*m) in apoptosis; an update. *Apoptosis*.

[46] Miao W., Hu L., Scrivens P. J., Batist G. (2005). Transcriptional regulation of NF-E2 p45-related factor (NRF2) expression by the aryl hydrocarbon receptor-xenobiotic response element signaling pathway: direct cross-talk between phase I and II drug-metabolizing enzymes. *The Journal of Biological Chemistry*.

[47] Michalik L., Wahli W. (2007). Peroxisome proliferator-activated receptors (PPARs) in skin health, repair and disease. *Biochimica et Biophysica Acta—Molecular and Cell Biology of Lipids*.

[48] Fluhr J. W., Sassning S., Lademann O. (2012). *In vivo* skin treatment with tissue-tolerable plasma influences skin physiology and antioxidant profile in human stratum corneum. *Experimental Dermatology*.

[49] Kalghatgi S., Kelly C. M., Cerchar E. (2011). Effects of non-thermal plasma on mammalian cells. *PLoS ONE*.

[50] Schmidt A., Dietrich S., Steuer A. (2015). Non-thermal plasma activates human keratinocytes by stimulation of antioxidant and phase II pathways. *The Journal of Biological Chemistry*.

[51] Nastuta A. V., Topala I., Grigoras C., Pohoata V., Popa G. (2011). Stimulation of wound healing by helium atmospheric pressure plasma treatment. *Journal of Physics D: Applied Physics*.

[52] García-Alcantara E., López-Callejas R., Morales-Ramírez P. R. (2013). Accelerated mice skin acute wound healing in vivo by combined treatment of argon and helium plasma needle. *Archives of Medical Research*.

[53] Soneja A., Drews M., Malinski T. (2005). Role of nitric oxide, nitroxidative and oxidative stress in wound healing. *Pharmacological Reports*.

[54] Schäfer M., Werner S. (2008). Oxidative stress in normal and impaired wound repair. *Pharmacological Research*.

[55] Loo A. E. K., Wong Y. T., Ho R. (2012). Effects of hydrogen peroxide on wound healing in mice in relation to oxidative damage. *PLoS ONE*.

[56] Martin P. (1997). Wound healing—aiming for perfect skin regeneration. *Science*.

[57] Wu Y.-S., Chen S.-N. (2014). Apoptotic cell: linkage of inflammation and wound healing. *Frontiers in Pharmacology*.

[58] Dobrynin D., Wu A., Kalghatgi S. (2011). Live pig skin tissue and wound toxicity of cold plasma treatment. *Plasma Medicine*.

[59] Daeschlein G., Scholz S., Ahmed R. (2012). Cold plasma is well-tolerated and does not disturb skin barrier or reduce skin moisture. *Journal of the German Society of Dermatology*.

[60] Brehmer F., Haenssle H. A., Daeschlein G. (2015). Alleviation of chronic venous leg ulcers with a hand-held dielectric barrier discharge plasma generator (PlasmaDerm VU-2010): results of a monocentric, two-armed, open, prospective, randomized and controlled trial (NCT01415622). *Journal of the European Academy of Dermatology and Venereology*.

[61] Lademann J., Richter H., Alborova A. (2009). Risk assessment of the application of a plasma jet in dermatology. *Journal of Biomedical Optics*.

[62] Sosnin E. A., Stoffels E., Erofeev M. V., Kieft I. E., Kunts S. E. (2004). The effects of UV irradiation and gas plasma treatment on living mammalian cells and bacteria: a comparative approach. *IEEE Transactions on Plasma Science*.

[63] Stoffels E. (2007). ‘Tissue processing’ with atmospheric plasmas. *Contributions to Plasma Physics*.

[64] Arndt S., Landthaler M., Zimmermann J. L. (2015). Effects of cold atmospheric plasma (CAP) on *β*-defensins, inflammatory cytokines, and apoptosis-related molecules in keratinocytes in vitro and in vivo. *PLoS ONE*.

