# Supplementary material for: Proteomic Changes of Tissue-Tolerable Plasma Treated Airway Epithelial Cells and Their Relation to Wound Healing
Source: Biomed Res Int. 2015 Oct 11;2015:506059. doi: 10.1155/2015/506059 (PMC4619824; doi:10.1155/2015/506059)
Supplement: Supplementary file 1 — Supplementary Tables: - Table S-1: Register of all 1582 significantly regulated protein spots, including ratios and p-values - Table S-2: Register of all 778 identified protein spots including ratios used for further analyses. - Table S-3: Register of selected protein spots functioning in oxidative stress response (focus on detoxification of reactive oxygen species) and UV damage repair including ratios. - Table S-4: Register of selected protein spots functioning in cell death and cell proliferation. Supplementary Figure: - Figure S-1: Supporting information about the non-thermal plasma source and the technical procedure [file 506059.f1.pdf]

Lendeckel et al.:
Proteomic changes of tissue-tolerable plasma (TTP) treated airway epithelial cells and their relation to wound healing

Supplementary Figure S-1

A

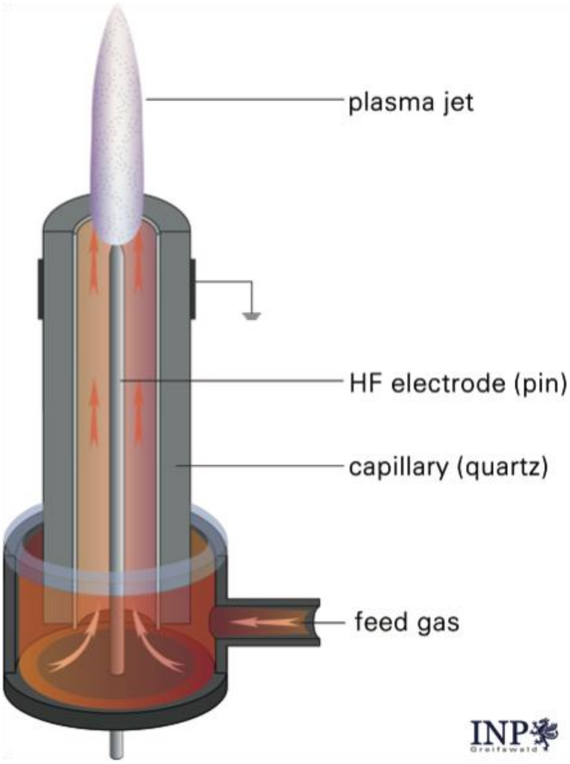
plasma jet
HF electrode (pin)
capillary (quartz)
feed gas
INP

B

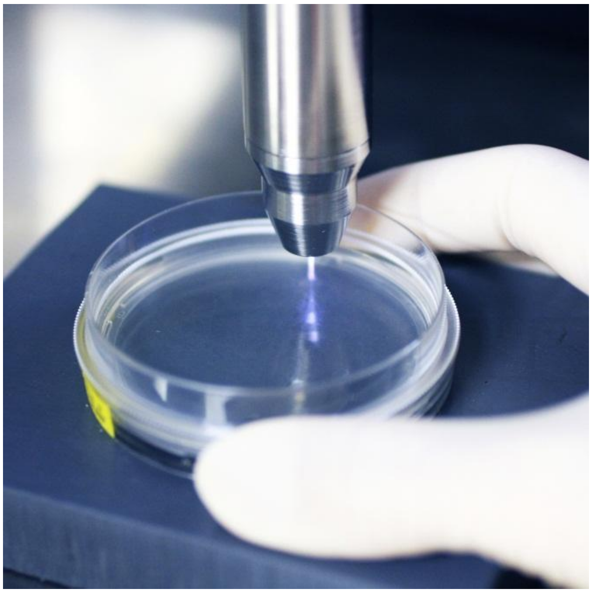

C

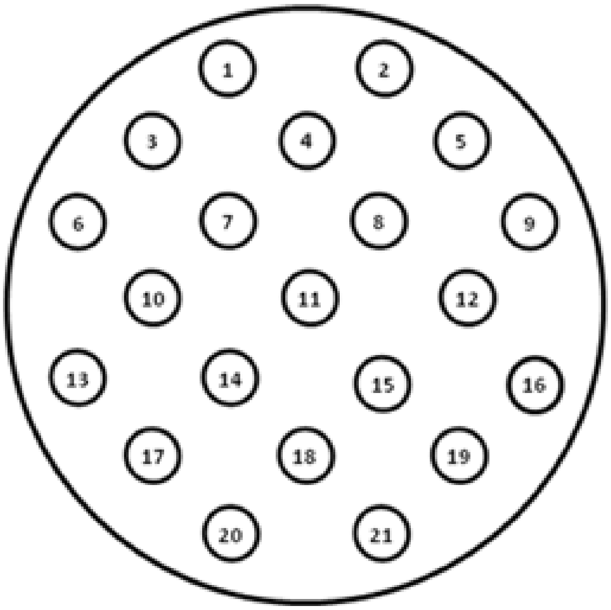
1
2
3
4
5
6
7
8
9
10
11
12
13
14
15
16
17
18
19
20
21

D

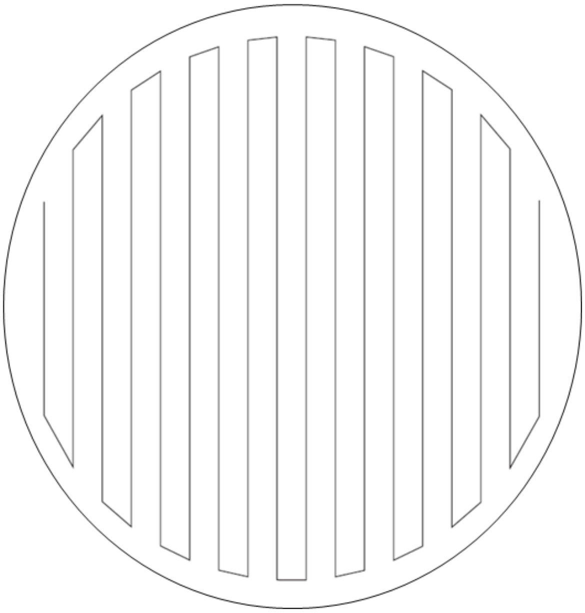

Lendeckel et al.:
Proteomic changes of tissue-tolerable plasma (TTP) treated airway epithelial cells and their relation to wound healing

Supplementary Table S-1

ID	Protein Labels	Co_24h (dose-dependent approach) ratio 'PL-30s-24h'	t-Test 'PL-30s-24h'	ratio 'PL-1min-24h'	t-Test 'PL-1min-24h'	ratio 'PL-120s-24h'	t-Test 'PL-120s-24h'	Co_48h (dose-dependent approach) ratio 'PL-30sec-48h'	t-Test 'PL-30sec-48h'	ratio 'PL-1min-48h'	t-Test 'PL-1min-48h'	ratio 'PL-2min-48h'	t-Test 'PL-2min-48h'
305		0,56	0,05	0,51	0,01	0,26	0,00	0,07	0,22	0,07	0,29	0,07	0,29
306		0,72	0,50	0,39	0,08	0,31	0,09	0,24	0,05	0,20	0,07	0,22	0,08
307		0,47	0,19	0,12	0,05	0,00	0,06	0,25	0,29	7,70	0,34	0,19	0,34
309		0,55	0,00	0,53	0,00	0,29	0,00	0,58	0,00	0,50	0,00	0,42	0,00
314		0,54	0,00	0,85	0,25	1,09	0,48	0,60	0,01	0,67	0,02	0,54	0,00
315		0,34	0,00	0,31	0,00	0,28	0,00	0,20	0,17	0,18	0,23	0,19	0,24
318	GRP78	0,45	0,00	0,33	0,00	0,19	0,00	0,04	0,32	0,05	0,41	0,05	0,41
319		0,37	0,00	0,40	0,00	0,27	0,00	0,41	0,07	68,02	0,22	0,09	0,02
325		1,12	0,00	1,26	0,01	1,50	0,00	1,06	0,11	1,09	0,10	0,93	0,02
327		0,72	0,00	0,77	0,00	0,81	0,00	0,82	0,01	0,80	0,01	0,98	0,81
331		0,75	0,00	0,83	0,02	0,81	0,03	0,80	0,05	0,79	0,04	1,21	0,31
338		0,12	0,00	0,59	0,44	0,07	0,01	0,26	0,09	1,07	0,94	0,08	0,05
339		0,61	0,00	0,55	0,00	0,44	0,00	0,67	0,03	0,51	0,00	0,60	0,01
341		0,54	0,00	0,79	0,02	0,81	0,07	0,70	0,00	0,87	0,12	0,71	0,01
348		0,68	0,00	0,71	0,00	0,67	0,00	0,84	0,00	0,70	0,00	0,78	0,02
349		0,66	0,00	0,54	0,00	0,24	0,00	0,81	0,01	0,62	0,00	0,58	0,00
350		0,73	0,00	0,64	0,00	0,40	0,00	0,80	0,00	0,65	0,00	0,57	0,00
351		0,53	0,00	0,43	0,00	0,12	0,00	0,63	0,00	0,54	0,00	0,36	0,00
352		0,46	0,00	0,33	0,00	0,05	0,00	0,58	0,00	0,50	0,00	0,33	0,00
354	SF3B2	0,55	0,00	0,47	0,00	0,16	0,00	0,81	0,00	0,66	0,00	0,59	0,00
355	SF3B2	0,50	0,00	0,43	0,00	0,21	0,00	0,77	0,00	0,58	0,00	0,53	0,00
356		0,66	0,00	0,64	0,00	0,41	0,00	0,73	0,00	0,63	0,00	0,50	0,00
361		0,63	0,00	0,19	0,00	0,00	0,00	0,75	0,01	1,09	0,72	0,49	0,01
364	HYOU1,OXRP	1,08	0,01	1,19	0,00	1,38	0,00	1,03	0,16	0,90	0,01	0,86	0,00
365	HYOU1,OXRP	0,89	0,00	0,96	0,13	1,02	0,36	0,92	0,00	0,83	0,00	0,71	0,00
371		0,83	0,01	0,72	0,00	0,48	0,00	0,93	0,21	0,90	0,22	0,77	0,00
372		0,49	0,00	0,43	0,00	0,17	0,00	0,76	0,29	0,44	0,00	0,24	0,00
376	HYOU1	1,31	0,00	1,48	0,00	1,90	0,00	1,16	0,00	1,01	0,61	1,07	0,03
377		0,45	0,00	0,25	0,00	0,00	0,00	0,53	0,07	0,64	0,18	0,29	0,02
388		0,40	0,00	0,29	0,00	0,11	0,00	0,52	0,00	0,37	0,00	0,21	0,00
393	ITA3	0,84	0,00	0,84	0,00	0,95	0,08	0,76	0,00	0,72	0,00	0,64	0,00
394		1,53	0,00	1,85	0,00	2,10	0,00	1,23	0,00	1,44	0,00	1,75	0,00
395	LPPRC	0,68	0,00	0,68	0,00	0,72	0,00	0,67	0,00	0,67	0,00	0,56	0,00
398		0,76	0,00	0,75	0,00	0,58	0,00	0,79	0,00	0,66	0,00	0,61	0,00
401		0,58	0,00	0,18	0,00	0,00	0,00	0,65	0,00	0,61	0,00	0,48	0,00
402		0,55	0,00	0,15	0,00	0,00	0,00	0,64	0,00	0,63	0,00	0,47	0,00
403		0,48	0,00	0,51	0,18	0,00	0,00	0,59	0,00	0,68	0,05	0,44	0,00
406		1,17	0,03	1,20	0,04	1,18	0,14	1,32	0,00	1,52	0,00	1,54	0,00
407		1,59	0,00	2,00	0,00	2,26	0,00	1,39	0,00	1,70	0,00	2,15	0,00
408	ITA3	0,87	0,00	0,95	0,05	1,18	0,00	0,87	0,00	0,78	0,00	0,80	0,00
409	MATR3	0,76	0,00	0,76	0,00	0,88	0,04	0,76	0,00	0,69	0,00	0,63	0,00
410	DPOD2	0,43	0,00	0,36	0,00	0,20	0,00	0,59	0,00	0,46	0,00	0,31	0,00
412		1,56	0,01	1,70	0,00	2,41	0,01	1,40	0,00	2,14	0,00	2,63	0,00
413		1,71	0,00	1,97	0,00	2,87	0,00	1,68	0,00	2,24	0,00	3,01	0,00
414		0,66	0,00	0,66	0,00	0,60	0,00	0,70	0,00	0,59	0,00	0,55	0,00
417	K1C9	1,58	0,01	1,86	0,00	2,76	0,00	1,57	0,00	2,34	0,00	3,08	0,00
418		1,83	0,00	1,90	0,00	2,67	0,00	1,78	0,00	2,23	0,00	3,03	0,00
419		2,02	0,00	2,04	0,00	2,81	0,00	1,74	0,00	2,13	0,00	2,72	0,00
420	ACTG,ITA3	0,93	0,02	0,97	0,12	1,28	0,00	0,86	0,02	0,84	0,01	0,77	0,00
423		1,34	0,00	1,58	0,00	1,82	0,00	1,21	0,00	1,34	0,00	1,64	0,00
426		0,91	0,26	0,70	0,00	0,61	0,00	1,20	0,01	0,90	0,02	1,07	0,31
427	ICAL	0,75	0,00	0,73	0,00	0,56	0,00	0,93	0,06	0,70	0,00	0,72	0,00
430	ICAL	0,72	0,00	0,65	0,00	0,59	0,00	0,93	0,05	0,72	0,00	0,75	0,00
435	DREB	0,70	0,00	0,67	0,00	0,48	0,00	0,85	0,00	0,79	0,00	0,67	0,00
436	DDB1,OXRP	0,72	0,00	0,66	0,00	0,61	0,00	0,78	0,00	0,67	0,00	0,65	0,00
438		0,69	0,00	0,62	0,00	0,48	0,00	0,71	0,00	0,59	0,00	0,63	0,00
439		0,53	0,00	0,64	0,00	0,55	0,00	0,68	0,00	0,49	0,00	0,52	0,00
440		0,61	0,00	0,41	0,00	0,25	0,00	0,62	0,00	0,59	0,00	0,49	0,00
441	HNRL2	0,58	0,00	0,54	0,00	0,35	0,00	0,63	0,00	0,52	0,00	0,48	0,00
442	DDB1	0,84	0,00	0,81	0,00	0,68	0,00	0,82	0,00	0,77	0,00	0,71	0,00
443		1,12	0,01	1,35	0,00	1,36	0,00	0,91	0,30	1,06	0,38	1,28	0,00
444		1,00	0,96	1,17	0,00	1,27	0,00	0,90	0,07	1,02	0,64	1,23	0,00
446	HNRPU,PPCE	0,40	0,00	0,61	0,00	0,36	0,00	0,72	0,00	0,45	0,00	0,38	0,00
447	HNRPU	0,83	0,00	0,70	0,00	0,42	0,00	0,80	0,00	0,68	0,00	0,62	0,00
452		0,74	0,00	0,19	0,00	0,18	0,00	0,75	0,00	0,67	0,00	0,57	0,00
453		0,67	0,00	0,33	0,00	0,13	0,00	0,69	0,00	0,67	0,00	0,58	0,00
457	HNRL2	0,57	0,00	0,52	0,00	0,31	0,00	0,59	0,00	0,45	0,00	0,45	0,00
458	HNRL2	0,61	0,00	0,53	0,00	0,34	0,00	0,65	0,00	0,54	0,00	0,50	0,00
460	MVP,SYAC,UBA1	0,64	0,00	0,60	0,00	0,39	0,00	0,73	0,00	0,61	0,00	0,53	0,00
462	ENPL	1,04	0,01	0,99	0,78	1,29	0,00	1,00	0,84	0,92	0,00	0,87	0,00
463		0,67	0,22	0,42	0,03	0,71	0,35	0,58	0,01	0,58	0,01	1,42	0,15
464	HNRPU,HNRH1	0,87	0,10	0,66	0,02	0,70	0,14	0,73	0,02	0,66	0,00	0,88	0,14
466	UBE1,MVP,UBA1	0,60	0,00	0,56	0,00	0,38	0,00	0,76	0,00	0,62	0,00	0,55	0,00
470		0,78	0,00	0,65	0,01	0,92	0,67	0,74	0,01	0,53	0,00	0,86	0,03
475	ICAL	0,66	0,00	0,70	0,00	0,41	0,00	0,89	0,14	0,70	0,00	0,64	0,00
476		0,89	0,00	0,90	0,01	0,62	0,00	0,91	0,02	0,86	0,01	0,77	0,00
477	HS105,GPIA1	0,78	0,00	0,80	0,00	0,58	0,00	0,84	0,00	0,83	0,00	0,65	0,00
478	UBA1,TIF1B	0,62	0,00	0,61	0,00	0,40	0,00	0,69	0,00	0,60	0,00	0,54	0,00
479	TIF1B,HNRPU	0,47	0,00	0,30	0,00	0,20	0,00	0,65	0,00	0,41	0,00	0,45	0,00
480		0,75	0,00	0,23	0,00	0,00	0,00	0,87	0,02	0,61	0,00	0,64	0,00
483		0,70	0,00	0,68	0,00	0,43	0,00	0,80	0,00	0,71	0,00	0,59	0,00
484		0,80	0,00	0,79	0,00	0,55	0,00	0,87	0,01	0,77	0,00	0,68	0,00
486	HS105	0,79	0,00	0,78	0,00	0,54	0,00	0,87	0,00	0,84	0,00	0,67	0,00
487	MVP	0,76	0,00	0,74	0,00	0,51	0,00	0,80	0,00	0,67	0,00	0,59	0,00
490	HSP74	0,84	0,00	0,82	0,00	0,57	0,00	0,87	0,00	0,80	0,00	0,72	0,00
491	HSP74	0,72	0,00	0,71	0,00	0,49	0,00	0,80	0,00	0,75	0,00	0,63	0,00
494	HNRL2	0,70	0,00	0,49	0,00	0,35	0,00	0,83	0,08	0,76	0,02	0,65	0,00
495		0,91	0,07	0,90	0,09	0,58	0,00	0,92	0,01	0,89	0,01	0,84	0,00
496	GANAB	0,68	0,00	0,75	0,00	0,71	0,00	0,72	0,00	0,63	0,00	0,57	0,00
499	GANAB,IMMT	0,74	0,00	0,71	0,00	0,69	0,00	0,80	0,00	0,62	0,00	0,60	0,00
500		0,58	0,00	1,04	0,65	0,60	0,01	0,65	0,04	0,59	0,02	0,45	0,01
501		0,62	0,00	0,12	0,00	0,11	0,00	0,72	0,00	0,53	0,00	0,49	0,00
507	ACTN4,HS105	0,64	0,00	0,62	0,00	0,44	0,00	0,67	0,00	0,62	0,00	0,48	0,00
508	MSH2,GANAB	0,66	0,00	0,57	0,00	0,48	0,00	0,70	0,00	0,58	0,00	0,54	0,00
510	GANAB,IMMT	0,57	0,00	0,54	0,00	0,42	0,00	0,68	0,00	0,58	0,00	0,52	0,00
516		0,66	0,00	1,11	0,04	0,78	0,00	0,76	0,08	0,77	0,06	0,59	0,01
517	ACTN4	0,80	0,00	0,76	0,00	0,58	0,00	0,76	0,00	0,69	0,00	0,60	0,00

ID	Co_72h (dose-dependent approach) ratio 'PL-30sec-72h'	t-Test 'PL-30sec-72h'	ratio 'PI-1min-72h'	t-Test 'PI-1min-72h'	ratio 'PL-2min-72h'	t-Test 'PL-2min-72h'	PI30s_24h (time-dependent approach) ratio 'PL-30sec-48h'	t-Test 'PL-30sec-48h'	ratio 'PL-30sec-72h'	t-Test 'PL-30sec-72h'
305	0,40	0,16	0,31	0,12	0,43	0,18	0,68	0,37	0,72	0,36
306	0,73	0,06	0,61	0,27	0,63	0,02	0,48	0,26	0,61	0,36
307	0,33	0,20	0,34	0,14	0,31	0,15	0,89	0,75	0,41	0,17
309	0,50	0,00	0,56	0,00	0,47	0,00	1,10	0,07	0,93	0,25
314	0,65	0,06	0,87	0,45	0,63	0,04	1,05	0,74	0,91	0,29
315	0,13	0,10	2,08	0,65	0,10	0,09	1,05	0,83	0,58	0,28
318	0,09	0,03	0,12	0,06	0,56	0,40	0,84	0,61	0,26	0,02
319	0,21	0,00	0,58	0,03	0,15	0,00	1,11	0,75	0,44	0,16
325	1,12	0,08	1,17	0,07	0,91	0,33	0,87	0,00	0,87	0,01
327	0,96	0,68	1,08	0,35	0,90	0,25	1,07	0,23	1,09	0,15
331	1,01	0,91	1,21	0,05	0,93	0,42	1,08	0,48	1,07	0,49
338	1.000,00	N,A,	N,A,	N,A,	1.000,00	N,A,	5,16	0,02	6,58	0,31
339	0,78	0,01	0,93	0,29	0,66	0,00	1,24	0,03	1,19	0,01
341	0,17	0,28	0,12	0,25	0,18	0,28	1,04	0,54	0,98	0,76
348	0,60	0,00	0,74	0,03	0,57	0,00	1,16	0,02	0,95	0,47
349	0,53	0,00	0,68	0,00	0,46	0,00	1,17	0,04	0,95	0,58
350	0,66	0,00	0,76	0,00	0,57	0,00	1,12	0,02	1,04	0,32
351	0,48	0,00	0,52	0,00	0,40	0,00	1,20	0,01	1,05	0,36
352	0,44	0,00	0,46	0,00	0,38	0,00	1,25	0,02	0,98	0,66
354	0,58	0,00	0,70	0,00	0,51	0,00	1,35	0,00	1,10	0,01
355	0,64	0,00	0,73	0,00	0,49	0,00	1,40	0,00	1,19	0,01
356	0,63	0,00	0,75	0,00	0,54	0,00	1,14	0,03	1,03	0,56
361	0,66	0,00	0,75	0,01	0,45	0,00	1,19	0,02	1,06	0,41
364	0,85	0,00	0,97	0,03	0,78	0,00	0,96	0,04	0,84	0,00
365	0,85	0,00	0,92	0,00	0,73	0,00	0,99	0,70	0,86	0,00
371	0,70	0,00	0,84	0,01	0,59	0,00	1,09	0,19	0,90	0,15
372	0,39	0,04	0,39	0,04	0,16	0,01	1,84	0,09	1,16	0,11
376	0,85	0,00	1,03	0,22	0,85	0,00	0,90	0,00	0,79	0,00
377	0,41	0,17	0,50	0,23	0,21	0,08	1,30	0,00	1,16	0,10
388	0,26	0,09	0,30	0,11	0,14	0,06	1,48	0,00	1,11	0,35
393	0,71	0,00	0,81	0,00	0,62	0,00	0,99	0,63	1,00	0,89
394	0,96	0,14	1,34	0,00	1,12	0,00	0,85	0,00	0,81	0,00
395	0,69	0,00	0,77	0,00	0,62	0,00	0,97	0,21	0,96	0,24
398	0,67	0,00	0,78	0,00	0,59	0,00	1,13	0,00	0,96	0,14
401	0,58	0,00	0,70	0,00	0,50	0,00	1,16	0,00	1,02	0,63
402	0,56	0,00	0,60	0,01	0,50	0,00	1,18	0,00	1,00	0,98
403	0,55	0,00	0,61	0,01	0,49	0,00	1,17	0,00	1,03	0,25
406	1,10	0,10	1,37	0,00	1,16	0,10	0,90	0,05	0,79	0,00
407	1,01	0,85	1,52	0,00	1,26	0,00	0,82	0,00	0,78	0,00
408	0,91	0,04	1,03	0,13	0,77	0,00	1,06	0,08	1,01	0,88
409	0,65	0,00	0,75	0,00	0,54	0,00	0,97	0,25	0,96	0,33
410	0,49	0,00	0,62	0,00	0,39	0,00	1,31	0,00	1,18	0,06
412	1,22	0,08	1,41	0,00	1,30	0,03	0,76	0,02	0,85	0,12
413	1,53	0,00	1,71	0,00	1,69	0,00	0,88	0,12	0,85	0,05
414	0,60	0,00	0,70	0,00	0,51	0,00	1,02	0,56	1,01	0,76
417	1,43	0,02	1,57	0,01	1,54	0,01	0,84	0,09	0,88	0,21
418	1,41	0,00	1,60	0,00	1,57	0,00	0,88	0,10	0,80	0,01
419	1,33	0,00	1,50	0,00	1,40	0,00	0,84	0,06	0,80	0,00
420	0,88	0,02	0,93	0,02	0,69	0,00	0,99	0,72	1,10	0,07
423	1,04	0,13	1,41	0,00	1,13	0,00	0,90	0,00	0,85	0,00
426	0,85	0,26	1,29	0,06	0,83	0,26	1,11	0,24	0,92	0,31
427	0,77	0,00	0,91	0,00	0,64	0,00	1,23	0,00	1,08	0,06
430	0,80	0,00	0,90	0,00	0,66	0,00	1,22	0,00	1,03	0,54
435	0,74	0,00	0,91	0,01	0,69	0,00	1,05	0,12	0,90	0,00
436	0,79	0,00	0,86	0,00	0,69	0,00	1,07	0,01	0,97	0,18
438	0,56	0,00	0,69	0,00	0,48	0,00	1,02	0,73	0,95	0,35
439	0,59	0,00	0,73	0,00	0,52	0,00	1,18	0,01	1,16	0,02
440	0,52	0,00	0,61	0,00	0,50	0,00	1,07	0,12	0,98	0,63
441	0,60	0,00	0,71	0,00	0,48	0,00	1,13	0,00	1,02	0,58
442	0,75	0,00	0,88	0,00	0,70	0,00	0,98	0,44	0,88	0,00
443	0,74	0,00	0,92	0,01	0,81	0,00	0,92	0,17	0,98	0,59
444	0,79	0,00	0,95	0,12	0,89	0,04	0,92	0,22	0,94	0,14
446	0,59	0,00	0,76	0,00	0,38	0,00	1,80	0,01	1,66	0,02
447	0,57	0,00	0,74	0,00	0,50	0,00	0,97	0,50	0,85	0,02
452	0,66	0,00	0,78	0,00	0,59	0,00	1,02	0,50	0,93	0,02
453	0,64	0,00	0,76	0,00	0,61	0,00	0,98	0,72	0,90	0,08
457	0,55	0,00	0,66	0,00	0,45	0,00	1,23	0,00	1,19	0,00
458	0,60	0,00	0,73	0,00	0,50	0,00	1,14	0,00	1,05	0,07
460	0,70	0,00	0,78	0,00	0,59	0,00	1,24	0,00	1,17	0,00
462	1,15	0,00	0,84	0,00	0,98	0,38	1,14	0,00	1,06	0,01
463	0,57	0,00	0,73	0,01	0,91	0,48	0,68	0,19	0,94	0,80
464	0,67	0,00	0,79	0,01	0,75	0,00	0,90	0,36	0,94	0,41
466	0,70	0,00	0,82	0,00	0,64	0,00	1,20	0,00	1,01	0,58
470	0,60	0,00	0,71	0,00	0,61	0,00	0,99	0,93	1,03	0,78
475	0,72	0,00	0,88	0,00	0,63	0,00	1,24	0,01	1,12	0,07
476	0,76	0,00	0,97	0,08	0,85	0,00	0,90	0,00	0,74	0,00
477	0,74	0,00	0,89	0,00	0,77	0,00	0,92	0,00	0,75	0,00
478	0,65	0,00	0,75	0,00	0,56	0,00	1,25	0,00	1,18	0,00
479	0,73	0,00	0,73	0,00	0,62	0,00	1,37	0,01	1,23	0,02
480	0,67	0,00	0,73	0,01	0,56	0,00	1,46	0,00	1,21	0,03
483	0,59	0,00	0,81	0,00	0,62	0,00	1,07	0,01	0,82	0,00
484	0,66	0,00	0,91	0,02	0,68	0,00	0,94	0,02	0,79	0,00
486	0,80	0,00	0,94	0,01	0,83	0,00	0,93	0,01	0,76	0,00
487	0,65	0,00	0,81	0,00	0,63	0,00	1,15	0,00	1,01	0,75
490	0,73	0,00	0,92	0,00	0,76	0,00	1,00	0,85	0,87	0,00
491	0,81	0,00	0,86	0,00	0,81	0,00	1,03	0,14	0,91	0,00
494	0,87	0,02	0,99	0,82	0,80	0,00	1,02	0,70	0,88	0,00
495	0,70	0,00	0,94	0,00	0,79	0,00	0,89	0,01	0,78	0,00
496	0,62	0,00	0,75	0,00	0,48	0,00	1,11	0,00	1,11	0,00
499	0,63	0,00	0,78	0,00	0,49	0,00	1,18	0,00	1,12	0,01
500	0,38	0,00	0,80	0,00	0,42	0,00	1,20	0,17	0,93	0,48
501	0,74	0,00	0,79	0,00	0,52	0,00	1,33	0,00	1,24	0,00
507	0,73	0,00	0,79	0,00	0,68	0,00	1,06	0,01	0,92	0,00
508	0,68	0,00	0,75	0,00	0,52	0,00	1,11	0,01	1,05	0,07
510	0,55	0,00	0,72	0,00	0,45	0,00	1,19	0,01	1,19	0,00
516	0,46	0,00	0,82	0,00	0,45	0,00	1,05	0,55	0,88	0,04
517	0,63	0,00	0,81	0,00	0,65	0,00	0,96	0,00	0,83	0,00

ID	PI60s_24h (time-dependent approach) ratio 'PL-1min-48h'	t-Test 'PL-1min-48h'	ratio 'PI-1min-72h'	t-Test 'PI-1min-72h'	PI120s_24h (time-dependent approach) ratio 'PL-2min-48h'	t-Test 'PL-2min-48h'	ratio 'PL-2min-72h'	t-Test 'PL-2min-72h'
305	0,74	0,24	0,60	0,14	1,32	0,01	1,65	0,00
306	0,71	0,36	0,92	0,86	1,00	0,99	1,20	0,40
307	107,88	0,28	1,64	0,50	1.000,00	0,07	1.000,00	0,21
309	0,98	0,80	1,08	0,37	1,51	0,00	1,68	0,00
314	0,73	0,13	0,77	0,19	0,46	0,00	0,44	0,00
315	1,02	0,96	10,41	0,19	1,25	0,04	0,57	0,40
318	1,38	0,29	0,46	0,16	2,28	0,00	3,66	0,30
319	168,83	0,21	1,15	0,59	0,34	0,18	0,43	0,06
325	0,80	0,02	0,80	0,03	0,57	0,00	0,53	0,00
327	0,96	0,45	1,13	0,02	1,12	0,21	0,89	0,12
331	0,96	0,71	1,16	0,02	1,51	0,13	0,90	0,08
338	4,18	0,33	0,00	N,A,	2,77	0,31	2,82	0,46
339	1,05	0,57	1,59	0,00	1,53	0,02	1,40	0,02
341	0,90	0,35	0,46	0,03	0,72	0,07	0,69	0,02
348	0,92	0,14	1,12	0,14	1,09	0,39	0,92	0,19
349	1,09	0,33	1,45	0,00	2,36	0,01	2,26	0,01
350	1,03	0,36	1,36	0,00	1,44	0,00	1,62	0,00
351	1,26	0,03	1,39	0,04	2,91	0,01	3,78	0,00
352	1,51	0,07	1,41	0,17	6,68	0,00	8,03	0,00
354	1,29	0,00	1,57	0,00	3,33	0,00	3,27	0,00
355	1,23	0,00	1,59	0,00	2,30	0,00	2,16	0,00
356	1,02	0,52	1,27	0,00	1,28	0,00	1,44	0,00
361	5,79	0,01	4,08	0,01	1.000,00	0,02	1.000,00	0,00
364	0,77	0,00	0,87	0,00	0,63	0,00	0,60	0,00
365	0,83	0,00	0,87	0,00	0,68	0,00	0,65	0,00
371	1,22	0,07	1,26	0,01	1,54	0,00	1,32	0,12
372	1,22	0,20	1,32	0,19	1,68	0,10	1,43	0,31
376	0,70	0,00	0,84	0,00	0,58	0,00	0,54	0,00
377	2,78	0,04	2,46	0,03	1.000,00	0,01	1.000,00	0,00
388	1,44	0,02	1,82	0,02	2,19	0,08	2,24	0,08
393	0,94	0,05	1,13	0,00	0,73	0,00	0,76	0,00
394	0,82	0,00	0,94	0,01	0,88	0,00	0,69	0,00
395	0,97	0,44	1,08	0,02	0,77	0,00	0,82	0,01
398	0,95	0,38	1,13	0,03	1,14	0,02	1,12	0,01
401	3,50	0,01	3,98	0,00	1.000,00	0,00	1.000,00	0,00
402	4,25	0,01	3,88	0,02	1.000,00	0,00	1.000,00	0,00
403	1,25	0,72	1,06	0,93	1.000,00	0,00	1.000,00	0,00
406	1,01	0,93	0,96	0,50	1,04	0,68	0,83	0,13
407	0,79	0,00	0,93	0,02	0,89	0,02	0,68	0,00
408	0,87	0,00	1,04	0,05	0,71	0,00	0,63	0,00
409	0,89	0,01	1,11	0,01	0,71	0,00	0,69	0,00
410	1,20	0,01	1,75	0,00	1,44	0,05	1,97	0,00
412	1,07	0,50	0,90	0,22	0,92	0,67	0,58	0,03
413	1,02	0,75	0,83	0,01	0,94	0,54	0,56	0,00
414	0,84	0,01	1,17	0,02	0,88	0,12	0,95	0,45
417	1,07	0,48	0,83	0,01	0,95	0,74	0,54	0,01
418	1,07	0,47	0,88	0,14	1,03	0,81	0,62	0,01
419	1,02	0,84	0,89	0,17	0,94	0,50	0,60	0,00
420	0,93	0,01	1,11	0,00	0,65	0,00	0,63	0,00
423	0,85	0,00	0,98	0,18	0,90	0,00	0,68	0,00
426	1,08	0,07	1,81	0,00	1,48	0,01	1,35	0,04
427	0,96	0,43	1,32	0,00	1,28	0,00	1,22	0,00
430	1,04	0,45	1,27	0,00	1,20	0,00	1,03	0,17
435	1,01	0,84	1,16	0,02	1,20	0,00	1,21	0,00
436	1,00	0,97	1,14	0,00	1,05	0,03	1,00	0,90
438	0,94	0,51	1,30	0,01	1,31	0,01	1,17	0,15
439	0,70	0,00	1,17	0,01	0,88	0,24	0,98	0,85
440	1,49	0,12	1,69	0,04	2,04	0,07	2,26	0,03
441	1,01	0,82	1,30	0,00	1,45	0,00	1,36	0,00
442	0,96	0,23	1,08	0,02	1,05	0,08	1,02	0,33
443	0,88	0,03	1,01	0,79	1,06	0,15	0,89	0,08
444	0,89	0,04	0,97	0,38	1,00	0,91	0,84	0,02
446	0,73	0,02	1,40	0,00	1,05	0,78	1,21	0,20
447	0,98	0,66	1,32	0,00	1,49	0,00	1,46	0,00
452	3,52	0,03	4,23	0,01	3,18	0,03	3,46	0,02
453	1,94	0,08	2,15	0,04	4,24	0,01	4,40	0,00
457	1,02	0,64	1,54	0,00	1,73	0,00	1,77	0,00
458	1,08	0,10	1,48	0,00	1,59	0,00	1,58	0,00
460	1,08	0,02	1,38	0,00	1,43	0,00	1,60	0,00
462	1,11	0,10	0,82	0,01	0,80	0,00	0,73	0,00
463	1,07	0,72	1,90	0,00	1,58	0,19	1,42	0,22
464	1,07	0,67	1,44	0,04	1,35	0,21	1,29	0,30
466	1,06	0,11	1,29	0,00	1,38	0,00	1,47	0,00
470	0,85	0,29	1,47	0,01	0,98	0,90	0,89	0,58
475	0,92	0,14	1,29	0,00	1,42	0,00	1,57	0,00
476	0,83	0,00	0,93	0,03	1,09	0,01	1,18	0,00
477	0,89	0,01	0,88	0,00	0,96	0,05	1,06	0,02
478	1,09	0,02	1,36	0,00	1,50	0,00	1,56	0,00
479	1,35	0,01	1,91	0,00	2,27	0,00	2,49	0,00
480	3,25	0,02	4,14	0,00	1.000,00	0,00	1.000,00	0,00
483	0,96	0,24	1,15	0,00	1,27	0,00	1,40	0,00
484	0,85	0,00	1,09	0,05	1,07	0,02	1,18	0,00
486	0,92	0,02	0,91	0,01	1,06	0,01	1,16	0,00
487	1,00	0,98	1,28	0,00	1,26	0,00	1,44	0,00
490	0,93	0,10	1,12	0,01	1,22	0,00	1,36	0,00
491	0,98	0,66	0,98	0,55	1,20	0,00	1,32	0,00
494	1,34	0,00	1,43	0,00	1,64	0,00	1,65	0,00
495	0,88	0,05	1,08	0,14	1,28	0,00	1,40	0,00
496	0,88	0,04	1,21	0,01	0,85	0,01	0,82	0,00
499	0,96	0,51	1,47	0,00	0,95	0,20	0,94	0,13
500	0,61	0,00	1,09	0,03	0,80	0,02	1,00	0,97
501	4,87	0,01	6,67	0,00	4,83	0,01	4,68	0,01
507	1,01	0,76	1,04	0,35	1,12	0,01	1,25	0,00
508	1,09	0,01	1,36	0,00	1,18	0,00	1,11	0,01
510	1,08	0,32	1,69	0,00	1,25	0,12	1,35	0,04
516	0,63	0,00	0,92	0,10	0,69	0,00	0,72	0,00
517	0,91	0,05	1,11	0,02	1,05	0,10	1,18	0,00

518	ACTN4,ERO1A	0,69	0,00	0,64	0,00	0,47	0,00	0,71	0,00	0,66	0,00	0,53	0,00	0,69	0,00	0,80	0,00	0,69	0,00	1,00	0,63	0,85	0,00	1,01	0,72	1,06	0,07	1,11	0,01	1,25	0,00
519		0,56	0,00	0,57	0,00	0,41	0,00	0,72	0,00	0,57	0,00	0,56	0,00	0,52	0,00	0,72	0,00	0,48	0,00	1,12	0,10	0,95	0,49	0,86	0,04	1,28	0,00	1,17	0,06	1,19	0,03
520	NUCL	0,76	0,00	0,63	0,00	0,50	0,00	0,75	0,00	0,60	0,00	0,62	0,00	0,63	0,00	0,76	0,00	0,52	0,00	1,02	0,48	0,94	0,07	0,98	0,56	1,36	0,00	1,27	0,00	1,19	0,00
522		0,22	0,00	0,28	0,00	0,03	0,00	0,90	0,91	0,22	0,16	0,28	0,21	0,00	0,00	0,64	0,00	0,14	0,00	7,52	0,30	0,00	0,00	1,48	0,37	2,01	0,06	16,09	0,28	3,77	0,38
523		0,71	0,00	0,91	0,03	0,67	0,00	0,78	0,01	0,78	0,01	0,63	0,00	0,55	0,00	0,78	0,00	0,53	0,00	0,98	0,48	0,85	0,00	0,76	0,00	0,93	0,05	0,83	0,01	0,85	0,01
524		0,47	0,00	0,52	0,00	0,36	0,00	0,63	0,01	0,49	0,00	0,39	0,00	0,38	0,00	0,69	0,00	0,40	0,00	1,23	0,14	0,84	0,11	0,85	0,14	1,36	0,00	0,99	0,90	1,16	0,29
525		0,67	0,00	0,56	0,00	0,37	0,00	0,70	0,00	0,62	0,00	0,57	0,00	0,48	0,00	0,69	0,00	0,47	0,00	1,01	0,94	0,80	0,03	1,07	0,56	1,38	0,01	1,48	0,00	1,43	0,01
527		0,78	0,01	0,99	0,89	0,68	0,00	0,74	0,13	0,70	0,07	0,58	0,02	0,42	0,00	0,77	0,00	0,47	0,00	1,06	0,58	0,94	0,40	0,79	0,01	1,36	0,00	0,95	0,37	1,20	0,01
528		0,39	0,00	0,50	0,00	0,34	0,00	0,64	0,00	0,48	0,00	0,40	0,00	0,59	0,00	0,73	0,00	0,50	0,00	1,53	0,00	1,17	0,21	0,90	0,25	1,12	0,02	1,08	0,30	1,12	0,19
535	GLU2B	1,02	0,27	1,20	0,00	1,36	0,00	1,07	0,01	0,86	0,00	0,84	0,00	0,84	0,00	1,02	0,23	0,72	0,00	1,11	0,00	0,92	0,00	0,76	0,00	0,95	0,07	0,65	0,00	0,59	0,00
538		0,86	0,01	0,51	0,01	0,31	0,00	0,82	0,00	0,69	0,00	0,59	0,00	0,71	0,00	0,80	0,00	0,60	0,00	1,10	0,00	1,05	0,04	1,54	0,07	2,00	0,01	2,19	0,00	2,47	0,00
542	TERA	0,88	0,00	0,90	0,01	0,63	0,00	0,91	0,00	0,74	0,00	0,76	0,00	0,76	0,00	0,96	0,01	0,74	0,00	1,04	0,01	0,93	0,01	0,84	0,00	1,16	0,00	1,23	0,00	1,26	0,00
545	UBP5	0,82	0,00	0,76	0,00	0,56	0,00	0,86	0,00	0,67	0,00	0,66	0,00	0,76	0,00	0,90	0,00	0,72	0,00	1,06	0,04	0,88	0,00	0,89	0,03	1,13	0,01	1,18	0,01	1,22	0,00
550	UBP5,P3H1	0,67	0,00	0,67	0,00	0,48	0,00	0,78	0,00	0,67	0,00	0,59	0,00	0,70	0,00	0,79	0,00	0,62	0,00	1,09	0,00	0,94	0,02	0,95	0,30	1,09	0,04	1,17	0,00	1,18	0,00
553	HS90A	0,95	0,48	0,79	0,00	0,87	0,03	0,98	0,56	1,09	0,09	0,97	0,45	1,18	0,02	0,89	0,09	1,14	0,02	1,18	0,03	1,16	0,06	1,59	0,00	1,06	0,37	1,29	0,00	1,23	0,00
557	SRC8	0,70	0,00	0,72	0,00	0,49	0,00	0,79	0,00	0,69	0,00	0,58	0,00	0,65	0,00	0,89	0,00	0,63	0,00	1,16	0,00	0,99	0,77	0,98	0,60	1,32	0,00	1,22	0,00	1,35	0,00
558	P3H3	0,83	0,00	0,39	0,02	0,13	0,00	0,92	0,07	0,88	0,21	0,62	0,00	0,77	0,00	0,85	0,01	0,62	0,00	1,23	0,00	1,13	0,06	2,56	0,03	2,69	0,01	5,28	0,00	5,71	0,00
559	HS90A,DP13A,SRC8	0,67	0,00	0,85	0,07	0,59	0,00	0,76	0,00	0,67	0,00	0,54	0,00	0,87	0,03	0,92	0,10	0,75	0,00	1,13	0,01	0,94	0,19	0,78	0,04	0,79	0,04	0,91	0,08	0,92	0,31
564	P3H2,KU86,HS90A	0,83	0,00	0,91	0,05	0,77	0,00	0,89	0,11	0,79	0,00	0,75	0,00	0,75	0,00	0,97	0,45	0,75	0,00	1,04	0,40	0,86	0,00	0,83	0,00	1,01	0,74	0,94	0,11	0,93	0,09
565	CSDE1	0,57	0,00	0,18	0,00	0,03	0,00	0,74	0,01	0,69	0,00	0,49	0,00	0,84	0,04	0,85	0,00	0,55	0,00	0,99	0,75	0,89	0,14	2,94	0,01	2,87	0,02	13,70	0,00	12,04	0,00
566	PSMD2	0,91	0,01	0,92	0,03	0,68	0,00	0,87	0,00	0,88	0,01	0,72	0,00	0,71	0,00	0,93	0,04	0,83	0,00	0,84	0,00	0,69	0,00	0,84	0,01	0,90	0,02	0,93	0,07	1,08	0,08
567		0,97	0,69	1,18	0,00	0,89	0,01	1,03	0,70	1,00	1,00	0,82	0,03	0,68	0,00	1,05	0,13	0,86	0,00	0,94	0,40	0,71	0,01	0,75	0,00	0,92	0,02	0,81	0,00	0,99	0,57
571	HS90A	0,76	0,01	0,92	0,36	0,60	0,00	0,79	0,03	0,80	0,03	0,58	0,00	0,88	0,17	0,95	0,37	0,75	0,01	1,02	0,82	0,76	0,03	0,85	0,10	0,69	0,01	0,94	0,27	0,83	0,07
573	HS90A	0,74	0,00	0,88	0,20	0,62	0,00	0,86	0,00	0,90	0,01	0,65	0,00	0,92	0,05	0,97	0,34	0,94	0,07	0,89	0,02	0,74	0,00	0,78	0,04	0,66	0,01	0,81	0,00	0,91	0,02
574	HS90A,HS90B	0,88	0,02	0,96	0,46	0,68	0,00	0,90	0,07	0,90	0,14	0,66	0,00	0,90	0,11	0,95	0,48	0,92	0,18	0,93	0,12	0,78	0,00	0,85	0,05	0,75	0,01	0,89	0,04	1,02	0,54
575	HS90A	0,82	0,00	0,93	0,42	0,65	0,00	0,86	0,00	0,82	0,00	0,61	0,00	0,83	0,00	0,97	0,32	0,85	0,01	0,96	0,06	0,77	0,00	0,81	0,05	0,78	0,03	0,87	0,02	0,99	0,80
576		0,76	0,00	1,02	0,84	0,65	0,00	0,83	0,01	0,85	0,01	0,64	0,00	0,69	0,01	0,93	0,35	0,81	0,02	0,96	0,43	0,69	0,00	0,73	0,02	0,70	0,02	0,87	0,04	0,95	0,21
577	IMMT,PLOD3,HS90B	0,90	0,01	0,85	0,01	0,61	0,00	0,88	0,00	0,72	0,00	0,69	0,00	0,88	0,00	0,93	0,01	0,69	0,00	1,04	0,13	1,01	0,52	0,90	0,08	1,14	0,02	1,20	0,01	1,18	0,02
578		1,33	0,01	1,08	0,54	1,08	0,54	0,94	0,59	0,81	0,08	0,85	0,20	0,88	0,02	0,93	0,10	0,72	0,00	0,89	0,21	0,83	0,01	0,95	0,62	1,08	0,47	1,00	1,00	0,84	0,16
581		1,62	0,07	5,20	0,00	3,56	0,01	5,39	0,00	2,22	0,01	3,76	0,00	0,18	0,00	0,89	0,62	0,20	0,00	1,27	0,06	1,05	0,63	0,16	0,00	1,67	0,06	0,40	0,01	0,53	0,04
582		2,43	0,00	3,98	0,00	3,66	0,00	3,09	0,00	2,38	0,01	2,95	0,00	0,37	0,00	1,28	0,10	0,50	0,01	0,71	0,03	0,65	0,01	0,33	0,00	1,38	0,04	0,45	0,00	0,58	0,00
584	GELS,MX1,HS90A	0,63	0,00	0,66	0,00	0,40	0,00	0,76	0,00	0,61	0,00	0,69	0,00	0,67	0,00	0,86	0,00	0,60	0,00	1,29	0,00	1,23	0,00	0,99	0,94	1,51	0,00	1,83	0,00	1,73	0,00
585	MD1L1,GELS	0,47	0,00	0,51	0,00	0,38	0,00	0,70	0,00	0,56	0,00	0,58	0,00	0,75	0,00	0,85	0,00	0,62	0,00	1,48	0,00	1,48	0,00	1,08	0,13	1,56	0,00	1,52	0,00	1,52	0,00
586		0,81	0,53	1,55	0,24	0,83	0,62	1,91	0,02	1,58	0,13	1,57	0,08	0,13	0,00	0,71	0,14	0,15	0,00	0,62	0,10	0,46	0,03	0,27	0,02	1,33	0,34	0,50	0,04	0,53	0,07
587		1,58	0,00	2,32	0,00	2,26	0,00	2,31	0,00	1,25	0,35	1,89	0,01	0,46	0,01	1,10	0,58	0,59	0,02	0,84	0,02	0,86	0,10	0,31	0,00	1,39	0,06	0,48	0,00	0,76	0,05
589		0,69	0,00	0,69	0,02	0,38	0,00	0,79	0,02	0,67	0,00	0,64	0,00	0,60	0,00	0,89	0,05	0,60	0,00	1,08	0,25	0,89	0,00	0,92	0,55	1,32	0,04	1,57	0,00	1,58	0,01
590		0,60	0,00	0,27	0,00	0,09	0,00	0,53	0,00	0,46	0,00	0,46	0,00	0,62	0,00	0,74	0,00	0,47	0,00	0,99	0,78	1,11	0,06	1,90	0,15	2,93	0,01	5,85	0,00	5,74	0,00
593		0,79	0,00	0,81	0,04	0,71	0,01	0,87	0,04	0,77	0,00	0,86	0,01	0,74	0,00	0,95	0,19	0,75	0,00	1,06	0,20	0,97	0,21	0,91	0,29	1,22	0,03	1,18	0,07	1,12	0,20
594	GRP78	1,08	0,49	1,27	0,01	1,32	0,02	1,17	0,11	0,93	0,40	1,20	0,14	0,86	0,04	0,94	0,14	0,79	0,01	1,03	0,81	1,03	0,80	0,70	0,00	0,96	0,34	0,86	0,15	0,77	0,01
595		0,42	0,01	0,64	0,10	0,65	0,20	0,75	0,13	2,63	0,13	6,25	0,21	4,10	0,29	0,89	0,06	2,06	0,00	2,07	0,01	5,51	0,26	4,67	0,07	0,77	0,31	11,06	0,25	1,78	0,08
603	HS90A	0,92	0,04	1,08	0,08	1,11	0,15	0,96	0,51	0,96	0,52	1,05	0,45	0,80	0,00	0,97	0,33	0,84	0,00	1,03	0,54	0,94	0,15	0,87	0,01	0,98	0,56	0,93	0,32	0,82	0,03
604		0,74	0,17	0,17	0,01	6,07	0,36	0,42	0,07	0,29	0,03	0,32	0,04	0,64	0,00	0,74	0,00	0,49	0,00	0,66	0,07	0,72	0,06	2,00	0,37	3,62	0,04	0,06	0,31	0,07	0,32
607	THOP1	0,95	0,19	0,92	0,18	0,72	0,00	0,82	0,00	0,73	0,00	0,70	0,00	0,77	0,00	0,90	0,00	0,61	0,00	0,97	0,30	0,97	0,23	0,90	0,12	1,18	0,02	1,10	0,15	1,02	0,74
608		1,06	0,65	0,95	0,78	0,76	0,15	0,77	0,05	0,67	0,01	0,78	0,08	0,75	0,00	0,93	0,26	0,61	0,00	0,85	0,12	0,87	0,14	0,82	0,31	1,21	0,27	1,19	0,27	0,98	0,89
613		0,35	0,00	0,44	0,01	0,39	0,04	0,38	0,00	0,89	0,08	0,11	0,00	0,00	0,00	0,71	0,00	1,23	0,83	0,97	0,88	0,00	0,02	1,81	0,02	1,25	0,36	0,25	0,21	2,42	0,59
617	IF4B,NEBU	0,85	0,00	0,87	0,01	0,68	0,00	0,88	0,00	0,82	0,00	0,70	0,00	0,74	0,00	0,95	0,03	0,84	0,00	0,92	0,00	0,72	0,00	0,82	0,01	0,89	0,02	0,91	0,03	1,00	0,87
618	KU86,HS90A	0,73	0,00	0,66	0,00	0,53	0,00	0,75	0,00	0,63	0,00	0,62	0,00	0,68	0,00	0,76	0,00	0,57	0,00	1,08	0,02	0,98	0,29	1,02	0,60	1,23	0,00	1,23	0,00	1,13	0,00
622	GSPT1,HS90A,ERF3B	0,76	0,00	0,78	0,00	0,51	0,00	0,82	0,00	0,80	0,00	0,64	0,00	0,74	0,00	0,89	0,00	0,79	0,00	0,95	0,00	0,77	0,00	0,89	0,01	0,90	0,01	1,10	0,01	1,23	0,00
623	HS90A	0,76	0,00	0,78	0,00	0,63	0,00	0,80	0,00	0,71	0,00	0,71	0,00	0,60	0,00	0,80	0,00	0,60	0,00	0,98	0,59	0,87	0,01	0,86	0,00	1,14	0,00	1,07	0,24	1,05	0,36
624	DPYL2,ZYX,EZRI,CAL D1,IMMT	0,89	0,01	0,27	0,02	0,00	0,00	0,84	0,00	0,76	0,00	0,70	0,00	0,72	0,00	0,87	0,00	0,69	0,00	0,94	0,01	0,88	0,00	2,83	0,08	3,50	0,03	1.000,00	0,00	1.000,00	0,00
627	HS90A,MRE11,FETA	1,14	0,00	1,40	0,00	1,56	0,00	1,21	0,00	1,43	0,00	1,87	0,00	1,07	0,02	1,15	0,02	1,33	0,00	0,87	0,00	0,91	0,00	0,84	0,02	0,80	0,01	0,99	0,71	0,83	0,01
628	HS90A	1,58	0,00	1,95	0,00	2,46	0,00	1,48	0,00	1,84	0,00	2,60	0,00	1,32	0,00	1,46	0,00	1,65	0,00	0,84	0,00	0,92	0,00	0,84	0,00	0,82	0,00	0,94	0,12	0,74	0,00
629	KU86,HS90A	0,70	0,00	0,69	0,00	0,58	0,00	0,72	0,00	0,65	0,00	0,63	0,00	0,75	0,00	0,80	0,00	0,63	0,00	1,10	0,00	1,04	0,05	1,02	0,67	1,14	0,01	1,16	0,07	1,05	0,50
631	IMMT,PLOD3,THOP1	0,95	0,06	0,91	0,00	0,73	0,00	0,90	0,00	0,78	0,00	0,72	0,00	0,84	0,00	0,93	0,00	0,70	0,00	1,00	0,95	0,94	0,02	0,91	0,01	1,09	0,00	1,04	0,15	1,01	0,56
636		0,92	0,03	0,86	0,01	0,76	0,00	0,82	0,00	0,79	0,00	0,67	0,00	0,63	0,00	0,79	0,00	0,72	0,00	0,75	0,00	0,62	0,00	0,77	0,00	0,84	0,00	0,74	0,00	0,86	0,00
637	EZRI	0,71	0,00	0,89	0,28	1,04	0,72	0,80	0,00	0,83	0,00	0,73	0,00	0,87	0,00	0,87	0,00	0,87	0,00	1,01	0,76	0,95	0,10	0,84	0,15	0,76	0,05	0,63	0,01	0,65	0,01
638	MSH2	0,82	0,00	0,94	0,38	0,87	0,04	0,90	0,00	0,95	0,00	1,03	0,29	0,80	0,00	0,90	0,00	0,82	0,00	1,03	0,42	0,99	0,73	0,93	0,35	0,97	0,66	1,11	0,14	0,97	0,63
639		0,87	0,00	0,92	0,83	1,04	0,95	0,75	0,00	0,79	0,00	0,63	0,00	0,68	0,00	0,82	0,00	0,83	0,00	0,72	0,00	0,63	0,00	0,71	0,51	0,72	0,52	0,50	0,42	0,64	0,56
642		0,37	0,00	0,71	0,01	0,40	0,00	0,41	0,00	0,85	0,16	0,34	0,00	0,02	0,00	0,55	0,00	0,89	0,88	1,02	0,77	0,04	0,00	1,12	0,31	0,68	0,02	0,78	0,40	1,96	0,62
643	FKB10,DC1I2	0,95	0,07	1,08	0,01	0,89	0,01	1,01	0,66	0,96	0,28	0,86	0,00	0,79	0,00	1,01	0,57	0,81	0,00	0,98	0,38	0,81	0,00	0,82	0,00	0,90	0,00	0,90	0,01	0,88	0,00
644	DPYL2,ZYX,EZRI,CAL D1,IMMT	0,92	0,01	1,40	0,51	2,13	0,34	0,84	0,00	0,91	0,00	0,80	0,00	0,64	0,00	0,89	0,00	0,86	0,00	0,75	0,00	0,64	0,00	0,54	0,30	0,59	0,35	0,31	0,23	0,37	0,27
650	DC1I2	0,57	0,00	0,56	0,00	0,48	0,00	0,69	0,00	0,56	0,00	0,52	0,00	0,65	0,00	0,74	0,00	0,55	0,00	1,10	0,00	1,04	0,14	0,93	0,10	1,23	0,00	1,02	0,67	1,07	0,06
651	ACPH	0,75	0,00	0,79	0,00	0,61	0,00	0,84	0,00	0,71	0,00	0,64	0,00	0,73	0,00	0,85	0,00	0,60	0,00	1,16	0,00	1,03	0,04	0,92	0,04	1,14	0,00	1,10	0,00	1,05	0,02
652		1,09	0,05	1,27	0,00	1,05	0,03	0,86	0,00	1,03	0,47	0,88	0,01	0,67	0,00	0,92	0,02	1,03	0,46	0,65	0,00	0,54	0,00	0,66	0,00	0,64	0,00	0,68	0,00	0,86	0,01
658	GRP78	1,04	0,48	1,08	0,45	1,41	0,00	1,10	0,00	1,00	0,81	0,94	0,00	1,11	0,00	0,83	0,00	1,01	0,52	1,05	0,32	0,95	0,30	0,92	0,38	0,69	0,01	0,66	0,00	0,64	0,00
659	NDUS1	0,88	0,01	0,88	0,03	0,59	0,00	0,77	0,00	0,60	0,00	0,57	0,00	0,70	0,00	0,83	0,00	0,48	0,00	0,98	0,48	0,93	0,09	0,76	0,00	1,10	0,06	1,08	0,16	0,94	0,29
660	PDIA4	1,33	0,00	1,46	0,00	1,68	0,00	1,52	0,00	2,06	0,00	2,87	0,00	1,02	0,77	1,52	0,00	1,44	0,00	0,81	0,00	0,95	0,24	0,99	0,90	1,29	0,00	1,20	0,04	1,07	0,20
661	GRP78	1,69	0,00	2,06	0,00	2,89	0,00	1,55	0,00	1,39	0,00	1,74	0,00	1,19	0,00	1,79	0,00	1,64	0,00	0,82	0,00	0,68	0,00	0,60	0,00	0,84	0,00	0,54	0,00	0,55	0,00
662	GRP78	1,32	0,00	1,42	0,00	1,91	0,00	1,26	0,00	1,08	0,02	1,23	0,00	1,10	0,01	1,26	0,00	1,26	0,00	0,91	0,00	0,77	0,00	0,72	0,00	0,82	0,02	0,61	0,00	0,60	0,00
663	MEPD,THOP1	0,64	0,00	0,63	0,00	0,52	0,00	0,68	0,00	0,67	0,00	0,56	0,00	0,71	0,00	0,79	0,00	0,67	0,00	1,11	0,07	1,01	0,69	1,11	0,03	1,13	0,02	1,14	0,09	1,18	0,06
667	GRP75,PDIA4,HSP71	0,89	0,01	0,97	0,48	0,85	0,01	0,86	0,00	0,82	0,00	0,79	0,00	0,94	0,01	0,94	0,03	0,93	0,01	0,96	0,07	0,96	0,05	0,84	0,00	0,87	0,02	0,93	0,04	0,99	0,75
668	CPNE1,GRP75,PDIA4	0,97	0,12	1,13	0,02	1,30	0,00	1,11	0,01	1,15	0,00	1,23	0,00	0,87	0,00	1,06	0,07	0,89	0,03	0,98	0,26	1,04	0,21	0,86	0,01	1,09	0,06	0,80	0,00	0,79	0,00
672	GRP78	1,13	0,03	1,10	0,11	1,27	0,00	1,01	0,24	0,85	0,00	0,83	0,00	0,93	0,03	0,88	0,01	0,77	0,00	0,96	0,17	0,90	0,01	0,83	0,01	0,88	0,03	0,70	0,00	0,67	0,00
674		0,77	0,00	1,18	0,55	2,00	0,18	0,80	0,00	0,87	0,00	0,79	0,00	0,71	0,00	0,92	0,04	0,77	0,00	0,91	0,01	0,93	0,03	0,64	0,18	0,79	0,40	0,35	0,10	0,39	0,12
683	SYK,TRFL	1,18	0,00	1,26	0,64	2,39	0,18	1,25	0,00	1,65	0,00	2,16	0,00	1,28	0,00	1,47	0,00	1,65	0,00	0,89	0,00	0,98	0,40	1,10	0,82	1,06	0,90	0,76	0,55	0,63	0,36
688	TRFL	1,54	0,00	1,60	0,01	1,75	0,01	1,45	0,00	1,88	0,00	2,56	0,00	1,57	0,00	1,88	0,00	1,99	0,00	0,88	0,00	0,97	0,16	1,10	0,38	1,12	0,29	1,36	0,01	1,08	0,39
691	MAOX,SWP70,PDIA3	1,20	0,00	0,91	0,23	1,12	0,12	1,48	0,00	1,88	0,00	1,99	0,00	1,66	0,00	1,16	0,00	1,84	0,00	1,06	0,07	1,29	0,00	1,76	0,00	1,17	0,04	1,53	0,00	1,53	0,00
692	FKBP9	1,02	0,85	0,83	0,09	0,94	0,53	0,95	0,14	0,86	0,05	0,90	0,10	0,54	0,00	0,83	0,00	0,39	0,00	0,75	0,02	0,81	0,07	0,84	0,14	1,53	0,00	0,77	0,03	0,64	0,00
693	COR1B,CBR4	1,19	0,00	1,07	0,38	1,41	0,01	1,75	0,00	2,21	0,00	2,37	0,00	1,58	0,00	1,13	0,00	1,79	0,00	1,04	0,14	1,25	0,00	1,45	0,00	0,99	0,88	1,18	0,03	1,20	0,02
694	HSP7C,LMNB1	1,11	0,01	1,33	0,01	1,17	0,00	1,03	0,16	0,98	0,52	1,05	0,10	0,68	0,00	0,99	0,24	0,75	0,00	0,80	0,00	0,69	0,00	0,64	0,00	0,85	0,03	0,78	0,00	0,73	0,00
697	SDC10,AGM1,NDUS1, WDR72	1,60	0,00	1,44	0,00	1,82	0,00	1,63	0,00	2,22	0,00	2,57	0,00	1,55	0,00	1,08	0,11	1,80	0,00	0,91	0,00	1,08	0,00	1,37	0,00	0,84	0,00	1,26	0,00	1,11	0,00
703	SCFD1	0,92	0,03	1,39	0,51	2,82	0,22	0,94	0,01	1,19	0,00	1,50	0,00	1,03	0,18	1,15	0,00	1,24	0,00	0,93	0,01	1,00	0,98	0,78	0,60	0,74	0,54	0,48	0,31	0,39	0,24
706		1,01	0,87	1,03	0,82	0,82	0,23	0,99	0,91	1,05	0,56	0,93	0,41	1,37	0,07	1,24	0,00	1,22	0,11	0,85	0,03	0,70	0,02	0,89	0,29	0,63	0,00	0,98	0,92	0,77	0,20
708	LMNB1	1,16	0,00	1,19	0,00	0,85	0,00	0,83	0,00	0,73	0,00	0,71	0,00	0,66	0,00	0,73	0,00	0,46	0,00	0,91	0,04	0,89	0,01	0,79	0,00	0,96	0,14	1,07	0,03	0,84	0,00
709	LMNB1	0,68	0,00	0,69	0,00	0,74	0,00	0,84	0,00	0,68	0,00	0,72	0,00	0,78	0,00	0,88	0,00	0,55	0,00	1,11	0,01	1,14	0,00	0,89	0,05	1,26	0,00	0,88	0,00	0,73	0,00
710	GRP75,TF65,PDIA4,H SP7C, HSP71	1,02	0,23	1,15	0,00	1,14	0,00	0,98	0,25	0,93	0,01	1,04	0,08	0,83	0,00	1,05	0,02	0,90	0,00	0,90	0,00	0,86	0,00	0,76	0,00	0,96	0,18	0,86	0,00	0,84	0,00
712	LMNB1	0,95	0,09	1,01	0,64	0,67	0,00	0,90	0,00	0,74	0,00	0,78	0,00	0,65	0,00	0,74	0,00	0,47	0,00	1,06	0,09	1,04	0,25	0,82	0,00	1,11	0,01	1,31	0,00	1,06	0,03
713		1,88	0,00	2,46	0,00	3,18	0,00	1,67	0,00	2,44	0,00	3,25	0,00	2,04	0,00	2,21	0,00	2,51	0,00	0,80	0,00	0,92	0,02	0,89	0,00	0,76	0,00	0,92	0,00	0,67	0,00
714	RANB3	2,14	0,00	2,56	0,00	3,02	0,00	1,52	0,00	1,93	0,00	2,77	0,00	1,90	0,00	2,13	0,00	2,38	0,00	0,75	0,01	0,78	0,01	0,79	0,00	0,73	0,00	0,96	0,52	0,70	0,00
715		1,82	0,00	2,16	0,00	2,91	0,00	1,97	0,00	2,68	0,00	3,19	0,00	1,81	0,00	1,57	0,00	2,40	0,00	0,83	0,00	0,99	0,70	0,95	0,53	0,72	0,02	0,83	0,00	0,82	0,00
717	HSP71,PDIA4	1,99	0,00	2,81	0,00	3,40	0,00	1,80	0,00	2,45	0,00	3,72	0,00	1,61	0,00	2,01	0,00	2,46	0,00	0,76	0,00	0,84	0,00	0,73	0,00	0,75	0,00	0,92	0,00	0,76	0,00
719		1,70	0,00	2,13	0,00	2,51	0,00	1,31	0,00	1,60	0,00	2,24	0,00	1,87	0,00	1,97	0,00	2,25	0,00	0,82	0,01	0,85	0,02	0,80	0,00	0,72	0,00	0,95	0,32	0,70	0,01
720	HSP71	0,90	0,05	0,99	0,87	0,93	0,26	0,89	0,00	0,91	0,00	0,96	0,00	1,02	0,30	0,92	0,01	1,03	0,17	1,01	0,38	1,01	0,34	0,94	0,23	0,82	0,01	1,05	0,05	0,98	0,50
722		2,13	0,00	2,87	0,00	3,77	0,00	1,92	0,00	2,68	0,00	4,17	0,00	1,94	0,00	2,55	0,00	2,72	0,00	0,73	0,00	0,81	0,00	0,76	0,00	0,79	0,00	0,90	0,00	0,64	0,00
723	HSP71,LMNB2,GRP75	1,06	0,01	1,18	0,00	1,22	0,00	1,01	0,22	0,90	0,00	1,08	0,00	0,87	0,00	1,04	0,03	0,90	0,00	0,95	0,01	0,88	0,00	0,75	0,00	0,95	0,15	0,89	0,00	0,80	0,00
729		1,26	0,00	1,40	0,00	1,84	0,00	1,28	0,00	1,35	0,00	1,50	0,00	1,27	0,00	1,23	0,00	1,35	0,00	1,00	1,00	1,00	0,78	0,95	0,42	0,87	0,09	0,80	0,00	0,72	0,00
730		1,28	0,41	2,46	0,13	2,43	0,17	0,79	0,48	1,53	0,08	1,78	0,04	3,51	0,01	3,30	0,00	6,18	0,00	0,93	0,75	0,85	0,47	0,94	0,85	0,42	0,12	1,10	0,79	0,79	0,61

731		1,20	0,46	1,34	0,28	1,36	0,36	0,79	0,19	0,98	0,88	1,11	0,51	0,65	0,10	0,85	0,14	0,46	0,00	0,74	0,07	0,50	0,01	0,82	0,21	0,59	0,01	0,92	0,69	0,31	0,01
732		1,09	0,78	1,34	0,36	1,33	0,49	0,77	0,07	1,01	0,90	1,33	0,02	0,57	0,01	0,84	0,14	0,52	0,01	0,66	0,03	0,53	0,01	0,71	0,10	0,63	0,05	0,94	0,80	0,39	0,05
733		0,80	0,52	0,56	0,26	0,19	0,06	0,80	0,16	1,40	0,07	1,17	0,42	0,68	0,03	0,84	0,06	0,46	0,00	0,38	0,01	0,60	0,05	0,94	0,89	1,06	0,89	2,34	0,02	1,72	0,12
741		1,16	0,62	2,01	0,12	2,24	0,10	0,78	0,33	0,78	0,28	1,00	0,99	1,31	0,00	1,45	0,00	1,48	0,00	1,11	0,59	0,93	0,58	0,64	0,19	0,59	0,14	0,74	0,33	0,54	0,11
742		1,12	0,67	1,79	0,14	1,81	0,15	0,73	0,35	0,84	0,56	1,05	0,86	1,69	0,00	1,75	0,00	1,96	0,00	1,12	0,45	1,16	0,13	0,81	0,40	0,75	0,29	1,00	1,00	0,83	0,48
745	CALR,KAP0	2,02	0,00	2,75	0,00	3,59	0,00	1,80	0,00	2,49	0,00	3,64	0,00	1,96	0,00	2,46	0,00	2,60	0,00	0,78	0,00	0,88	0,00	0,80	0,00	0,81	0,00	0,89	0,00	0,66	0,00
746		1,39	0,16	2,12	0,01	2,29	0,01	1,08	0,68	1,35	0,08	1,83	0,00	1,98	0,00	2,03	0,00	2,62	0,00	0,95	0,72	0,92	0,51	0,79	0,08	0,62	0,01	0,98	0,87	0,74	0,10
747		2,09	0,00	2,84	0,00	3,71	0,00	1,86	0,00	2,60	0,00	3,95	0,00	2,03	0,00	2,55	0,00	2,75	0,00	0,76	0,00	0,85	0,00	0,78	0,00	0,79	0,00	0,90	0,00	0,65	0,00
748		2,05	0,00	2,83	0,00	3,68	0,00	1,83	0,00	2,58	0,00	3,87	0,00	2,03	0,00	2,52	0,00	2,73	0,00	0,77	0,00	0,88	0,00	0,79	0,00	0,79	0,00	0,91	0,00	0,66	0,00
749		1,58	0,00	1,74	0,00	2,24	0,00	1,48	0,00	1,62	0,00	2,36	0,00	1,92	0,00	2,14	0,00	2,27	0,00	0,85	0,00	0,92	0,04	0,84	0,00	0,92	0,01	0,96	0,11	0,76	0,00
750		1,67	0,02	2,12	0,00	2,57	0,00	1,62	0,00	2,15	0,00	3,36	0,00	3,35	0,00	3,13	0,00	4,13	0,00	0,77	0,04	0,97	0,68	0,81	0,00	0,71	0,00	1,04	0,56	0,77	0,01
751	TRXR1	2,00	0,00	2,51	0,00	3,35	0,00	1,65	0,00	2,32	0,00	3,51	0,00	2,17	0,00	2,30	0,00	2,73	0,00	0,75	0,00	0,91	0,00	0,84	0,00	0,76	0,00	0,95	0,18	0,68	0,00
754		2,06	0,00	2,70	0,00	3,40	0,00	1,72	0,00	2,23	0,00	3,36	0,00	1,90	0,00	2,45	0,00	2,45	0,00	0,75	0,00	0,76	0,00	0,74	0,00	0,75	0,01	0,88	0,24	0,60	0,01
756		1,86	0,00	2,26	0,00	2,98	0,00	1,57	0,00	2,02	0,00	2,96	0,00	1,91	0,00	2,25	0,00	2,32	0,00	0,78	0,00	0,89	0,00	0,82	0,00	0,86	0,01	0,91	0,12	0,67	0,00
759		1,94	0,00	2,70	0,00	3,49	0,00	1,70	0,00	2,34	0,00	3,37	0,00	1,89	0,00	2,29	0,00	2,42	0,00	0,80	0,00	0,91	0,00	0,79	0,00	0,80	0,00	0,88	0,00	0,65	0,00
760	TCPG	0,74	0,00	0,62	0,01	0,46	0,00	0,83	0,00	0,85	0,00	0,71	0,00	0,79	0,00	0,90	0,00	0,75	0,00	1,00	0,81	0,95	0,03	1,23	0,21	1,29	0,12	1,41	0,10	1,47	0,07
765	LMNA	0,81	0,00	0,47	0,01	0,36	0,00	0,90	0,08	0,95	0,55	0,67	0,00	0,62	0,00	0,73	0,00	0,51	0,00	1,03	0,40	0,96	0,18	1,86	0,04	1,95	0,02	1,72	0,00	1,76	0,00
767	2AAB,UBQL1,IRGQ	2,46	0,00	3,02	0,00	4,17	0,00	1,78	0,00	2,33	0,00	3,84	0,00	2,15	0,00	2,46	0,00	2,92	0,00	0,74	0,00	0,90	0,00	0,78	0,00	0,84	0,00	0,94	0,01	0,72	0,00
768	MCM6,2AAA	2,41	0,00	2,86	0,00	3,89	0,00	1,87	0,00	2,52	0,00	3,98	0,00	2,28	0,00	2,50	0,00	2,92	0,00	0,71	0,00	0,87	0,00	0,81	0,00	0,80	0,00	0,94	0,02	0,69	0,00
769		2,27	0,00	2,73	0,00	3,76	0,00	1,84	0,00	2,47	0,00	3,63	0,00	2,21	0,00	2,31	0,00	2,76	0,00	0,74	0,00	0,87	0,00	0,83	0,00	0,76	0,00	0,89	0,00	0,66	0,00
770	HNRPK	2,31	0,00	2,77	0,00	3,79	0,00	1,91	0,00	2,64	0,00	3,85	0,00	2,34	0,00	2,47	0,00	2,85	0,00	0,74	0,00	0,87	0,00	0,85	0,00	0,76	0,00	0,90	0,00	0,64	0,00
771	TCPG	0,91	0,01	0,40	0,02	0,10	0,00	0,92	0,00	0,91	0,01	0,83	0,00	0,83	0,00	0,99	0,46	0,84	0,00	0,96	0,04	0,92	0,01	2,17	0,05	2,49	0,02	7,87	0,00	8,50	0,00
774	TM1L2	2,19	0,00	2,79	0,00	3,75	0,00	1,51	0,00	2,16	0,00	3,25	0,00	1,87	0,00	2,04	0,00	2,38	0,00	0,72	0,00	0,85	0,00	0,81	0,00	0,73	0,00	0,91	0,09	0,63	0,00
776		2,81	0,00	3,49	0,00	4,87	0,00	1,94	0,00	2,65	0,00	4,52	0,00	2,42	0,00	2,83	0,00	3,46	0,00	0,71	0,00	0,87	0,00	0,78	0,00	0,82	0,00	0,95	0,19	0,72	0,00
777	LKHA4	0,71	0,00	0,79	0,03	0,87	0,05	0,73	0,00	0,86	0,00	0,83	0,00	0,65	0,00	0,78	0,00	0,70	0,00	0,99	0,68	0,93	0,03	1,06	0,52	1,01	0,89	0,92	0,24	0,83	0,02
778	DPYL2,LKHA4	0,79	0,00	0,39	0,02	0,07	0,00	0,83	0,00	0,92	0,07	0,74	0,00	0,82	0,01	0,92	0,04	0,85	0,01	0,93	0,00	0,87	0,01	2,11	0,05	1,96	0,08	8,99	0,00	9,57	0,00
782		1,94	0,00	2,23	0,00	2,94	0,00	1,69	0,00	2,18	0,00	3,08	0,00	2,02	0,00	2,21	0,00	2,38	0,00	0,77	0,00	0,86	0,00	0,86	0,01	0,81	0,00	0,92	0,04	0,67	0,00
785		2,09	0,00	2,86	0,00	3,78	0,00	1,86	0,00	2,67	0,00	3,85	0,00	1,98	0,00	2,45	0,00	2,59	0,00	0,76	0,00	0,89	0,00	0,80	0,00	0,80	0,00	0,88	0,00	0,64	0,00
787	HNRPK	1,27	0,00	1,20	0,00	1,23	0,00	1,27	0,00	1,14	0,02	1,27	0,00	1,16	0,00	1,33	0,00	1,14	0,00	0,88	0,00	0,77	0,00	0,84	0,01	0,94	0,14	0,90	0,03	0,78	0,00
788	HNRPK	0,98	0,43	0,95	0,16	0,81	0,00	1,05	0,00	0,81	0,00	0,87	0,00	0,92	0,00	1,10	0,00	0,74	0,00	1,03	0,11	1,00	0,98	0,82	0,00	1,24	0,00	1,03	0,40	0,97	0,46
790	HNRPK	1,11	0,00	1,00	0,89	0,92	0,02	1,15	0,00	0,98	0,11	0,92	0,00	1,08	0,00	1,19	0,00	0,95	0,05	0,92	0,00	0,79	0,00	0,87	0,00	0,97	0,11	0,89	0,00	0,84	0,00
791	CPNE1	0,73	0,00	0,74	0,00	0,59	0,00	0,79	0,00	0,73	0,00	0,71	0,00	0,71	0,00	0,83	0,00	0,66	0,00	1,04	0,02	0,97	0,04	0,94	0,12	1,13	0,01	1,17	0,00	1,13	0,00
793	TCPA	0,83	0,00	0,79	0,00	0,61	0,00	0,89	0,00	0,83	0,00	0,71	0,00	0,92	0,00	0,98	0,11	0,86	0,00	0,97	0,05	0,89	0,00	0,96	0,17	1,00	0,96	1,06	0,09	1,14	0,00
796	TCPQ,HNRPK	0,84	0,00	0,71	0,00	0,64	0,00	0,94	0,00	0,70	0,00	0,73	0,00	0,96	0,00	0,96	0,00	0,69	0,00	1,11	0,00	1,14	0,00	0,97	0,26	1,34	0,00	1,13	0,01	1,08	0,03
798	TCPE,PNCB,TCPQ,HDAC2	0,87	0,00	0,87	0,00	0,68	0,00	0,87	0,00	0,84	0,00	0,70	0,00	0,85	0,00	0,89	0,00	0,82	0,00	0,92	0,00	0,81	0,00	0,88	0,01	0,86	0,00	0,94	0,04	1,00	0,97
801	2AAA	1,05	0,00	1,13	0,05	1,19	0,00	0,95	0,06	0,95	0,10	1,09	0,01	0,91	0,02	1,03	0,32	0,98	0,60	0,95	0,01	0,90	0,00	0,89	0,06	0,95	0,34	0,96	0,13	0,86	0,00
802		1,71	0,00	2,20	0,00	2,88	0,00	1,30	0,00	1,57	0,00	2,16	0,00	1,17	0,00	1,53	0,00	1,34	0,00	0,78	0,00	0,89	0,01	0,73	0,00	0,91	0,00	0,78	0,01	0,61	0,00
804	TCPE	1,12	0,00	1,05	0,26	0,83	0,00	1,02	0,14	0,90	0,00	0,84	0,00	0,80	0,00	0,96	0,19	0,80	0,00	0,88	0,00	0,74	0,00	0,82	0,00	0,95	0,21	0,98	0,41	1,00	0,97
809	ULA1	0,82	0,00	0,70	0,00	0,56	0,00	0,80	0,00	0,71	0,00	0,62	0,00	0,75	0,00	0,87	0,00	0,68	0,00	1,00	0,83	0,91	0,00	1,03	0,57	1,24	0,00	1,14	0,11	1,23	0,03
810	PEPD,PDIA3,TBA1B	0,93	0,00	0,97	0,65	1,14	0,00	0,95	0,00	0,94	0,00	0,84	0,00	1,07	0,01	0,82	0,00	0,91	0,00	1,14	0,00	1,15	0,00	1,07	0,28	0,84	0,03	0,82	0,00	0,80	0,00
813	CPNE2,P4HA1,TCPA,PDIA	0,99	0,89	1,01	0,84	1,27	0,01	1,02	0,40	1,01	0,49	1,22	0,00	0,97	0,34	1,15	0,00	0,92	0,03	0,91	0,04	0,85	0,01	0,89	0,09	0,99	0,87	0,86	0,02	0,64	0,00
818	UBP14	0,85	0,00	0,89	0,00	0,69	0,00	0,88	0,00	0,80	0,00	0,70	0,00	0,80	0,00	0,89	0,00	0,79	0,00	0,99	0,06	0,88	0,00	0,86	0,00	0,94	0,06	0,98	0,47	1,08	0,04
820	PDIA1	1,00	0,64	1,01	0,84	1,13	0,00	0,95	0,00	0,87	0,00	0,82	0,00	1,01	0,40	0,91	0,00	0,80	0,00	1,07	0,00	1,03	0,03	0,96	0,47	0,92	0,14	0,82	0,00	0,72	0,00
821	UBP14,IMA2	1,09	0,01	0,99	0,73	0,87	0,00	1,04	0,05	0,83	0,00	0,89	0,00	0,96	0,02	1,05	0,06	0,91	0,00	0,95	0,02	0,87	0,00	0,83	0,01	1,05	0,27	1,01	0,80	1,03	0,42
822	HSP7C,AINX	0,93	0,02	0,83	0,00	0,68	0,00	0,93	0,16	0,84	0,01	0,72	0,00	0,92	0,05	0,96	0,30	0,85	0,00	0,97	0,16	0,90	0,00	0,98	0,44	1,05	0,21	1,02	0,66	1,13	0,01
824	PDIA1	1,32	0,00	1,49	0,00	1,90	0,00	1,18	0,00	0,93	0,00	1,03	0,06	0,96	0,00	1,16	0,00	0,89	0,00	1,00	0,55	0,87	0,00	0,69	0,00	0,93	0,03	0,60	0,00	0,56	0,00
825	GDIA,ANXA3,NUCB1	1,23	0,00	1,50	0,00	1,84	0,00	1,08	0,00	1,23	0,00	1,54	0,00	1,17	0,00	1,27	0,00	1,32	0,00	0,90	0,00	0,92	0,00	0,84	0,00	0,82	0,00	0,86	0,00	0,70	0,00
826	CH60,STK3	1,30	0,00	1,69	0,00	1,35	0,00	1,14	0,00	1,14	0,02	1,17	0,00	0,80	0,00	1,16	0,01	0,91	0,04	0,83	0,00	0,68	0,00	0,64	0,00	0,75	0,00	0,82	0,00	0,73	0,00
827	CH60	1,63	0,00	1,71	0,00	1,46	0,00	1,41	0,00	1,17	0,00	1,26	0,00	1,08	0,00	1,38	0,00	1,14	0,00	0,86	0,00	0,71	0,00	0,68	0,00	0,86	0,01	0,86	0,00	0,83	0,00
828	GDIA,VTDB,2AAA	1,77	0,00	2,23	0,00	2,89	0,00	1,42	0,00	1,70	0,00	2,40	0,00	1,66	0,00	1,96	0,00	2,07	0,00	0,82	0,00	0,87	0,00	0,78	0,00	0,81	0,00	0,85	0,00	0,66	0,00
829		0,89	0,05	0,80	0,04	0,89	0,26	0,81	0,04	0,99	0,89	1,31	0,01	0,65	0,02	0,74	0,02	0,71	0,02	0,87	0,03	0,96	0,73	1,17	0,10	1,21	0,06	1,38	0,01	1,03	0,78
831	CALR	1,03	0,01	1,18	0,00	1,25	0,00	1,16	0,00	0,97	0,03	1,00	0,81	0,90	0,06	1,05	0,42	0,80	0,00	1,21	0,00	1,04	0,01	0,88	0,00	1,06	0,23	0,85	0,00	0,77	0,00
835	TBA1B,TBAK,TBA3,IFIT3	0,95	0,13	0,87	0,04	0,72	0,00	0,79	0,00	0,82	0,00	0,69	0,00	1,01	0,55	0,83	0,00	0,87	0,00	1,03	0,03	0,96	0,04	1,16	0,02	0,86	0,03	1,18	0,00	1,08	0,03
836	PDIA3,TBA1B	1,31	0,05	1,58	0,01	1,97	0,00	1,30	0,00	1,24	0,01	1,75	0,00	1,28	0,00	1,29	0,00	1,29	0,05	1,00	0,97	1,08	0,34	0,79	0,03	0,91	0,25	0,90	0,12	0,73	0,03
837	TRXR1	0,79	0,00	0,37	0,01	0,10	0,00	0,94	0,03	1,03	0,65	1,00	0,86	0,94	0,08	0,99	0,45	1,12	0,01	1,02	0,36	0,95	0,11	2,40	0,03	2,14	0,04	8,38	0,00	8,66	0,00
840	TBA1B,TBAK,TBA3,VIME	0,95	0,06	0,78	0,00	0,85	0,00	1,00	0,89	0,96	0,03	0,83	0,00	1,04	0,08	0,85	0,00	0,91	0,01	1,18	0,00	1,14	0,00	1,37	0,00	1,12	0,00	1,10	0,01	1,11	0,01
841		2,13	0,00	3,08	0,00	4,30	0,00	2,04	0,00	2,81	0,00	3,99	0,00	2,01	0,00	2,67	0,00	2,81	0,00	0,84	0,00	0,87	0,00	0,81	0,00	0,80	0,00	0,82	0,00	0,60	0,00
842	NP1L4	1,86	0,00	2,45	0,00	3,01	0,00	1,65	0,00	2,18	0,00	3,20	0,00	1,85	0,00	2,30	0,00	2,43	0,00	0,79	0,00	0,87	0,00	0,80	0,00	0,82	0,00	0,95	0,03	0,70	0,00
843	PNCB,FKBP4,TCPE,TCPQ,HDAC2	0,81	0,00	0,80	0,00	0,66	0,00	0,82	0,00	0,74	0,00	0,62	0,00	0,75	0,00	0,86	0,00	0,72	0,00	0,97	0,03	0,81	0,00	0,89	0,02	0,94	0,14	0,90	0,01	0,97	0,20
845	NP1L1	1,76	0,00	2,29	0,00	2,82	0,00	1,48	0,00	1,76	0,00	2,59	0,00	1,46	0,00	1,81	0,00	1,80	0,00	0,84	0,00	0,86	0,00	0,77	0,00	0,82	0,00	0,92	0,04	0,67	0,00
846	NP1L4	1,72	0,00	2,27	0,00	2,81	0,00	1,55	0,00	1,98	0,00	2,86	0,00	1,74	0,00	2,09	0,00	2,19	0,00	0,82	0,00	0,91	0,00	0,80	0,00	0,83	0,00	0,93	0,01	0,70	0,00
847	TCPE,HNRPK	0,95	0,00	0,93	0,09	0,69	0,00	0,93	0,03	0,81	0,00	0,73	0,00	0,64	0,00	0,91	0,01	0,64	0,00	0,92	0,00	0,76	0,00	0,83	0,00	1,10	0,03	1,01	0,86	1,05	0,21
848		0,96	0,26	1,01	0,87	1,07	0,15	0,96	0,21	0,94	0,03	1,03	0,23	0,92	0,06	0,98	0,38	1,03	0,09	0,98	0,59	1,00	0,95	0,92	0,16	1,02	0,77	0,94	0,16	1,00	0,97
849	TRXR1	0,88	0,00	0,52	0,04	0,34	0,02	0,89	0,00	0,90	0,00	0,83	0,00	0,87	0,01	0,96	0,07	0,95	0,05	0,95	0,01	0,87	0,01	1,62	0,14	1,64	0,13	2,24	0,09	2,42	0,06
853	STABP	1,77	0,00	2,47	0,00	3,03	0,00	1,59	0,00	2,29	0,00	3,13	0,00	1,87	0,00	2,17	0,00	2,40	0,00	0,78	0,00	0,89	0,00	0,81	0,00	0,74	0,00	0,91	0,04	0,67	0,00
854	PDIA3,HNRPF,VATB2	1,12	0,00	1,30	0,00	1,58	0,00	1,12	0,00	0,90	0,00	0,94	0,01	0,85	0,00	1,04	0,01	0,78	0,00	1,05	0,00	0,94	0,00	0,73	0,00	1,00	0,91	0,63	0,00	0,61	0,00
855		0,91	0,00	0,37	0,03	0,14	0,00	0,92	0,00	0,99	0,82	0,81	0,00	0,92	0,01	0,98	0,10	0,95	0,01	0,91	0,01	0,86	0,00	2,42	0,07	2,28	0,08	5,16	0,00	5,75	0,00
858		1,67	0,00	2,26	0,00	2,75	0,00	1,43	0,00	1,97	0,00	2,59	0,00	1,65	0,00	1,93	0,00	2,03	0,00	0,80	0,00	0,88	0,00	0,81	0,00	0,76	0,00	0,88	0,00	0,65	0,00
859	TBA1B,TBA1C,VIME,TBA3,TBA6,DBNL,TBB5	1,00	0,78	0,95	0,05	0,79	0,00	0,94	0,00	0,84	0,00	0,77	0,00	0,81	0,00	0,91	0,01	0,74	0,00	0,96	0,01	0,87	0,00	0,90	0,00	1,03	0,22	1,00	0,86	1,01	0,52
864	PDIA3,VATB2	1,07	0,01	1,28	0,00	1,45	0,00	1,02	0,70	1,00	0,97	0,91	0,08	0,82	0,00	0,88	0,01	0,70	0,00	0,95	0,20	0,95	0,15	0,78	0,00	0,86	0,01	0,63	0,00	0,60	0,00
866		1,26	0,01	0,58	0,10	1,95	0,51	1,27	0,03	1,64	0,00	1,73	0,00	1,17	0,49	1,17	0,03	1,56	0,00	0,89	0,00	0,97	0,88	2,51	0,01	2,11	0,02	0,79	0,77	0,83	0,82
871	FKBP4	0,88	0,00	0,96	0,13	0,72	0,00	0,94	0,00	0,92	0,00	0,77	0,00	0,80	0,00	0,91	0,01	0,78	0,00	0,97	0,03	0,86	0,00	0,87	0,00	0,89	0,00	0,98	0,34	1,02	0,35
875	TBB2C,TBB3,TBB5	0,78	0,00	0,77	0,00	0,55	0,00	0,77	0,00	0,75	0,00	0,66	0,00	0,82	0,00	0,85	0,00	0,72	0,00	1,02	0,27	0,93	0,00	1,00	0,93	0,99	0,73	1,23	0,00	1,16	0,00
876	TBAK	0,76	0,00	0,85	0,01	0,64	0,00	0,77	0,00	0,70	0,00	0,57	0,00	0,61	0,00	0,83	0,00	0,57	0,00	0,99	0,63	0,81	0,00	0,81	0,01	0,98	0,74	0,88	0,08	0,89	0,12
878	FKBP4,HMCS1,HNRPK	0,86	0,00	0,95	0,10	0,71	0,00	0,96	0,00	0,94	0,00	0,79	0,00	0,89	0,00	0,99	0,34	0,88	0,00	0,94	0,00	0,81	0,00	0,83	0,00	0,81	0,00	0,93	0,05	0,96	0,16
879	HNRPK,TBL1R,HMCS1,TBA3	0,81	0,00	0,79	0,00	0,60	0,00	0,75	0,00	0,67	0,00	0,55	0,00	0,51	0,00	0,65	0,00	0,53	0,00	0,93	0,01	0,75	0,00	0,86	0,01	0,96	0,40	0,93	0,13	1,04	0,40
883		0,82	0,00	0,99	0,69	1,06	0,00	0,86	0,00	0,82	0,00	0,83	0,00	0,79	0,00	0,88	0,00	0,69	0,00	1,04	0,07	1,03	0,09	0,82	0,00	0,96	0,16	0,78	0,00	0,71	0,00
884	K2C8,UAP56,IF2B,GSHB	0,97	0,10	0,92	0,05	0,76	0,00	0,95	0,00	0,95	0,00	0,80	0,00	1,07	0,01	0,85	0,00	0,80	0,00	1,05	0,01	1,09	0,00	1,09	0,03	0,91	0,04	1,13	0,00	1,05	0,12
885	SYWC	0,78	0,00	0,73	0,00	0,52	0,00	0,83	0,00	0,79	0,00	0,67	0,00	0,76	0,00	0,87	0,00	0,70	0,00	1,13	0,00	1,17	0,00	1,15	0,06	1,42	0,00	1,36	0,01	1,60	0,00
888	TCPB,RUVB1	0,87	0,00	0,62	0,06	0,46	0,01	0,93	0,00	0,86	0,00	0,73	0,00	0,88	0,00	0,97	0,01	0,81	0,00	0,98	0,26	0,89	0,00	1,28	0,32	1,38	0,19	1,47	0,11	1,56	0,07
890	TBA1B	0,80	0,00	0,78	0,00	0,59	0,00	0,81	0,00	0,65	0,00	0,58	0,00	0,75	0,00	0,84	0,00	0,63	0,00	1,15	0,04	1,20	0,01	0,94	0,20	1,38	0,00	1,11	0,02	1,38	0,00
892	K2C8	1,27	0,00	1,15	0,04	0,97	0,45	1,14	0,00	0,93	0,02	0,89	0,00	0,90	0,00	1,12	0,00	0,73	0,00	0,96	0,09	0,90	0,00	0,86	0,02	1,23	0,00	0,97	0,35	0,95	0,21
893	DD19A,TBG1,PPP5	0,84	0,00	0,38	0,01	0,14	0,00	0,86	0,00	0,85	0,00	0,72	0,00	0,85	0,00	0,87	0,00	0,83	0,00	0,95	0,15	0,90	0,03	2,06	0,05	2,01	0,05	4,73	0,00	5,26	0,00
894	AL1B1	1,01	0,86	0,36	0,03	0,09	0,00	1,03	0,23	0,93	0,02	0,87	0,00	0,92	0,00	1,05	0,00	0,91	0,00	0,92	0,03	0,84	0,00	2,34	0,08	2,70	0,04	8,98	0,00	9,64	0,00
897	CALR,NP1L1	1,13	0,01	1,25	0,00	1,23	0,00	1,23	0,00	0,94	0,02	0,97	0,07	0,90	0,00	1,22	0,00	0,84	0,00	1,26	0,00	1,11	0,00	0,86	0,00	1,36	0,00	0,90	0,03	0,95	0,15
898		1,22	0,00	1,56	0,00	1,82	0,00	1,12	0,04	1,27	0,00	1,52	0,00	1,18	0,02	1,32	0,00	1,23	0,01	0,94	0,22	1,03	0,45	0,83	0,00	0,90	0,01	0,86	0,00	0,72	0,00
899	K2C8	1,43	0,00	1,27	0,00	1,04	0,16	1,24	0,00	1,18	0,00	1,02	0,33	1,05	0,03	1,35	0,00	0,91	0,01	0,86	0,00	0,81	0,00	0,93	0,10	1,17	0,00	0,98	0,50	0,98	0,40
900	K2C8	1,17	0,00	1,00	0,97	0,85	0,00	1,11	0,00	1,06	0,02	0,88	0,00	1,08	0,00	1,09	0,01	0,86	0,00	0,90	0,00	0,87	0,00	1,01	0,57	1,03	0,28	0,99	0,71	0,97	0,17
902	DD19A,MPPA	0,74	0,00	0,31	0,00	0,23	0,00	0,73	0,00	0,68	0,00	0,53	0,00	0,73	0,00	0,77	0,00	0,56	0,00	0,93	0,02	0,92	0,02	2,03	0,05	2,33	0,02	2,22	0,05	2,34	0,04
904	TBA1B,TBB5	0,75	0,00	0,69	0,00	0,65	0,00	0,83	0,00	0,78	0,00	0,65	0,00	0,81	0,00	0,96	0,41	0,93	0,28	0,96	0,20	0,80	0,00	0,99	0,75	1,03	0,49	0,87	0,09	1,06	0,41
906	TBA1C,PTRF,TBA4A,TBB5	0,73	0,00	0,67	0,00	0,58	0,00	0,81	0,00	0,75	0,00	0,62	0,00	0,75	0,00	0,87	0,00	0,71	0,00	0,95	0,01	0,79	0,00	0,95	0,27	1,01	0,87	0,93	0,15	0,95	0,69
908	SYHC	0,67	0,00	0,70	0,00	0,45	0,00	0,78	0,00	0,73	0,00	0,57	0,00	0,74	0,00	0,82	0,00	0,66	0,00	1,12	0,01	1,05	0,23	0,99	0,59	1,10	0,00	1,20	0,01	1,36	0,00
909	CNDP2,HNRH1	0,66	0,00	0,65	0,00	0,49	0,00	0,70	0,00	0,59	0,00	0,48	0,00	0,74	0,00	0,76	0,00	0,56	0,00	1,08	0,09	1,08	0,10	0,93	0,06	1,13	0,00	1,00	0,94	1,10	0,01
913	K2C8,PSMD5	1,19	0,00	1,03	0,40	0,83	0,01	1,17	0,00	1,47	0,00	1,08	0,02	1,20	0,01	1,37	0,00	1,16	0,03	0,81	0,00	0,78	0,00	1,18	0,00	1,03	0,43	1,09	0,16	1,08	0,15

916		2,06	0,00	2,94	0,00	3,88	0,00	1,87	0,00	2,65	0,00	3,67	0,00	1,99	0,00	2,52	0,00	2,70	0,00	0,80	0,00	0,87	0,00	0,80	0,00	0,77	0,00	0,83	0,00	0,63	0,00
918	HNRH1,CNDP2,SNX6	0,63	0,00	0,66	0,00	0,51	0,00	0,79	0,00	0,69	0,00	0,61	0,00	0,77	0,00	0,86	0,00	0,62	0,00	1,19	0,00	1,19	0,00	0,98	0,52	1,25	0,00	1,13	0,00	1,18	0,00
920		0,60	0,00	0,54	0,00	0,42	0,00	0,60	0,00	0,50	0,00	0,38	0,00	0,68	0,00	0,75	0,00	0,54	0,00	1,15	0,01	1,14	0,02	1,07	0,12	1,39	0,00	1,06	0,24	1,32	0,00
921	SEPT8,HNRH1	0,67	0,00	0,66	0,00	0,54	0,00	0,72	0,00	0,61	0,00	0,54	0,00	0,75	0,00	0,79	0,00	0,60	0,00	1,12	0,04	1,16	0,01	0,96	0,32	1,24	0,00	1,05	0,31	1,18	0,00
922	RHG01,HNRH2	0,67	0,00	0,40	0,00	0,20	0,00	0,71	0,00	0,69	0,00	0,58	0,00	0,70	0,00	0,75	0,00	0,61	0,00	0,96	0,23	0,99	0,78	1,57	0,05	1,78	0,02	2,65	0,04	2,94	0,02
923	HNRH1,CNDP2	0,69	0,00	0,76	0,00	0,56	0,00	0,79	0,00	0,57	0,00	0,51	0,00	0,72	0,00	0,86	0,00	0,51	0,00	1,17	0,03	1,21	0,01	0,77	0,00	1,30	0,00	0,95	0,28	1,06	0,16
925	CALR,TBB2C	1,06	0,03	1,16	0,00	1,19	0,00	1,14	0,00	0,91	0,00	0,87	0,00	0,87	0,00	1,09	0,00	0,79	0,00	1,16	0,00	1,02	0,31	0,84	0,00	1,16	0,00	0,79	0,00	0,83	0,00
926	NP1L1	1,04	0,45	1,00	0,95	0,75	0,01	1,16	0,00	0,79	0,00	0,65	0,00	0,92	0,07	1,16	0,00	0,85	0,02	1,05	0,29	0,71	0,00	0,74	0,00	0,94	0,27	0,82	0,05	0,93	0,42
927	K1C17,ATPB,PDIA6	1,18	0,00	1,30	0,00	1,25	0,00	1,14	0,00	0,94	0,00	0,92	0,00	0,92	0,00	1,11	0,00	0,81	0,00	0,92	0,00	0,80	0,00	0,69	0,00	0,87	0,00	0,70	0,00	0,66	0,00
929	NP1L1,SYAP1	1,09	0,03	1,00	0,97	0,75	0,01	1,20	0,00	0,92	0,00	0,74	0,00	0,91	0,05	1,13	0,00	0,96	0,32	0,93	0,07	0,63	0,00	0,77	0,00	0,86	0,01	0,83	0,05	0,97	0,67
934	SYDC,HNRH1,PTN1,BLMH	0,77	0,00	0,32	0,02	0,01	0,00	0,81	0,00	0,71	0,00	0,61	0,00	0,70	0,00	0,81	0,00	0,61	0,00	1,02	0,53	1,00	0,92	2,19	0,10	2,79	0,03	68,19	0,00	76,90	0,00
935	RD23A	0,87	0,03	0,73	0,00	0,55	0,00	0,88	0,09	0,55	0,00	0,46	0,00	0,93	0,06	0,87	0,00	0,54	0,00	1,30	0,01	1,06	0,21	0,97	0,38	1,20	0,00	1,07	0,17	0,99	0,76
936	BASP	0,85	0,01	0,68	0,00	0,55	0,00	0,97	0,45	0,60	0,00	0,51	0,00	1,06	0,05	0,95	0,04	0,65	0,00	1,37	0,00	1,20	0,00	1,07	0,02	1,35	0,00	1,12	0,14	1,14	0,09
937	SCRN1	0,69	0,00	0,66	0,00	0,39	0,00	0,75	0,00	0,62	0,00	0,53	0,00	0,68	0,00	0,76	0,00	0,60	0,00	1,15	0,00	1,05	0,04	0,99	0,58	1,21	0,00	1,44	0,00	1,65	0,00
938		0,51	0,01	0,31	0,00	0,19	0,00	0,76	0,11	0,34	0,00	0,26	0,00	1,05	0,37	1,02	0,68	0,65	0,01	1,68	0,01	0,76	0,03	1,26	0,03	1,24	0,01	1,54	0,02	1,28	0,16
940	GDIB	0,67	0,00	0,65	0,00	0,49	0,00	0,69	0,00	0,67	0,00	0,54	0,00	0,70	0,00	0,75	0,00	0,63	0,00	1,03	0,10	0,98	0,22	1,03	0,62	1,08	0,24	1,08	0,49	1,18	0,15
941	CD2B2	0,91	0,12	0,93	0,22	0,69	0,00	0,92	0,13	0,64	0,00	0,50	0,00	0,74	0,00	0,92	0,00	0,56	0,00	1,15	0,07	0,80	0,01	0,77	0,00	0,97	0,54	0,82	0,03	0,80	0,03
942	DCTN2,PABP2	0,93	0,00	0,92	0,03	0,81	0,00	0,94	0,00	0,80	0,00	0,74	0,00	0,83	0,00	0,93	0,01	0,73	0,00	1,01	0,66	0,91	0,00	0,86	0,00	1,02	0,47	0,91	0,04	0,92	0,03
943	GSHB,K2C8,DDX39	0,80	0,00	0,70	0,00	0,50	0,00	0,82	0,01	0,67	0,00	0,62	0,00	0,75	0,00	0,87	0,00	0,59	0,00	1,07	0,18	0,93	0,07	0,99	0,83	1,23	0,00	1,29	0,01	1,18	0,01
948		0,85	0,00	0,79	0,00	0,55	0,00	0,99	0,29	0,76	0,00	0,70	0,00	0,88	0,00	0,93	0,01	0,74	0,00	1,06	0,02	0,83	0,00	0,89	0,01	0,96	0,22	1,17	0,02	1,08	0,10
949	RUVB2,NEMO	0,98	0,07	0,96	0,23	0,78	0,00	0,95	0,00	0,87	0,00	0,78	0,00	0,86	0,00	0,95	0,00	0,78	0,00	0,93	0,00	0,86	0,00	0,86	0,00	0,96	0,21	0,95	0,10	0,97	0,24
950	SHLB2,AL9A1	0,74	0,08	1,02	0,77	0,58	0,00	0,91	0,07	0,97	0,54	0,69	0,00	0,84	0,00	0,95	0,04	0,90	0,00	0,99	0,95	0,82	0,33	0,76	0,01	0,67	0,00	0,96	0,46	1,12	0,04
951	BLMH,LA,PA2G4	0,74	0,00	0,69	0,02	0,59	0,00	0,80	0,00	0,79	0,00	0,73	0,00	0,71	0,00	0,86	0,00	0,71	0,00	1,01	0,50	0,97	0,15	1,08	0,57	1,26	0,10	1,16	0,01	1,22	0,00
953	ENOG,RBBP7	0,76	0,00	0,81	0,00	0,54	0,00	0,86	0,00	0,75	0,00	0,64	0,00	0,74	0,00	0,85	0,00	0,62	0,00	1,04	0,01	0,86	0,00	0,84	0,00	0,91	0,01	1,09	0,01	1,00	0,85
954	PSMD5,SH3G1	0,77	0,00	0,71	0,00	0,51	0,00	0,75	0,00	0,71	0,00	0,59	0,00	0,75	0,00	0,84	0,01	0,67	0,00	1,04	0,10	0,94	0,04	1,08	0,00	1,15	0,01	1,25	0,00	1,27	0,00
956	SNX6,HNRH1	0,68	0,00	0,70	0,00	0,50	0,00	0,78	0,00	0,71	0,00	0,60	0,00	0,80	0,00	0,84	0,00	0,67	0,00	1,13	0,00	1,13	0,00	0,99	0,71	1,16	0,00	1,18	0,02	1,32	0,00
957	F10A1,PDIA6,TXND4	1,05	0,01	1,10	0,01	1,26	0,00	1,06	0,00	0,88	0,00	0,87	0,00	0,95	0,00	1,04	0,00	0,83	0,00	1,03	0,02	0,95	0,00	0,82	0,00	1,00	0,87	0,70	0,00	0,69	0,00
958	CDC37,PRS6B,DCTN2,PDIA6	0,81	0,00	0,84	0,00	0,66	0,00	0,90	0,00	0,80	0,00	0,71	0,00	0,77	0,00	0,93	0,01	0,73	0,00	1,05	0,02	0,87	0,00	0,89	0,01	1,02	0,60	1,01	0,83	1,03	0,26
959	KAP0,TXND5	0,71	0,00	0,70	0,00	0,55	0,00	0,78	0,00	0,62	0,00	0,54	0,00	0,72	0,00	0,76	0,00	0,52	0,00	1,07	0,01	0,98	0,44	0,86	0,00	1,06	0,11	0,95	0,26	0,91	0,05
960	GSHB	0,88	0,00	0,88	0,00	0,71	0,00	0,92	0,00	0,80	0,00	0,74	0,00	0,86	0,00	0,92	0,00	0,73	0,00	1,08	0,00	1,06	0,02	0,94	0,00	1,12	0,00	1,07	0,05	1,11	0,00
963		1,11	0,00	1,19	0,00	1,16	0,00	1,24	0,00	0,93	0,00	0,88	0,00	0,88	0,00	1,07	0,00	0,81	0,00	1,24	0,00	1,03	0,14	0,87	0,00	1,17	0,00	0,85	0,00	0,90	0,01
964	IF2B,ARP3,QCR1,PDIA3	0,80	0,00	0,80	0,00	0,62	0,00	0,83	0,00	0,65	0,00	0,64	0,00	0,74	0,00	0,87	0,01	0,65	0,00	1,10	0,01	0,96	0,07	0,86	0,00	1,13	0,00	1,09	0,10	1,08	0,08
965	ARP3	0,61	0,00	0,70	0,00	0,42	0,00	0,68	0,00	0,68	0,00	0,51	0,00	0,65	0,00	0,74	0,00	0,60	0,00	1,05	0,36	0,94	0,25	0,92	0,01	0,94	0,02	1,16	0,02	1,28	0,00
971	HNRH1,GDIB,2ABA	0,78	0,00	0,82	0,00	0,59	0,00	0,78	0,00	0,77	0,00	0,69	0,00	0,70	0,00	0,85	0,00	0,68	0,00	0,96	0,14	0,89	0,00	0,89	0,00	1,02	0,36	1,11	0,03	1,14	0,01
972	SHLB2	0,62	0,00	0,72	0,00	0,48	0,00	0,75	0,00	0,61	0,00	0,56	0,00	0,69	0,00	0,77	0,00	0,54	0,00	1,30	0,07	1,24	0,14	0,91	0,10	1,19	0,00	1,25	0,00	1,25	0,00
973	TBG1,MPPB	0,74	0,00	0,79	0,00	0,53	0,00	0,84	0,00	0,83	0,00	0,72	0,00	0,72	0,00	0,93	0,00	0,69	0,00	0,99	0,73	0,89	0,00	0,92	0,21	1,08	0,23	1,19	0,11	1,21	0,08
974	SYDC	0,86	0,00	0,84	0,05	0,65	0,00	0,96	0,01	0,93	0,00	0,80	0,00	0,99	0,77	1,13	0,00	0,97	0,13	0,95	0,05	0,82	0,00	0,95	0,57	0,96	0,60	1,05	0,36	1,05	0,34
975	VIME	1,42	0,00	1,24	0,00	1,07	0,21	1,48	0,00	1,10	0,00	1,00	0,81	1,47	0,00	1,49	0,00	1,35	0,00	0,99	0,79	0,62	0,00	0,84	0,00	0,72	0,00	0,88	0,03	0,75	0,00
977	TXND5,HNRPF,TBAK	0,91	0,00	1,04	0,29	1,15	0,00	1,02	0,30	0,84	0,00	0,72	0,00	0,87	0,00	0,96	0,21	0,65	0,00	1,08	0,00	1,00	0,85	0,78	0,00	0,97	0,40	0,61	0,00	0,59	0,00
978	APMAP,SNX6	0,90	0,00	0,91	0,01	0,82	0,00	0,78	0,00	0,77	0,00	0,69	0,00	0,84	0,00	0,88	0,00	0,72	0,00	0,89	0,00	0,88	0,00	0,87	0,00	0,92	0,01	0,86	0,02	0,83	0,01
981	K1C17	1,04	0,27	0,81	0,00	0,68	0,00	0,74	0,00	0,78	0,00	0,62	0,00	0,67	0,00	0,71	0,00	0,76	0,00	0,56	0,00	0,47	0,00	0,75	0,00	0,63	0,00	0,72	0,00	0,81	0,00
982	PDIA6	1,28	0,00	1,36	0,00	1,60	0,00	1,20	0,00	0,91	0,01	1,01	0,78	0,99	0,52	1,15	0,00	0,98	0,23	0,97	0,10	0,88	0,00	0,70	0,00	0,96	0,18	0,65	0,00	0,69	0,00
983	DNJA2,APMAP,SNX6	0,83	0,00	0,84	0,00	0,65	0,00	0,79	0,00	0,79	0,00	0,68	0,00	0,74	0,01	0,88	0,13	0,69	0,00	0,91	0,02	0,84	0,00	0,88	0,03	0,98	0,70	0,99	0,83	0,99	0,88
985		0,73	0,00	0,60	0,00	0,37	0,00	0,77	0,00	0,64	0,00	0,49	0,00	0,90	0,01	0,89	0,00	0,61	0,00	1,16	0,01	1,04	0,19	1,15	0,00	1,24	0,00	1,43	0,00	1,38	0,00
986	K1C18,ACTB,NUP43	0,97	0,04	0,95	0,18	0,72	0,00	0,98	0,02	0,93	0,00	0,75	0,00	0,98	0,43	0,86	0,00	0,75	0,00	1,04	0,01	0,98	0,07	1,00	0,93	0,88	0,02	1,07	0,04	1,01	0,55
987		0,70	0,02	0,64	0,05	0,45	0,00	0,74	0,21	0,96	0,86	0,81	0,36	0,63	0,00	0,90	0,19	0,71	0,04	0,69	0,05	0,54	0,01	0,98	0,93	0,85	0,51	1,17	0,46	0,95	0,81
989	KAP0,TXND5	1,07	0,01	1,23	0,01	1,41	0,03	1,00	0,95	0,91	0,00	0,83	0,00	0,92	0,03	1,02	0,60	0,79	0,00	0,95	0,13	0,92	0,01	0,76	0,00	0,88	0,05	0,60	0,01	0,60	0,01
990	IF4A1,HNRPF,ACTG	0,84	0,00	0,84	0,00	0,74	0,00	0,92	0,00	0,78	0,00	0,70	0,00	0,93	0,00	0,96	0,14	0,70	0,00	1,05	0,00	0,98	0,16	0,90	0,01	1,02	0,56	0,92	0,01	0,84	0,00
992	TXND5,ACL6A	0,60	0,00	0,93	0,04	0,59	0,00	0,75	0,00	0,82	0,00	0,52	0,00	0,83	0,02	0,96	0,55	0,66	0,00	1,18	0,00	0,98	0,67	0,83	0,00	0,73	0,00	0,83	0,00	0,80	0,00
993	GDIB,SAHH,IDH3A	0,88	0,00	0,85	0,01	0,75	0,00	0,88	0,00	0,87	0,00	0,75	0,00	0,77	0,00	0,95	0,00	0,81	0,00	0,93	0,00	0,84	0,00	0,95	0,32	1,07	0,19	0,93	0,30	1,02	0,71
996		1,20	0,10	0,92	0,59	0,83	0,33	1,25	0,29	0,56	0,00	0,45	0,00	1,16	0,51	0,89	0,38	0,74	0,01	1,52	0,08	0,58	0,01	0,88	0,45	0,58	0,02	0,80	0,26	0,53	0,02
997	PRS7	0,82	0,00	0,86	0,00	0,64	0,00	0,83	0,00	0,88	0,00	0,68	0,00	0,78	0,00	0,86	0,00	0,72	0,00	0,88	0,00	0,81	0,00	0,89	0,01	0,86	0,00	0,93	0,04	0,97	0,22
998	TBG1,MPPB	0,71	0,00	0,73	0,00	0,59	0,00	0,78	0,00	0,74	0,00	0,60	0,00	0,70	0,00	0,89	0,00	0,60	0,00	1,04	0,18	0,95	0,12	0,96	0,45	1,17	0,01	0,96	0,55	0,98	0,73
1000		0,78	0,00	0,60	0,00	0,40	0,00	0,68	0,00	0,66	0,00	0,42	0,00	0,81	0,01	0,80	0,01	0,79	0,01	0,85	0,05	0,61	0,00	1,06	0,21	0,77	0,00	1,01	0,61	1,14	0,04
1001	ENOG	0,79	0,00	0,68	0,00	0,55	0,00	0,85	0,00	0,66	0,00	0,64	0,00	0,82	0,00	0,83	0,00	0,63	0,00	1,15	0,00	1,15	0,00	1,04	0,19	1,36	0,00	1,22	0,00	1,25	0,00
1002	PRS6A	0,86	0,00	0,90	0,01	0,78	0,00	0,90	0,00	0,81	0,00	0,71	0,00	0,83	0,00	0,90	0,00	0,74	0,00	0,95	0,03	0,85	0,00	0,82	0,00	0,88	0,01	0,83	0,00	0,83	0,00
1005	RINI	0,66	0,00	0,68	0,00	0,38	0,00	0,69	0,00	0,67	0,00	0,50	0,00	0,69	0,00	0,78	0,00	0,69	0,00	1,04	0,15	0,89	0,00	1,00	0,88	1,00	0,84	1,30	0,00	1,54	0,00
1006		0,52	0,03	0,49	0,04	0,23	0,01	0,65	0,21	0,54	0,11	0,32	0,03	0,90	0,39	1,05	0,07	0,59	0,01	0,96	0,83	0,59	0,03	0,86	0,60	0,75	0,34	1,07	0,78	0,86	0,67
1007		0,86	0,06	0,58	0,00	0,42	0,00	0,62	0,06	0,58	0,04	0,40	0,01	0,73	0,00	0,51	0,00	0,75	0,01	0,51	0,00	0,40	0,00	0,71	0,08	0,43	0,01	0,67	0,06	0,85	0,35
1008	ACTG,ACTB	0,93	0,00	0,98	0,31	0,76	0,00	0,94	0,02	0,95	0,04	0,77	0,00	0,65	0,00	0,91	0,00	0,63	0,00	0,85	0,00	0,77	0,00	0,81	0,00	1,02	0,44	0,85	0,00	0,90	0,01
1009		0,68	0,12	0,87	0,01	0,69	0,00	0,79	0,00	0,77	0,00	0,71	0,00	0,69	0,00	0,78	0,00	0,62	0,00	1,10	0,71	0,99	0,96	0,85	0,03	0,88	0,05	0,99	0,91	0,88	0,25
1013	UQCR1	1,01	0,72	1,07	0,01	0,67	0,00	0,91	0,01	0,83	0,00	0,73	0,00	0,90	0,00	1,07	0,00	0,88	0,00	0,87	0,00	0,79	0,00	0,74	0,00	0,88	0,00	1,03	0,20	1,15	0,00
1015	ENOA	1,92	0,00	2,91	0,00	3,48	0,00	1,66	0,00	2,54	0,00	3,38	0,00	1,86	0,00	2,29	0,00	2,52	0,00	0,80	0,00	0,90	0,00	0,80	0,00	0,73	0,00	0,90	0,00	0,67	0,00
1017		0,93	0,47	1,04	0,76	1,11	0,58	0,75	0,02	0,94	0,44	0,83	0,04	0,75	0,00	0,85	0,00	0,73	0,00	0,78	0,03	0,89	0,12	0,88	0,35	0,90	0,41	0,73	0,13	0,72	0,13
1019	IF4A1,ACTG,HNRPF,NUDC	1,07	0,00	1,03	0,21	0,86	0,00	1,02	0,09	1,03	0,02	0,80	0,00	1,03	0,24	1,04	0,15	0,94	0,04	0,82	0,00	0,67	0,00	0,86	0,00	0,71	0,00	0,81	0,00	0,77	0,00
1022	M6PBP,IF35,HNRPF	0,95	0,00	0,94	0,07	0,74	0,00	0,89	0,00	0,89	0,00	0,70	0,00	0,85	0,00	0,93	0,00	0,76	0,00	0,90	0,00	0,83	0,00	0,90	0,02	0,92	0,04	0,90	0,00	0,95	0,01
1024	CALU	1,00	0,93	1,17	0,00	1,30	0,00	1,05	0,01	0,91	0,00	0,84	0,00	0,75	0,00	0,95	0,00	0,76	0,00	1,10	0,00	0,99	0,28	0,81	0,00	1,07	0,02	0,68	0,00	0,76	0,00
1025		0,91	0,03	0,71	0,00	0,66	0,00	0,86	0,03	0,78	0,01	0,64	0,00	0,92	0,43	0,83	0,12	0,95	0,54	0,92	0,02	0,59	0,00	1,07	0,27	0,68	0,00	0,94	0,17	0,84	0,02
1026		0,45	0,01	0,62	0,09	0,37	0,02	0,49	0,00	0,61	0,01	0,32	0,00	0,30	0,00	0,38	0,00	0,24	0,00	1,08	0,57	0,60	0,07	0,97	0,90	0,55	0,06	0,85	0,48	0,59	0,08
1027	CALU,ACTG	1,09	0,00	1,30	0,00	1,47	0,00	1,01	0,62	0,89	0,00	0,84	0,00	0,69	0,00	0,83	0,00	0,68	0,00	1,07	0,02	1,01	0,61	0,79	0,00	1,02	0,43	0,66	0,00	0,73	0,00
1028		1,13	0,15	1,48	0,02	1,48	0,02	0,67	0,25	0,95	0,83	1,08	0,76	1,09	0,27	1,77	0,00	1,10	0,60	0,93	0,60	0,74	0,00	1,00	0,99	0,92	0,50	1,14	0,33	0,58	0,02
1030	PP1R7,IF4A1	0,78	0,00	0,70	0,00	0,53	0,00	0,81	0,00	0,66	0,00	0,56	0,00	0,76	0,00	0,74	0,00	0,58	0,00	1,14	0,01	1,04	0,27	1,02	0,46	1,12	0,00	1,15	0,01	1,16	0,01
1031	VAT1	0,71	0,00	0,52	0,10	0,82	0,79	0,77	0,00	0,78	0,00	0,65	0,00	0,85	0,00	0,88	0,00	0,75	0,00	0,99	0,63	1,02	0,28	1,36	0,47	1,43	0,39	0,73	0,75	0,78	0,80
1032	PA2G4,SAHH,ILEU	0,66	0,00	0,32	0,01	0,00	0,00	0,68	0,00	0,75	0,00	0,54	0,00	0,69	0,00	0,75	0,00	0,67	0,00	1,01	0,46	0,95	0,00	2,29	0,07	2,15	0,09	2,240,386	0,00	2,605,427	0,00
1033	TXND4,ACTG,PRS6B	0,95	0,10	1,08	0,05	1,09	0,02	0,94	0,00	0,83	0,00	0,74	0,00	0,88	0,00	1,02	0,20	0,78	0,00	1,06	0,01	0,96	0,05	0,83	0,00	0,99	0,58	0,73	0,00	0,75	0,00
1034	ACTG,K1C18	1,06	0,01	1,08	0,06	0,87	0,00	1,04	0,00	0,89	0,00	0,85	0,00	0,83	0,00	0,94	0,02	0,68	0,00	0,96	0,00	0,89	0,00	0,81	0,00	0,99	0,78	0,96	0,16	0,89	0,00
1035	ACTG,K1C18,CHD9	0,88	0,00	0,87	0,12	0,67	0,00	0,98	0,20	0,86	0,00	0,72	0,00	0,86	0,00	0,90	0,01	0,69	0,00	0,98	0,01	0,88	0,00	0,86	0,12	0,92	0,36	0,95	0,25	0,92	0,09
1036		0,94	0,24	1,07	0,23	1,06	0,46	0,73	0,01	0,73	0,00	0,61	0,00	0,80	0,00	0,80	0,00	0,67	0,00	0,84	0,03	0,88	0,03	0,75	0,00	0,78	0,00	0,62	0,00	0,65	0,00
1037	CALU,ACTG	1,02	0,28	1,22	0,00	1,39	0,00	1,14	0,00	0,97	0,16	0,91	0,00	0,95	0,00	1,17	0,00	0,93	0,00	1,20	0,00	1,01	0,54	0,85	0,00	1,04	0,05	0,70	0,00	0,73	0,00
1041	MP2K2	0,75	0,00	0,70	0,37	0,20	0,00	0,78	0,00	0,79	0,00	0,65	0,00	0,80	0,00	0,81	0,00	0,69	0,00	1,10	0,00	1,09	0,00	1,18	0,69	1,19	0,68	3,37	0,03	3,51	0,02
1044	VIME	1,69	0,00	1,43	0,01	1,21	0,00	1,69	0,00	1,38	0,00	1,33	0,00	1,71	0,00	2,09	0,00	1,55	0,00	0,93	0,01	0,58	0,00	0,90	0,19	0,84	0,06	1,03	0,37	0,74	0,00
1045		0,82	0,02	0,90	0,60	1,06	0,91	0,89	0,51	2,48	0,16	1,78	0,43	0,11	0,00	0,79	0,29	0,33	0,04	0,82	0,29	0,36	0,00	2,08	0,23	2,49	0,00	1,27	0,78	0,88	0,88
1046	ARP3,TADBP	0,81	0,10	1,00	0,99	0,80	0,00	0,85	0,00	0,77	0,00	0,69	0,00	0,64	0,00	0,73	0,00	0,57	0,00	1,08	0,50	1,01	0,93	0,79	0,00	0,92	0,00	0,89	0,01	0,90	0,01
1047	ADRM1,ACTG,TMOD3,ATPB	0,78	0,00	0,95	0,37	0,66	0,00	0,83	0,02	0,85	0,05	0,64	0,00	0,81	0,00	0,85	0,00	0,76	0,00	0,96	0,26	0,83	0,00	0,80	0,01	0,71	0,00	0,87	0,02	0,91	0,06
1048		0,90	0,39	0,64	0,11	0,31	0,02	0,53	0,20	2,64	0,04	1,44	0,27	0,21	0,00	0,35	0,01	0,41	0,02	0,20	0,00	0,23	0,00	1,37	0,41	0,53	0,17	1,54	0,44	1,30	0,64
1052	SET,ADRM1,MYL3	0,81	0,00	0,87	0,01	0,55	0,00	0,83	0,00	0,70	0,00	0,63	0,00	0,74	0,00	0,87	0,01	0,70	0,00	1,08	0,01	0,90	0,00	0,85	0,02	0,98	0,60	1,21	0,02	1,25	0,01
1053	ACTG,M6PBP	1,10	0,00	1,09	0,16	0,87	0,00	0,98	0,11	0,83	0,00	0,82	0,00	0,79	0,00	1,03	0,16	0,77	0,00	0,93	0,00	0,82	0,00	0,78	0,01	1,07	0,24	0,97	0,17	0,99	0,63
1054		0,96	0,01	1,02	0,40	0,86	0,00	0,99	0,18	0,90	0,00	0,77	0,00	0,84	0,00	0,97	0,32	0,78	0,00	0,91	0,00	0,80	0,00	0,78	0,00	0,87	0,01	0,80	0,00	0,84	0,00
1055	ACTG,IF34	0,83	0,11	0,99	0,94	0,99	0,96	1,03	0,70	1,30	0,01	1,31	0,08	1,33	0,00	1,13	0,00	1,28	0,01	0,79	0,11	0,87	0,27	0,84	0,33	0,62	0,05	0,84	0,48	0,70	0,17
1056		1,00	0,94	1,11	0,02	0,80	0,01	1,10	0,09	1,23	0,00	1,06	0,18	0,98	0,79	0,95	0,63	0,94	0,40	0,93	0,21	0,88	0,06	0,94	0,09	0,77	0,02	1,12	0,09	1,06	0,29
1057		0,70	0,03	0,91	0,04	0,72	0,00	0,91	0,07	1,22	0,09	1,63	0,14	0,43	0,00	0,99	0,95	0,48	0,00	1,17	0,29	0,86	0,40	1,21	0,10	1,55	0,00	2,04	0,11	0,95	0,49
1058	SUCB1,IF34,ACTB,ACTG	0,51	0,00	0,62	0,00	0,46	0,00	0,69	0,00	0,57	0,00	0,47	0,00	0,74	0,00	0,84	0,00	0,55	0,00	1,23	0,01	1,21	0,02	0,84	0,05	1,14	0,06	0,93	0,54	1,00	0,97
1059		0,63	0,13	1,40	0,12	1,12	0,66	0,59	0,03	0,58	0,05	0,44	0,01	0,38	0,00	0,73	0,01	0,32	0,00	1,30	0,17	0,88	0,29	0,58	0,02	0,76	0,03	0,54	0,05	0,42	0,00
1060	PPME1	0,80	0,00	0,80	0,00	0,57	0,00	0,72	0,00	0,75	0,00	0,59	0,00	0,80	0,00	0,81	0,00	0,67	0,00	0,81	0,00	0,87	0,00	0,85	0,00	0,88	0,00	0,94	0,07	1,01	0,59
1061	RCN1,ACTG	0,99	0,43	1,13	0,00	1,22	0,00	0,99	0,58	0,79	0,00	0,80	0,00	0,90	0,00	1,05	0,00	0,81	0,00	1,08	0,00	0,96	0,01	0,76	0,00	0,98	0,35	0,71	0,00	0,70	0,00
1062		0,72	0,01	0,60	0,00	0,42	0,00	0,82	0,01	0,58	0,00	0,59	0,00	0,74	0,00	0,71	0,00	0,45	0,00	1,07	0,53	1,15	0,16	0,89	0,22	1,33	0,01	1,30	0,09	1,21	0,18
1064	ACTG,ACTB,K1C18	0,95	0,50	0,72	0,01	0,80	0,01	0,78	0,00	0,87	0,00	0,73	0,00	1,28	0,00	0,79	0,00	1,23	0,00	1,22	0,02	1,31	0,00	1,81	0,00	1,06	0,53	1,35	0,00	1,48	0,00
1067	ACTG	0,95	0,63	0,51	0,01	0,47	0,00	0,76	0,00	0,84	0,00	0,72	0,00	1,36	0,00	0,82	0,04	1,30	0,00	1,21	0,03	1,37	0,00	2,48	0,00	1,54	0,08	2,30	0,00	2,62	0,00

1070	ACTB	0,91	0,00	0,95	0,51	0,75	0,02	0,89	0,00	0,99	0,37	0,96	0,23	0,81	0,00	0,98	0,31	0,93	0,00	0,81	0,00	0,74	0,00	0,85	0,08	0,85	0,08	1,05	0,61	1,03	0,80
1073	ACTG,ACTB	1,00	0,93	1,28	0,03	1,04	0,44	0,92	0,00	0,91	0,01	0,84	0,00	0,60	0,00	0,94	0,41	0,64	0,00	0,78	0,00	0,69	0,00	0,60	0,00	0,85	0,10	0,68	0,00	0,71	0,00
1074	ACTG	1,15	0,00	1,10	0,06	0,92	0,00	1,04	0,02	0,81	0,00	0,83	0,00	0,86	0,00	1,09	0,01	0,86	0,00	0,92	0,00	0,81	0,00	0,74	0,00	1,07	0,17	0,91	0,00	1,00	0,81
1077	AHSA1,ACTG,CSN4	0,88	0,00	0,95	0,14	0,76	0,00	0,92	0,00	0,90	0,00	0,76	0,00	0,82	0,00	0,95	0,02	0,82	0,00	0,86	0,00	0,75	0,00	0,78	0,00	0,80	0,00	0,82	0,00	0,86	0,01
1078	EIF3G,ARP3,TADBP,ACTB	0,69	0,00	0,77	0,00	0,57	0,00	0,84	0,01	0,78	0,00	0,64	0,00	0,69	0,00	0,80	0,00	0,76	0,00	1,06	0,56	0,83	0,08	0,88	0,02	0,86	0,02	0,97	0,47	1,11	0,15
1080	ACTG,K1C18,ACTB	0,77	0,00	0,78	0,00	0,62	0,00	0,81	0,00	0,73	0,00	0,59	0,00	0,70	0,00	0,83	0,00	0,65	0,00	0,95	0,00	0,83	0,00	0,84	0,00	0,97	0,29	0,86	0,00	0,96	0,09
1081		0,72	0,01	0,70	0,01	0,48	0,00	0,74	0,00	0,73	0,00	0,65	0,00	0,51	0,00	0,80	0,02	0,51	0,00	0,89	0,18	0,85	0,10	0,90	0,35	1,36	0,01	1,19	0,30	1,29	0,10
1082		0,97	0,55	1,49	0,02	1,25	0,05	0,92	0,16	1,02	0,74	0,96	0,42	0,52	0,03	1,06	0,80	0,63	0,07	0,71	0,00	0,66	0,00	0,51	0,00	0,86	0,40	0,57	0,00	0,61	0,00
1083	SPS1	0,70	0,02	0,85	0,00	0,60	0,00	0,83	0,00	0,77	0,00	0,68	0,00	0,77	0,00	0,77	0,00	0,64	0,00	1,08	0,60	1,13	0,40	0,82	0,00	0,93	0,04	1,02	0,74	1,08	0,07
1084	TADBP,PPME1	0,69	0,00	0,72	0,00	0,47	0,00	0,64	0,00	0,70	0,00	0,51	0,00	0,56	0,00	0,71	0,00	0,65	0,00	1,02	0,43	0,92	0,00	1,08	0,02	1,13	0,00	1,19	0,00	1,57	0,00
1085		0,08	0,26	0,08	0,25	0,01	0,30	0,07	0,27	0,14	0,31	0,00	0,24	0,01	0,35	0,08	0,38	0,00	0,34	2,96	0,33	0,43	0,57	6,04	0,11	4,36	0,44	0,00	0,14	1,09	0,94
1086	K1C18,ACTB,NUP43	0,79	0,00	0,85	0,00	0,68	0,00	0,84	0,00	0,83	0,00	0,69	0,00	0,73	0,00	0,89	0,00	0,71	0,00	1,00	0,93	0,90	0,00	0,91	0,04	1,03	0,48	0,96	0,22	1,03	0,43
1087	GALK1,ILEU,DNJBB,ROAA,SAHH	1,02	0,29	0,60	0,20	0,22	0,00	1,06	0,01	1,02	0,23	0,92	0,00	0,94	0,00	1,01	0,18	0,84	0,00	0,98	0,32	0,91	0,00	1,61	0,24	1,68	0,19	4,04	0,01	3,86	0,01
1093	SCMC1,ILEU	0,70	0,00	0,38	0,00	0,06	0,00	0,79	0,00	0,65	0,00	0,63	0,00	0,86	0,00	0,83	0,00	0,63	0,00	1,16	0,00	1,25	0,00	1,74	0,07	2,18	0,01	10,97	0,00	10,76	0,00
1094		0,70	0,00	1,44	0,03	1,27	0,33	0,55	0,06	0,71	0,10	0,57	0,03	0,25	0,15	0,71	0,61	0,28	0,17	0,72	0,17	0,95	0,68	0,45	0,00	1,31	0,57	0,41	0,03	0,58	0,10
1096		0,77	0,01	0,59	0,08	0,65	0,10	0,83	0,01	0,82	0,01	0,78	0,00	0,61	0,00	0,80	0,00	0,55	0,00	0,86	0,05	0,75	0,01	1,12	0,73	1,30	0,41	0,95	0,87	0,79	0,45
1099	CSN4,MPI,BPNT1	0,97	0,17	0,99	0,60	0,94	0,10	0,92	0,02	0,86	0,00	0,74	0,00	0,90	0,00	0,91	0,00	0,82	0,00	0,94	0,03	0,85	0,00	0,86	0,00	0,84	0,00	0,78	0,00	0,80	0,00
1100		0,66	0,01	0,51	0,01	0,37	0,01	0,92	0,67	0,86	0,43	0,56	0,03	0,50	0,00	0,81	0,01	0,62	0,02	1,32	0,02	0,72	0,00	1,60	0,03	1,50	0,04	1,43	0,20	1,57	0,25
1105		0,72	0,00	0,67	0,00	0,45	0,00	0,81	0,00	0,66	0,00	0,61	0,00	0,73	0,00	0,74	0,00	0,62	0,00	1,24	0,00	1,19	0,00	1,08	0,15	1,27	0,00	1,49	0,00	1,61	0,00
1106	CAPG	0,79	0,00	0,79	0,00	0,70	0,00	0,87	0,00	0,74	0,00	0,72	0,00	0,85	0,00	0,96	0,00	0,73	0,00	1,11	0,00	1,16	0,00	0,94	0,06	1,30	0,00	1,04	0,21	1,14	0,00
1107		1,03	0,82	0,87	0,41	0,81	0,35	1,43	0,00	1,08	0,24	1,23	0,02	1,69	0,07	1,81	0,05	1,39	0,23	1,14	0,04	1,04	0,59	1,01	0,93	1,32	0,13	1,24	0,27	1,09	0,64
1114	ACTB	1,31	0,00	1,70	0,00	1,96	0,00	1,29	0,00	1,55	0,00	1,71	0,00	1,35	0,00	1,53	0,00	1,67	0,00	0,84	0,00	0,81	0,00	0,78	0,02	0,70	0,00	0,74	0,03	0,67	0,01
1115	THOC3	0,71	0,10	0,88	0,12	0,60	0,00	0,96	0,69	0,97	0,69	0,79	0,05	0,72	0,00	0,85	0,00	0,66	0,00	1,23	0,31	1,13	0,55	1,00	1,00	1,07	0,37	1,20	0,00	1,22	0,00
1116	UBCP1	0,86	0,00	0,54	0,07	0,34	0,01	0,94	0,04	0,80	0,00	0,78	0,00	1,00	0,97	0,97	0,01	0,77	0,00	1,10	0,03	1,15	0,01	1,50	0,24	1,77	0,09	2,32	0,05	2,24	0,06
1119	STML2,ACTG	1,51	0,00	1,56	0,00	2,05	0,00	1,33	0,00	1,12	0,00	1,19	0,00	1,18	0,00	1,21	0,00	1,06	0,00	0,86	0,00	0,79	0,00	0,71	0,00	0,78	0,00	0,57	0,00	0,52	0,00
1121	SPSY,ARSA1	0,75	0,00	0,73	0,00	0,53	0,00	0,77	0,00	0,76	0,00	0,58	0,00	0,75	0,00	0,85	0,00	0,69	0,00	1,01	0,41	0,92	0,00	1,02	0,39	1,07	0,02	1,07	0,15	1,20	0,00
1124		1,26	0,26	1,11	0,46	0,62	0,03	0,95	0,63	1,06	0,24	0,79	0,07	0,84	0,00	1,17	0,02	0,88	0,01	0,59	0,05	0,59	0,04	0,74	0,05	0,94	0,58	0,99	0,96	1,26	0,12
1125		0,63	0,00	0,70	0,01	0,53	0,00	0,82	0,06	0,67	0,01	0,74	0,03	0,78	0,02	1,02	0,78	0,75	0,01	1,14	0,30	1,16	0,27	0,84	0,22	1,37	0,03	1,23	0,10	1,31	0,01
1127	RCN1,SPRC	0,96	0,02	1,19	0,00	1,22	0,00	0,95	0,00	0,90	0,00	0,74	0,00	0,70	0,00	0,78	0,00	0,61	0,00	1,05	0,01	0,90	0,00	0,79	0,00	0,81	0,00	0,64	0,00	0,62	0,00
1128	EI2BL,CAPG,ROAA	0,71	0,00	0,70	0,00	0,53	0,00	0,81	0,00	0,67	0,00	0,64	0,00	0,84	0,00	0,92	0,00	0,70	0,00	1,15	0,00	1,25	0,00	0,97	0,14	1,39	0,00	1,21	0,00	1,39	0,00
1129		0,84	0,00	0,88	0,02	0,63	0,00	0,90	0,03	0,94	0,04	0,74	0,00	0,66	0,00	0,81	0,00	0,65	0,00	0,86	0,00	0,73	0,00	0,85	0,01	0,86	0,02	0,93	0,23	0,96	0,56
1130		0,13	0,03	0,34	0,08	0,14	0,07	0,51	0,31	0,28	0,11	0,21	0,09	1,23	0,28	0,93	0,55	1,23	0,20	5,44	0,09	3,42	0,01	1,12	0,57	0,97	0,89	2,05	0,15	3,05	0,01
1131	TMOD3	0,89	0,00	0,90	0,01	0,78	0,00	0,92	0,00	0,77	0,00	0,70	0,00	0,81	0,00	0,92	0,00	0,70	0,00	1,00	0,82	0,88	0,00	0,82	0,00	1,00	0,96	0,87	0,01	0,87	0,01
1135		0,87	0,37	1,09	0,64	0,65	0,05	0,93	0,35	0,95	0,66	0,69	0,00	0,67	0,13	0,77	0,29	0,67	0,12	1,07	0,52	0,90	0,41	0,87	0,43	0,84	0,33	1,06	0,46	1,22	0,07
1136		1,14	0,04	1,02	0,74	0,81	0,02	0,97	0,41	0,82	0,00	0,81	0,00	0,78	0,01	0,97	0,73	0,80	0,02	0,88	0,01	0,79	0,00	0,84	0,00	1,10	0,31	1,04	0,31	1,15	0,00
1137	GALK1,ILEU,DNJBB,ROAA	0,70	0,00	0,28	0,01	0,84	0,81	0,82	0,00	0,73	0,00	0,61	0,00	0,70	0,00	0,78	0,00	0,58	0,00	1,13	0,00	1,10	0,01	2,46	0,06	3,02	0,02	0,70	0,72	0,75	0,76
1138		0,60	0,00	0,65	0,00	0,38	0,00	0,71	0,00	0,79	0,00	0,54	0,00	0,90	0,17	0,89	0,01	0,82	0,01	1,06	0,37	1,20	0,10	1,09	0,17	1,09	0,20	1,27	0,20	1,71	0,02
1139	ILEU,SCMC1,ROAA	0,80	0,00	0,42	0,01	0,15	0,00	0,90	0,00	0,78	0,00	0,79	0,00	0,75	0,00	0,84	0,01	0,65	0,00	1,04	0,24	1,05	0,18	1,70	0,08	2,23	0,01	5,03	0,00	5,04	0,00
1142	SET	1,12	0,06	0,96	0,47	0,68	0,00	1,09	0,01	0,88	0,00	0,86	0,00	0,78	0,00	0,89	0,03	0,69	0,00	0,85	0,01	0,73	0,00	0,81	0,00	0,98	0,62	1,12	0,16	1,08	0,29
1143	SPB6	0,85	0,00	0,90	0,02	1,17	0,00	0,92	0,00	0,81	0,00	0,78	0,00	0,87	0,00	0,93	0,00	0,78	0,00	1,12	0,00	1,13	0,00	0,93	0,06	1,13	0,01	0,70	0,00	0,74	0,00
1145	EI2BL	0,59	0,00	0,64	0,00	0,47	0,00	0,76	0,00	0,62	0,00	0,59	0,00	0,63	0,00	0,76	0,00	0,50	0,00	1,27	0,00	1,38	0,00	0,94	0,03	1,53	0,00	1,21	0,00	1,37	0,00
1146	RFC2	0,77	0,01	0,36	0,02	0,00	0,00	0,93	0,40	0,79	0,00	0,75	0,00	0,77	0,01	0,82	0,00	0,60	0,00	1,18	0,11	1,07	0,43	2,12	0,07	2,42	0,03	2,783,752	0,00	2,465,027	0,00
1151		0,95	0,10	1,28	0,00	0,95	0,26	1,00	0,95	0,85	0,00	0,86	0,00	0,79	0,00	0,90	0,01	0,71	0,00	1,01	0,55	0,87	0,00	0,65	0,00	0,74	0,00	0,88	0,02	0,79	0,00
1152	ARSA1	0,86	0,00	0,78	0,00	0,66	0,00	0,86	0,00	0,71	0,00	0,70	0,00	0,78	0,00	0,84	0,00	0,69	0,00	1,09	0,01	0,98	0,19	0,98	0,58	1,17	0,00	1,14	0,02	1,11	0,02
1153	TRA2B,CSN4	0,87	0,00	0,89	0,00	0,67	0,00	0,85	0,00	0,77	0,00	0,71	0,00	0,79	0,00	0,92	0,00	0,73	0,00	0,94	0,05	0,87	0,00	0,83	0,00	0,98	0,55	1,03	0,46	1,04	0,30
1154	CNN3,IDH3A	0,87	0,00	0,79	0,00	0,52	0,00	1,06	0,00	0,89	0,00	0,78	0,00	0,93	0,00	1,04	0,04	0,76	0,00	1,02	0,45	0,93	0,05	0,95	0,03	1,15	0,00	1,24	0,00	1,26	0,00
1156	RSSA,SET	0,92	0,00	1,19	0,05	0,89	0,01	0,91	0,00	0,79	0,00	0,73	0,00	0,69	0,00	0,77	0,00	0,59	0,00	1,00	0,85	0,88	0,00	0,67	0,00	0,75	0,01	0,83	0,00	0,78	0,00
1157	SET	0,83	0,00	0,89	0,00	0,61	0,00	0,90	0,00	0,78	0,00	0,61	0,00	0,67	0,00	0,84	0,00	0,61	0,00	1,01	0,52	0,72	0,00	0,81	0,00	0,85	0,00	0,93	0,02	0,89	0,00
1158		0,82	0,00	0,83	0,00	0,59	0,00	0,96	0,08	0,91	0,03	0,76	0,00	0,92	0,00	1,02	0,61	0,94	0,16	0,97	0,39	0,80	0,00	0,89	0,02	0,86	0,00	1,06	0,16	1,12	0,09
1161	STRAP	0,77	0,00	0,88	0,00	0,54	0,00	0,82	0,00	0,91	0,01	0,65	0,00	0,76	0,00	0,89	0,00	0,77	0,00	0,92	0,01	0,78	0,00	0,89	0,00	0,79	0,00	1,03	0,21	1,13	0,00
1164		0,83	0,00	1,11	0,06	1,00	0,95	0,90	0,00	0,74	0,00	0,67	0,00	0,71	0,00	0,81	0,00	0,66	0,00	1,10	0,00	0,90	0,00	0,68	0,00	0,77	0,00	0,68	0,00	0,69	0,00
1165	CNN3	0,87	0,00	0,79	0,00	0,64	0,00	0,83	0,00	0,76	0,00	0,63	0,00	0,80	0,00	0,93	0,02	0,81	0,00	0,88	0,00	0,74	0,00	0,90	0,08	0,95	0,35	0,92	0,09	1,02	0,64
1166	GIPC1	0,91	0,02	0,51	0,10	0,30	0,04	1,01	0,88	0,95	0,04	0,86	0,00	1,00	0,94	1,04	0,06	0,95	0,10	0,98	0,59	0,98	0,47	1,66	0,23	1,82	0,15	2,59	0,12	2,88	0,08
1167	GLRX3,TXNL2,SNAG,CPIN1	0,82	0,00	0,77	0,00	0,61	0,00	0,92	0,00	0,80	0,00	0,70	0,00	0,82	0,00	0,92	0,00	0,75	0,00	1,01	0,55	0,91	0,00	0,93	0,01	1,08	0,01	1,03	0,11	1,12	0,00
1169	DJB11,ROAA	0,82	0,00	0,94	0,20	0,78	0,02	0,90	0,01	0,85	0,00	0,73	0,00	0,85	0,00	0,95	0,01	0,82	0,00	1,00	0,88	0,91	0,01	0,83	0,01	0,88	0,04	0,86	0,13	0,91	0,38
1173	FSTL1,ANXA2	0,80	0,00	0,90	0,08	0,93	0,48	0,72	0,00	0,72	0,00	0,64	0,00	0,59	0,00	0,73	0,00	0,63	0,00	0,84	0,00	0,70	0,00	0,75	0,00	0,78	0,01	0,64	0,01	0,65	0,01
1174	IDH3A	0,99	0,84	0,87	0,00	0,61	0,00	1,10	0,01	0,95	0,08	0,92	0,01	0,88	0,01	1,07	0,07	0,76	0,00	0,88	0,02	0,84	0,01	0,88	0,00	1,16	0,00	1,21	0,00	1,18	0,03
1175		0,88	0,00	0,76	0,00	0,49	0,00	0,92	0,00	0,83	0,00	0,75	0,00	0,84	0,00	0,96	0,16	0,75	0,00	0,96	0,04	0,93	0,01	1,00	1,00	1,21	0,02	1,40	0,02	1,47	0,01
1178		1,27	0,02	1,48	0,15	1,06	0,40	1,06	0,51	2,12	0,00	2,76	0,00	1,35	0,10	2,13	0,00	1,48	0,35	0,72	0,00	0,80	0,03	1,25	0,25	1,08	0,68	2,27	0,00	1,05	0,89
1181		0,95	0,07	0,99	0,85	0,81	0,00	0,97	0,27	0,90	0,01	0,84	0,00	0,84	0,00	1,02	0,16	0,90	0,00	0,95	0,01	0,85	0,00	0,84	0,00	0,98	0,52	0,97	0,37	1,07	0,09
1182		0,59	0,00	0,61	0,00	0,45	0,00	0,54	0,00	0,47	0,00	0,40	0,00	0,48	0,00	0,60	0,00	0,47	0,00	1,04	0,44	0,91	0,09	0,86	0,07	1,10	0,19	1,00	0,98	1,19	0,04
1183	BIEA,TALDO	0,86	0,00	0,81	0,05	0,67	0,01	0,99	0,08	0,83	0,00	0,75	0,00	0,92	0,00	1,02	0,08	0,82	0,00	1,10	0,00	1,01	0,65	0,98	0,86	1,17	0,10	1,07	0,60	1,14	0,34
1185	IF32,NPM	1,02	0,05	1,08	0,02	0,80	0,00	1,08	0,00	0,99	0,48	0,85	0,00	0,92	0,00	1,07	0,00	0,97	0,07	0,90	0,00	0,73	0,00	0,77	0,00	0,80	0,00	0,90	0,01	0,98	0,45
1186	PP1B,NAGK	0,96	0,15	0,89	0,02	0,73	0,00	1,07	0,01	0,96	0,12	0,83	0,00	0,98	0,43	1,06	0,04	0,95	0,04	0,93	0,01	0,81	0,00	0,90	0,03	0,95	0,21	0,95	0,13	1,04	0,27
1187		1,15	0,00	1,23	0,02	1,03	0,60	1,29	0,00	1,10	0,01	1,10	0,00	1,09	0,11	1,31	0,00	1,21	0,00	0,93	0,02	0,79	0,00	0,74	0,00	0,88	0,09	0,88	0,06	0,98	0,74
1188	DDAH1,EIF3I,NPM	1,06	0,00	1,04	0,27	0,80	0,00	1,10	0,00	0,96	0,11	0,88	0,00	1,01	0,77	1,14	0,00	1,00	0,98	0,93	0,00	0,86	0,00	0,83	0,00	0,98	0,60	0,99	0,72	1,12	0,03
1189		0,91	0,50	1,02	0,69	0,82	0,01	1,11	0,00	0,94	0,01	0,86	0,00	1,03	0,03	1,14	0,00	1,05	0,01	1,07	0,64	0,87	0,39	0,81	0,00	0,86	0,00	0,92	0,06	0,99	0,68
1190		1,07	0,10	1,18	0,14	0,80	0,04	1,21	0,00	1,07	0,00	0,96	0,03	1,08	0,48	1,28	0,08	1,16	0,18	0,90	0,01	0,73	0,00	0,72	0,02	0,79	0,08	0,96	0,65	1,05	0,59
1193		0,47	0,27	0,63	0,51	0,24	0,19	0,93	0,84	1,28	0,52	0,96	0,92	0,80	0,45	1,69	0,04	1,67	0,17	1,40	0,29	0,78	0,56	1,44	0,48	1,24	0,68	2,83	0,03	3,17	0,07
1194	HDGF	0,76	0,00	0,76	0,00	0,55	0,00	0,86	0,00	0,70	0,00	0,67	0,00	0,70	0,00	0,85	0,00	0,59	0,00	1,15	0,00	1,05	0,13	0,94	0,04	1,28	0,00	1,25	0,00	1,24	0,01
1195		0,95	0,64	1,00	0,88	0,80	0,00	1,11	0,00	0,95	0,10	0,85	0,00	1,07	0,02	1,18	0,00	1,02	0,37	1,04	0,74	0,90	0,42	0,84	0,00	0,94	0,02	0,93	0,01	1,01	0,49
1196	IF2A	0,75	0,00	0,83	0,00	0,60	0,00	0,84	0,00	0,86	0,00	0,68	0,00	0,77	0,00	0,84	0,00	0,75	0,00	0,98	0,06	0,87	0,00	0,91	0,01	0,87	0,00	0,99	0,80	1,07	0,09
1197		0,83	0,00	0,91	0,19	0,52	0,00	0,88	0,00	0,91	0,00	0,80	0,00	0,72	0,00	0,86	0,01	0,71	0,00	0,86	0,00	0,83	0,00	0,81	0,02	0,91	0,21	1,25	0,07	1,32	0,03
1200	NPM	1,08	0,01	1,04	0,24	0,87	0,00	1,06	0,37	0,96	0,39	0,85	0,02	1,18	0,04	1,23	0,00	1,12	0,02	0,95	0,31	0,88	0,04	0,89	0,01	0,95	0,14	0,95	0,09	1,03	0,43
1201		0,90	0,40	0,94	0,10	0,78	0,00	1,15	0,00	1,04	0,03	0,89	0,00	0,94	0,01	1,17	0,00	0,97	0,03	1,02	0,90	0,82	0,22	0,89	0,00	0,97	0,32	0,92	0,06	0,97	0,44
1203	HNRPC	0,75	0,00	0,63	0,00	0,71	0,02	0,90	0,03	0,86	0,02	0,73	0,00	0,79	0,00	0,85	0,00	0,66	0,00	0,91	0,15	1,07	0,21	1,03	0,63	1,36	0,00	0,77	0,09	0,94	0,63
1212		1,61	0,09	1,33	0,34	1,34	0,23	2,02	0,00	4,82	0,00	4,14	0,00	1,17	0,60	2,47	0,00	2,36	0,00	0,41	0,01	0,43	0,02	1,20	0,43	1,09	0,69	1,01	0,94	1,03	0,88
1214	SUGT1	0,84	0,00	0,89	0,01	0,73	0,00	0,89	0,00	0,90	0,00	0,71	0,00	0,84	0,00	0,91	0,00	0,81	0,00	0,88	0,00	0,77	0,00	0,84	0,00	0,79	0,00	0,81	0,00	0,86	0,00
1219		0,86	0,02	0,78	0,00	0,70	0,00	0,90	0,10	0,84	0,03	0,73	0,00	1,01	0,94	1,17	0,16	0,82	0,09	0,84	0,00	0,65	0,00	0,87	0,00	0,83	0,01	0,84	0,04	0,65	0,00
1223		0,82	0,61	0,86	0,63	0,89	0,71	0,94	0,71	3,07	0,00	0,65	0,19	0,68	0,20	0,26	0,01	1,10	0,65	0,39	0,08	0,55	0,22	1,21	0,39	0,20	0,01	0,25	0,00	0,83	0,34
1225	NPM,EF1D	1,04	0,33	0,95	0,36	0,86	0,07	1,10	0,19	1,15	0,01	0,96	0,42	1,23	0,10	1,66	0,00	1,59	0,00	0,75	0,00	0,57	0,00	0,85	0,02	0,85	0,03	0,79	0,03	0,90	0,26
1226		0,91	0,07	0,98	0,59	0,91	0,09	0,99	0,87	1,04	0,41	0,89	0,08	1,01	0,84	1,07	0,02	0,90	0,01	0,88	0,02	0,90	0,02	0,86	0,00	0,89	0,00	0,80	0,00	0,80	0,00
1228		1,42	0,05	1,35	0,31	1,06	0,66	1,39	0,02	1,31	0,00	1,21	0,18	1,38	0,08	1,65	0,01	2,03	0,02	0,76	0,08	0,57	0,01	0,75	0,31	0,72	0,27	0,88	0,39	1,13	0,54
1229		0,82	0,02	0,74	0,00	0,57	0,00	0,80	0,01	0,93	0,11	0,73	0,00	0,96	0,54	0,88	0,19	0,93	0,52	0,87	0,17	0,87	0,12	1,11	0,10	0,87	0,18	1,13	0,05	1,20	0,24
1230		1,30	0,00	1,27	0,00	1,03	0,58	1,32	0,00	1,19	0,00	1,24	0,00	0,94	0,47	1,37	0,00	1,13	0,11	0,81	0,00	0,67	0,00	0,75	0,00	0,99	0,72	0,96	0,27	1,01	0,90
1231	HDGF	0,91	0,48	0,66	0,00	0,56	0,00	1,04	0,70	0,83	0,05	0,84	0,06	0,98	0,86	1,00	0,88	0,88	0,01	0,91	0,49	0,83	0,28	1,00	0,95	1,16	0,03	1,18	0,07	1,20	0,04
1233		0,69	0,00	0,90	0,09	0,70	0,00	0,80	0,01	0,98	0,70	0,63	0,00	0,79	0,03	0,86	0,07	0,73	0,00	0,95	0,36	0,81	0,02	0,89	0,09	0,68	0,00	0,73	0,00	0,74	0,00
1236		1,07	0,74	1,26	0,13	1,19	0,38	0,37	0,12	0,46	0,18	0,32	0,10	0,54	0,11	0,89	0,53	3,19	0,35	0,73	0,22	0,42	0,04	0,78	0,19	0,59	0,01	0,58	0,04	2,23	0,52
1241	PP2AB,CT077	0,90	0,00	0,90	0,00	0,73	0,00	1,01	0,16	0,89	0,00	0,80	0,00	0,93	0,01	1,01	0,64	0,87	0,00	1,01	0,70	0,89	0,00	0,89	0,00	0,96	0,02	0,99	0,62	1,02	0,15
1243	PPP6,LDHB,PDXK	0,87	0,02	0,90	0,01	0,75	0,00	0,91	0,01	0,84	0,00	0,70	0,00	0,81	0,00	1,00	0,85	0,77	0,00	1,08	0,16	0,98	0,60	0,95	0,09	1,16	0,00	0,95	0,15	1,07	0,11
1248	RLA0	0,85	0,00	0,91	0,04	0,84	0,01	0,88	0,00	0,85	0,00	0,70	0,00	0,82	0,00	1,03	0,20	0,79	0,00	0,94	0,07	0,87	0,02	0,84	0,00	1,02	0,68	0,75	0,00	0,84	0,02
1251	EF1D	1,08	0,04	1,10	0,02	0,96	0,36	1,14	0,00	1,10	0,00	0,88	0,00	1,21	0,00	1,22	0,00	1,27	0,00	0,74	0,00	0,56	0,00	0,70	0,00	0,56	0,00	0,64	0,00	0,66	0,00
1252	PP2AA,ACTG,ANXA2	0,94	0,02	1,00	0,93	0,78	0,00	1,06	0,04	0,98	0,45	0,88	0,00	0,83	0,00	0,96	0,01	0,86	0,00	0,99	0,68	0,86	0,00	0,86	0,01	0,93	0,08	0,99	0,77	1,06	0,10
1254	NACA,ANXA2	1,46	0,00	1,40	0,00	1,13	0,05	1,32	0,00	1,16	0,00	0,98	0,38	1,17	0,00	1,30	0,00	1,17	0,00	0,80	0,00	0,59	0,00	0,74	0,00	0,69	0,00	0,77	0,00	0,77	0,00
1255	ODPB	0,77	0,00	0,76	0,00	0,67	0,00	0,82	0,00	0,71	0,00	0,64	0,00	0,80	0,00	0,89	0,00	0,66	0,00	1,05	0,01	1,01	0,45	0,93	0,26	1,14	0,06	0,94	0,14	0,95	0,14
1256		0,86	0,00	0,89	0,01	0,65	0,00	0,87	0,00	0,78	0,00	0,69	0,00	0,92	0,03	1,00	0,92	0,80	0,00	0,98	0,23	0,90	0,00	0,84	0,00	0,94	0,05	1,01	0,84	1,03	0,52
1260		0,86	0,02	0,89	0,01	0,69	0,00	0,91	0,05	0,83	0,00	0,70	0,00	0,74	0,00	0,81	0,00	0,70	0,00	1,01	0,93	0,87	0,09	0,89	0,01	0,91	0,01	0,96	0,45	1,01	0,88
1265		0,96	0,53	1,16	0,57	0,77	0,14	1,17	0,07	1,00	0,98	0,98	0,67	0,88	0,03	1,15	0,01	0,95	0,16	1,04	0,63	0,85	0,04	0,74	0,30	0,93	0,76	1,09	0,62	1,15	0,42
1266		1,26	0,07	1,06	0,79	0,65	0,01	1,10	0,61	1,35	0,04	0,92	0,74	1,05	0,74	1,34	0,02	1,23	0,43	0,71	0,06	0,55	0,01	1,03	0,89	0,84	0,43	1,15	0,61	1,25	0,47

1267	SGTA,HDGF,ANXA2	1,00	1,00	0,86	0,01	0,81	0,01	1,07	0,01	0,82	0,00	0,85	0,00	0,96	0,05	1,03	0,13	0,86	0,00	1,00	0,95	0,91	0,18	0,89	0,00	1,14	0,00	0,98	0,75	1,01	0,83
1268	CHM4B,RPB3	1,00	0,77	0,93	0,00	0,82	0,00	1,00	0,50	0,82	0,00	0,79	0,00	1,01	0,61	1,03	0,02	0,83	0,00	0,98	0,07	0,88	0,00	0,87	0,00	0,98	0,23	0,94	0,04	0,89	0,00
1269	TXNL1,ANXA2	0,86	0,00	0,84	0,00	0,75	0,00	0,97	0,25	0,86	0,00	0,78	0,00	0,93	0,01	1,04	0,03	0,88	0,00	0,96	0,12	0,79	0,00	0,87	0,00	0,90	0,01	0,89	0,03	0,86	0,01
1270	TALDO	1,06	0,54	0,66	0,05	0,25	0,00	1,18	0,03	1,17	0,05	1,01	0,94	0,75	0,04	1,09	0,47	0,82	0,16	0,85	0,02	0,60	0,00	1,36	0,10	1,41	0,08	3,04	0,01	2,74	0,02
1272	PP1A	0,88	0,02	0,61	0,08	0,27	0,00	1,04	0,23	0,98	0,48	0,80	0,00	0,72	0,04	0,90	0,51	0,74	0,05	0,85	0,00	0,66	0,00	1,14	0,66	1,17	0,62	2,09	0,01	2,19	0,01
1277	SAE1,EF1D	0,98	0,21	1,01	0,54	0,92	0,02	1,01	0,70	0,81	0,00	0,82	0,00	0,84	0,00	0,91	0,00	0,78	0,00	1,00	1,00	0,87	0,00	0,78	0,00	0,90	0,00	0,86	0,00	0,85	0,00
1278	EF1D	1,07	0,00	1,14	0,00	1,02	0,25	1,03	0,50	0,91	0,04	0,80	0,00	0,91	0,00	0,98	0,05	0,85	0,00	0,92	0,00	0,73	0,00	0,76	0,00	0,73	0,00	0,75	0,00	0,71	0,00
1279	PP2AB	0,66	0,00	0,82	0,01	0,49	0,00	0,73	0,00	0,74	0,00	0,60	0,00	0,76	0,00	0,84	0,00	0,64	0,00	1,15	0,00	1,09	0,10	0,93	0,16	0,98	0,68	1,25	0,07	1,24	0,07
1281		0,87	0,08	0,85	0,08	0,85	0,09	1,07	0,50	0,83	0,00	0,83	0,01	0,69	0,00	0,89	0,06	0,62	0,00	1,04	0,73	0,83	0,09	0,83	0,10	1,09	0,38	0,83	0,14	0,76	0,09
1282	ODPB,CSN6,GBB2,GB B1	0,60	0,00	0,69	0,00	0,41	0,00	0,63	0,00	0,59	0,00	0,48	0,00	0,61	0,00	0,78	0,00	0,50	0,00	1,13	0,00	1,07	0,01	0,92	0,03	1,19	0,00	1,27	0,02	1,30	0,02
1283		0,87	0,03	0,82	0,03	0,71	0,01	0,99	0,87	0,86	0,03	0,76	0,01	0,82	0,00	0,96	0,25	0,73	0,00	1,10	0,25	0,85	0,02	1,01	0,87	1,06	0,43	1,04	0,75	0,93	0,49
1286	ID1736519	0,92	0,00	1,00	0,96	0,85	0,00	0,96	0,36	0,79	0,00	0,76	0,00	0,79	0,00	0,89	0,00	0,66	0,00	0,98	0,60	0,85	0,00	0,74	0,00	0,88	0,00	0,84	0,00	0,76	0,00
1289		1,05	0,00	1,08	0,10	1,02	0,68	0,96	0,23	0,84	0,00	0,80	0,00	0,80	0,00	0,94	0,01	0,69	0,00	0,81	0,00	0,73	0,00	0,70	0,00	0,83	0,00	0,70	0,00	0,65	0,00
1291	IPYR,GBB1,GBB2	0,84	0,00	0,89	0,00	0,72	0,00	0,89	0,00	0,86	0,00	0,70	0,00	0,83	0,00	0,91	0,00	0,80	0,00	0,96	0,02	0,85	0,00	0,88	0,00	0,88	0,00	0,87	0,01	0,95	0,06
1293	ANXA3,IPYR	0,87	0,00	0,99	0,73	0,90	0,00	0,85	0,00	0,87	0,00	0,71	0,00	0,76	0,00	0,88	0,00	0,75	0,00	0,94	0,00	0,86	0,00	0,86	0,00	0,88	0,00	0,78	0,00	0,83	0,00
1295		0,36	0,03	0,62	0,24	0,34	0,06	0,28	0,05	0,94	0,88	0,12	0,02	0,15	0,01	0,29	0,02	0,11	0,01	0,66	0,35	0,45	0,11	1,28	0,59	0,50	0,19	0,29	0,12	0,34	0,17
1296	PPP6,PGP,CAZA2	0,70	0,01	0,84	0,01	0,67	0,00	0,82	0,01	0,76	0,00	0,58	0,00	0,66	0,00	0,89	0,00	0,61	0,00	1,18	0,13	1,01	0,90	0,91	0,03	1,15	0,00	0,87	0,01	0,98	0,70
1298		0,83	0,00	0,56	0,22	0,08	N,A,	0,88	0,00	0,86	0,00	0,70	0,00	0,99	0,77	0,91	0,01	0,75	0,03	1,09	0,00	1,12	0,00	1,56	0,35	1,52	0,38	8,67	N,A,	8,47	N,A,
1299		0,22	0,45	0,01	0,34	0,01	0,41	0,36	0,52	0,03	0,32	0,03	0,32	2,77	0,45	0,59	0,10	0,51	0,25	1,45	0,78	0,45	0,66	2,10	0,22	1,86	0,20	3,69	0,22	2,36	0,52
1300	IPYR	0,98	0,13	1,06	0,04	0,72	0,00	0,97	0,09	0,87	0,00	0,82	0,00	0,73	0,00	0,91	0,00	0,72	0,00	0,96	0,00	0,86	0,00	0,80	0,00	0,98	0,29	1,10	0,04	1,14	0,00
1301		0,17	0,01	0,62	0,22	0,23	0,03	0,23	0,01	0,65	0,31	0,05	0,00	0,02	0,03	0,22	0,06	0,01	0,02	1,70	0,07	0,18	0,01	1,38	0,56	0,50	0,19	0,28	0,27	0,06	0,15
1302		1,04	0,96	0,39	0,23	0,22	0,12	0,36	0,16	37,03	0,28	75,33	0,29	0,33	0,13	0,48	0,06	0,93	0,91	0,24	0,34	0,20	0,40	64,71	0,28	0,75	0,76	236,92	0,38	2,64	0,42
1303		1,03	0,67	0,78	0,03	0,59	0,01	0,81	0,00	0,80	0,00	0,59	0,00	0,88	0,10	0,86	0,04	0,83	0,02	0,65	0,00	0,65	0,00	0,85	0,10	0,83	0,09	0,84	0,31	1,08	0,63
1304	PCNA	0,82	0,00	0,79	0,00	0,68	0,00	0,88	0,00	0,66	0,00	0,65	0,00	0,79	0,00	0,88	0,00	0,58	0,00	1,19	0,00	1,06	0,01	0,93	0,00	1,23	0,00	1,06	0,08	0,93	0,02
1305	SNAA,SFRS1	0,73	0,00	0,76	0,00	0,60	0,00	0,71	0,00	0,66	0,00	0,54	0,00	0,72	0,00	0,72	0,00	0,61	0,00	1,02	0,26	0,92	0,00	0,91	0,00	0,88	0,00	0,95	0,16	0,93	0,08
1308	LZTL1,SFRS1,SPEE	0,73	0,00	0,86	0,00	0,51	0,00	0,81	0,00	0,89	0,00	0,61	0,00	0,69	0,00	0,83	0,00	0,69	0,00	0,86	0,00	0,71	0,00	0,81	0,00	0,73	0,00	0,94	0,11	1,03	0,12
1312	OTUB1	0,81	0,00	0,76	0,00	0,60	0,00	0,81	0,00	0,72	0,00	0,63	0,00	0,83	0,00	0,87	0,00	0,75	0,00	1,06	0,01	0,95	0,04	1,02	0,25	1,07	0,01	1,13	0,02	1,17	0,00
1313		0,00	0,19	0,56	0,51	0,41	0,46	0,39	0,11	0,99	0,99	0,23	0,07	0,56	0,12	0,63	0,01	0,08	0,00	1.000,00	0,01	1.000,00	0,09	0,94	0,92	0,54	0,33	0,29	0,38	0,09	0,25
1315	TPM1	0,76	0,00	0,82	0,00	0,65	0,00	0,59	0,00	0,62	0,00	0,47	0,00	0,49	0,00	0,54	0,00	0,52	0,00	0,85	0,00	0,75	0,00	0,83	0,00	0,76	0,00	0,79	0,00	0,93	0,01
1316	PP4C,EF1D	1,05	0,15	1,14	0,03	0,99	0,90	0,95	0,60	0,84	0,16	0,75	0,05	0,82	0,00	0,95	0,00	0,84	0,00	0,98	0,39	0,77	0,00	0,80	0,00	0,82	0,00	0,82	0,02	0,84	0,01
1318	CLIC4,PPIE	0,83	0,02	0,89	0,08	0,75	0,02	0,96	0,17	0,79	0,00	0,81	0,00	0,87	0,00	0,87	0,00	0,78	0,00	1,10	0,11	1,01	0,80	0,84	0,03	0,95	0,35	1,03	0,80	1,02	0,86
1319	DNJC9	0,61	0,00	0,76	0,00	0,53	0,00	0,83	0,00	0,75	0,00	0,71	0,00	0,82	0,00	0,86	0,00	0,68	0,00	1,17	0,24	1,19	0,20	0,85	0,06	1,01	0,95	1,15	0,30	1,14	0,33
1321	OTUB1	1,07	0,12	0,88	0,04	0,83	0,02	0,99	0,87	0,77	0,04	0,74	0,03	1,05	0,22	1,03	0,38	0,92	0,02	1,03	0,40	0,81	0,00	0,98	0,65	0,97	0,59	1,00	0,99	0,92	0,17
1325	WDR61,ANXA5	0,71	0,00	0,89	0,03	0,70	0,00	0,74	0,00	0,78	0,00	0,64	0,00	0,69	0,00	0,78	0,00	0,61	0,00	0,99	0,41	0,91	0,00	0,84	0,01	0,82	0,01	0,87	0,06	0,82	0,02
1327		0,00	0,12	0,20	0,14	0,03	0,13	0,00	0,23	0,18	0,30	0,00	0,23	0,03	0,08	0,03	0,08	0,00	0,07	1.000,00	0,27	1.000,00	0,27	1,65	0,64	0,06	0,49	0,00	0,46	0,00	0,46
1328	PCNA,CX026,AN32E	1,06	0,26	0,97	0,51	0,97	0,72	1,06	0,21	0,74	0,00	0,83	0,01	0,83	0,00	0,99	0,74	0,69	0,00	1,15	0,01	0,94	0,08	0,89	0,00	1,23	0,00	0,99	0,89	0,86	0,06
1331	PP1A,DHC3	0,77	0,00	0,95	0,25	0,68	0,00	0,85	0,01	0,92	0,01	0,77	0,01	0,78	0,00	0,92	0,00	0,76	0,00	1,17	0,01	1,09	0,02	1,03	0,52	1,04	0,27	1,19	0,18	1,19	0,08
1334	ANXA5	1,02	0,45	1,12	0,01	1,12	0,00	1,04	0,25	0,87	0,01	0,86	0,01	0,85	0,00	0,98	0,53	0,76	0,00	1,08	0,02	0,97	0,24	0,83	0,00	1,03	0,36	0,82	0,00	0,79	0,00
1335	TSNAX	1,16	0,00	0,83	0,62	0,88	0,03	1,13	0,06	1,30	0,00	1,54	0,00	1,22	0,00	1,25	0,00	1,15	0,01	1,01	0,81	1,12	0,02	1,61	0,17	1,59	0,18	1,80	0,00	1,39	0,01
1338		1,92	0,00	0,96	0,96	0,34	0,05	1,78	0,02	2,97	0,00	5,28	0,00	2,01	0,00	2,27	0,00	2,87	0,00	0,71	0,02	0,90	0,35	2,34	0,13	2,02	0,23	11,65	0,00	7,15	0,00
1339		0,79	0,04	0,84	0,65	1,08	0,92	0,77	0,01	0,78	0,01	0,66	0,00	0,71	0,21	0,88	0,59	0,64	0,13	0,87	0,01	0,87	0,03	0,84	0,69	1,02	0,97	0,55	0,51	0,58	0,54
1340		0,81	0,06	1,20	0,39	1,76	0,07	0,88	0,58	0,65	0,11	0,67	0,12	0,89	0,51	1,03	0,83	1,18	0,46	1,49	0,03	1,10	0,38	0,74	0,18	0,86	0,44	0,52	0,04	0,67	0,18
1341	COPE,ANXA5	0,91	0,01	1,08	0,03	0,85	0,00	0,94	0,16	0,90	0,03	0,77	0,00	0,75	0,00	0,98	0,01	0,71	0,00	1,01	0,68	0,88	0,00	0,82	0,00	0,96	0,08	0,89	0,01	0,89	0,02
1343	ANXA4	0,91	0,07	1,06	0,01	1,16	0,00	0,87	0,00	0,83	0,00	0,76	0,00	0,88	0,00	0,86	0,00	0,81	0,00	0,92	0,12	0,87	0,02	0,76	0,00	0,73	0,00	0,64	0,00	0,63	0,00
1345	ANXA5,SFRS2	1,51	0,00	1,54	0,00	1,93	0,00	1,40	0,00	1,05	0,53	1,33	0,02	0,86	0,22	1,31	0,06	0,84	0,16	1,04	0,35	0,88	0,00	0,77	0,00	1,31	0,02	0,77	0,02	0,67	0,00
1349		0,93	0,02	0,96	0,12	0,80	0,00	0,93	0,16	0,89	0,03	0,82	0,01	0,91	0,02	0,94	0,06	0,84	0,02	0,86	0,01	0,76	0,00	0,79	0,00	0,75	0,00	0,87	0,10	0,80	0,01
1351	ANXA3,CAPZB,IPYR	0,77	0,00	0,81	0,00	0,59	0,00	0,78	0,00	0,78	0,00	0,63	0,00	0,81	0,00	0,79	0,00	0,74	0,00	1,00	0,69	0,95	0,00	0,93	0,02	0,88	0,00	1,04	0,13	1,13	0,00
1352	ECH1,PSDE	0,81	0,00	0,73	0,41	0,63	0,40	0,79	0,00	0,72	0,00	0,72	0,00	0,77	0,00	0,74	0,00	0,52	0,00	1,15	0,01	1,29	0,00	1,16	0,71	1,38	0,40	1,32	0,63	1,10	0,87
1354	C1QBP	0,76	0,00	0,86	0,00	0,69	0,00	0,91	0,00	0,85	0,00	0,71	0,00	0,77	0,00	0,95	0,00	0,69	0,00	1,09	0,00	0,97	0,20	0,89	0,01	1,04	0,17	0,93	0,20	0,95	0,32
1355	MARE1,EFHD2	0,88	0,00	0,92	0,01	0,77	0,00	0,91	0,00	0,84	0,00	0,74	0,00	0,86	0,00	0,93	0,00	0,80	0,00	0,94	0,00	0,84	0,00	0,82	0,00	0,86	0,00	0,87	0,01	0,89	0,01
1358	UBA1,3HIDH	0,67	0,00	0,69	0,00	0,50	0,00	0,62	0,00	0,53	0,00	0,47	0,00	0,69	0,00	0,64	0,00	0,47	0,00	1,04	0,50	1,09	0,09	0,87	0,01	1,00	0,96	1,04	0,53	1,01	0,85
1359		0,92	0,01	0,93	0,01	0,82	0,00	0,92	0,17	0,75	0,00	0,78	0,01	0,79	0,05	0,92	0,36	0,74	0,03	1,18	0,00	1,07	0,06	0,96	0,33	1,23	0,00	1,14	0,12	1,12	0,12
1360	MARE1,TBCB,ANXA5	0,91	0,00	0,94	0,02	0,78	0,00	0,93	0,00	0,79	0,00	0,76	0,00	0,82	0,00	0,94	0,00	0,75	0,00	0,98	0,12	0,89	0,00	0,80	0,00	0,98	0,38	0,92	0,00	0,95	0,01
1363	C1QBP	1,21	0,00	1,18	0,00	0,97	0,27	1,29	0,00	0,99	0,73	0,99	0,52	1,10	0,01	1,36	0,00	1,06	0,14	1,03	0,18	0,83	0,00	0,82	0,00	1,06	0,05	0,99	0,67	1,00	0,90
1364	NUDT5,ANXA5,SFRS2	0,90	0,00	0,91	0,00	0,83	0,00	1,01	0,68	0,83	0,00	0,76	0,00	0,93	0,00	1,05	0,01	0,81	0,00	1,13	0,00	1,03	0,11	0,92	0,01	1,14	0,00	0,93	0,10	0,96	0,26
1366		0,70	0,00	0,54	0,00	0,32	0,00	0,89	0,01	0,69	0,00	0,71	0,00	0,91	0,04	1,09	0,08	0,94	0,47	1,36	0,00	1,32	0,00	1,36	0,00	2,04	0,00	2,36	0,00	2,97	0,00
1367	RFA2	1,02	0,77	0,22	0,01	0,16	0,00	0,99	0,87	0,83	0,01	0,89	0,10	0,99	0,84	0,97	0,42	0,79	0,00	1,08	0,42	1,16	0,08	4,19	0,01	5,28	0,00	6,14	0,00	5,80	0,00
1369	TPM4	0,92	0,00	0,88	0,00	0,66	0,00	0,89	0,00	0,77	0,00	0,66	0,00	0,80	0,00	0,87	0,00	0,71	0,00	0,96	0,02	0,86	0,00	0,88	0,00	0,97	0,20	1,01	0,77	1,07	0,01
1372	TPM3,TPM4,TPM1,TP M2,TPM3L	0,80	0,00	0,77	0,00	0,58	0,00	0,85	0,00	0,72	0,00	0,67	0,00	0,83	0,01	0,92	0,16	0,75	0,00	1,11	0,01	1,02	0,52	0,98	0,41	1,16	0,01	1,22	0,00	1,28	0,00
1373	PSME2,1433E,PHB,14 33Z	1,21	0,00	1,42	0,00	1,82	0,00	0,96	0,02	0,87	0,00	0,78	0,00	0,77	0,00	0,82	0,00	0,72	0,00	0,85	0,00	0,75	0,00	0,65	0,00	0,67	0,00	0,46	0,00	0,46	0,00
1374	TPM3,TPM4,TPM2,TP M3L	0,93	0,00	0,91	0,00	0,68	0,00	0,95	0,00	0,81	0,00	0,70	0,00	0,83	0,00	0,91	0,00	0,73	0,00	1,02	0,19	0,93	0,00	0,89	0,00	1,04	0,06	1,04	0,14	1,11	0,00
1375	RFA2	0,66	0,00	0,51	0,00	0,41	0,00	0,83	0,00	0,63	0,00	0,50	0,00	0,90	0,00	0,87	0,00	0,51	0,00	1,19	0,09	1,30	0,02	1,18	0,00	1,64	0,00	1,14	0,06	1,19	0,00
1378	CLIC1	0,88	0,00	0,85	0,00	0,71	0,00	0,90	0,00	0,80	0,00	0,67	0,00	0,87	0,00	0,94	0,00	0,77	0,00	0,98	0,32	0,92	0,00	0,90	0,01	1,02	0,38	0,91	0,02	1,00	0,93
1379		0,81	0,01	0,59	0,00	0,48	0,00	0,83	0,00	0,75	0,00	0,51	0,00	0,67	0,00	0,95	0,11	0,41	0,00	1,15	0,03	0,92	0,22	1,41	0,00	1,78	0,00	1,19	0,04	0,96	0,49
1382	EF1B	1,00	0,92	0,99	0,66	0,85	0,00	1,08	0,00	0,89	0,00	0,81	0,00	0,97	0,01	1,12	0,00	0,97	0,01	0,97	0,09	0,77	0,00	0,80	0,00	0,90	0,00	0,85	0,00	0,91	0,01
1383		0,78	0,00	0,80	0,00	0,64	0,00	0,92	0,00	0,80	0,00	0,74	0,00	0,78	0,00	0,95	0,01	0,74	0,00	0,99	0,73	0,82	0,00	0,84	0,02	0,97	0,51	0,98	0,81	0,95	0,55
1386	ALBU	0,75	0,00	0,72	0,00	0,61	0,00	0,78	0,00	0,82	0,00	0,65	0,00	0,73	0,00	0,85	0,00	0,66	0,00	1,00	0,91	0,89	0,05	1,09	0,16	1,07	0,14	1,04	0,67	0,99	0,86
1391	1433E,CLIC1	0,90	0,02	0,84	0,04	0,70	0,01	0,99	0,78	0,76	0,00	0,70	0,00	0,89	0,04	1,10	0,07	0,70	0,00	1,05	0,21	1,00	0,93	0,86	0,09	1,33	0,00	0,96	0,65	1,02	0,81
1392	PSME2	0,87	0,00	0,82	0,00	0,71	0,00	0,81	0,00	0,67	0,00	0,63	0,00	0,70	0,00	0,79	0,00	0,57	0,00	1,11	0,00	1,01	0,51	0,98	0,25	1,21	0,00	1,05	0,06	1,01	0,72
1393	GSTO1	0,90	0,02	0,28	0,01	0,00	0,00	0,96	0,13	0,90	0,01	0,76	0,00	0,98	0,35	0,97	0,02	0,84	0,00	1,11	0,02	1,13	0,01	3,41	0,02	3,67	0,01	1.000,00	0,00	1.000,00	0,00
1394		1,34	0,00	1,12	0,06	1,50	0,00	1,13	0,00	0,88	0,00	0,93	0,00	0,90	0,08	1,15	0,02	0,85	0,02	0,87	0,00	0,77	0,00	0,81	0,00	1,17	0,00	0,64	0,00	0,65	0,00
1395		1,15	0,01	0,94	0,18	0,83	0,02	1,01	0,62	0,87	0,00	0,81	0,00	1,00	0,67	1,10	0,03	0,83	0,00	0,96	0,07	0,88	0,00	1,00	0,94	1,18	0,01	1,06	0,21	1,00	0,96
1396	PSME3,GSTO1	0,92	0,00	0,90	0,00	0,75	0,00	0,97	0,05	0,83	0,00	0,76	0,00	0,96	0,00	0,97	0,00	0,81	0,00	1,12	0,00	1,14	0,00	0,97	0,20	1,17	0,00	1,08	0,02	1,17	0,00
1398		1,85	0,01	2,35	0,05	1,60	0,07	0,97	0,47	0,88	0,04	0,96	0,53	0,70	0,16	1,38	0,09	0,63	0,11	1,03	0,53	0,93	0,23	0,73	0,26	1,46	0,08	1,18	0,04	0,98	0,83
1399	3HIDH,PSME1,CDV3	0,87	0,00	0,86	0,00	0,76	0,00	0,86	0,00	0,69	0,00	0,65	0,00	0,78	0,00	0,78	0,00	0,58	0,00	1,21	0,00	1,16	0,00	0,97	0,29	1,17	0,00	1,03	0,24	0,98	0,44
1401	CPNS1,GDIR	0,75	0,00	0,93	0,02	0,67	0,00	0,84	0,00	0,82	0,00	0,62	0,00	0,71	0,00	0,81	0,00	0,62	0,00	1,06	0,00	0,93	0,00	0,83	0,00	0,85	0,00	0,87	0,00	0,91	0,00
1402	PSB7	0,84	0,01	0,85	0,00	0,62	0,00	0,93	0,01	0,90	0,00	0,74	0,00	0,90	0,00	0,95	0,00	0,85	0,00	0,93	0,18	0,86	0,03	0,89	0,00	0,90	0,00	1,00	0,91	1,11	0,00
1403	1433E	0,87	0,00	0,83	0,00	0,68	0,00	0,93	0,00	0,68	0,00	0,64	0,00	0,71	0,00	0,88	0,00	0,59	0,00	1,11	0,00	0,92	0,00	0,85	0,00	1,20	0,00	0,97	0,24	0,98	0,45
1404	SPEE,CATD	0,98	0,20	1,08	0,04	1,12	0,00	1,13	0,00	0,93	0,00	0,90	0,00	1,21	0,00	1,31	0,00	1,03	0,19	1,26	0,00	1,29	0,00	0,94	0,06	1,28	0,00	0,88	0,00	0,97	0,07
1406		1,29	0,00	1,15	0,08	1,46	0,00	1,04	0,37	0,94	0,15	0,87	0,02	0,92	0,14	1,07	0,15	0,89	0,04	0,74	0,00	0,68	0,00	0,76	0,01	0,89	0,11	0,55	0,00	0,58	0,00
1408	GSTO1,ERP29	0,92	0,00	0,56	0,04	0,25	0,01	0,95	0,03	0,84	0,00	0,75	0,00	0,86	0,00	0,93	0,00	0,67	0,00	1,05	0,03	0,98	0,12	1,53	0,14	1,74	0,05	3,07	0,02	2,84	0,03
1409	PSME2,CLIC4	0,81	0,00	0,77	0,00	0,64	0,00	0,79	0,00	0,74	0,00	0,61	0,00	0,80	0,00	0,84	0,00	0,68	0,00	1,04	0,02	1,04	0,01	1,02	0,14	1,15	0,00	1,03	0,19	1,13	0,00
1410		0,78	0,00	0,86	0,05	0,66	0,00	0,65	0,00	0,78	0,00	0,50	0,00	0,56	0,00	0,67	0,00	0,43	0,00	0,82	0,00	0,73	0,00	0,90	0,16	0,79	0,01	0,74	0,04	0,67	0,02
1411		1,11	0,00	1,00	0,94	0,98	0,34	1,01	0,51	0,75	0,00	0,80	0,00	0,85	0,00	0,94	0,01	0,72	0,00	1,08	0,00	1,00	0,78	0,89	0,04	1,25	0,00	0,98	0,36	0,97	0,12
1412	PA1B2,PSME1,DDAH2	0,80	0,00	0,85	0,00	0,74	0,00	0,91	0,00	0,79	0,00	0,72	0,00	0,92	0,00	0,97	0,01	0,76	0,00	1,14	0,00	1,14	0,00	0,94	0,06	1,13	0,00	0,98	0,42	1,02	0,49
1414		1,08	0,00	1,13	0,01	1,12	0,03	0,93	0,00	0,78	0,00	0,75	0,00	0,80	0,00	0,92	0,07	0,70	0,00	1,00	0,66	0,97	0,07	0,80	0,00	1,08	0,07	0,77	0,00	0,82	0,01
1417	PSA3	0,87	0,00	0,93	0,02	0,75	0,00	0,93	0,00	0,81	0,00	0,71	0,00	0,82	0,00	0,91	0,00	0,73	0,00	1,04	0,00	0,94	0,00	0,85	0,00	0,98	0,46	0,92	0,02	0,98	0,47
1419	TIF1B,1433G	0,90	0,00	0,95	0,10	0,75	0,00	0,88	0,00	0,78	0,00	0,68	0,00	0,77	0,00	0,88	0,00	0,69	0,00	1,02	0,28	0,89	0,00	0,85	0,00	0,97	0,40	0,95	0,02	0,97	0,21
1420	HSPB1	1,15	0,00	1,05	0,27	0,73	0,00	1,21	0,00	0,98	0,30	0,95	0,10	0,92	0,01	1,25	0,00	0,82	0,01	0,98	0,30	0,80	0,00	0,86	0,01	1,19	0,00	1,19	0,01	1,11	0,19
1425	1433G	0,89	0,00	1,01	0,82	0,71	0,00	0,88	0,00	0,88	0,00	0,68	0,00	0,79	0,00	0,86	0,00	0,78	0,00	0,98	0,23	0,83	0,00	0,86	0,00	0,80	0,00	0,95	0,07	1,02	0,40
1429	1433T,1433Z	0,81	0,00	0,78	0,00	0,61	0,00	0,86	0,00	0,66	0,00	0,59	0,00	0,68	0,00	0,85	0,00	0,62	0,00	1,07	0,02	0,87	0,00	0,86	0,00	1,13	0,00	0,99	0,64	1,06	0,02
1430	1433Z	0,94	0,00	0,90	0,00	0,73	0,00	0,95	0,02	0,74	0,00	0,70	0,00	0,77	0,00	0,92	0,04	0,69	0,00	1,03	0,07	0,90	0,00	0,85	0,00	1,14	0,01	0,98	0,32	1,05	0,19
1431		0,92	0,01	0,98	0,62	0,86	0,00	1,03	0,23	0,82	0,00	0,75	0,00	0,92	0,02	1,00	0,87	0,74	0,00	1,19	0,00	1,16	0,00	0,89	0,01	1,18	0,00	0,92	0,02	0,99	0,65
1432	NNMT	0,75	0,00	0,79	0,00	0,68	0,00	0,81	0,00	0,75	0,00	0,63	0,00	0,87	0,00	0,92	0,00	0,67	0,00	1,13	0,00	1,08	0,00	1,00	0,78	1,09	0,00	0,95	0,02	0,91	0,00
1433	PSME1,6PGL,ERP29	1,04	0,38	1,28	0,00	1,52	0,00	1,13	0,00	0,98	0,18	0,96	0,02	0,86	0,00	0,97	0,01	0,81	0,00	1,07	0,13	0,93	0,14	0,76	0,00	0,86	0,00	0,62	0,00	0,60	0,00
1434		1,02	0,47	1,11	0,01	0,90	0,12	1,08	0,05	0,88	0,00	0,81	0,00	0,83	0,00	0,95	0,20	0,66	0,00	1,16	0,00	1,16	0,00	0,88	0,00	1,21	0,00	0,99	0,84	1,04	0,48
1435	ECHM	0,77	0,00	0,69	0,00	0,50	0,00	0,79	0,00	0,70	0,00	0,62	0,00	0,83	0,00	0,85	0,00	0,56	0,00	1,08	0,00	1,15	0,00	1,06	0,23	1,32	0,00	1,30	0,04	1,20	0,12
1437		0,86	0,00	0,85	0,05	0,66	0,01	0,95	0,13	0,79	0,00	0,71	0,00	0,62	0,00	0,97	0,34	0,51	0,00	1,03	0,45	0,71	0,00	0,86	0,08	1,11	0,16	1,01	0,97	0,76	0,07

1438	1433S	0,86	0,00	0,87	0,00	0,67	0,00	0,88	0,00	0,73	0,00	0,65	0,00	0,82	0,00	0,85	0,00	0,64	0,00	1,02	0,17	0,95	0,00	0,84	0,00	0,98	0,49	0,97	0,27	0,96	0,09
1439		1,09	0,00	1,14	0,00	1,20	0,00	1,07	0,01	0,92	0,00	0,90	0,00	0,99	0,86	1,15	0,05	0,87	0,07	1,02	0,23	0,97	0,02	0,84	0,00	1,08	0,04	0,78	0,00	0,77	0,00
1440	CAPZB	0,78	0,09	0,95	0,29	0,77	0,02	0,87	0,00	0,75	0,00	0,68	0,00	0,81	0,00	0,88	0,00	0,62	0,00	1,15	0,34	1,15	0,34	0,81	0,00	1,01	0,80	0,90	0,27	0,88	0,19
1443	PNPO	0,84	0,00	0,67	0,04	0,16	0,00	0,91	0,01	0,85	0,00	0,75	0,00	0,84	0,00	0,93	0,00	0,77	0,00	1,01	0,80	0,95	0,04	1,19	0,35	1,31	0,15	4,42	0,00	4,60	0,00
1446	RANG,PSA3	0,72	0,00	0,81	0,00	0,59	0,00	0,83	0,00	0,70	0,00	0,59	0,00	0,67	0,00	0,77	0,00	0,57	0,00	1,10	0,00	0,96	0,02	0,83	0,00	0,98	0,47	0,96	0,10	1,00	0,97
1449	1433B,1433G,TIF1B	0,86	0,00	0,92	0,02	0,74	0,00	0,90	0,00	0,77	0,00	0,68	0,00	0,67	0,00	0,85	0,00	0,58	0,00	1,07	0,01	0,90	0,00	0,85	0,00	1,05	0,16	0,95	0,16	0,91	0,02
1451		0,36	0,01	0,44	0,05	0,16	0,01	0,48	0,30	0,63	0,47	0,25	0,13	0,20	0,05	0,25	0,06	0,10	0,03	1,56	0,36	0,71	0,37	1,67	0,38	0,74	0,53	1,87	0,21	0,79	0,85
1452		1,09	0,06	1,06	0,07	0,84	0,00	1,09	0,17	0,86	0,07	0,81	0,03	0,81	0,01	0,98	0,72	0,78	0,00	0,97	0,34	0,78	0,00	0,79	0,00	0,97	0,10	0,93	0,12	0,97	0,34
1453	HSPB1,ECHM	0,87	0,00	0,61	0,01	0,16	0,00	0,92	0,00	0,85	0,00	0,82	0,00	0,81	0,00	0,92	0,00	0,77	0,00	0,95	0,00	0,85	0,00	1,25	0,17	1,36	0,06	4,72	0,00	4,44	0,00
1455	HSPB1	1,03	0,42	1,02	0,79	0,76	0,01	1,24	0,00	0,98	0,61	0,95	0,26	1,27	0,00	1,28	0,00	1,24	0,00	1,13	0,01	0,93	0,04	0,90	0,21	0,94	0,47	1,18	0,04	1,23	0,02
1457		0,84	0,00	0,88	0,00	0,68	0,00	0,93	0,01	0,82	0,00	0,72	0,00	0,75	0,00	0,88	0,01	0,69	0,00	1,11	0,00	1,01	0,20	0,94	0,00	1,12	0,00	1,06	0,03	1,15	0,00
1458	IDI1,PSB10,EXOS4	0,84	0,01	0,89	0,01	0,77	0,03	0,85	0,00	0,66	0,00	0,63	0,00	0,81	0,00	0,81	0,00	0,59	0,00	1,20	0,00	1,18	0,00	0,88	0,00	1,11	0,00	0,96	0,68	0,93	0,60
1460	PSB7	1,05	0,22	1,05	0,29	0,99	0,73	1,02	0,40	0,90	0,01	0,87	0,00	0,90	0,00	0,98	0,21	0,76	0,00	0,97	0,40	0,93	0,04	0,86	0,01	1,01	0,73	0,88	0,00	0,84	0,00
1461		1,06	0,08	0,63	0,11	0,45	0,02	1,10	0,01	1,00	0,98	0,99	0,87	0,68	0,00	1,00	0,91	0,61	0,00	0,92	0,02	0,82	0,00	1,43	0,23	2,05	0,02	1,98	0,05	1,75	0,10
1463	ERP29,TPIS,ETHE1	0,94	0,01	0,65	0,16	0,61	0,24	1,07	0,00	0,82	0,00	0,76	0,00	1,05	0,00	1,04	0,00	0,77	0,00	1,10	0,00	1,02	0,13	1,22	0,54	1,46	0,22	1,21	0,68	1,17	0,75
1464		0,29	0,00	0,47	0,05	0,21	0,01	0,78	0,39	1,10	0,75	0,84	0,53	1,91	0,09	0,94	0,74	8,92	0,30	1,37	0,09	2,52	0,03	1,21	0,61	0,78	0,52	2,04	0,10	16,32	0,36
1465	IF6	0,98	0,27	1,07	0,05	0,69	0,00	1,09	0,01	1,06	0,00	0,83	0,00	0,96	0,10	1,16	0,00	1,04	0,26	0,93	0,02	0,77	0,00	0,82	0,00	0,84	0,00	1,00	0,94	1,16	0,01
1466		2,21	0,00	2,51	0,00	2,04	0,00	1,76	0,00	1,70	0,00	1,84	0,00	1,51	0,00	2,05	0,00	1,52	0,00	0,78	0,00	0,67	0,00	0,67	0,00	0,80	0,00	0,89	0,00	0,73	0,00
1467	CPNS1	0,94	0,00	0,99	0,65	0,84	0,00	0,95	0,00	0,93	0,00	0,76	0,00	1,00	0,75	0,98	0,04	0,89	0,00	0,97	0,05	0,96	0,02	0,90	0,00	0,88	0,00	0,87	0,00	0,94	0,00
1468	PHB,HSPB1	0,97	0,29	1,02	0,52	0,79	0,00	0,97	0,04	0,82	0,00	0,71	0,00	0,77	0,00	0,88	0,00	0,58	0,00	0,95	0,02	0,84	0,00	0,77	0,00	0,91	0,01	0,86	0,01	0,77	0,00
1470		0,19	0,00	0,47	0,03	0,20	0,00	0,26	0,11	0,40	0,21	0,14	0,07	0,20	0,10	0,28	0,13	0,05	0,06	1,88	0,14	1,16	0,48	1,19	0,74	0,67	0,35	0,93	0,91	0,29	0,19
1471	PRDX4	0,90	0,29	1,11	0,01	1,21	0,00	1,08	0,00	0,98	0,13	0,91	0,00	0,99	0,12	1,16	0,00	0,92	0,00	1,04	0,66	0,89	0,31	0,77	0,00	0,85	0,00	0,65	0,00	0,62	0,00
1472		1,04	0,22	0,99	0,89	1,15	0,27	1,08	0,01	1,05	0,08	0,96	0,26	1,09	0,00	1,06	0,01	0,99	0,71	0,83	0,00	0,74	0,00	0,85	0,07	0,76	0,01	0,67	0,02	0,60	0,01
1473	ECHM	1,07	0,02	0,93	0,60	0,47	0,03	1,17	0,00	0,97	0,27	1,06	0,03	0,98	0,39	1,13	0,00	0,91	0,00	1,04	0,05	0,93	0,00	0,99	0,96	1,23	0,11	2,17	0,03	1,99	0,05
1475		1,08	0,45	2,03	0,01	1,57	0,01	0,73	0,08	1,85	0,00	1,12	0,35	1,21	0,13	1,75	0,01	1,69	0,00	1,04	0,76	0,83	0,04	1,40	0,02	0,64	0,03	1,10	0,33	0,80	0,06
1476	PSA5	1,07	0,00	1,06	0,01	0,85	0,00	0,98	0,29	0,92	0,00	0,79	0,00	0,94	0,00	0,95	0,00	0,86	0,00	0,97	0,10	0,94	0,00	0,92	0,00	0,96	0,01	0,98	0,44	1,07	0,01
1477		0,83	0,00	0,90	0,22	0,79	0,14	0,95	0,06	0,88	0,00	0,80	0,00	0,85	0,00	0,95	0,02	0,74	0,00	1,02	0,37	0,92	0,04	0,87	0,14	0,93	0,43	0,91	0,58	0,84	0,33
1478	ACTG,NNMT,CLIC4	1,73	0,00	1,93	0,00	2,47	0,01	1,35	0,00	1,19	0,00	1,11	0,01	0,97	0,20	1,08	0,00	0,90	0,00	0,89	0,00	0,74	0,00	0,69	0,00	0,73	0,00	0,51	0,01	0,48	0,01
1479	CI032	1,07	0,01	1,01	0,80	0,79	0,00	0,90	0,01	0,91	0,00	0,71	0,00	0,81	0,00	0,91	0,00	0,83	0,00	0,87	0,00	0,78	0,00	0,93	0,01	0,93	0,01	0,93	0,02	1,09	0,03
1481		0,97	0,15	0,88	0,30	0,34	0,00	1,06	0,01	0,90	0,00	0,97	0,09	0,80	0,00	0,99	0,62	0,79	0,00	0,98	0,29	0,83	0,00	0,92	0,53	1,14	0,28	2,61	0,00	2,38	0,00
1484		0,84	0,31	1,04	0,54	0,91	0,16	1,07	0,11	0,99	0,80	0,84	0,00	0,94	0,01	0,98	0,18	0,78	0,00	1,15	0,41	0,99	0,95	0,86	0,04	0,83	0,01	0,84	0,01	0,76	0,00
1486		1,06	0,64	0,57	0,02	0,44	0,01	1,14	0,41	0,92	0,63	1,14	0,50	1,81	0,20	0,99	0,98	1,46	0,41	1,29	0,01	1,70	0,08	1,94	0,00	1,72	0,07	3,07	0,00	3,27	0,03
1488		0,91	0,12	0,73	0,01	0,68	0,01	1,08	0,09	0,84	0,00	0,91	0,01	1,08	0,00	1,27	0,00	0,90	0,11	1,07	0,23	1,13	0,02	1,02	0,75	1,64	0,00	1,20	0,06	1,26	0,07
1490		0,42	0,09	0,60	0,31	0,47	0,18	0,60	0,40	1,08	0,87	1,01	0,99	4,32	0,16	1,08	0,27	20,91	0,31	3,24	0,02	4,63	0,15	4,06	0,01	0,81	0,63	4,85	0,01	20,09	0,39
1491	UCHL1	0,83	0,00	0,88	0,02	0,63	0,00	0,95	0,02	0,89	0,00	0,77	0,00	0,84	0,00	1,00	0,70	0,88	0,00	1,01	0,66	0,90	0,01	0,90	0,03	1,01	0,84	1,08	0,26	1,25	0,01
1492	HSPB1,PIMT	0,88	0,00	0,41	0,02	0,60	0,45	0,96	0,02	0,80	0,00	0,76	0,00	0,87	0,00	1,00	0,72	0,73	0,00	1,07	0,01	1,07	0,01	1,91	0,10	2,59	0,02	1,25	0,78	1,32	0,72
1494	NDUS3,HSPB1,PSB4	0,89	0,00	0,91	0,09	0,76	0,00	0,95	0,01	0,94	0,01	0,80	0,00	0,95	0,08	0,97	0,02	0,84	0,00	0,98	0,13	0,93	0,04	0,94	0,27	0,93	0,19	0,95	0,15	0,96	0,15
1495	GRB2	0,85	0,00	0,95	0,10	0,81	0,01	0,93	0,00	0,89	0,00	0,80	0,00	0,86	0,00	1,01	0,21	0,82	0,00	0,96	0,08	0,83	0,00	0,82	0,00	0,88	0,01	0,86	0,06	0,84	0,04
1497	PRDX4	1,08	0,11	0,92	0,19	0,92	0,47	1,06	0,05	0,96	0,07	0,92	0,02	1,01	0,67	1,02	0,46	0,94	0,02	0,96	0,11	0,95	0,05	1,02	0,66	1,13	0,02	0,97	0,81	1,04	0,72
1499		0,67	0,00	0,69	0,05	0,40	0,00	0,77	0,18	0,77	0,20	0,55	0,02	0,93	0,67	1,10	0,53	0,81	0,61	1,20	0,02	1,00	0,99	1,16	0,41	1,13	0,41	1,42	0,01	1,44	0,54
1501	TCTP,CBX1	1,04	0,01	1,00	0,96	0,86	0,00	1,10	0,00	0,91	0,00	0,82	0,00	0,98	0,09	1,07	0,00	0,92	0,00	0,91	0,00	0,72	0,00	0,78	0,00	0,82	0,00	0,81	0,00	0,81	0,00
1502	CRK	1,16	0,00	1,53	0,00	1,86	0,00	0,97	0,01	1,49	0,00	1,48	0,00	1,21	0,00	1,23	0,00	1,41	0,00	0,65	0,00	0,67	0,00	0,76	0,00	0,52	0,00	0,61	0,00	0,49	0,00
1505		0,78	0,00	0,83	0,00	0,76	0,00	0,84	0,00	0,81	0,00	0,69	0,00	0,84	0,00	0,90	0,00	0,77	0,00	1,03	0,16	0,98	0,11	0,92	0,02	0,98	0,43	0,87	0,07	0,92	0,18
1506		0,93	0,01	0,88	0,02	0,65	0,00	1,01	0,42	0,94	0,01	0,81	0,00	1,04	0,02	1,16	0,00	0,97	0,02	0,98	0,31	0,96	0,05	0,96	0,30	1,12	0,02	1,12	0,19	1,27	0,01
1509		1,29	0,02	1,01	0,94	0,93	0,65	1,09	0,05	0,86	0,00	0,94	0,09	0,91	0,10	1,03	0,55	0,95	0,38	0,87	0,05	0,75	0,00	0,88	0,07	1,09	0,22	1,03	0,80	1,08	0,59
1510	PRDX3	0,69	0,00	0,28	0,00	0,05	0,00	0,79	0,00	0,65	0,00	0,53	0,00	0,79	0,00	0,85	0,00	0,55	0,00	1,18	0,00	1,23	0,00	2,38	0,04	3,27	0,01	11,47	0,00	12,58	0,00
1511	PARK7	0,86	0,00	0,68	0,09	0,50	0,12	0,95	0,00	0,81	0,00	0,72	0,00	0,89	0,00	0,92	0,00	0,71	0,00	1,07	0,00	1,02	0,13	1,16	0,50	1,34	0,18	1,41	0,46	1,42	0,45
1512	CATB	1,13	0,00	1,30	0,00	1,99	0,00	1,00	0,85	1,26	0,00	1,56	0,00	1,03	0,04	1,08	0,00	1,14	0,00	0,80	0,00	0,88	0,00	0,88	0,00	0,80	0,00	0,71	0,00	0,55	0,00
1513	GDIR1,LGUL	0,78	0,00	0,84	0,00	0,73	0,00	0,85	0,00	0,81	0,00	0,72	0,00	0,82	0,00	0,87	0,00	0,71	0,00	1,06	0,01	1,04	0,01	0,93	0,00	1,02	0,30	0,96	0,26	0,95	0,22
1514		2,15	0,00	0,71	0,49	0,46	0,06	1,10	0,02	1,24	0,00	1,35	0,00	0,78	0,00	1,10	0,00	0,99	0,82	0,68	0,00	0,51	0,00	2,33	0,05	2,15	0,08	3,95	0,00	3,03	0,01
1516	GSTP1	0,81	0,00	0,86	0,00	0,68	0,00	0,87	0,00	0,75	0,00	0,69	0,00	0,81	0,00	0,88	0,00	0,68	0,00	1,10	0,00	1,11	0,00	0,88	0,00	1,12	0,00	1,01	0,49	1,09	0,00
1518		0,95	0,57	0,63	0,02	0,69	0,10	1,11	0,37	1,33	0,03	0,99	0,93	1,01	0,98	1,02	0,94	1,35	0,17	0,83	0,01	0,70	0,00	1,50	0,03	1,06	0,71	1,02	0,93	1,29	0,22
1519	PSD10	0,92	0,09	0,86	0,01	0,82	0,08	0,94	0,00	0,86	0,00	0,76	0,00	0,86	0,00	0,93	0,00	0,80	0,00	1,01	0,84	0,91	0,09	0,98	0,68	1,05	0,29	0,90	0,36	0,94	0,57
1520		1,10	0,03	1,24	0,00	1,07	0,26	1,09	0,00	0,97	0,36	0,99	0,51	0,88	0,00	1,11	0,00	0,88	0,00	0,97	0,23	0,87	0,00	0,77	0,00	0,98	0,24	0,91	0,07	0,90	0,05
1521		0,86	0,05	0,79	0,00	0,71	0,00	0,95	0,04	0,80	0,00	0,74	0,00	0,94	0,15	1,04	0,39	0,83	0,00	1,07	0,24	0,91	0,17	0,99	0,69	1,11	0,02	1,01	0,91	0,97	0,26
1522	PFD3	0,85	0,01	0,73	0,15	0,56	0,07	0,95	0,01	0,88	0,00	0,73	0,00	0,92	0,08	1,03	0,15	0,78	0,03	1,01	0,86	0,90	0,10	1,10	0,67	1,17	0,46	1,19	0,58	1,15	0,69
1523		1,36	0,00	1,21	0,00	1,21	0,02	1,23	0,00	1,11	0,00	1,03	0,10	1,11	0,00	1,37	0,00	1,19	0,00	0,78	0,00	0,57	0,00	0,79	0,00	0,78	0,00	0,73	0,00	0,68	0,00
1524	NDUV2	1,12	0,07	1,08	0,28	0,99	0,96	1,05	0,10	0,92	0,04	0,79	0,00	0,94	0,05	1,12	0,00	0,73	0,00	1,00	0,93	0,90	0,09	0,92	0,24	1,12	0,09	0,86	0,29	0,79	0,15
1526	UBE2K,RPE,CBX5,GSTP1	0,89	0,00	0,96	0,10	0,95	0,06	0,93	0,00	0,89	0,00	0,83	0,00	0,85	0,00	0,92	0,00	0,78	0,00	0,99	0,57	0,93	0,00	0,88	0,00	0,93	0,01	0,84	0,00	0,80	0,00
1530	CBX5,CATB	0,67	0,00	0,78	0,00	0,58	0,00	0,78	0,00	0,76	0,00	0,66	0,00	0,67	0,00	0,70	0,00	0,59	0,00	1,14	0,00	1,05	0,01	0,96	0,33	0,95	0,27	1,13	0,19	1,07	0,61
1532		0,64	0,02	0,38	0,01	0,10	0,00	0,74	0,05	1,14	0,77	0,38	0,00	0,06	0,07	0,09	0,08	0,15	0,10	1,01	0,92	0,55	0,01	2,64	0,17	1,37	0,54	3,24	0,06	8,38	0,30
1533		0,75	0,00	0,80	0,04	0,60	0,01	0,58	0,00	0,76	0,00	0,44	0,00	0,57	0,03	0,67	0,06	0,56	0,05	0,94	0,19	0,86	0,03	1,17	0,17	0,97	0,68	0,90	0,56	1,08	0,81
1534		0,64	0,00	0,75	0,01	0,56	0,00	0,55	0,00	0,58	0,00	0,37	0,00	0,56	0,01	0,69	0,02	0,39	0,00	1,19	0,00	0,96	0,63	1,07	0,51	1,01	0,86	0,92	0,29	0,77	0,15
1535		0,74	0,00	0,60	0,10	0,36	0,02	0,84	0,01	0,82	0,00	0,59	0,00	0,72	0,00	0,87	0,00	0,55	0,00	1,16	0,06	1,00	0,98	1,41	0,28	1,51	0,19	1,70	0,22	1,60	0,34
1536	SFRS3	0,76	0,04	0,83	0,11	0,81	0,13	0,79	0,00	0,72	0,00	0,59	0,00	0,87	0,00	0,83	0,00	0,66	0,00	1,21	0,05	1,22	0,04	1,01	0,91	1,06	0,48	0,86	0,20	0,88	0,34
1537		0,26	0,01	0,31	0,03	0,10	0,01	0,28	0,02	1,98	0,03	0,50	0,09	0,14	0,06	0,02	0,04	0,12	0,06	0,94	0,90	1,72	0,40	5,65	0,00	0,17	0,13	4,24	0,12	3,46	0,55
1538		0,88	0,00	0,92	0,13	0,81	0,04	0,98	0,61	0,80	0,00	0,73	0,00	0,80	0,00	0,95	0,04	0,64	0,00	1,04	0,36	0,94	0,02	0,81	0,01	1,07	0,17	0,84	0,12	0,82	0,10
1540		1,18	0,28	0,56	0,01	0,54	0,02	1,46	0,02	1,49	0,02	1,84	0,01	1,48	0,01	1,07	0,62	1,17	0,22	0,71	0,08	0,99	0,96	1,54	0,06	1,51	0,04	1,99	0,02	1,73	0,02
1541	PRDX2,GSTP1	0,71	0,00	0,77	0,00	0,59	0,00	0,85	0,00	0,71	0,00	0,65	0,00	0,74	0,00	0,84	0,00	0,60	0,00	1,24	0,00	1,19	0,00	0,95	0,23	1,25	0,00	1,14	0,01	1,17	0,00
1542		0,49	0,15	0,42	0,08	0,37	0,10	0,17	0,12	0,41	0,24	0,20	0,13	0,14	0,01	0,53	0,17	0,17	0,02	0,50	0,36	0,43	0,25	1,41	0,38	1,90	0,24	0,79	0,68	0,69	0,54
1543		0,32	0,02	0,41	0,04	0,20	0,02	0,15	0,02	0,43	0,07	0,09	0,01	0,58	0,44	0,38	0,00	0,10	0,00	0,76	0,75	2,67	0,51	1,68	0,22	1,36	0,54	0,70	0,71	0,73	0,80
1544		0,84	0,02	0,84	0,30	0,73	0,36	0,95	0,10	0,75	0,00	0,67	0,00	0,78	0,00	0,82	0,00	0,63	0,00	1,04	0,30	0,90	0,02	0,83	0,34	0,96	0,82	0,84	0,67	0,85	0,68
1545		0,76	0,04	0,82	0,05	0,80	0,14	0,94	0,17	0,76	0,00	0,68	0,00	0,79	0,01	0,99	0,65	0,52	0,02	1,15	0,21	0,94	0,65	0,86	0,13	1,09	0,28	0,79	0,15	0,59	0,13
1547	CBX1	0,90	0,01	0,92	0,04	0,67	0,00	1,01	0,62	0,94	0,01	0,78	0,00	0,93	0,10	0,97	0,40	0,87	0,01	1,02	0,30	0,94	0,01	0,92	0,02	0,95	0,08	1,05	0,41	1,17	0,04
1550		0,82	0,00	0,74	0,01	0,53	0,00	1,05	0,22	0,93	0,08	0,78	0,00	0,74	0,00	0,93	0,02	0,70	0,00	1,03	0,30	0,95	0,02	1,00	0,97	1,30	0,01	1,19	0,25	1,40	0,04
1553	STAM1,PSB9	0,99	0,92	1,10	0,21	0,86	0,17	0,90	0,01	0,93	0,28	0,69	0,00	0,71	0,00	0,92	0,03	0,70	0,00	0,94	0,15	0,74	0,00	0,87	0,12	0,86	0,02	0,84	0,13	0,84	0,09
1556		0,28	0,10	0,44	0,18	0,37	0,21	0,42	0,16	1,68	0,19	0,63	0,39	0,28	0,01	0,90	0,71	0,51	0,07	1,31	0,40	1,05	0,87	3,37	0,01	2,15	0,04	1,52	0,43	1,48	0,32
1557		0,30	0,10	0,56	0,45	0,77	0,63	0,13	0,05	0,66	0,40	1,36	0,77	0,20	0,04	0,69	0,35	0,41	0,09	0,25	0,31	0,39	0,42	0,65	0,68	0,72	0,74	0,98	0,98	0,31	0,11
1559		2,51	0,00	2,43	0,00	2,46	0,00	1,64	0,00	1,26	0,00	1,55	0,00	1,28	0,00	1,57	0,00	1,20	0,00	0,66	0,00	0,55	0,00	0,52	0,00	0,69	0,00	0,63	0,00	0,52	0,00
1560	ATP5H,CBX5	1,25	0,00	1,33	0,00	1,33	0,00	1,16	0,00	0,99	0,32	0,94	0,00	1,02	0,09	1,13	0,00	0,89	0,00	0,92	0,00	0,82	0,00	0,74	0,00	0,86	0,01	0,70	0,00	0,68	0,00
1562		1,08	0,01	1,04	0,62	1,05	0,20	1,27	0,00	0,73	0,00	0,91	0,06	0,85	0,00	1,13	0,01	0,69	0,00	1,16	0,00	0,93	0,00	0,69	0,00	1,29	0,01	0,86	0,01	0,77	0,00
1563		0,83	0,16	0,88	0,40	0,82	0,33	0,86	0,62	0,57	0,14	0,74	0,35	0,89	0,07	1,11	0,04	0,60	0,04	1,19	0,24	1,16	0,16	0,75	0,13	1,37	0,04	1,04	0,84	0,79	0,50
1564		0,17	0,11	0,38	0,21	0,16	0,15	0,01	0,05	1,23	0,78	0,22	0,12	0,52	0,44	4,28	0,37	0,14	0,09	0,06	0,30	9,68	0,30	3,45	0,26	36,62	0,26	1,49	0,64	2,84	0,48
1565	SFRS3	0,76	0,01	0,80	0,03	0,63	0,01	0,71	0,00	0,71	0,00	0,57	0,00	0,68	0,00	0,84	0,00	0,58	0,00	1,09	0,09	0,92	0,14	1,04	0,64	1,08	0,25	1,06	0,68	0,94	0,69
1566		1,58	0,03	1,48	0,19	1,18	0,49	1,55	0,04	1,74	0,02	2,05	0,00	0,61	0,44	0,80	0,70	0,96	0,93	0,89	0,33	0,74	0,02	1,06	0,79	1,05	0,82	1,57	0,03	1,57	0,16
1568		1,03	0,23	1,03	0,58	0,69	0,00	0,95	0,03	1,14	0,00	0,78	0,00	0,85	0,02	0,97	0,51	1,01	0,90	0,86	0,00	0,77	0,00	1,04	0,31	0,88	0,03	1,07	0,36	1,37	0,00
1569	APT	0,88	0,01	0,90	0,03	0,76	0,01	0,97	0,40	0,89	0,01	0,79	0,00	0,94	0,13	0,99	0,81	0,82	0,00	0,99	0,78	0,93	0,09	0,89	0,01	0,96	0,26	0,92	0,22	0,93	0,31
1570		0,64	0,10	0,55	0,06	0,26	0,02	1,18	0,69	1,98	0,07	2,04	0,03	1,04	0,92	0,92	0,84	0,74	0,59	1,03	0,92	0,99	0,98	1,98	0,02	1,01	0,96	4,36	0,00	1,73	0,41
1571		0,77	0,00	0,75	0,00	0,63	0,00	0,85	0,00	0,78	0,00	0,68	0,00	0,79	0,00	0,87	0,00	0,68	0,00	1,05	0,08	0,95	0,02	1,00	0,95	1,08	0,16	1,02	0,81	0,99	0,93
1572	SFRS3	0,55	0,00	0,40	0,01	0,03	0,00	0,73	0,01	0,62	0,00	0,43	0,00	0,64	0,00	0,79	0,00	0,31	0,00	1,11	0,13	0,98	0,70	1,30	0,50	1,66	0,16	10,80	0,00	7,63	0,00
1574		0,24	0,15	0,74	0,62	0,55	0,46	1,12	0,73	1,15	0,66	0,76	0,43	3,17	0,00	2,47	0,00	1,70	0,03	6,68	0,00	16,61	0,00	2,22	0,02	4,16	0,00	1,95	0,03	3,83	0,00
1575		1,10	0,36	1,05	0,74	0,98	0,92	0,99	0,79	1,16	0,04	1,01	0,84	1,02	0,70	1,06	0,23	1,00	0,98	0,70	0,02	0,60	0,00	0,86	0,38	0,66	0,05	0,82	0,43	0,67	0,18
1576	TCTP	0,84	0,00	0,81	0,00	0,59	0,00	0,91	0,01	0,81	0,00	0,69	0,00	0,79	0,00	0,90	0,01	0,69	0,00	0,99	0,56	0,81	0,00	0,91	0,05	0,95	0,21	1,07	0,50	0,99	0,95
1577		1,13	0,07	1,03	0,64	0,98	0,71	1,45	0,00	0,84	0,02	1,02	0,68	1,04	0,28	1,17	0,00	0,80	0,00	0,76	0,00	0,64	0,00	0,48	0,00	0,79	0,00	0,62	0,00	0,57	0,00
1578	SFRS3	0,79	0,02	0,73	0,01	0,75	0,10	0,75	0,03	0,55	0,00	0,44	0,00	0,71	0,00	0,69	0,00	0,55	0,00	1,10	0,31	0,85	0,03	0,87	0,07	0,90	0,08	0,67	0,08	0,70	0,10
1580		0,99	0,78	0,99	0,86	0,92	0,47	0,94	0,09	1,04	0,64	0,87	0,00	1,02	0,46	0,98	0,67	0,89	0,01	0,92	0,08	0,89	0,01	1,02	0,87	0,86	0,15	0,92	0,48	0,84	0,19
1582		1,39	0,27	1,37	0,41	1,05	0,90	1,47	0,23	2,15	0,12	1,91	0,05	0,83	0,69	1,06	0,90	1,54	0,48	0,90	0,58	0,99	0,94	1,34	0,46	1,28	0,33	1,56	0,07	2,44	0,25
1586	RBM8A	0,83	0,00	0,84	0,00	0,60	0,00	0,98	0,28	0,78	0,00	0,74	0,00	0,84	0,00	0,90	0,01	0,67	0,00	1,16	0,00	1,03	0,10	0,91	0,02	1,09	0,04	1,21	0,00	1,14	0,01
1589	SORCN	0,72	0,00	0,72	0,00	0,47	0,00	0,84	0,00	0,79	0,00	0,61	0,00	0,74	0,00	0,84	0,00	0,60	0,00	1,06	0,03	0,94	0,03	1,00	0,98	1,07	0,04	1,17	0,02	1,17	0,02
1590		0,93	0,52	0,69	0,05	0,31	0,00	1,18	0,19	0,94	0,56	0,85	0,21	1,96	0,00	1,89	0,00	1,46	0,01	1,37	0,01	1,87	0,00	1,48	0,01	2,42	0,00	2,92	0,00	4,11	0,00
1592		0,51	0,00	0,26	0,00	0,00	0,00	1,28	0,05	0,70	0,03	0,81	0,09	1,10	0,05	1,01	0,78	0,55	0,00	2,04	0,00	2,40	0,00	2,15	0,08	4,23	0,00	1.000,00	0,00	1.000,00	0,00

1593		1,00	0,98	0,91	0,02	0,87	0,00	1,28	0,01	0,75	0,00	0,87	0,06	0,82	0,00	1,11	0,01	0,60	0,00	1,35	0,00	1,09	0,02	0,87	0,01	1,63	0,00	1,06	0,01	0,91	0,01
1594		0,56	0,00	0,51	0,00	0,39	0,00	0,69	0,00	0,57	0,00	0,47	0,00	0,66	0,00	0,75	0,00	0,49	0,00	1,20	0,01	1,10	0,05	1,08	0,00	1,37	0,00	1,18	0,01	1,16	0,00
1596	BID,RBM8A	0,69	0,00	0,69	0,00	0,49	0,00	0,79	0,00	0,81	0,00	0,65	0,00	0,68	0,00	0,71	0,00	0,50	0,00	0,97	0,21	0,72	0,00	0,98	0,77	0,75	0,00	1,12	0,10	0,74	0,01
1598		2,21	0,00	1,82	0,01	3,25	0,00	1,99	0,00	2,32	0,00	2,31	0,00	1,88	0,00	1,82	0,00	2,59	0,00	0,51	0,00	0,41	0,00	0,72	0,04	0,49	0,00	0,40	0,00	0,39	0,00
1600		0,79	0,00	0,83	0,00	0,62	0,00	0,85	0,00	0,87	0,00	0,64	0,00	0,79	0,00	0,91	0,00	0,91	0,02	0,87	0,00	0,67	0,00	0,85	0,00	0,73	0,00	0,84	0,02	0,99	0,87
1601		0,20	0,18	0,16	0,15	0,21	0,25	0,07	0,00	0,88	0,52	0,04	0,00	2,16	0,59	0,91	0,78	0,06	0,00	0,31	0,52	6,81	0,41	4,60	0,01	3,52	0,07	0,14	0,15	0,17	0,18
1602		0,80	0,00	0,82	0,01	0,52	0,00	0,93	0,08	1,23	0,08	1,04	0,29	0,74	0,04	1,10	0,42	0,77	0,07	1,01	0,85	0,92	0,12	1,30	0,06	1,34	0,01	1,74	0,00	1,50	0,02
1603		0,00	0,02	0,25	0,07	0,04	0,04	0,01	0,02	22,14	0,32	0,00	0,02	0,00	0,31	0,00	0,23	0,00	0,31	1.000,00	0,29	ð	ð	140,18	0,22	0,00	0,20	0,00	0,22	0,00	0,22
1604	RBM8A	0,68	0,00	0,65	0,00	0,40	0,00	0,70	0,00	0,74	0,04	0,54	0,00	0,59	0,00	0,80	0,00	0,26	0,00	0,93	0,25	0,77	0,04	1,03	0,85	1,09	0,39	1,23	0,22	0,58	0,11
1608		0,73	0,01	0,66	0,00	0,21	0,00	0,66	0,03	1,52	0,13	0,76	0,07	0,64	0,05	0,85	0,42	0,62	0,03	0,82	0,22	0,94	0,67	2,09	0,04	1,40	0,09	3,29	0,00	3,19	0,00
1609		1,01	0,78	1,19	0,01	1,17	0,01	0,98	0,33	0,89	0,00	0,71	0,00	0,89	0,00	0,94	0,02	0,73	0,00	1,13	0,00	0,95	0,03	0,87	0,02	0,84	0,01	0,72	0,00	0,67	0,00
1610	TEBP	0,57	0,01	0,81	0,38	0,46	0,01	0,92	0,02	0,73	0,00	0,63	0,00	0,42	0,01	0,71	0,12	0,35	0,01	1,27	0,00	1,07	0,28	0,70	0,17	1,26	0,26	1,06	0,30	1,09	0,35
1615		0,31	0,09	0,20	0,06	0,05	0,06	0,07	0,01	1,39	0,58	0,06	0,01	0,25	0,00	0,79	0,01	0,15	0,00	0,27	0,08	0,29	0,05	7,87	0,10	1,40	0,17	1,51	0,79	1,08	0,94
1617		0,48	0,16	0,43	0,18	0,23	0,11	0,47	0,01	0,76	0,19	0,23	0,00	0,63	0,17	0,72	0,20	0,27	0,01	0,92	0,70	1,22	0,54	1,65	0,28	1,56	0,29	0,95	0,94	1,09	0,88
1618	CBX3	0,34	0,00	0,65	0,05	0,14	0,00	0,03	0,12	0,73	0,54	0,36	0,19	4,54	0,22	0,73	0,00	0,17	0,00	0,08	0,06	15,15	0,15	1,02	0,93	1,28	0,24	2,46	0,15	1,43	0,56
1619		1,20	0,00	0,95	0,33	0,69	0,00	1,05	0,03	1,01	0,79	0,83	0,00	1,02	0,59	1,19	0,00	1,03	0,30	0,76	0,00	0,69	0,00	0,91	0,03	1,02	0,55	1,05	0,35	1,23	0,01
1621		0,71	0,00	0,80	0,36	0,96	0,90	0,62	0,00	0,64	0,00	0,42	0,00	0,75	0,00	0,75	0,00	0,68	0,01	0,66	0,00	0,70	0,00	0,61	0,18	0,64	0,21	0,33	0,12	0,48	0,20
1623		0,22	0,00	0,36	0,06	0,11	0,01	0,29	0,12	0,34	0,11	0,08	0,04	0,85	0,19	0,84	0,15	0,52	0,03	4,30	0,24	1,99	0,01	3,05	0,11	1,22	0,73	2,31	0,16	2,37	0,24
1628	SKP1	0,98	0,70	1,10	0,19	0,94	0,40	1,00	0,95	0,80	0,00	0,72	0,00	0,64	0,00	1,00	0,96	0,62	0,00	1,09	0,12	0,73	0,00	0,78	0,01	1,01	0,79	0,82	0,02	0,73	0,03
1630	MTND,SSRD	0,90	0,48	0,93	0,68	0,79	0,37	1,09	0,01	0,82	0,00	0,76	0,00	0,82	0,00	0,78	0,00	0,69	0,00	1,16	0,34	0,88	0,45	0,83	0,34	0,80	0,26	0,92	0,79	0,84	0,60
1631		0,71	0,01	0,95	0,24	0,57	0,00	0,91	0,00	0,91	0,00	0,68	0,00	0,84	0,01	1,00	0,94	0,83	0,00	1,23	0,07	0,99	0,94	0,91	0,00	0,88	0,00	1,15	0,04	1,22	0,01
1632	NDKA,COMD1,SODC	0,94	0,02	1,01	0,64	0,80	0,00	0,98	0,06	0,96	0,03	0,78	0,00	0,91	0,00	1,00	0,68	0,91	0,00	0,90	0,00	0,79	0,00	0,82	0,00	0,81	0,00	0,84	0,00	0,94	0,00
1638	SSRD	0,68	0,00	0,79	0,00	0,52	0,00	0,74	0,00	0,87	0,00	0,57	0,00	0,97	0,24	0,50	0,00	1,01	0,65	1,06	0,40	0,93	0,37	1,07	0,19	0,41	0,00	1,05	0,52	1,26	0,02
1640		1,05	0,92	0,29	0,03	0,41	0,11	1,09	0,54	0,71	0,01	0,70	0,01	0,80	0,25	1,36	0,09	0,75	0,13	0,44	0,19	0,22	0,08	1,01	0,96	1,32	0,29	0,71	0,32	0,52	0,13
1641	COF1	0,59	0,00	0,69	0,01	0,55	0,01	0,60	0,00	0,60	0,00	0,42	0,00	0,63	0,00	0,67	0,00	0,45	0,00	0,96	0,06	1,03	0,14	0,82	0,20	0,93	0,60	0,72	0,18	0,80	0,31
1645	NDKA,BT3L4	0,92	0,02	1,08	0,09	0,85	0,01	0,97	0,05	0,93	0,01	0,81	0,00	0,85	0,05	0,81	0,09	0,78	0,01	1,12	0,00	1,03	0,25	0,91	0,04	0,83	0,10	1,01	0,77	1,02	0,65
1647	MLRM	0,59	0,00	0,78	0,00	0,52	0,00	0,72	0,00	0,70	0,00	0,54	0,00	0,65	0,00	0,70	0,00	0,57	0,00	1,07	0,00	0,96	0,01	0,79	0,00	0,79	0,00	0,91	0,00	0,96	0,08
1648		0,80	0,05	0,75	0,14	0,68	0,10	0,89	0,25	0,60	0,01	0,65	0,01	0,54	0,00	1,08	0,00	0,23	0,00	1,28	0,02	0,68	0,00	0,92	0,70	1,45	0,04	1,11	0,66	0,34	0,04
1650		1,11	0,00	0,98	0,44	0,97	0,10	1,20	0,00	0,70	0,00	0,90	0,01	0,94	0,01	1,09	0,00	0,76	0,00	1,21	0,00	1,03	0,21	0,80	0,00	1,34	0,00	1,05	0,22	0,95	0,13
1651	SODC	0,87	0,00	0,90	0,00	0,75	0,00	1,09	0,00	0,83	0,00	0,84	0,00	0,76	0,00	0,99	0,57	0,66	0,00	1,16	0,00	1,07	0,04	0,86	0,00	1,35	0,00	1,04	0,04	1,09	0,01
1652		1,00	0,93	0,51	0,09	0,16	0,00	0,99	0,54	0,98	0,50	0,85	0,00	1,00	0,96	0,91	0,01	1,01	0,86	0,83	0,00	0,76	0,00	1,62	0,23	1,36	0,47	4,51	0,00	4,84	0,00
1654		0,79	0,22	0,56	0,01	0,54	0,02	0,71	0,08	0,69	0,10	0,77	0,32	0,90	0,56	0,88	0,14	0,74	0,10	0,91	0,57	1,09	0,70	1,26	0,21	1,51	0,00	1,44	0,30	1,32	0,27
1655		1,70	0,02	1,32	0,09	2,03	0,01	1,14	0,26	0,79	0,01	0,86	0,05	0,98	0,74	0,73	0,02	1,06	0,55	0,86	0,37	0,47	0,01	0,76	0,08	0,45	0,00	0,54	0,01	0,42	0,01
1656	IF1AX	0,85	0,02	0,98	0,63	0,78	0,01	1,00	0,96	0,93	0,07	0,84	0,01	0,86	0,01	1,11	0,02	0,85	0,01	1,09	0,42	0,88	0,09	0,87	0,01	0,98	0,70	0,98	0,78	0,95	0,51
1657		1,32	0,62	0,52	0,18	0,53	0,29	0,48	0,33	2,72	0,25	1,52	0,56	0,17	0,00	0,95	0,83	0,20	0,00	0,52	0,36	0,10	0,07	7,33	0,12	1,41	0,33	4,02	0,25	0,29	0,13
1660	SSRD	1,31	0,02	1,37	0,11	1,31	0,45	1,09	0,20	1,12	0,07	0,90	0,29	1,08	0,02	1,23	0,00	1,03	0,58	0,99	0,83	0,89	0,08	0,97	0,84	0,97	0,83	0,82	0,57	0,85	0,62
1663		1,03	0,30	1,07	0,01	1,11	0,00	1,03	0,67	0,90	0,01	0,88	0,01	0,95	0,20	0,87	0,01	0,89	0,02	0,94	0,29	0,62	0,00	0,78	0,00	0,54	0,00	0,74	0,00	0,53	0,00
1664		1,02	0,73	1,17	0,03	1,22	0,02	0,97	0,47	0,92	0,00	0,79	0,00	0,76	0,01	1,13	0,09	0,67	0,00	0,97	0,47	0,84	0,02	0,79	0,00	1,09	0,11	0,66	0,00	0,62	0,00
1665		0,92	0,14	1,11	0,26	1,01	0,95	1,03	0,19	1,01	0,66	0,76	0,00	0,96	0,00	1,13	0,00	0,89	0,00	1,11	0,02	0,93	0,09	0,90	0,21	0,91	0,26	0,75	0,01	0,79	0,01
1666		1,09	0,04	1,25	0,00	0,86	0,07	1,12	0,00	0,87	0,00	0,85	0,00	0,79	0,00	1,08	0,05	0,71	0,00	1,03	0,34	0,86	0,00	0,69	0,00	1,03	0,43	0,99	0,83	0,98	0,72
1669		0,85	0,01	1,10	0,09	0,93	0,38	0,92	0,03	0,86	0,00	0,76	0,00	1,04	0,34	0,94	0,03	0,94	0,03	1,18	0,02	1,08	0,20	0,84	0,01	0,76	0,00	0,89	0,23	0,89	0,18
1672	HSB11	0,88	0,02	1,01	0,88	0,79	0,02	0,91	0,01	0,87	0,00	0,71	0,00	0,79	0,00	0,85	0,01	0,75	0,00	0,93	0,10	0,73	0,00	0,78	0,00	0,69	0,00	0,82	0,06	0,78	0,03
1676		1,04	0,90	1,39	0,38	1,70	0,10	0,22	0,33	0,50	0,54	0,39	0,44	0,51	0,00	0,95	0,52	0,84	0,58	0,87	0,43	0,71	0,12	1,51	0,33	1,00	1,00	0,95	0,82	0,72	0,37
1677		1,36	0,10	2,85	0,01	4,36	0,01	1,52	0,19	2,01	0,03	3,25	0,00	0,87	0,39	2,20	0,00	3,25	0,01	0,94	0,58	0,53	0,00	0,59	0,06	0,64	0,09	0,62	0,12	0,62	0,17
1683		5,42	0,02	4,15	0,09	3,75	0,03	0,82	0,70	2,22	0,04	5,64	0,00	0,39	0,35	1,61	0,37	3,70	0,15	0,15	0,02	0,06	0,01	0,53	0,26	0,30	0,11	1,50	0,23	0,78	0,63
1684	STMN1,PTN1	0,96	0,06	1,38	0,00	1,02	0,43	0,95	0,01	0,97	0,04	0,86	0,00	0,78	0,00	1,01	0,65	0,93	0,00	0,93	0,01	0,71	0,00	0,66	0,00	0,63	0,00	0,78	0,00	0,79	0,00
1686	STMN1	1,02	0,47	1,56	0,00	1,14	0,21	1,04	0,05	1,16	0,00	0,94	0,01	0,93	0,01	1,27	0,00	1,13	0,01	0,93	0,01	0,71	0,00	0,68	0,00	0,63	0,00	0,76	0,03	0,77	0,04
1689	STMN1	0,64	0,00	0,64	0,00	0,48	0,00	0,82	0,00	0,58	0,00	0,54	0,00	0,66	0,00	0,73	0,00	0,44	0,00	1,33	0,00	1,27	0,00	0,93	0,02	1,39	0,00	1,16	0,00	1,12	0,00
1692		0,97	0,51	0,52	0,12	0,04	0,00	0,94	0,09	0,95	0,19	0,73	0,00	0,87	0,00	0,97	0,01	0,83	0,01	0,97	0,57	0,85	0,02	1,81	0,16	1,74	0,19	17,32	0,00	18,63	0,00
1693	TXD12,STMN1	0,96	0,51	1,30	0,01	0,98	0,88	0,99	0,84	1,03	0,51	0,87	0,01	0,80	0,00	1,24	0,00	1,00	0,98	0,95	0,19	0,70	0,00	0,72	0,00	0,80	0,01	0,82	0,20	0,85	0,33
1695	STMN1	0,89	0,00	0,79	0,01	0,65	0,00	1,06	0,00	0,65	0,00	0,73	0,00	0,78	0,00	0,92	0,04	0,56	0,00	1,25	0,00	1,13	0,00	0,86	0,06	1,50	0,00	1,19	0,01	1,13	0,03
1697	IF5A1	0,74	0,00	0,86	0,00	0,54	0,00	0,83	0,00	0,86	0,00	0,62	0,00	0,82	0,00	0,87	0,00	0,79	0,00	1,01	0,71	0,91	0,00	0,90	0,00	0,83	0,00	1,03	0,17	1,21	0,00
1700		2,69	0,00	2,34	0,01	3,18	0,00	1,47	0,00	1,48	0,00	1,70	0,00	1,20	0,01	1,52	0,00	1,86	0,00	0,63	0,00	0,43	0,00	0,73	0,08	0,63	0,03	0,61	0,01	0,56	0,00
1701	STMN1,1433S	0,96	0,07	0,94	0,00	0,77	0,00	1,00	0,86	0,86	0,00	0,74	0,00	1,00	0,87	0,95	0,03	0,88	0,00	1,00	0,93	0,93	0,01	0,88	0,00	0,90	0,00	0,92	0,02	1,01	0,46
1702	CALM	1,03	0,04	1,22	0,00	1,01	0,50	1,05	0,01	0,91	0,00	0,84	0,00	0,94	0,34	1,30	0,00	0,85	0,00	1,02	0,17	0,94	0,29	0,75	0,00	1,10	0,00	0,83	0,00	0,87	0,00
1703	HSB11	1,08	0,05	1,15	0,05	0,83	0,05	1,02	0,34	0,99	0,48	0,80	0,00	0,84	0,00	1,08	0,01	0,91	0,01	0,87	0,00	0,68	0,00	0,80	0,01	0,83	0,02	0,89	0,21	0,97	0,69
1704		0,73	0,35	0,44	0,04	0,11	0,02	0,74	0,04	1,06	0,61	0,75	0,05	0,57	0,01	0,70	0,04	0,46	0,00	0,80	0,42	0,82	0,46	1,90	0,00	1,64	0,01	5,43	0,00	4,40	0,00
1705		1,30	0,03	1,04	0,58	0,91	0,26	0,97	0,52	1,31	0,00	1,31	0,00	0,80	0,00	0,75	0,00	0,91	0,02	0,73	0,02	0,43	0,00	1,24	0,01	0,51	0,00	1,42	0,01	0,71	0,01
1706	MRLC2	0,96	0,24	0,97	0,56	0,96	0,52	1,10	0,04	0,92	0,01	0,93	0,02	0,91	0,03	1,16	0,00	0,83	0,00	1,05	0,26	0,94	0,09	0,87	0,02	1,19	0,01	0,88	0,07	0,86	0,05
1707		0,75	0,34	0,37	0,03	0,03	0,01	0,68	0,02	0,90	0,19	0,66	0,01	0,33	0,06	0,63	0,28	0,19	0,02	0,83	0,54	0,90	0,81	2,25	0,01	3,46	0,06	23,94	0,00	15,13	0,00
1709		0,73	0,00	0,80	0,00	0,67	0,00	0,84	0,00	0,78	0,00	0,62	0,00	0,73	0,00	0,84	0,00	0,63	0,00	1,12	0,01	1,00	0,87	0,95	0,09	1,05	0,13	0,90	0,03	0,93	0,08
1712		0,97	0,23	1,04	0,33	0,83	0,00	1,01	0,75	0,98	0,30	0,79	0,00	0,96	0,47	1,09	0,04	1,00	1,00	1,03	0,45	0,80	0,00	0,92	0,08	0,85	0,01	0,93	0,11	0,97	0,67
1716		0,63	0,01	0,70	0,02	0,51	0,01	0,65	0,08	0,99	0,96	0,66	0,04	0,73	0,04	0,84	0,12	0,93	0,60	1,11	0,65	1,44	0,05	1,53	0,02	1,47	0,02	1,39	0,07	2,25	0,01
1717		0,53	0,01	0,91	0,68	0,33	0,02	0,50	0,00	1,13	0,29	0,41	0,00	0,57	0,00	0,47	0,00	0,56	0,00	0,79	0,17	0,69	0,08	1,03	0,89	0,33	0,02	1,03	0,95	1,10	0,84
1719		0,54	0,00	1,10	0,26	0,72	0,08	0,84	0,43	1,46	0,06	0,99	0,94	0,87	0,41	1,27	0,18	0,60	0,10	1,37	0,04	2,23	0,02	1,17	0,07	1,60	0,04	1,22	0,27	1,15	0,77
1720		0,16	0,02	0,23	0,02	0,19	0,04	0,60	0,52	0,26	0,22	0,02	0,12	0,00	0,19	0,01	0,19	0,02	0,19	12,74	0,08	0,29	0,31	3,67	0,01	0,74	0,73	0,30	0,03	2,20	0,56
1721		0,69	0,28	0,89	0,80	0,61	0,26	0,39	0,26	1,78	0,26	1,35	0,55	2,38	0,29	2,42	0,16	2,23	0,14	0,50	0,06	1,29	0,65	1,77	0,21	1,01	0,98	1,96	0,11	1,35	0,41
1723	CT077	0,62	0,01	1,32	0,70	0,30	0,02	0,55	0,16	0,68	0,26	0,59	0,17	1,06	0,79	0,88	0,53	0,73	0,22	1,10	0,71	1,68	0,08	0,64	0,57	0,65	0,58	2,45	0,10	2,40	0,15
1730		0,74	0,04	1,02	0,84	0,69	0,01	0,98	0,58	0,91	0,03	0,74	0,00	0,67	0,00	0,85	0,08	0,57	0,00	1,52	0,01	1,38	0,04	1,03	0,78	1,27	0,04	1,23	0,12	1,27	0,11
1732		0,99	0,86	1,10	0,35	1,27	0,21	1,26	0,19	1,39	0,07	1,76	0,20	0,81	0,14	1,29	0,08	1,22	0,16	0,98	0,87	0,59	0,00	0,97	0,81	0,84	0,11	1,06	0,88	0,69	0,08
1735		1,04	0,13	0,99	0,76	0,75	0,00	1,10	0,01	0,91	0,00	0,80	0,00	1,12	0,00	1,06	0,02	0,96	0,06	1,14	0,00	1,03	0,24	1,00	0,95	1,03	0,53	1,16	0,04	1,23	0,01
1736		1,04	0,33	1,02	0,85	0,87	0,32	1,22	0,00	0,91	0,02	0,91	0,08	1,29	0,00	1,18	0,00	1,06	0,03	1,17	0,01	1,20	0,00	0,89	0,32	1,12	0,30	1,04	0,77	1,18	0,24
1739		0,52	0,01	1,04	0,93	0,28	0,01	1,72	0,17	0,97	0,88	0,73	0,13	0,69	0,10	0,82	0,49	0,67	0,42	2,22	0,09	0,94	0,81	0,63	0,43	0,55	0,37	1,76	0,29	1,70	0,61
1740	MYL6,RLA2	1,00	0,62	1,08	0,01	0,90	0,00	1,02	0,40	0,93	0,01	0,79	0,00	0,88	0,00	0,98	0,02	0,85	0,00	0,92	0,00	0,76	0,00	0,78	0,00	0,78	0,00	0,79	0,00	0,82	0,00
1746	PFD5	0,74	0,00	0,98	0,88	0,79	0,27	0,98	0,55	0,84	0,00	0,69	0,00	0,85	0,00	0,88	0,00	0,71	0,00	1,22	0,02	0,98	0,77	0,78	0,13	0,76	0,09	0,80	0,37	0,75	0,28
1750		0,79	0,14	1,00	0,99	0,88	0,40	0,77	0,00	0,73	0,00	0,57	0,00	0,67	0,00	0,68	0,00	0,56	0,00	1,08	0,53	0,95	0,68	0,80	0,05	0,76	0,03	0,71	0,03	0,71	0,03
1755	MYL6	0,84	0,00	0,96	0,05	0,74	0,00	0,86	0,00	0,76	0,00	0,64	0,00	0,70	0,00	0,80	0,00	0,67	0,00	0,94	0,01	0,79	0,00	0,74	0,00	0,79	0,00	0,81	0,00	0,86	0,01
1758		0,85	0,00	0,84	0,00	0,68	0,00	0,97	0,38	0,79	0,00	0,69	0,00	0,83	0,00	0,92	0,00	0,75	0,01	1,04	0,09	0,87	0,00	0,86	0,00	0,97	0,22	0,92	0,06	0,97	0,78
1759		1,02	0,79	1,05	0,71	0,83	0,26	1,17	0,01	0,89	0,00	0,82	0,00	0,86	0,01	0,96	0,20	0,83	0,00	1,00	0,98	0,78	0,01	0,73	0,07	0,84	0,25	0,86	0,42	0,92	0,65
1761		1,40	0,00	1,65	0,00	2,17	0,00	1,40	0,00	0,96	0,27	1,27	0,00	0,64	0,00	1,18	0,11	0,57	0,00	1,09	0,12	1,11	0,27	0,63	0,00	1,73	0,00	0,63	0,00	0,64	0,00
1762		0,83	0,03	1,04	0,74	0,59	0,00	0,97	0,66	0,90	0,21	0,65	0,00	0,67	0,00	0,87	0,04	0,66	0,00	1,02	0,80	0,67	0,00	0,77	0,05	0,70	0,02	0,97	0,82	0,93	0,59
1769	COTL1	0,92	0,00	1,09	0,06	0,79	0,00	1,04	0,08	1,06	0,01	0,77	0,00	0,88	0,04	1,05	0,00	0,94	0,03	0,98	0,08	0,82	0,00	0,84	0,00	0,82	0,00	0,84	0,00	1,01	0,71
1772		1,10	0,00	1,21	0,01	0,96	0,58	1,21	0,00	1,07	0,17	0,90	0,05	1,03	0,39	1,14	0,00	0,96	0,52	0,96	0,07	0,77	0,00	0,76	0,00	0,78	0,00	0,82	0,06	0,83	0,14
1776	OAT	0,95	0,21	0,75	0,30	0,69	0,35	1,06	0,33	1,07	0,24	0,79	0,01	0,82	0,73	1,20	0,70	0,76	0,64	0,92	0,18	0,79	0,00	1,18	0,57	1,46	0,19	0,94	0,90	0,99	0,99
1780	TBCA	0,98	0,39	0,98	0,64	0,86	0,05	1,08	0,01	0,86	0,00	0,85	0,00	0,92	0,43	1,09	0,00	0,86	0,00	1,06	0,01	0,91	0,36	0,85	0,02	1,09	0,12	0,95	0,42	0,98	0,70
1782		1,26	0,02	1,37	0,00	1,64	0,00	0,86	0,35	0,48	0,01	0,54	0,01	0,70	0,03	1,01	0,98	1,43	0,64	1,38	0,01	0,95	0,59	0,71	0,00	1,27	0,19	0,66	0,00	1,52	0,66
1784	PEA15	0,80	0,00	0,83	0,01	0,56	0,00	0,82	0,00	0,69	0,00	0,59	0,00	0,65	0,00	0,74	0,00	0,60	0,00	1,02	0,58	0,80	0,04	0,82	0,01	0,88	0,07	1,03	0,42	1,06	0,14
1795		1,14	0,00	1,19	0,00	1,23	0,00	1,01	0,93	0,68	0,02	0,71	0,03	0,61	0,01	1,21	0,38	0,94	0,85	1,16	0,03	0,85	0,04	0,75	0,00	1,62	0,06	0,76	0,00	1,21	0,67
1802	PROF2,TR112	0,76	0,00	0,90	0,10	0,64	0,00	0,80	0,00	0,66	0,00	0,53	0,00	1,01	0,97	0,84	0,00	0,95	0,83	1,42	0,00	1,39	0,28	0,98	0,83	0,97	0,59	1,12	0,41	1,55	0,22
1820		1,00	0,99	0,57	0,15	0,70	0,39	0,97	0,85	0,90	0,61	1,01	0,97	2,39	0,01	1,12	0,00	1,88	0,00	1,05	0,71	1,32	0,23	1,71	0,03	1,09	0,58	1,56	0,10	1,48	0,07
1821	THIO,RFA3,LEG1,COX5A	1,39	0,00	1,50	0,00	1,69	0,00	1,19	0,08	0,86	0,18	0,97	0,78	1,75	0,10	1,21	0,00	2,08	0,01	1,22	0,00	1,36	0,28	0,81	0,01	0,87	0,01	0,82	0,04	1,33	0,14
1954		1,26	0,03	1,29	0,27	1,40	0,02	N,A,	N,A,	N,A,	N,A,	N,A,	N,A,	0,00	0,41	0,49	0,64	0,01	0,41	0,00	N,A,	1,75	0,14	0,00	N,A,	200,82	0,32	0,00	N,A,	2,25	0,19
2111	GLU2B	1,16	0,00	1,30	0,00	1,33	0,00	1,24	0,00	1,13	0,00	1,14	0,00	0,86	0,00	1,20	0,00	1,03	0,31	0,94	0,04	0,73	0,00	0,77	0,00	0,91	0,00	0,75	0,00	0,76	0,00
2174	CALR	1,16	0,00	1,37	0,00	1,46	0,00	1,22	0,00	1,04	0,03	1,18	0,00	0,94	0,00	1,18	0,00	0,93	0,00	1,11	0,00	0,99	0,71	0,81	0,00	1,07	0,03	0,85	0,00	0,78	0,00
2183		1,61	0,00	2,12	0,00	2,55	0,00	1,52	0,00	1,99	0,00	2,76	0,00	1,73	0,00	2,02	0,00	2,17	0,00	0,82	0,00	0,90	0,00	0,82	0,00	0,80	0,00	0,94	0,10	0,72	0,00
2200	TBA3	1,66	0,00	2,22	0,00	2,82	0,00	1,50	0,00	2,11	0,00	2,96	0,00	1,77	0,00	2,07	0,00	2,25	0,00	0,80	0,00	0,92	0,00	0,84	0,00	0,81	0,00	0,93	0,15	0,69	0,00
2214		0,68	0,00	0,78	0,05	0,48	0,00	0,86	0,08	1,05	0,58	0,69	0,00	0,53	0,00	0,80	0,00	0,57	0,00	0,77	0,01	0,59	0,00	0,82	0,16	0,77	0,06	0,87	0,05	0,89	0,15
2239	PDIA1	1,57	0,00	2,02	0,00	3,04	0,00	1,36	0,00	1,15	0,00	1,40	0,00	1,05	0,15	1,38	0,00	1,15	0,00	0,89	0,00	0,79	0,00	0,58	0,00	0,81	0,00	0,47	0,00	0,45	0,00
2286		1,02	0,30	1,08	0,05	0,86	0,00	1,10	0,00	0,93	0,07	0,82	0,00	0,92	0,04	1,01	0,48	0,87	0,07	1,00	0,97	0,80	0,00	0,80	0,00	0,83	0,00	0,88	0,02	0,89	0,20
2294		1,01	0,80	1,02	0,57	0,65	0,00	1,07	0,02	0,98	0,28	0,79	0,00	0,86	0,00	1,06	0,01	0,92	0,00	0,94	0,07	0,72	0,00	0,85	0,00	0,87	0,01	1,07	0,17	1,19	0,00
2310	TEBP	0,74	0,00	0,79	0,00	0,44	0,00	0,93	0,01	0,79	0,00	0,67	0,00	0,56	0,07	0,52	0,06	0,44	0,03	1,12	0,02	0,95	0,20	0,89	0,01	0,83	0,03	1,35	0,00	1,27	0,00

2330		2,01	0,00	0,95	0,84	1,49	0,55	1,61	0,01	1,27	0,15	1,42	0,06	0,79	0,45	1,36	0,04	1,48	0,02	0,57	0,00	0,41	0,01	0,96	0,87	1,51	0,08	0,68	0,57	1,05	0,93
2359		0,63	0,00	0,60	0,00	0,19	0,00	0,99	0,97	0,92	0,68	0,75	0,22	2,24	0,00	2,37	0,00	1,83	0,04	1,47	0,01	2,19	0,00	1,44	0,01	2,45	0,00	3,76	0,00	6,02	0,01
2379		1,78	0,02	0,45	0,08	0,28	0,06	1,58	0,09	0,62	0,19	1,01	0,97	4,09	0,00	2,93	0,00	2,41	0,01	1,00	0,97	1,47	0,02	1,54	0,16	4,15	0,00	4,05	0,00	5,51	0,00
2437		0,76	0,03	0,89	0,33	0,50	0,01	0,65	0,00	0,97	0,73	0,62	0,00	0,61	0,05	1,21	0,30	0,60	0,05	0,86	0,13	0,86	0,16	1,09	0,42	1,44	0,03	1,24	0,15	1,28	0,27
2522		1,35	0,00	1,74	0,00	2,05	0,00	1,01	0,87	0,99	0,68	1,01	0,78	0,84	0,00	0,85	0,00	1,03	0,73	0,70	0,00	0,52	0,00	0,54	0,00	0,41	0,00	0,47	0,00	0,42	0,00
2575		1,04	0,55	0,92	0,32	0,93	0,45	1,19	0,00	0,77	0,00	0,84	0,00	0,97	0,52	1,28	0,00	0,82	0,00	1,16	0,03	0,88	0,07	0,85	0,02	1,31	0,00	0,93	0,37	0,84	0,08
2723		0,97	0,31	1,03	0,24	0,78	0,00	1,04	0,03	0,85	0,00	0,77	0,00	0,93	0,00	1,04	0,09	0,85	0,00	1,01	0,69	0,91	0,00	0,78	0,00	0,95	0,01	0,92	0,00	1,03	0,05
2753		0,67	0,01	0,84	0,14	0,68	0,06	1,22	0,01	0,93	0,34	0,92	0,28	1,22	0,06	1,60	0,00	0,97	0,79	1,84	0,00	2,08	0,00	1,11	0,23	2,18	0,00	1,37	0,06	1,63	0,03
2998	TBB2C,TFG	1,02	0,24	1,09	0,05	0,98	0,30	0,98	0,29	1,01	0,68	0,96	0,14	0,77	0,00	0,99	0,39	0,84	0,00	0,86	0,00	0,78	0,00	0,82	0,00	0,94	0,13	0,87	0,01	0,89	0,01
3104	TBB2C,TBB6,TBB2A,SMCE1	0,92	0,00	0,86	0,00	0,65	0,00	0,86	0,00	0,86	0,00	0,80	0,00	0,73	0,00	0,90	0,00	0,75	0,00	0,88	0,00	0,81	0,00	0,93	0,07	1,05	0,15	1,15	0,01	1,16	0,00
3111	TBB2C,TBB6,TBB2A,SMCE1	0,93	0,00	0,86	0,00	0,68	0,00	0,94	0,01	0,83	0,00	0,74	0,00	0,80	0,00	0,97	0,17	0,79	0,00	0,98	0,29	0,88	0,00	0,94	0,14	1,16	0,01	1,06	0,10	1,21	0,00
3150	TBB3,TBB2C	0,96	0,00	1,01	0,87	0,83	0,00	1,02	0,30	0,91	0,00	0,83	0,00	0,78	0,00	0,97	0,01	0,75	0,00	0,94	0,00	0,72	0,00	0,80	0,00	0,85	0,00	0,88	0,01	0,79	0,00
3179		2,16	0,00	2,67	0,00	3,50	0,00	1,62	0,00	2,05	0,00	3,16	0,00	1,99	0,00	2,41	0,00	2,72	0,00	0,76	0,00	0,86	0,00	0,77	0,00	0,84	0,00	0,91	0,02	0,72	0,00
3227	NUCB1,VIME	1,42	0,00	1,78	0,00	2,14	0,00	1,27	0,00	1,43	0,00	1,75	0,00	1,35	0,00	1,50	0,00	1,52	0,00	0,90	0,00	0,90	0,00	0,81	0,00	0,80	0,00	0,82	0,00	0,67	0,00
3316	MCM6,2AAA	2,26	0,00	2,79	0,00	3,86	0,00	1,79	0,00	2,34	0,00	3,56	0,00	2,09	0,00	2,33	0,00	2,66	0,00	0,77	0,00	0,89	0,00	0,82	0,00	0,80	0,00	0,90	0,00	0,66	0,00
3406	IMA7	1,00	0,94	1,14	0,04	1,42	0,00	0,89	0,29	0,93	0,43	1,16	0,16	0,89	0,25	1,01	0,96	1,05	0,59	0,90	0,16	0,89	0,05	0,83	0,00	0,88	0,07	0,83	0,03	0,74	0,00
3542	UBA5	0,69	0,00	0,48	0,00	0,33	0,00	0,88	0,05	0,72	0,00	0,50	0,00	0,82	0,00	0,79	0,01	0,56	0,00	1,18	0,07	0,97	0,64	1,39	0,00	1,35	0,02	1,43	0,00	1,39	0,01
3616		1,06	0,22	1,27	0,00	1,38	0,00	1,05	0,10	1,00	0,99	1,10	0,02	0,93	0,01	1,01	0,25	0,93	0,01	1,04	0,41	0,98	0,65	0,83	0,00	0,89	0,01	0,84	0,00	0,75	0,00
3670	KAP2,IF4A1	0,83	0,12	0,56	0,00	0,48	0,00	0,92	0,10	0,66	0,00	0,57	0,00	0,83	0,00	0,83	0,00	0,65	0,00	1,15	0,26	0,93	0,57	1,21	0,03	1,37	0,00	1,24	0,11	1,26	0,16
3737	ATPB	1,41	0,00	1,33	0,01	1,62	0,00	1,30	0,00	0,90	0,05	1,02	0,70	1,00	0,92	1,30	0,00	0,96	0,30	0,92	0,01	0,74	0,00	0,68	0,00	1,02	0,79	0,63	0,00	0,61	0,00
3760	GRP78	2,45	0,00	3,63	0,00	5,67	0,00	2,16	0,00	2,03	0,00	2,84	0,00	1,47	0,01	3,16	0,00	2,74	0,00	0,74	0,00	0,64	0,00	0,47	0,00	0,93	0,16	0,42	0,00	0,52	0,00
3841	HS90A	1,21	0,00	1,31	0,00	1,09	0,05	1,15	0,00	0,97	0,37	0,99	0,79	0,69	0,01	1,02	0,89	0,78	0,03	0,94	0,11	0,68	0,00	0,73	0,00	0,92	0,37	0,90	0,03	0,85	0,00
3931	HS90A	1,07	0,20	1,01	0,83	0,86	0,03	1,11	0,02	1,10	0,04	1,09	0,06	1,02	0,65	1,08	0,03	1,01	0,74	0,94	0,18	0,79	0,00	0,99	0,79	0,89	0,01	1,14	0,01	0,97	0,12
4106	HS90B,HS90A	0,96	0,65	0,72	0,01	0,97	0,63	0,92	0,01	1,01	0,63	0,88	0,00	1,33	0,00	0,83	0,12	1,29	0,01	1,30	0,01	1,40	0,00	1,91	0,00	1,15	0,28	1,22	0,00	1,33	0,00
4295	P3H1	0,94	0,09	0,99	0,72	0,98	0,47	0,92	0,02	0,82	0,00	0,68	0,00	0,81	0,00	0,99	0,29	0,73	0,00	0,94	0,11	0,77	0,00	0,80	0,00	0,90	0,03	0,67	0,00	0,67	0,00
4476		0,71	0,00	0,76	0,00	0,56	0,00	0,80	0,00	0,73	0,00	0,59	0,00	0,68	0,00	0,81	0,00	0,64	0,00	1,05	0,09	0,84	0,00	0,89	0,02	0,93	0,04	0,99	0,68	1,00	0,99
4489		0,99	0,75	0,94	0,27	0,76	0,00	0,94	0,31	0,61	0,00	0,66	0,00	0,66	0,00	0,88	0,00	0,66	0,00	0,99	0,89	0,79	0,00	0,68	0,00	1,11	0,07	0,91	0,20	1,03	0,57
4596	GANAB	0,67	0,00	0,74	0,00	0,49	0,00	0,73	0,01	0,63	0,00	0,54	0,00	0,54	0,00	0,76	0,00	0,56	0,00	1,01	0,81	0,87	0,03	0,79	0,01	1,09	0,16	1,02	0,78	1,23	0,02
4694		0,87	0,01	0,89	0,01	0,70	0,00	1,04	0,35	0,88	0,03	0,87	0,02	0,82	0,00	1,03	0,33	0,79	0,00	1,13	0,01	0,97	0,40	0,93	0,06	1,19	0,00	1,17	0,02	1,16	0,02
4808		0,92	0,06	1,00	0,92	0,88	0,02	0,99	0,75	1,00	0,97	0,97	0,44	0,85	0,00	1,09	0,03	0,90	0,02	0,96	0,17	0,83	0,00	0,90	0,01	0,99	0,59	0,98	0,57	0,93	0,00
4923		0,63	0,01	0,57	0,00	0,46	0,00	0,88	0,08	0,72	0,01	0,63	0,00	0,84	0,06	0,82	0,03	0,61	0,00	1,42	0,00	1,24	0,02	1,29	0,01	1,35	0,00	1,41	0,03	1,25	0,04
5273		0,83	0,00	0,87	0,00	0,47	0,00	0,85	0,01	0,93	0,30	0,81	0,00	0,47	0,00	0,91	0,04	0,59	0,00	0,79	0,00	0,64	0,00	0,83	0,02	1,18	0,01	1,34	0,00	1,42	0,00
5403	HYOU1	0,72	0,00	0,76	0,00	0,39	0,00	0,68	0,00	0,60	0,00	0,57	0,00	0,66	0,01	0,84	0,10	0,70	0,01	1,09	0,23	0,95	0,35	0,90	0,06	1,14	0,01	1,67	0,00	1,84	0,00
5526	HYOU1	0,68	0,00	0,62	0,00	0,34	0,00	0,69	0,00	0,60	0,00	0,54	0,00	0,70	0,00	0,81	0,00	0,66	0,00	1,14	0,02	0,97	0,29	1,07	0,31	1,22	0,00	1,74	0,00	1,81	0,00
5884		0,90	0,19	1,20	0,22	0,46	0,00	0,75	0,00	0,84	0,18	0,78	0,05	0,20	0,30	0,23	0,31	0,35	0,39	1,02	0,79	0,80	0,03	0,86	0,39	0,68	0,09	2,05	0,02	2,74	0,01
5909		0,62	0,00	0,56	0,00	0,31	0,00	0,62	0,00	0,44	0,00	0,49	0,00	0,52	0,00	0,66	0,00	0,42	0,00	1,15	0,01	1,19	0,01	0,91	0,04	1,68	0,00	1,83	0,00	1,92	0,00
6662		0,84	0,00	1,15	0,11	1,45	0,00	0,70	0,00	0,87	0,25	0,62	0,00	0,59	0,00	0,76	0,02	0,61	0,00	0,83	0,01	0,68	0,01	0,75	0,06	0,63	0,00	0,42	0,00	0,40	0,00
6678		0,88	0,02	1,22	0,03	1,59	0,00	0,81	0,01	0,88	0,27	0,76	0,02	0,71	0,02	0,88	0,14	0,71	0,00	0,89	0,16	0,74	0,02	0,70	0,02	0,66	0,00	0,47	0,00	0,41	0,00
7106		1,55	0,00	1,73	0,00	2,28	0,00	1,30	0,00	1,17	0,00	1,34	0,00	0,64	0,00	0,90	0,18	0,73	0,01	0,85	0,00	0,72	0,00	0,68	0,00	0,89	0,05	0,60	0,00	0,55	0,00
7425		1,42	0,00	1,74	0,00	1,95	0,00	1,41	0,00	1,69	0,00	2,10	0,00	0,88	0,05	1,37	0,00	1,11	0,05	0,80	0,00	0,73	0,00	0,79	0,00	0,94	0,05	0,88	0,03	0,68	0,00
7589		1,67	0,00	1,97	0,00	2,25	0,00	1,35	0,00	1,50	0,00	1,77	0,00	0,86	0,03	1,30	0,00	0,99	0,80	0,84	0,00	0,77	0,00	0,79	0,00	0,99	0,62	0,81	0,02	0,66	0,00
7726		0,77	0,00	0,77	0,00	0,64	0,00	0,87	0,00	0,76	0,00	0,65	0,00	0,65	0,00	0,90	0,05	0,68	0,00	1,15	0,00	0,82	0,00	1,00	0,94	1,14	0,06	1,02	0,43	1,02	0,41
7894	MVP,TIF1B,MVP	0,85	0,00	0,75	0,00	0,54	0,00	0,87	0,00	0,70	0,00	0,68	0,00	0,73	0,00	0,84	0,00	0,65	0,00	1,08	0,00	1,02	0,22	0,97	0,33	1,33	0,00	1,32	0,00	1,43	0,00
8068		0,78	0,00	0,82	0,00	0,58	0,00	0,81	0,00	0,80	0,00	0,62	0,00	0,69	0,00	0,83	0,00	0,71	0,00	0,90	0,01	0,75	0,00	0,85	0,00	0,86	0,00	0,94	0,09	1,05	0,08
8247	ACTN4	0,59	0,00	0,53	0,00	0,35	0,00	0,71	0,00	0,57	0,00	0,49	0,00	0,71	0,00	0,80	0,00	0,56	0,00	1,30	0,00	1,12	0,00	1,17	0,00	1,42	0,00	1,52	0,00	1,50	0,00
8429		0,86	0,00	0,88	0,02	0,68	0,00	0,94	0,03	0,82	0,00	0,72	0,00	0,74	0,00	0,94	0,20	0,73	0,00	1,11	0,00	0,90	0,00	0,94	0,22	1,12	0,06	1,06	0,03	1,11	0,01
8503		0,80	0,00	0,75	0,00	0,56	0,00	0,79	0,00	0,75	0,00	0,65	0,00	0,61	0,00	0,82	0,00	0,62	0,00	0,93	0,01	0,84	0,00	0,93	0,17	1,19	0,01	1,09	0,02	1,21	0,00
8700	ACTN4,MVP	0,41	0,00	0,56	0,00	0,41	0,00	1,06	0,18	0,71	0,00	0,70	0,00	0,69	0,02	1,02	0,87	0,57	0,00	2,22	0,00	2,64	0,00	1,11	0,48	2,91	0,00	1,48	0,00	2,24	0,00
9000		0,60	0,00	0,64	0,00	0,44	0,00	1,05	0,66	0,87	0,25	0,86	0,19	0,96	0,73	1,17	0,19	0,89	0,33	1,35	0,01	1,81	0,00	1,05	0,33	2,04	0,00	1,49	0,00	2,25	0,00
9044		1,37	0,00	1,60	0,00	1,92	0,00	1,25	0,00	1,42	0,00	1,78	0,00	1,00	0,93	1,40	0,00	1,11	0,04	0,90	0,00	0,84	0,00	0,87	0,00	1,01	0,77	0,91	0,00	0,66	0,00
9232		1,03	0,19	1,16	0,00	1,42	0,00	0,90	0,00	0,77	0,00	0,84	0,00	0,90	0,07	1,02	0,10	0,79	0,00	0,98	0,30	0,89	0,04	0,73	0,00	0,89	0,00	0,66	0,00	0,56	0,00
9421	SF3B2	1,22	0,01	1,53	0,00	1,93	0,00	1,05	0,05	0,95	0,07	0,99	0,68	0,81	0,00	0,94	0,25	0,73	0,00	1,05	0,33	0,97	0,54	0,76	0,00	0,90	0,05	0,62	0,00	0,55	0,00
9875	TERA	0,70	0,00	0,67	0,00	0,45	0,00	0,78	0,00	0,64	0,00	0,57	0,00	0,73	0,00	0,86	0,00	0,66	0,00	1,09	0,00	0,97	0,21	0,93	0,03	1,19	0,00	1,22	0,00	1,34	0,00
10099	HOOK3,HS90A	1,11	0,02	1,27	0,01	1,31	0,02	1,61	0,00	1,59	0,01	1,37	0,00	1,14	0,10	1,20	0,04	1,26	0,00	1,49	0,00	1,40	0,01	1,29	0,05	1,29	0,02	1,08	0,33	1,31	0,01
10303		0,97	0,17	0,98	0,52	0,93	0,19	1,01	0,92	1,01	0,89	0,92	0,15	0,87	0,01	0,98	0,37	0,93	0,04	1,02	0,70	0,84	0,00	1,01	0,89	0,93	0,08	0,96	0,50	0,93	0,24
10509	LG3BP	1,45	0,00	1,65	0,00	1,80	0,00	1,29	0,00	1,27	0,01	1,28	0,00	0,90	0,02	1,26	0,00	1,01	0,67	0,94	0,10	0,74	0,00	0,81	0,01	0,92	0,01	0,75	0,00	0,67	0,00
10719	GRP78	1,27	0,00	1,46	0,00	2,18	0,00	0,98	0,66	0,90	0,09	0,88	0,03	1,57	0,00	1,80	0,00	1,31	0,00	1,28	0,00	1,16	0,02	1,02	0,73	1,16	0,05	0,67	0,00	0,57	0,00
10923	HS90A	0,65	0,00	0,77	0,01	0,58	0,00	0,81	0,00	0,67	0,00	0,55	0,00	0,72	0,00	0,91	0,02	0,75	0,00	1,05	0,17	0,84	0,00	0,73	0,01	0,89	0,20	0,81	0,00	0,97	0,25
11367		0,88	0,06	1,01	0,90	0,66	0,00	0,78	0,00	0,71	0,00	0,59	0,00	0,66	0,00	0,82	0,00	0,54	0,00	1,02	0,62	0,93	0,14	0,82	0,01	1,00	0,95	1,03	0,51	1,00	0,91
11611		1,21	0,37	1,12	0,58	0,69	0,26	0,53	0,00	0,57	0,00	0,45	0,00	0,33	0,00	0,41	0,00	0,17	0,00	0,74	0,02	0,80	0,04	0,86	0,23	1,10	0,43	1,11	0,58	0,75	0,03
11837		1,00	0,99	1,11	0,42	1,07	0,62	1,04	0,39	1,01	0,83	1,02	0,68	0,72	0,00	0,97	0,45	0,60	0,00	0,96	0,69	0,96	0,64	0,84	0,06	1,15	0,06	0,88	0,03	0,74	0,00
12067	GRP78	1,17	0,25	1,04	0,78	1,03	0,82	1,06	0,37	1,00	0,94	1,08	0,30	0,66	0,00	1,11	0,03	0,61	0,00	0,72	0,02	0,66	0,01	0,76	0,05	1,24	0,05	0,83	0,04	0,69	0,00
12547	HDAC1,HNRPK,CH60	0,87	0,00	0,90	0,00	0,81	0,00	0,94	0,02	0,89	0,02	0,87	0,00	0,98	0,30	1,08	0,01	0,92	0,16	1,02	0,26	0,96	0,09	0,94	0,12	1,02	0,33	1,02	0,07	0,96	0,56
12822		0,93	0,30	0,72	0,00	0,63	0,00	1,07	0,02	1,51	0,00	1,06	0,01	1,11	0,27	1,60	0,00	1,29	0,04	0,90	0,05	0,83	0,01	1,65	0,00	1,55	0,00	1,32	0,00	1,43	0,00
13083	TXND5,SH3G1	0,77	0,00	0,80	0,00	0,57	0,00	0,86	0,00	0,58	0,00	0,56	0,00	0,71	0,00	0,82	0,00	0,59	0,00	1,21	0,00	0,99	0,75	0,80	0,00	1,11	0,05	1,08	0,06	1,12	0,01
13335	DPOD2	0,66	0,00	0,69	0,00	0,40	0,00	0,69	0,00	0,71	0,00	0,50	0,00	0,68	0,00	0,65	0,00	0,46	0,00	1,28	0,01	1,40	0,00	1,26	0,00	1,26	0,00	1,55	0,03	1,57	0,02
13616	TPP1,HNRPF	0,89	0,00	0,93	0,02	0,76	0,00	0,94	0,03	0,75	0,00	0,72	0,00	0,80	0,01	0,90	0,11	0,65	0,00	0,94	0,01	0,84	0,00	0,72	0,00	0,90	0,02	0,85	0,00	0,80	0,00
13655	IF4A1,NDRG3,RBBP7,PDIA6,VIME	1,00	0,97	1,00	0,95	0,72	0,00	0,87	0,00	0,80	0,00	0,70	0,00	0,61	0,00	0,90	0,02	0,58	0,00	0,90	0,02	0,82	0,00	0,83	0,00	1,20	0,00	1,00	0,98	1,08	0,04
13892	ACL6A,IF4A1,HNRPF	0,81	0,00	0,86	0,00	0,69	0,00	0,97	0,27	0,87	0,01	0,75	0,00	0,99	0,59	1,08	0,04	0,71	0,00	1,17	0,00	1,15	0,00	0,98	0,52	1,18	0,00	1,07	0,12	0,98	0,11
14100	CH60	1,17	0,00	1,11	0,03	0,95	0,25	1,12	0,00	1,00	0,92	0,98	0,24	0,96	0,17	1,17	0,00	0,93	0,05	0,88	0,00	0,79	0,00	0,84	0,00	1,02	0,56	0,96	0,20	0,95	0,12
14770	ACTG,SET	1,07	0,09	1,13	0,01	0,84	0,01	1,03	0,58	0,87	0,01	0,82	0,00	0,75	0,00	1,03	0,51	0,76	0,00	0,87	0,01	0,71	0,00	0,70	0,00	0,93	0,06	0,88	0,02	0,92	0,05
15097	ACTG,MEP50	0,93	0,05	1,05	0,26	0,66	0,00	0,99	0,83	0,90	0,04	0,80	0,00	0,78	0,00	0,98	0,71	0,81	0,00	1,01	0,76	0,88	0,01	0,81	0,00	0,98	0,73	1,14	0,08	1,30	0,00
15367		0,54	0,01	0,36	0,00	0,17	0,00	0,13	0,00	0,50	0,08	0,27	0,01	0,10	0,00	0,39	0,03	0,12	0,00	0,12	0,00	0,17	0,01	0,67	0,35	1,03	0,94	0,75	0,69	0,66	0,48
15652	ENOG	0,99	0,64	0,84	0,00	0,68	0,00	0,92	0,00	0,69	0,00	0,70	0,00	0,83	0,00	0,87	0,00	0,66	0,00	0,97	0,30	0,90	0,01	0,85	0,00	1,10	0,01	1,07	0,14	1,05	0,26
15904	TBAK	1,17	0,00	1,21	0,02	0,98	0,69	0,90	0,02	0,74	0,00	0,73	0,00	0,56	0,01	0,72	0,06	0,58	0,01	0,92	0,01	0,68	0,00	0,74	0,00	0,85	0,09	0,89	0,06	0,84	0,04
16614		1,00	0,97	1,11	0,02	1,24	0,00	1,08	0,36	0,88	0,20	1,05	0,56	0,77	0,01	1,04	0,52	0,73	0,00	1,01	0,74	0,89	0,07	0,74	0,00	1,09	0,11	0,80	0,00	0,69	0,00
16655	PHP14	0,70	0,00	0,82	0,04	0,60	0,01	0,83	0,00	0,66	0,01	0,64	0,02	1,91	0,00	0,89	0,07	1,56	0,18	2,10	0,00	3,96	0,00	1,41	0,12	1,56	0,00	1,87	0,09	3,76	0,05
16669	TXNL5	0,82	0,01	0,88	0,09	0,69	0,00	0,97	0,48	0,73	0,02	0,75	0,06	2,44	0,01	1,24	0,00	1,99	0,09	1,96	0,00	3,59	0,01	1,38	0,08	1,69	0,00	1,78	0,05	3,44	0,06
16755		0,48	0,01	1,12	0,51	1,03	0,85	0,51	0,00	0,70	0,06	0,56	0,01	0,52	0,00	0,58	0,00	0,41	0,00	1,34	0,14	1,57	0,03	0,79	0,18	0,74	0,06	0,68	0,01	0,58	0,00
16834		0,77	0,01	0,74	0,01	0,57	0,01	0,81	0,00	0,83	0,01	0,68	0,00	0,69	0,00	0,85	0,01	0,65	0,00	0,96	0,46	0,84	0,01	1,02	0,78	1,08	0,30	1,09	0,58	1,07	0,62
16864	PRDX4	0,85	0,28	0,96	0,51	0,95	0,66	1,12	0,00	0,90	0,01	0,94	0,14	0,86	0,00	1,03	0,32	0,86	0,01	1,10	0,53	0,97	0,85	0,78	0,01	1,03	0,66	0,82	0,15	0,86	0,25
16882		0,86	0,42	1,11	0,11	0,87	0,06	0,98	0,45	0,85	0,00	0,81	0,00	0,81	0,00	1,07	0,07	0,65	0,00	1,13	0,50	1,08	0,68	0,76	0,00	1,12	0,06	0,93	0,20	0,87	0,03
16921		0,94	0,19	1,14	0,02	1,28	0,00	0,85	0,00	1,08	0,02	0,91	0,01	0,79	0,00	0,92	0,03	0,85	0,00	0,75	0,00	0,64	0,00	0,78	0,00	0,61	0,00	0,59	0,00	0,51	0,00
16938	PSB4	1,06	0,35	1,05	0,78	1,07	0,76	0,96	0,45	1,01	0,93	0,86	0,04	0,82	0,00	1,07	0,15	0,83	0,00	0,76	0,00	0,65	0,00	0,82	0,26	0,87	0,41	0,67	0,18	0,66	0,16
17069	CAPZB,ANXA3,IPYR	0,81	0,11	0,65	0,01	0,41	0,00	0,78	0,00	0,64	0,00	0,58	0,00	0,81	0,00	0,71	0,00	0,63	0,00	1,22	0,06	1,16	0,14	1,26	0,04	1,27	0,09	1,83	0,00	1,81	0,00
17135	CAZA2	0,78	0,00	0,89	0,00	0,72	0,00	0,90	0,00	0,88	0,00	0,73	0,00	0,88	0,00	0,90	0,01	0,82	0,00	1,12	0,02	0,99	0,71	0,96	0,10	0,88	0,00	0,99	0,76	0,99	0,63
17216	CSN6,CT077,CAZA1	0,44	0,00	0,59	0,00	0,27	0,00	0,50	0,00	0,55	0,00	0,36	0,00	0,41	0,00	0,55	0,00	0,32	0,00	1,25	0,00	1,08	0,04	1,03	0,27	1,09	0,01	1,51	0,00	1,42	0,00
17229	GBB2	0,46	0,00	0,58	0,00	0,31	0,00	0,53	0,00	0,55	0,00	0,39	0,00	0,45	0,00	0,55	0,00	0,37	0,00	1,22	0,00	1,16	0,00	0,99	0,74	1,11	0,02	1,35	0,01	1,40	0,01
17270	CSN4	0,86	0,00	0,79	0,00	0,69	0,00	0,87	0,00	0,75	0,00	0,71	0,00	0,80	0,00	0,90	0,00	0,78	0,00	1,03	0,49	1,00	0,93	0,97	0,27	1,22	0,00	1,05	0,22	1,21	0,00
17332		0,76	0,00	0,91	0,02	0,84	0,00	0,89	0,00	0,74	0,00	0,64	0,00	0,77	0,00	0,82	0,00	0,65	0,00	1,17	0,00	1,02	0,62	0,82	0,00	0,91	0,03	0,77	0,00	0,78	0,00
17364	TBA1A,PEPD	0,83	0,12	1,10	0,04	1,09	0,11	0,93	0,07	0,96	0,35	1,08	0,13	0,73	0,00	0,82	0,00	0,76	0,00	1,03	0,81	0,95	0,66	0,80	0,00	0,80	0,00	0,91	0,11	0,75	0,00
17374	PDIA3,PEPD	0,98	0,74	1,11	0,11	1,19	0,06	0,87	0,00	0,91	0,01	0,98	0,32	0,86	0,00	1,02	0,46	0,87	0,00	0,91	0,17	0,91	0,16	0,85	0,03	0,96	0,41	0,85	0,07	0,76	0,02
17391	PAK2,PDIA3	0,94	0,04	1,03	0,62	0,91	0,09	0,97	0,10	0,88	0,00	0,93	0,02	0,96	0,21	1,21	0,00	1,01	0,82	1,04	0,23	0,92	0,03	0,86	0,04	1,06	0,29	1,02	0,78	0,99	0,89
17422	P4HA1,TCPA,PDIA3	1,06	0,37	0,96	0,41	0,75	0,00	1,05	0,02	1,01	0,45	1,09	0,00	1,37	0,01	1,38	0,05	1,45	0,01	0,90	0,11	0,91	0,15	0,95	0,27	1,01	0,90	1,31	0,00	1,36	0,00
17458	TCPA,CPNE2	0,80	0,00	0,80	0,01	0,83	0,01	1,01	0,81	0,81	0,00	0,78	0,00	1,01	0,74	1,01	0,74	0,67	0,00	1,22	0,00	1,08	0,16	0,97	0,67	1,07	0,27	0,91	0,13	0,69	0,00
17482	GELS	1,04	0,62	1,42	0,03	1,75	0,00	0,91	0,31	1,10	0,26	1,48	0,00	0,66	0,00	0,85	0,00	0,67	0,00	1,16	0,03	1,31	0,00	1,02	0,87	1,24	0,07	1,11	0,15	0,79	0,02
17545	MX1	0,85	0,29	1,09	0,34	1,29	0,05	0,76	0,00	0,86	0,02	1,28	0,00	0,57	0,00	0,71	0,00	0,53	0,00	1,16	0,26	1,40	0,02	1,03	0,59	1,37	0,01	1,29	0,01	0,88	0,09
17707		1,50	0,00	1,91	0,00	2,40	0,00	1,40	0,00	1,60	0,00	2,20	0,00	0,83	0,00	0,91	0,03	1,06	0,36	0,84	0,01	0,84	0,01	0,76	0,03	0,72	0,02	0,83	0,15	0,67	0,03
17878		0,71	0,00	0,66	0,00	0,52	0,00	0,77	0,00	0,71	0,00	0,60	0,00	0,85	0,00	0,94	0,01	0,86	0,00	0,96	0,11	0,78	0,00	0,95	0,37	0,93	0,27	1,02	0,81	1,08	0,42

18275	GRP75,PDIA4	1,00	0,99	1,14	0,08	1,21	0,02	0,91	0,01	0,76	0,00	0,79	0,00	0,97	0,36	1,08	0,00	0,87	0,00	1,12	0,00	1,06	0,06	0,82	0,01	1,04	0,35	0,80	0,00	0,78	0,00
19644	TTL12	1,09	0,01	1,21	0,01	1,16	0,06	1,11	0,03	1,28	0,00	1,33	0,00	0,90	0,09	1,15	0,02	1,20	0,01	0,80	0,00	0,67	0,00	0,83	0,01	0,78	0,00	0,90	0,14	0,85	0,05
20028	NEBU	0,82	0,00	0,90	0,11	0,76	0,01	0,90	0,21	0,87	0,07	0,91	0,22	0,78	0,00	0,94	0,11	0,91	0,22	1,00	0,99	0,82	0,01	0,87	0,08	0,91	0,17	1,09	0,28	1,04	0,72
20414	HS90A	1,24	0,00	1,44	0,00	1,61	0,01	1,22	0,01	1,48	0,00	1,96	0,00	1,09	0,07	1,24	0,00	1,53	0,00	0,82	0,00	0,82	0,00	0,86	0,08	0,81	0,04	1,02	0,85	0,89	0,24
21718	HS90A	0,75	0,00	0,86	0,04	0,81	0,03	0,89	0,02	0,86	0,01	0,87	0,01	0,82	0,00	0,93	0,03	0,86	0,00	1,04	0,19	0,96	0,13	0,88	0,11	0,95	0,47	0,94	0,45	0,93	0,42
22159	PDIA4,HSP71,ANXA6,TF65,GRP75	1,47	0,00	2,16	0,00	2,40	0,00	1,47	0,00	1,91	0,00	2,97	0,00	1,38	0,00	1,80	0,00	2,07	0,00	0,81	0,00	0,90	0,00	0,72	0,00	0,80	0,01	1,00	0,94	0,83	0,00
23159		0,75	0,00	0,80	0,00	0,86	0,02	0,77	0,00	0,64	0,00	0,65	0,00	0,89	0,04	0,91	0,04	0,73	0,00	1,06	0,21	0,98	0,60	0,82	0,00	0,93	0,03	0,77	0,01	0,70	0,00
23217		0,63	0,00	0,58	0,00	0,36	0,00	0,69	0,00	0,65	0,00	0,54	0,00	0,58	0,00	0,69	0,00	0,54	0,00	0,97	0,41	0,90	0,06	1,00	0,96	1,17	0,00	1,33	0,00	1,46	0,00
24906		0,47	0,00	0,30	0,00	0,09	0,00	0,53	0,01	0,61	0,00	0,53	0,00	0,33	0,29	0,60	0,44	0,34	0,26	0,97	0,83	0,50	0,21	1,74	0,21	1,39	0,63	5,36	0,00	2,81	0,54
25129		0,79	0,00	0,72	0,02	0,49	0,00	0,93	0,61	1,23	0,16	0,60	0,01	0,80	0,01	1,22	0,01	1,08	0,66	0,90	0,34	0,47	0,00	1,31	0,11	0,78	0,08	0,94	0,69	1,03	0,91
25674	PLOD3	1,46	0,00	1,33	0,00	1,45	0,00	1,12	0,00	0,98	0,35	0,84	0,00	0,84	0,00	0,96	0,00	0,74	0,00	0,79	0,01	0,73	0,00	0,75	0,00	0,91	0,05	0,59	0,00	0,64	0,00
25932		0,53	0,00	0,29	0,00	0,17	0,00	0,55	0,00	0,53	0,00	0,26	0,00	0,60	0,00	0,76	0,01	0,33	0,00	0,94	0,23	0,74	0,00	1,63	0,03	1,69	0,02	1,41	0,25	1,26	0,68
26688		0,80	0,36	0,68	0,26	0,00	0,01	0,70	0,05	0,67	0,02	0,88	0,54	0,64	0,00	0,87	0,03	0,72	0,00	0,70	0,06	0,73	0,05	0,78	0,46	1,18	0,53	1.000,00	0,01	1.000,00	0,00
26959	CTNA1	0,51	0,00	0,07	0,00	0,00	0,00	0,53	0,00	0,36	0,00	0,28	0,00	0,34	0,00	0,65	0,00	0,37	0,00	0,91	0,48	0,61	0,01	4,53	0,01	8,61	0,00	1.000,00	0,00	1.000,00	0,00
27152		0,84	0,01	0,16	0,00	0,00	0,00	0,79	0,00	0,50	0,00	0,51	0,00	0,61	0,00	0,74	0,01	0,38	0,00	1,29	0,00	1,18	0,02	4,28	0,01	7,42	0,00	1.000,00	0,00	1.000,00	0,00
27793		0,82	0,01	0,88	0,08	0,45	0,00	0,64	0,02	0,75	0,06	0,63	0,01	0,64	0,00	0,89	0,08	0,87	0,04	0,92	0,29	0,74	0,00	1,01	0,90	0,96	0,58	1,64	0,00	1,82	0,00
28319	SYG,CALD1	0,68	0,00	0,60	0,23	6,91	0,27	0,76	0,00	0,74	0,00	0,62	0,00	0,79	0,00	0,87	0,01	0,86	0,02	0,99	0,46	0,98	0,47	1,09	0,86	1,23	0,66	0,08	0,24	0,11	0,25
29087		1,65	0,00	2,37	0,01	3,87	0,00	1,81	0,00	2,68	0,00	3,82	0,00	1,93	0,00	2,33	0,00	2,67	0,00	0,80	0,00	0,94	0,01	0,83	0,21	0,80	0,15	0,72	0,10	0,56	0,03
29100	TADBP,ACY1	0,61	0,00	0,74	0,00	0,48	0,00	0,65	0,00	0,60	0,00	0,43	0,00	0,55	0,00	0,61	0,00	0,51	0,00	1,19	0,06	0,98	0,81	0,89	0,08	0,89	0,12	0,99	0,90	1,17	0,06
29521	HNRH1,HNRH2	0,66	0,00	0,65	0,00	0,49	0,00	0,76	0,00	0,68	0,00	0,60	0,00	0,74	0,00	0,83	0,00	0,60	0,00	1,07	0,13	1,10	0,05	0,97	0,06	1,23	0,00	1,15	0,01	1,20	0,00
29990	TCPA	1,07	0,14	1,07	0,27	1,01	0,85	1,14	0,03	1,27	0,00	1,27	0,00	1,03	0,50	1,31	0,00	1,05	0,27	0,85	0,00	0,85	0,00	0,94	0,20	1,08	0,12	1,00	0,98	0,92	0,03
30544		0,91	0,07	0,80	0,00	0,74	0,00	0,90	0,02	1,00	0,99	0,94	0,05	0,67	0,00	0,84	0,00	0,70	0,00	0,85	0,01	0,82	0,00	1,09	0,06	1,16	0,00	1,10	0,00	1,04	0,03
30752	SODC	0,71	0,00	0,63	0,00	0,53	0,00	0,70	0,00	0,68	0,00	0,54	0,00	0,68	0,00	0,70	0,00	0,56	0,00	0,84	0,01	0,78	0,00	0,93	0,10	0,91	0,03	0,87	0,05	0,86	0,07
31044		1,14	0,08	0,76	0,40	0,68	0,17	1,24	0,00	0,92	0,05	1,04	0,34	1,09	0,05	1,24	0,00	0,90	0,05	0,97	0,28	0,94	0,09	1,08	0,83	1,61	0,12	1,37	0,23	1,31	0,30
31063	MYH13,SEPT15,ACTY,OAT	0,63	0,00	0,43	0,06	0,27	0,02	0,69	0,00	0,69	0,00	0,56	0,00	0,69	0,00	0,74	0,00	0,61	0,00	1,16	0,00	1,19	0,00	1,71	0,27	1,85	0,20	2,20	0,21	2,45	0,15
31086		0,57	0,00	0,31	0,01	0,20	0,00	0,69	0,00	0,83	0,00	0,60	0,00	0,85	0,00	0,97	0,01	1,12	0,02	0,98	0,27	1,01	0,91	2,17	0,07	2,14	0,08	2,53	0,13	3,93	0,02
33115		0,63	0,00	0,19	0,00	0,01	0,00	0,83	0,00	0,59	0,00	0,60	0,00	0,92	0,04	1,01	0,65	0,59	0,00	1,41	0,00	1,23	0,00	3,31	0,02	4,56	0,00	47,80	0,00	37,67	0,00
35204		0,82	0,00	0,51	0,02	0,26	0,00	0,90	0,00	0,81	0,00	0,79	0,00	0,85	0,00	0,98	0,29	0,94	0,07	1,03	0,41	0,99	0,87	1,50	0,14	1,85	0,03	2,89	0,01	3,52	0,00
35820	DPYL2	1,65	0,00	0,75	0,48	0,11	0,00	1,55	0,00	2,35	0,00	3,28	0,00	1,23	0,03	1,44	0,00	1,53	0,00	0,75	0,00	0,88	0,09	2,53	0,02	2,28	0,03	23,27	0,00	15,97	0,00
36428	U2AF2	0,83	0,00	0,27	0,02	0,00	0,00	0,91	0,03	0,94	0,14	1,04	0,36	0,66	0,00	0,76	0,00	0,67	0,00	1,00	0,99	1,02	0,68	3,20	0,05	3,61	0,03	1.000,00	0,00	1.000,00	0,00
37898		0,45	0,00	0,37	0,00	0,22	0,00	0,57	0,00	0,53	0,00	0,45	0,00	0,55	0,00	0,63	0,00	0,49	0,00	1,24	0,00	1,14	0,01	1,38	0,25	1,58	0,13	1,97	0,12	2,06	0,10
39482		1,73	0,00	1,75	0,00	2,35	0,00	1,64	0,00	1,90	0,00	2,32	0,00	1,16	0,05	1,35	0,01	1,17	0,05	0,84	0,08	0,83	0,04	0,96	0,62	0,96	0,62	0,87	0,29	0,62	0,01
40401		2,43	0,00	3,10	0,00	3,99	0,00	1,70	0,00	2,27	0,00	3,40	0,00	2,27	0,00	2,60	0,00	2,92	0,00	0,73	0,00	0,80	0,00	0,76	0,00	0,72	0,00	0,88	0,05	0,63	0,00
41097		2,46	0,00	3,19	0,00	4,60	0,00	1,56	0,03	2,21	0,00	3,26	0,00	1,51	0,00	1,86	0,00	2,02	0,00	0,69	0,00	0,77	0,00	0,75	0,01	0,74	0,00	0,77	0,07	0,55	0,00
41789		1,62	0,00	1,61	0,01	2,14	0,01	1,26	0,06	1,44	0,00	1,98	0,00	0,75	0,00	1,04	0,12	0,98	0,35	0,87	0,19	0,82	0,02	1,00	0,99	1,14	0,14	1,04	0,76	0,80	0,14
42780		3,26	0,01	3,51	0,00	4,59	0,00	1,75	0,00	2,34	0,00	3,48	0,00	2,77	0,00	2,74	0,00	3,34	0,00	0,68	0,10	0,73	0,16	0,84	0,15	0,67	0,01	0,96	0,76	0,62	0,03
43156		3,24	0,00	4,01	0,00	6,02	0,00	1,80	0,00	2,45	0,00	3,55	0,00	2,70	0,00	2,63	0,00	3,20	0,00	0,67	0,01	0,82	0,10	0,74	0,07	0,64	0,02	0,71	0,16	0,52	0,03
43845		1,39	0,00	1,76	0,06	3,17	0,07	1,41	0,00	0,97	0,70	1,17	0,10	1,01	0,85	1,15	0,01	0,98	0,68	1,08	0,22	0,92	0,25	0,59	0,06	0,82	0,35	0,39	0,10	0,39	0,09
44539	MARCS	0,95	0,83	0,92	0,70	1,18	0,51	1,11	0,30	0,85	0,16	0,88	0,23	0,58	0,00	0,75	0,00	1,18	0,53	1,13	0,41	0,87	0,47	0,90	0,48	1,16	0,24	0,72	0,08	1,42	0,34
45562		1,13	0,00	1,27	0,00	1,28	0,00	1,14	0,01	1,24	0,00	1,42	0,00	1,07	0,01	1,19	0,00	1,09	0,01	0,94	0,03	1,02	0,48	0,91	0,01	1,02	0,46	1,04	0,19	0,93	0,04
46258		0,80	0,00	0,88	0,00	0,83	0,00	0,79	0,00	0,75	0,00	0,71	0,00	0,72	0,00	0,81	0,00	0,64	0,00	0,99	0,65	0,98	0,42	0,85	0,00	1,00	0,89	0,86	0,00	0,83	0,01
47508		0,93	0,02	0,88	0,00	0,75	0,00	1,04	0,08	0,82	0,00	0,79	0,00	0,93	0,01	1,00	0,84	0,79	0,00	1,08	0,02	0,96	0,14	0,90	0,01	1,10	0,01	1,01	0,77	1,01	0,87
48368	BASP	0,81	0,03	0,45	0,00	0,35	0,00	0,90	0,31	0,55	0,00	0,48	0,00	0,54	0,00	0,68	0,00	0,34	0,00	1,16	0,19	0,86	0,37	1,29	0,06	2,01	0,00	1,46	0,01	1,28	0,42
49585		1,32	0,00	1,56	0,00	1,67	0,00	1,44	0,00	1,35	0,00	1,82	0,00	1,19	0,00	1,59	0,00	1,45	0,00	0,92	0,13	0,84	0,02	0,73	0,00	0,96	0,08	0,92	0,11	0,81	0,00
50273	NSAP,CALR	1,13	0,00	1,19	0,00	1,26	0,00	1,18	0,00	1,11	0,00	1,36	0,00	1,05	0,01	1,30	0,00	1,02	0,19	1,06	0,00	0,98	0,04	0,95	0,06	1,15	0,00	1,09	0,07	0,85	0,01
51068		0,97	0,52	0,87	0,06	0,70	0,00	0,95	0,36	0,61	0,00	0,55	0,00	0,85	0,01	0,97	0,12	0,60	0,00	1,17	0,04	0,85	0,02	0,84	0,04	1,08	0,16	0,95	0,47	0,83	0,04
51803		0,65	0,00	0,35	0,00	0,07	0,00	0,67	0,01	0,44	0,00	0,31	0,00	0,65	0,00	0,52	0,00	0,52	0,00	1,49	0,00	1,29	0,00	1,83	0,00	1,93	0,00	6,82	0,00	10,25	0,00
52561		1,05	0,13	1,19	0,00	1,23	0,00	0,89	0,00	0,79	0,00	0,77	0,00	0,58	0,00	0,75	0,00	0,60	0,00	0,86	0,00	0,76	0,00	0,67	0,00	0,87	0,00	0,63	0,00	0,67	0,00
53317	CALU	1,06	0,05	1,23	0,00	1,37	0,00	1,03	0,24	0,88	0,00	0,83	0,00	0,62	0,00	0,77	0,00	0,63	0,00	1,05	0,05	0,90	0,00	0,77	0,00	0,97	0,34	0,66	0,00	0,72	0,00
54018	CALU	1,05	0,21	1,21	0,00	1,20	0,01	0,84	0,00	0,74	0,00	0,69	0,00	0,48	0,00	0,64	0,00	0,51	0,00	0,83	0,00	0,65	0,00	0,64	0,00	0,76	0,00	0,60	0,00	0,61	0,00
55507	RCN1	1,07	0,03	1,15	0,00	1,19	0,00	1,02	0,07	0,83	0,00	0,88	0,00	0,77	0,00	0,94	0,18	0,74	0,00	0,93	0,01	0,83	0,00	0,70	0,00	0,93	0,10	0,72	0,00	0,71	0,00
55610	TPM1,TPM2	0,76	0,00	0,61	0,00	0,50	0,00	0,49	0,00	0,47	0,00	0,36	0,00	0,42	0,00	0,46	0,00	0,42	0,00	0,74	0,00	0,64	0,00	0,89	0,00	0,87	0,01	0,84	0,02	0,98	0,62
56308	TPM2	0,86	0,02	0,82	0,00	0,64	0,00	0,82	0,00	0,73	0,00	0,65	0,00	0,68	0,00	0,92	0,00	0,77	0,00	0,93	0,31	0,86	0,04	0,88	0,00	1,21	0,00	1,01	0,90	1,31	0,01
57056		1,14	0,02	0,89	0,18	0,82	0,28	0,95	0,01	0,87	0,00	0,78	0,00	0,76	0,03	0,80	0,14	0,93	0,59	0,78	0,00	0,58	0,00	0,92	0,32	0,78	0,10	0,89	0,57	0,99	0,95
57776		1,49	0,00	1,80	0,00	2,19	0,00	1,28	0,01	1,08	0,29	1,22	0,02	1,25	0,01	1,16	0,06	0,99	0,90	1,14	0,14	1,22	0,03	0,79	0,04	0,93	0,42	0,73	0,00	0,66	0,00
57837		0,84	0,02	1,01	0,92	0,47	0,00	0,67	0,00	0,68	0,00	0,69	0,00	0,29	0,26	0,40	0,33	0,42	0,34	0,96	0,54	0,84	0,02	0,80	0,06	0,97	0,69	1,76	0,00	2,17	0,00
58577		0,98	0,80	0,78	0,09	0,51	0,00	0,93	0,57	1,15	0,05	0,66	0,06	0,81	0,23	0,89	0,30	1,13	0,63	0,87	0,27	0,59	0,01	1,36	0,04	0,82	0,30	1,18	0,58	1,59	0,22
59353	EF1B	0,97	0,32	0,83	0,00	0,83	0,00	1,08	0,07	0,83	0,00	0,85	0,01	0,98	0,71	1,13	0,01	0,94	0,16	1,08	0,04	0,94	0,09	0,97	0,33	1,26	0,00	0,99	0,80	1,04	0,34
60178		1,09	0,00	0,86	0,01	0,76	0,00	1,18	0,01	0,88	0,01	0,87	0,01	0,83	0,00	0,90	0,01	0,86	0,00	0,82	0,00	0,65	0,00	0,77	0,00	0,89	0,05	0,87	0,01	0,97	0,25
61022		1,50	0,01	1,13	0,19	0,82	0,14	1,45	0,00	1,04	0,56	1,21	0,01	1,56	0,00	1,62	0,00	1,28	0,03	0,87	0,08	0,86	0,13	0,83	0,03	1,19	0,01	1,33	0,01	1,30	0,08
61862	PCNA	0,83	0,00	0,83	0,00	0,61	0,00	0,81	0,00	0,68	0,00	0,56	0,00	0,70	0,00	0,83	0,00	0,55	0,00	1,06	0,09	0,84	0,00	0,88	0,00	1,00	0,86	0,99	0,83	0,90	0,06
62715		0,85	0,00	0,99	0,81	0,67	0,00	0,99	0,71	0,87	0,01	0,77	0,00	0,72	0,00	0,98	0,40	0,69	0,00	1,19	0,00	1,01	0,74	0,89	0,07	1,18	0,00	1,17	0,04	1,23	0,02
64437	EF1B	0,98	0,28	0,98	0,69	0,67	0,00	1,07	0,19	0,89	0,00	0,76	0,00	0,78	0,00	1,10	0,01	0,76	0,00	1,07	0,15	0,75	0,00	0,88	0,03	1,06	0,17	1,11	0,03	1,07	0,25
65305		0,93	0,36	0,76	0,00	0,56	0,00	0,97	0,54	0,84	0,01	0,78	0,01	0,75	0,00	0,85	0,00	0,82	0,00	1,11	0,21	0,90	0,18	1,17	0,04	1,25	0,00	1,48	0,01	1,64	0,00
66151		2,07	0,00	1,43	0,02	1,63	0,04	1,81	0,01	1,09	0,41	1,35	0,02	2,28	0,00	2,65	0,00	2,79	0,00	1,28	0,07	1,00	1,00	1,11	0,19	1,68	0,00	1,21	0,19	1,56	0,01
66993		1,09	0,24	0,92	0,30	0,89	0,28	1,21	0,00	0,86	0,04	1,00	0,95	1,17	0,04	1,27	0,00	1,01	0,83	1,01	0,84	0,96	0,34	0,86	0,07	1,24	0,00	1,02	0,79	1,02	0,79
68693		0,84	0,00	1,02	0,72	0,94	0,18	0,99	0,83	0,83	0,00	0,76	0,00	1,16	0,00	1,17	0,00	1,07	0,03	1,21	0,00	1,07	0,05	0,83	0,03	0,89	0,08	0,82	0,01	0,88	0,03
69657		1,60	0,00	2,13	0,00	3,22	0,00	1,48	0,01	1,20	0,19	1,42	0,01	0,48	0,03	1,16	0,44	0,95	0,85	1,24	0,01	0,73	0,00	0,76	0,01	1,32	0,00	0,59	0,00	0,71	0,22
71376		1,07	0,58	0,98	0,91	0,59	0,00	1,53	0,01	1,22	0,05	1,21	0,06	0,86	0,67	1,08	0,81	0,84	0,63	1,25	0,15	1,07	0,55	1,09	0,64	1,46	0,04	1,79	0,00	1,89	0,00
71401		0,72	0,00	0,85	0,02	0,54	0,00	0,89	0,00	0,82	0,00	0,63	0,00	0,72	0,00	0,93	0,00	0,66	0,00	1,49	0,00	1,30	0,00	1,16	0,02	1,42	0,00	1,41	0,00	1,58	0,00
71445		2,20	0,00	2,62	0,00	3,70	0,00	2,31	0,00	2,66	0,00	2,87	0,00	1,95	0,00	2,48	0,00	2,78	0,00	0,82	0,09	0,80	0,04	0,79	0,11	0,85	0,24	0,61	0,04	0,68	0,08
72360	MLRN	0,89	0,01	0,81	0,02	0,62	0,01	1,10	0,02	0,86	0,00	0,73	0,00	1,15	0,01	1,13	0,05	0,86	0,02	1,17	0,01	1,10	0,05	1,00	0,96	1,19	0,07	1,13	0,38	1,20	0,22
74132		1,85	0,00	2,07	0,00	2,67	0,01	1,07	0,28	1,11	0,05	0,94	0,22	0,97	0,55	1,23	0,00	0,88	0,10	0,99	0,88	0,85	0,01	0,92	0,26	0,96	0,52	0,60	0,02	0,53	0,01
75108		1,06	0,41	0,99	0,95	0,66	0,00	0,87	0,04	1,02	0,65	0,85	0,01	1,16	0,12	1,26	0,01	1,29	0,01	0,66	0,00	0,70	0,01	0,83	0,09	0,81	0,09	1,05	0,35	1,27	0,03
75950		0,88	0,00	0,88	0,13	0,56	0,01	1,06	0,58	0,85	0,03	0,68	0,00	0,86	0,26	0,87	0,29	0,60	0,02	1,06	0,52	0,99	0,82	0,85	0,09	1,00	0,96	1,07	0,70	1,09	0,64
78618		0,97	0,27	1,04	0,35	1,14	0,01	0,90	0,02	0,76	0,00	0,67	0,00	0,80	0,00	0,87	0,01	0,76	0,00	0,99	0,69	0,80	0,00	0,78	0,00	0,82	0,00	0,62	0,00	0,65	0,00
80360		0,78	0,03	0,75	0,04	0,47	0,01	0,80	0,03	0,72	0,00	0,55	0,00	0,92	0,05	0,86	0,01	0,61	0,00	1,18	0,07	1,41	0,00	1,10	0,31	1,39	0,01	1,34	0,17	1,57	0,06
82135		1,16	0,01	0,92	0,03	0,76	0,01	1,03	0,23	0,79	0,00	0,79	0,00	1,17	0,00	1,27	0,00	1,12	0,00	0,94	0,14	0,83	0,00	0,91	0,01	1,14	0,00	1,11	0,10	1,22	0,01
83022		0,88	0,00	1,04	0,37	0,78	0,00	0,95	0,34	0,81	0,00	0,71	0,00	0,67	0,00	0,87	0,00	0,63	0,00	1,09	0,13	0,87	0,00	0,79	0,00	0,96	0,31	0,91	0,05	0,91	0,21
83942		0,85	0,01	0,96	0,42	0,56	0,00	0,80	0,00	0,81	0,00	0,64	0,00	0,69	0,00	0,86	0,01	0,67	0,00	0,84	0,00	0,70	0,00	0,76	0,00	0,77	0,00	1,04	0,69	1,02	0,83
85707		0,88	0,02	0,84	0,01	0,74	0,00	0,91	0,10	0,77	0,00	0,69	0,00	0,84	0,00	0,89	0,00	0,68	0,00	1,09	0,10	1,01	0,87	0,97	0,35	1,12	0,00	0,97	0,58	0,96	0,58
86593		1,01	0,77	1,11	0,03	1,14	0,05	1,12	0,05	1,06	0,34	1,14	0,15	0,76	0,06	1,04	0,69	0,80	0,10	0,93	0,06	0,82	0,00	0,79	0,00	1,02	0,61	0,83	0,06	0,76	0,01
87491	TPM2	0,98	0,72	1,04	0,41	0,89	0,04	0,91	0,01	0,93	0,00	0,69	0,00	0,83	0,05	0,94	0,38	1,02	0,77	0,87	0,01	0,64	0,00	0,83	0,00	0,69	0,00	0,72	0,00	0,88	0,08
88373	HDGF	0,72	0,00	0,67	0,00	0,47	0,00	0,86	0,01	0,69	0,00	0,67	0,00	0,71	0,00	0,80	0,00	0,56	0,00	1,10	0,05	1,03	0,52	0,95	0,24	1,25	0,00	1,32	0,00	1,24	0,01
89291		0,74	0,01	0,64	0,00	0,43	0,00	0,91	0,17	0,83	0,02	0,64	0,00	0,67	0,00	0,75	0,00	0,43	0,00	0,79	0,02	0,63	0,00	0,84	0,10	0,82	0,08	0,95	0,71	0,70	0,06
90190	SET	1,19	0,24	0,77	0,25	0,56	0,04	1,10	0,01	0,69	0,00	0,86	0,02	0,80	0,00	0,81	0,00	0,69	0,00	1,05	0,43	0,87	0,09	1,03	0,89	1,35	0,09	1,74	0,00	1,58	0,00
91144		1,04	0,66	0,93	0,19	0,68	0,00	0,81	0,08	0,76	0,03	0,72	0,02	0,50	0,00	0,86	0,02	0,58	0,00	0,79	0,04	0,66	0,01	0,83	0,01	1,27	0,00	1,08	0,27	1,17	0,26
92002	SET,SPSY,HSP7C,RSSA	0,85	0,00	0,96	0,38	0,67	0,00	0,78	0,10	0,58	0,01	0,61	0,01	0,78	0,00	0,86	0,00	0,76	0,00	1,26	0,01	0,78	0,00	0,83	0,01	0,77	0,00	1,26	0,05	0,97	0,80
92901		1,21	0,00	1,23	0,00	2,10	0,00	1,07	0,12	0,80	0,00	0,84	0,00	1,00	1,00	1,19	0,00	1,04	0,17	1,00	0,93	0,80	0,00	0,74	0,00	0,93	0,12	0,45	0,00	0,48	0,00
93790	TBB5,TBB2C,ENOG	0,60	0,00	0,57	0,00	0,44	0,00	0,76	0,00	0,61	0,00	0,44	0,00	0,71	0,00	0,67	0,00	0,51	0,00	1,07	0,32	0,87	0,09	0,93	0,38	0,87	0,18	0,86	0,22	0,85	0,07
94683	RD23B	1,02	0,62	1,14	0,01	1,30	0,00	0,96	0,16	0,88	0,01	0,90	0,02	0,80	0,00	0,77	0,00	0,67	0,00	0,98	0,65	0,94	0,16	0,81	0,00	0,82	0,00	0,72	0,00	0,63	0,00
95609	PDIA1	0,90	0,02	0,99	0,81	0,93	0,01	0,96	0,13	0,90	0,01	0,88	0,00	0,75	0,00	0,89	0,00	0,70	0,00	0,94	0,10	0,90	0,02	0,80	0,00	0,97	0,51	0,83	0,00	0,81	0,00
97395		1,05	0,33	1,08	0,20	1,22	0,01	0,92	0,06	0,98	0,66	1,18	0,00	0,99	0,86	1,16	0,06	1,11	0,13	0,90	0,03	0,88	0,01	0,94	0,23	1,00	0,99	1,00	0,93	0,85	0,00
99319	PDIA1	0,94	0,07	0,96	0,41	0,94	0,07	0,96	0,01	0,83	0,00	0,86	0,00	0,96	0,10	0,95	0,23	0,82	0,00	0,99	0,72	0,97	0,14	0,84	0,01	0,94	0,25	0,90	0,02	0,83	0,00
99729		1,01	0,79	1,23	0,01	1,52	0,00	0,90	0,39	0,95	0,62	1,03	0,84	0,82	0,06	1,00	0,98	0,82	0,07	0,87	0,01	0,86	0,01	0,75	0,00	0,87	0,02	0,66	0,00	0,58	0,00
99730	RD23B,PDIA1	0,93	0,08	1,00	0,94	1,04	0,31	0,90	0,00	0,82	0,00	0,77	0,00	0,87	0,00	0,83	0,00	0,69	0,00	1,03	0,14	0,93	0,01	0,88	0,00	0,83	0,00	0,79	0,00	0,67	0,00
102094		2,18	0,00	2,76	0,00	4,06	0,00	1,63	0,00	2,21	0,00	3,40	0,00	1,98	0,00	2,34	0,00	2,73	0,00	0,73	0,00	0,87	0,00	0,78	0,01	0,81	0,02	0,82	0,00	0,64	0,00
103047		1,29	0,00	1,25	0,00	1,37	0,00	1,16	0,00	1,12	0,00	1,39	0,00	0,97	0,01	1,20	0,00	1,07	0,01	0,88	0,00	0,88	0,00	0,88	0,01	1,13	0,01	1,00	0,92	0,92	0,04
104405		1,86	0,00	2,27	0,00	3,04	0,00	1,42	0,00	1,81	0,00	2,73	0,00	1,44	0,00	1,58	0,00	1,95	0,00	0,79	0,00	0,91	0,00	0,82	0,00	0,81	0,00	0,93	0,08	0,75	0,00

104582	ENPL	0,97	0,30	1,00	0,97	1,05	0,05	1,00	0,96	0,87	0,01	0,82	0,00	0,84	0,00	1,03	0,47	0,71	0,00	1,05	0,07	0,96	0,15	0,89	0,01	1,15	0,00	0,80	0,00	0,75	0,00
105671	GRP78,ENPL	1,04	0,61	1,12	0,38	1,23	0,14	1,37	0,00	1,02	0,69	0,94	0,24	0,95	0,54	0,89	0,31	1,10	0,27	1,30	0,01	0,95	0,34	0,90	0,37	0,83	0,18	0,75	0,06	0,93	0,49
108562		1,51	0,01	1,75	0,01	1,85	0,02	0,91	0,70	1,17	0,49	1,42	0,14	2,13	0,00	1,82	0,00	2,38	0,00	0,90	0,18	0,96	0,66	1,00	0,96	0,70	0,01	1,15	0,34	0,87	0,39
109442		0,68	0,01	0,50	0,01	0,62	0,02	0,92	0,45	0,98	0,84	0,81	0,14	0,63	0,11	2,05	0,03	0,58	0,08	0,57	0,02	0,39	0,00	0,82	0,55	1,75	0,09	0,54	0,06	0,39	0,02
110384		0,71	0,01	0,58	0,03	0,46	0,02	0,79	0,04	0,94	0,55	0,68	0,02	0,70	0,03	1,54	0,08	0,56	0,01	0,65	0,01	0,40	0,00	0,95	0,84	1,07	0,83	0,85	0,68	0,48	0,17
111550		1,05	0,23	1,10	0,02	1,12	0,01	0,99	0,89	0,92	0,08	0,89	0,04	0,89	0,01	0,95	0,03	0,80	0,00	0,99	0,79	0,95	0,25	0,88	0,00	0,97	0,09	0,84	0,00	0,80	0,00
112515		0,51	0,01	0,42	0,00	0,34	0,01	0,93	0,33	0,65	0,00	0,46	0,00	0,92	0,36	0,64	0,00	0,52	0,00	1,44	0,01	1,14	0,37	1,22	0,10	0,97	0,79	1,06	0,79	0,96	0,86
113114		0,84	0,02	0,92	0,17	0,72	0,01	0,77	0,08	0,65	0,01	0,62	0,01	0,61	0,00	0,80	0,00	0,64	0,00	0,94	0,48	0,79	0,00	0,73	0,00	0,94	0,07	0,89	0,15	0,96	0,71
114043		0,71	0,00	0,71	0,01	0,34	0,00	0,83	0,05	0,65	0,01	0,63	0,00	0,52	0,00	0,82	0,00	0,53	0,00	1,11	0,06	0,92	0,34	0,87	0,29	1,43	0,00	1,79	0,00	1,97	0,00
114983		0,75	0,00	0,70	0,00	0,35	0,00	0,97	0,31	0,72	0,02	0,86	0,04	0,54	0,00	0,85	0,00	0,57	0,00	1,02	0,77	0,87	0,17	0,81	0,17	1,45	0,00	1,95	0,01	1,98	0,00
115953		0,80	0,08	0,88	0,30	0,68	0,08	0,86	0,20	0,69	0,02	0,77	0,03	0,69	0,00	0,87	0,00	0,66	0,00	1,03	0,80	0,95	0,38	0,74	0,03	1,07	0,26	1,07	0,68	1,06	0,78
118821		0,74	0,21	1,64	0,10	1,00	0,98	0,80	0,06	0,97	0,67	0,95	0,51	0,40	0,00	0,82	0,00	0,57	0,00	1,23	0,20	1,34	0,14	0,67	0,10	1,23	0,25	1,08	0,42	1,41	0,02
119751		0,74	0,00	0,83	0,01	0,58	0,00	0,81	0,05	0,67	0,01	0,59	0,00	0,54	0,00	0,77	0,00	0,53	0,00	1,10	0,01	0,89	0,00	0,81	0,01	1,14	0,03	1,02	0,77	1,12	0,13
120878	GRP78,PDIA4	0,95	0,67	0,99	0,92	0,83	0,18	1,10	0,09	0,89	0,05	0,91	0,10	0,79	0,00	0,95	0,36	0,58	0,00	0,96	0,65	0,93	0,38	0,75	0,05	1,08	0,47	0,91	0,08	0,78	0,03
121837		1,11	0,62	0,71	0,09	0,64	0,03	1,08	0,25	1,21	0,02	1,32	0,01	0,70	0,00	1,13	0,02	0,63	0,00	0,60	0,05	0,70	0,11	1,04	0,77	1,75	0,00	1,28	0,01	1,09	0,35
122740	DP13B	1,17	0,16	2,37	0,01	4,00	0,00	1,20	0,15	1,05	0,72	1,58	0,00	0,74	0,00	1,33	0,00	1,36	0,00	0,70	0,01	0,80	0,03	0,31	0,00	0,71	0,11	0,27	0,00	0,43	0,00
124300		1,98	0,00	2,46	0,00	2,95	0,00	1,46	0,00	1,72	0,00	2,50	0,00	1,60	0,00	1,70	0,00	1,82	0,00	0,76	0,00	0,89	0,07	0,72	0,00	0,76	0,00	0,88	0,02	0,67	0,00
124470		0,95	0,06	1,02	0,62	0,87	0,00	0,94	0,15	0,86	0,01	0,87	0,01	0,74	0,00	0,93	0,01	0,71	0,00	1,03	0,41	0,94	0,01	0,88	0,01	1,11	0,02	1,05	0,11	0,99	0,78
125371		0,59	0,01	0,52	0,00	0,43	0,01	0,77	0,00	0,63	0,00	0,61	0,01	0,95	0,63	0,76	0,02	0,57	0,00	1,63	0,00	1,38	0,03	1,49	0,01	1,24	0,00	1,74	0,05	1,12	0,28
126195	ICAL	0,81	0,00	0,80	0,00	0,66	0,00	0,91	0,04	0,71	0,00	0,73	0,00	0,73	0,00	0,87	0,00	0,61	0,00	1,18	0,00	1,08	0,01	0,94	0,16	1,32	0,00	1,16	0,00	1,13	0,00
129069	DDB1	1,02	0,70	0,99	0,82	0,89	0,04	0,89	0,00	0,81	0,00	0,82	0,00	0,68	0,00	0,87	0,00	0,68	0,00	0,93	0,04	0,80	0,00	0,87	0,01	1,06	0,10	0,97	0,20	0,92	0,04
129210		0,99	0,43	0,99	0,65	1,02	0,55	1,01	0,89	0,79	0,00	0,79	0,00	0,69	0,00	0,86	0,01	0,67	0,00	1,02	0,53	0,68	0,00	0,80	0,00	0,85	0,00	0,78	0,00	0,65	0,00
130985		0,71	0,00	0,70	0,01	0,63	0,01	0,81	0,17	0,76	0,11	0,63	0,02	0,69	0,00	0,82	0,01	0,65	0,00	1,15	0,14	0,91	0,22	1,08	0,52	1,10	0,30	1,01	0,97	0,97	0,81
131133		0,71	0,00	0,79	0,02	0,64	0,00	0,74	0,00	0,72	0,00	0,61	0,00	0,60	0,00	0,77	0,00	0,57	0,00	1,07	0,14	0,88	0,06	0,94	0,42	1,01	0,86	0,99	0,87	0,93	0,36
131962		0,66	0,09	0,78	0,27	0,39	0,03	0,73	0,10	0,51	0,02	0,29	0,00	0,30	0,00	0,83	0,01	0,21	0,00	0,77	0,06	0,55	0,00	0,45	0,00	1,28	0,05	0,52	0,01	0,65	0,14
132415		2,66	0,00	3,62	0,00	4,30	0,00	2,05	0,00	3,06	0,00	4,75	0,00	2,37	0,00	3,24	0,00	3,33	0,00	0,67	0,01	0,70	0,01	0,73	0,01	0,71	0,00	0,96	0,72	0,61	0,01
132924	ENPL	0,75	0,05	0,86	0,23	0,76	0,10	0,90	0,42	0,64	0,04	0,71	0,04	0,73	0,00	0,89	0,00	0,67	0,00	1,10	0,12	0,94	0,38	0,68	0,01	1,00	0,92	0,86	0,07	0,86	0,07
135817		0,87	0,34	0,94	0,79	0,60	0,05	0,89	0,19	1,11	0,54	0,82	0,15	1,42	0,32	1,43	0,00	1,23	0,35	0,70	0,04	0,53	0,04	0,80	0,39	0,49	0,04	0,93	0,77	0,66	0,23
136093		0,93	0,20	0,92	0,22	0,68	0,00	0,85	0,00	0,88	0,00	0,70	0,00	0,65	0,00	0,94	0,01	0,81	0,00	0,81	0,00	0,69	0,00	0,84	0,03	1,01	0,91	0,91	0,09	1,16	0,04
137923		0,98	0,71	1,02	0,74	1,09	0,21	0,96	0,64	0,83	0,04	0,83	0,04	0,60	0,00	0,83	0,00	0,62	0,00	1,06	0,51	0,88	0,09	0,87	0,09	1,17	0,03	0,82	0,03	0,82	0,02
138687		1,19	0,05	1,21	0,02	1,33	0,01	0,95	0,24	0,95	0,25	1,01	0,78	0,64	0,00	0,87	0,00	0,71	0,00	0,84	0,01	0,73	0,00	0,82	0,00	0,97	0,36	0,80	0,00	0,72	0,00
139104		0,83	0,00	0,84	0,00	0,82	0,01	0,91	0,04	0,82	0,00	0,77	0,00	0,59	0,00	0,76	0,00	0,48	0,00	1,19	0,01	1,09	0,09	1,07	0,27	1,39	0,00	1,02	0,78	0,90	0,16
140457		1,06	0,80	0,81	0,45	0,58	0,10	1,05	0,83	0,84	0,43	0,66	0,13	1,14	0,32	0,90	0,47	0,74	0,01	0,97	0,86	0,72	0,13	1,01	0,96	0,75	0,36	1,11	0,66	0,85	0,48
141431		0,57	0,18	0,51	0,14	0,38	0,11	1,00	1,00	0,54	0,05	0,33	0,01	0,98	0,93	0,74	0,30	0,43	0,03	1,66	0,17	1,55	0,14	1,01	0,96	1,32	0,39	0,84	0,33	1,04	0,91
142473		0,69	0,00	0,64	0,00	0,45	0,00	0,72	0,02	0,64	0,01	0,66	0,01	0,67	0,00	0,79	0,00	0,48	0,00	1,04	0,40	0,95	0,22	1,00	0,98	1,19	0,04	1,49	0,01	1,04	0,52
144537	SUGT1,NUBP1	0,71	0,00	0,70	0,00	0,53	0,00	0,79	0,00	0,74	0,00	0,55	0,00	0,77	0,00	0,78	0,00	0,62	0,00	0,95	0,09	0,86	0,00	0,91	0,03	0,89	0,01	0,90	0,13	0,93	0,09
145564		0,82	0,14	0,75	0,01	0,63	0,01	0,89	0,01	0,84	0,00	0,77	0,00	0,73	0,00	0,80	0,01	0,75	0,00	0,86	0,25	0,82	0,16	0,89	0,08	0,99	0,90	0,96	0,70	1,09	0,44
146603		0,96	0,47	0,96	0,32	1,11	0,02	0,81	0,01	0,87	0,03	0,82	0,01	0,93	0,13	1,07	0,18	1,08	0,16	0,79	0,00	0,71	0,00	0,86	0,01	0,83	0,01	0,69	0,00	0,72	0,00
147614		0,98	0,62	1,25	0,02	1,08	0,25	0,93	0,52	1,08	0,53	1,04	0,61	0,38	0,00	0,92	0,64	0,46	0,01	0,62	0,00	0,57	0,00	0,55	0,00	1,05	0,73	0,62	0,00	0,60	0,00
149908		0,89	0,06	0,89	0,06	0,72	0,01	0,99	0,55	0,82	0,00	0,79	0,00	0,91	0,02	0,95	0,25	0,77	0,00	1,03	0,37	1,04	0,20	0,85	0,01	1,09	0,07	1,02	0,79	1,09	0,16
152055	CATD,CLIC1	0,81	0,00	0,99	0,72	0,93	0,06	1,02	0,56	0,79	0,00	0,82	0,00	1,10	0,10	0,96	0,51	0,71	0,00	1,86	0,00	2,26	0,00	1,18	0,00	1,62	0,00	1,30	0,00	1,27	0,00
153119		0,93	0,31	0,98	0,74	0,73	0,00	1,08	0,21	0,92	0,12	0,83	0,01	1,19	0,00	1,02	0,49	0,86	0,01	1,35	0,00	1,52	0,00	1,09	0,16	1,25	0,01	1,33	0,00	1,41	0,00
154183		1,02	0,82	1,03	0,62	0,75	0,02	1,18	0,00	0,83	0,00	0,88	0,01	0,92	0,06	1,05	0,08	0,77	0,00	1,25	0,02	1,06	0,48	0,87	0,02	1,21	0,00	1,26	0,01	1,22	0,02
155439		0,95	0,41	1,15	0,01	0,74	0,00	0,94	0,32	1,05	0,25	0,91	0,13	0,62	0,00	0,91	0,33	0,65	0,01	0,86	0,07	0,70	0,00	0,79	0,00	0,85	0,01	1,07	0,32	0,95	0,48
156308		0,64	0,00	0,52	0,00	0,38	0,00	0,81	0,04	0,84	0,07	0,63	0,00	0,79	0,00	0,95	0,12	0,71	0,00	0,98	0,38	0,80	0,00	1,25	0,00	1,19	0,00	1,29	0,00	1,24	0,00
157378		0,88	0,02	0,87	0,01	0,67	0,00	0,92	0,24	1,03	0,72	0,69	0,00	0,91	0,02	1,07	0,13	0,94	0,07	0,87	0,01	0,64	0,00	0,98	0,55	0,76	0,00	0,86	0,00	0,87	0,00
158651		0,68	0,01	0,57	0,00	0,52	0,00	0,81	0,00	0,59	0,00	0,50	0,00	0,85	0,27	0,64	0,08	0,54	0,01	1,32	0,02	1,05	0,61	1,15	0,12	0,95	0,80	1,07	0,45	0,88	0,29
159535		0,66	0,00	0,66	0,00	0,30	0,00	0,84	0,02	0,67	0,00	0,61	0,00	0,61	0,00	0,84	0,04	0,58	0,00	1,30	0,01	0,91	0,21	1,05	0,60	1,25	0,03	2,08	0,00	1,88	0,01
160578		1,04	0,46	1,01	0,94	0,95	0,44	0,80	0,24	0,79	0,18	0,70	0,10	1,25	0,07	1,01	0,94	1,20	0,19	1,08	0,49	1,11	0,22	1,10	0,20	0,92	0,25	1,04	0,79	1,16	0,28
161872		1,21	0,00	1,06	0,12	0,91	0,01	1,12	0,00	0,83	0,00	0,85	0,00	0,85	0,01	1,07	0,17	0,84	0,01	1,02	0,49	0,88	0,00	0,86	0,00	1,27	0,00	1,01	0,67	1,15	0,00
163898	CBX1	0,86	0,00	0,85	0,00	0,70	0,00	0,92	0,00	0,76	0,00	0,71	0,00	0,74	0,00	0,81	0,00	0,64	0,00	0,92	0,00	0,76	0,00	0,78	0,00	0,85	0,00	0,88	0,01	0,82	0,00
165075		0,94	0,47	0,90	0,32	0,78	0,14	1,09	0,56	0,63	0,01	0,61	0,01	1,16	0,03	0,92	0,06	0,94	0,38	1,19	0,22	0,98	0,80	0,72	0,03	0,81	0,10	0,80	0,25	0,96	0,82
166201		0,94	0,28	0,89	0,07	0,72	0,02	1,51	0,00	0,85	0,08	1,05	0,52	1,01	0,75	1,23	0,00	0,96	0,30	1,56	0,00	1,48	0,00	0,92	0,25	1,90	0,00	1,41	0,02	1,82	0,00
167310		0,78	0,00	0,88	0,02	0,57	0,00	0,83	0,01	0,76	0,00	0,62	0,00	0,69	0,00	0,87	0,00	0,73	0,00	0,99	0,89	0,73	0,00	0,81	0,00	0,83	0,00	1,02	0,81	1,07	0,34
168476		0,70	0,00	0,80	0,07	0,52	0,00	0,83	0,04	0,64	0,00	0,44	0,00	0,86	0,03	0,77	0,01	0,72	0,00	1,12	0,26	0,98	0,81	0,76	0,08	0,77	0,10	0,79	0,21	1,11	0,50
169541		1,05	0,06	1,06	0,02	0,83	0,00	0,91	0,04	0,77	0,00	0,52	0,00	0,58	0,00	0,91	0,29	0,48	0,00	0,70	0,00	0,42	0,00	0,59	0,00	0,65	0,00	0,51	0,00	0,44	0,00
170666		0,70	0,01	0,86	0,14	1,17	0,15	0,92	0,33	0,85	0,08	1,02	0,84	0,88	0,02	1,03	0,56	0,74	0,00	1,56	0,00	1,58	0,00	1,18	0,03	1,51	0,00	1,04	0,65	0,80	0,04
172969	PSA3,TPD54	0,94	0,04	0,84	0,00	0,69	0,00	0,97	0,51	0,82	0,00	0,76	0,00	0,92	0,03	0,98	0,66	0,80	0,00	0,99	0,76	0,99	0,45	0,94	0,04	1,17	0,01	1,05	0,24	1,17	0,00
174420	ACTG	1,03	0,05	1,14	0,01	0,91	0,01	1,06	0,01	1,02	0,21	0,93	0,01	0,96	0,10	1,23	0,00	1,05	0,06	0,87	0,00	0,78	0,00	0,76	0,00	0,92	0,06	0,86	0,01	0,98	0,43
176254	PDIA6,F10A1,PABP2,PRS6B	0,94	0,02	0,97	0,13	0,82	0,00	0,99	0,44	0,81	0,00	0,77	0,00	0,88	0,00	0,98	0,41	0,77	0,00	1,07	0,01	0,95	0,01	0,85	0,00	1,04	0,05	0,95	0,01	0,96	0,00
177384	PR56A,TXND5	0,87	0,01	0,86	0,00	0,69	0,00	0,88	0,00	0,75	0,00	0,66	0,00	0,83	0,00	0,89	0,05	0,72	0,00	0,95	0,08	0,80	0,00	0,83	0,00	0,88	0,02	0,91	0,06	0,88	0,02
178566		0,65	0,00	0,47	0,00	0,21	0,00	0,81	0,01	0,53	0,00	0,48	0,00	1,21	0,03	0,99	0,84	0,97	0,70	1,07	0,20	0,91	0,21	0,98	0,87	1,04	0,77	1,95	0,00	2,22	0,01
181505		1,09	0,03	1,33	0,01	1,31	0,00	1,07	0,42	1,25	0,01	1,13	0,14	1,34	0,00	1,22	0,01	1,14	0,01	1,04	0,21	1,09	0,01	1,01	0,86	0,83	0,05	0,92	0,09	0,77	0,00
182136		1,02	0,69	0,81	0,00	0,55	0,00	0,64	0,00	0,75	0,00	0,52	0,00	0,79	0,02	0,93	0,43	0,80	0,03	0,65	0,00	0,73	0,00	0,96	0,38	1,08	0,29	0,97	0,73	1,36	0,01
183880		0,78	0,00	0,72	0,00	0,46	0,00	0,75	0,00	0,64	0,00	0,57	0,00	0,74	0,00	0,84	0,01	0,63	0,00	1,05	0,35	0,98	0,64	0,97	0,46	1,21	0,01	1,35	0,02	1,41	0,02
185058		1,13	0,00	1,31	0,00	1,03	0,08	0,93	0,00	0,86	0,00	0,90	0,00	0,72	0,00	0,92	0,01	0,70	0,00	0,87	0,00	0,76	0,00	0,69	0,00	0,83	0,00	0,92	0,00	0,81	0,00
185509		0,82	0,00	0,94	0,05	0,78	0,00	0,79	0,01	0,70	0,00	0,68	0,00	0,54	0,00	0,71	0,00	0,56	0,00	0,94	0,08	0,74	0,00	0,73	0,00	0,85	0,01	0,85	0,01	0,80	0,00
186642		0,95	0,52	1,17	0,06	1,12	0,17	0,94	0,05	0,84	0,02	0,79	0,00	0,57	0,00	1,12	0,21	0,53	0,00	1,07	0,28	1,04	0,59	0,78	0,01	1,64	0,00	0,76	0,01	0,80	0,00
187834	KBP	0,92	0,56	1,10	0,39	1,08	0,54	0,85	0,00	0,71	0,00	0,65	0,00	0,51	0,00	0,91	0,14	0,40	0,00	1,27	0,04	1,23	0,07	0,89	0,24	1,84	0,00	0,83	0,11	0,83	0,01
189820		1,16	0,00	1,21	0,01	1,08	0,03	1,05	0,06	1,01	0,57	0,99	0,71	0,75	0,02	1,03	0,68	0,83	0,05	0,85	0,00	0,71	0,00	0,79	0,00	0,93	0,18	0,87	0,00	0,83	0,00
191012	HNRPK,TBL1R,HMCS1,TBA3	0,89	0,00	0,92	0,09	0,71	0,00	0,90	0,00	0,78	0,00	0,69	0,00	0,75	0,00	0,93	0,08	0,64	0,00	0,98	0,11	0,88	0,00	0,82	0,01	1,04	0,34	0,93	0,21	0,93	0,23
191574	FKB10	0,91	0,05	1,09	0,07	0,76	0,00	0,94	0,30	0,78	0,00	0,67	0,00	0,68	0,02	0,81	0,17	0,73	0,05	1,18	0,01	0,90	0,01	0,81	0,00	0,90	0,19	1,00	0,94	1,17	0,02
193389		0,99	0,93	1,22	0,05	1,30	0,02	1,00	0,93	0,87	0,03	0,95	0,26	0,84	0,01	1,07	0,17	0,68	0,00	1,05	0,54	1,08	0,30	0,75	0,00	1,12	0,04	0,76	0,00	0,66	0,00
193636		0,74	0,00	0,73	0,00	0,52	0,00	0,79	0,01	0,72	0,00	0,64	0,00	0,68	0,00	0,85	0,00	0,63	0,00	1,03	0,35	0,96	0,02	0,96	0,26	1,21	0,00	1,18	0,00	1,23	0,00
195754	ITA3	0,94	0,01	1,05	0,05	1,40	0,00	0,92	0,00	0,75	0,00	0,74	0,00	0,91	0,01	1,03	0,23	0,67	0,00	1,14	0,00	1,12	0,01	0,83	0,00	1,12	0,00	0,61	0,00	0,55	0,00
196961		0,80	0,00	0,72	0,00	0,54	0,00	0,81	0,02	0,69	0,00	0,66	0,00	0,60	0,00	0,82	0,00	0,58	0,00	0,93	0,01	0,87	0,00	0,88	0,02	1,33	0,00	1,11	0,04	1,26	0,00
197182	MVP,UBA1	0,69	0,00	0,69	0,00	0,46	0,00	0,73	0,00	0,61	0,00	0,59	0,00	0,64	0,00	0,77	0,00	0,59	0,00	1,20	0,00	1,12	0,01	1,00	0,86	1,34	0,00	1,45	0,00	1,54	0,00
198671	ERO1A	0,97	0,33	0,97	0,70	1,11	0,29	0,95	0,49	0,80	0,01	0,75	0,00	0,84	0,00	0,96	0,26	0,70	0,00	1,04	0,48	0,94	0,01	0,88	0,16	1,07	0,40	0,72	0,01	0,69	0,01
200032		1,16	0,04	1,09	0,60	1,04	0,55	1,00	0,97	0,66	0,00	0,83	0,10	0,73	0,00	0,97	0,73	0,64	0,00	1,09	0,07	0,87	0,01	0,77	0,15	1,24	0,16	1,00	0,99	0,84	0,01
201269	UQCR1,HNRPK,RUVB2	0,63	0,00	0,56	0,05	0,55	0,02	0,55	0,00	0,58	0,00	0,41	0,00	0,49	0,00	0,70	0,00	0,48	0,00	0,88	0,17	0,73	0,00	1,05	0,89	1,19	0,59	0,76	0,39	0,84	0,56
202423	K2C8	1,22	0,14	1,14	0,25	0,84	0,18	1,10	0,20	1,34	0,00	0,91	0,23	1,04	0,94	0,55	0,42	0,70	0,57	0,99	0,94	1,69	0,01	1,30	0,02	0,96	0,88	1,19	0,17	1,67	0,01
203683	TXND5,ACL6A	1,58	0,00	2,39	0,00	2,80	0,00	1,19	0,00	1,28	0,00	1,38	0,00	1,27	0,00	1,80	0,00	1,69	0,00	0,75	0,00	0,69	0,00	0,53	0,00	0,65	0,00	0,49	0,00	0,52	0,00
204237	M6PBP,IF35,HNRPF	1,14	0,00	1,13	0,01	1,05	0,22	0,97	0,15	1,00	0,99	0,82	0,00	1,05	0,05	1,05	0,02	0,95	0,04	0,80	0,00	0,79	0,00	0,83	0,00	0,79	0,00	0,73	0,00	0,77	0,00
206082		1,06	0,10	1,04	0,17	0,84	0,01	1,02	0,33	0,87	0,00	0,78	0,00	0,83	0,00	1,02	0,64	0,82	0,00	0,95	0,15	0,83	0,00	0,82	0,00	1,03	0,41	0,92	0,12	1,03	0,47
207376	KCRB	0,77	0,00	0,79	0,02	0,68	0,00	0,73	0,01	0,54	0,00	0,59	0,00	0,54	0,00	0,62	0,00	0,36	0,00	0,80	0,04	0,68	0,00	0,58	0,00	0,77	0,03	0,73	0,00	0,52	0,00
209100	ACTG,ACTB	0,76	0,00	0,99	0,95	0,87	0,43	0,88	0,14	0,86	0,15	0,74	0,00	0,48	0,00	0,73	0,01	0,63	0,00	1,01	0,92	0,65	0,00	0,76	0,27	0,75	0,25	0,75	0,22	0,74	0,19
210320		0,90	0,03	1,01	0,62	0,79	0,00	0,91	0,09	0,93	0,07	0,68	0,00	0,64	0,00	0,91	0,09	0,69	0,00	0,87	0,03	0,68	0,00	0,79	0,00	0,85	0,00	0,74	0,00	0,84	0,00
211213		1,52	0,00	1,64	0,00	2,49	0,00	1,24	0,05	0,99	0,89	1,02	0,83	1,10	0,14	1,09	0,00	1,10	0,02	0,87	0,08	0,71	0,00	0,64	0,00	0,65	0,00	0,43	0,00	0,43	0,00
212449		2,82	0,00	3,73	0,00	4,80	0,00	3,33	0,00	2,69	0,00	2,80	0,00	4,75	0,00	6,54	0,00	5,05	0,00	1,15	0,16	1,01	0,95	0,70	0,04	1,05	0,76	0,57	0,02	0,63	0,03
213992	ANXA5	0,76	0,00	0,84	0,00	0,57	0,00	0,79	0,00	0,73	0,00	0,63	0,00	0,65	0,00	0,66	0,00	0,56	0,00	0,99	0,69	0,86	0,00	0,82	0,00	0,80	0,00	1,06	0,27	0,99	0,89
214766	NPM	1,02	0,36	0,81	0,01	0,75	0,00	1,06	0,19	0,84	0,01	0,77	0,00	0,99	0,83	1,03	0,43	1,01	0,91	0,92	0,02	0,85	0,00	0,92	0,23	1,11	0,13	0,91	0,31	1,18	0,17
216132		1,22	0,11	0,77	0,00	0,75	0,06	1,08	0,49	0,85	0,09	1,05	0,69	0,86	0,04	1,09	0,29	0,84	0,16	1,09	0,55	1,14	0,21	1,37	0,02	2,31	0,00	1,72	0,04	1,81	0,03
217409	HEXB	0,97	0,10	0,97	0,50	0,92	0,15	0,92	0,04	0,81	0,00	0,71	0,00	0,89	0,00	0,83	0,00	0,66	0,00	1,02	0,65	1,07	0,00	0,89	0,04	0,99	0,83	0,82	0,02	0,84	0,02
218682		0,71	0,00	0,73	0,01	0,53	0,00	0,75	0,00	0,75	0,00	0,59	0,00	0,63	0,00	0,72	0,01	0,52	0,00	0,97	0,44	0,91	0,02	0,95	0,59	1,01	0,91	1,04	0,72	1,01	0,95
219940		1,52	0,00	1,66	0,00	2,19	0,00	1,28	0,00	1,20	0,02	1,24	0,00	1,40	0,00	1,27	0,00	1,21	0,02	0,86	0,01	0,87	0,01	0,74	0,00	0,72	0,00	0,58	0,00	0,52	0,00
221306		1,01	0,91	0,86	0,53	1,07	0,59	1,21	0,18	0,56	0,01	0,70	0,05	1,00	0,93	0,97	0,60	0,73	0,01	1,19	0,08	0,92	0,08	0,64	0,21	1,06	0,82	0,64	0,03	0,63	0,03
223745	MTND	0,82	0,09	1,10	0,53	0,87	0,56	0,79	0,17	0,86	0,23	0,63	0,02	1,05	0,46	0,82	0,04	0,96	0,53	1,01	0,94	0,93	0,33	0,82	0,18	0,54	0,01	0,77	0,36	0,81	0,43
226058		1,14	0,19	0,98	0,75	1,09	0,28	1,10	0,00	0,89	0,06	0,85	0,00	1,02	0,57	1,07	0,10	0,91	0,03	1,03	0,64	0,89	0,13	0,97	0,65	1,08	0,10	0,83	0,00	0,83	0,01
228572	PSME2	0,86	0,07	0,74	0,01	0,68	0,01	0,87	0,03	0,82	0,01	0,73	0,00	0,88	0,01	0,95	0,15	0,74	0,00	1,05	0,36	0,96	0,31	1,13	0,08	1,20	0,00	1,09	0,09	1,01	0,87

229688		0,81	0,45	0,97	0,44	0,94	0,63	1,45	0,01	1,26	0,07	1,21	0,23	1,39	0,00	1,21	0,00	1,23	0,00	1,42	0,19	1,21	0,48	1,04	0,59	0,88	0,04	1,03	0,88	0,93	0,62
230824	PDIA1	1,09	0,37	1,14	0,12	1,12	0,20	0,79	0,02	0,79	0,01	0,69	0,00	0,89	0,08	1,06	0,25	0,72	0,00	1,02	0,83	1,13	0,21	0,98	0,74	1,30	0,00	0,87	0,10	0,90	0,10
232052		1,19	0,03	5,39	0,35	1,41	0,24	0,75	0,56	0,98	0,96	1,04	0,90	1,12	0,38	1,17	0,11	0,90	0,31	0,54	0,03	0,65	0,01	0,16	0,33	0,15	0,33	0,63	0,14	0,44	0,05
233300		0,76	0,23	0,91	0,12	0,77	0,03	0,94	0,02	0,99	0,53	0,81	0,00	0,91	0,00	0,95	0,08	0,91	0,03	1,04	0,86	0,93	0,79	0,91	0,16	0,81	0,01	0,88	0,30	0,92	0,46
234358	PDIA3,PEPD	1,24	0,07	1,64	0,00	2,09	0,00	1,25	0,00	1,50	0,00	2,07	0,00	1,16	0,00	1,54	0,00	1,44	0,00	0,89	0,25	0,85	0,15	0,80	0,02	0,85	0,06	0,88	0,07	0,63	0,00
235608	COR1B	0,90	0,00	1,01	0,84	1,02	0,62	0,84	0,00	0,82	0,00	1,09	0,02	0,65	0,00	0,79	0,00	0,75	0,00	1,03	0,04	0,94	0,00	0,90	0,00	1,02	0,36	1,18	0,00	0,96	0,13
237648		1,04	0,83	2,48	0,04	3,20	0,01	1,53	0,00	2,37	0,00	3,24	0,00	0,95	0,51	1,26	0,01	1,22	0,03	1,04	0,81	1,26	0,21	0,68	0,21	0,71	0,24	0,72	0,15	0,53	0,03
238040		1,07	0,68	1,50	0,01	1,86	0,00	1,19	0,00	1,47	0,00	1,86	0,00	1,17	0,01	1,32	0,00	1,35	0,00	1,03	0,82	1,06	0,67	0,91	0,21	0,86	0,07	0,93	0,29	0,70	0,01
240147		0,47	0,00	0,38	0,00	0,14	0,00	0,59	0,00	0,51	0,00	0,36	0,00	0,40	0,00	0,52	0,00	0,33	0,00	1,07	0,26	0,89	0,04	1,14	0,31	1,41	0,01	2,12	0,03	2,40	0,00
240305		0,55	0,00	0,47	0,00	0,17	0,00	0,67	0,00	0,58	0,00	0,44	0,00	0,53	0,00	0,65	0,00	0,45	0,00	1,11	0,10	0,90	0,01	1,12	0,21	1,29	0,01	2,34	0,01	2,46	0,00
242682		0,65	0,00	0,59	0,00	0,20	0,00	0,73	0,00	0,67	0,00	0,50	0,00	0,54	0,00	0,65	0,00	0,46	0,00	1,04	0,41	0,84	0,01	1,04	0,65	1,11	0,32	2,25	0,00	2,32	0,01
242868		0,80	0,00	0,79	0,00	0,83	0,00	0,81	0,00	0,79	0,00	0,69	0,00	0,76	0,00	0,86	0,00	0,68	0,00	0,95	0,07	0,96	0,17	0,93	0,01	1,10	0,01	0,78	0,00	0,82	0,01
244116	DPOD2	0,36	0,00	0,20	0,00	0,02	0,00	0,42	0,00	0,29	0,00	0,07	0,00	0,36	0,00	0,44	0,00	0,17	0,00	1,32	0,08	1,17	0,12	1,60	0,22	2,52	0,00	3,48	0,21	8,63	0,04
245554		1,06	0,79	0,57	0,02	0,25	0,01	1,11	N,A,	13,03	N,A,	2,41	N,A,	0,30	0,01	1,13	0,07	1,88	0,01	0,36	N,A,	0,08	0,00	7,78	0,20	0,55	0,00	3,27	0,24	2,09	0,01
246907		0,35	0,54	0,61	0,70	0,80	0,87	1,17	0,03	1,83	0,00	2,67	0,00	1,25	0,06	1,59	0,00	1,95	0,00	1,05	0,96	1,01	0,99	0,93	0,53	0,74	0,03	1,05	0,65	0,69	0,03
249066		1,05	0,86	2,06	0,02	2,53	0,01	1,22	0,02	1,64	0,00	2,34	0,01	1,44	0,02	1,86	0,04	1,87	0,05	1,13	0,50	1,29	0,14	0,77	0,14	0,85	0,45	0,89	0,57	0,70	0,12
250337		1,40	0,02	1,91	0,00	2,31	0,01	1,72	0,00	2,42	0,00	3,70	0,00	1,00	0,96	0,97	0,80	1,43	0,01	0,81	0,07	0,86	0,16	0,83	0,09	0,61	0,00	1,06	0,69	0,75	0,10
250849		1,10	0,07	0,93	0,19	0,96	0,50	1,12	0,01	1,31	0,00	1,39	0,00	1,19	0,08	1,16	0,16	1,26	0,05	0,76	0,00	0,79	0,00	1,04	0,30	0,91	0,12	1,08	0,22	0,95	0,52
254557		1,06	0,25	1,21	0,00	1,46	0,00	0,83	0,08	0,99	0,92	1,32	0,01	0,68	0,00	0,76	0,00	0,74	0,00	0,90	0,08	0,94	0,16	0,95	0,16	0,93	0,26	1,05	0,43	0,75	0,00
257339		1,05	0,45	0,98	0,81	0,75	0,01	1,07	0,24	0,66	0,00	0,60	0,00	0,84	0,01	1,03	0,03	0,64	0,00	1,11	0,13	0,75	0,01	0,74	0,01	0,99	0,85	0,88	0,20	0,80	0,06
258601		0,97	0,90	0,77	0,33	0,44	0,10	1,45	0,06	0,72	0,14	0,56	0,00	0,54	0,05	1,01	0,97	0,57	0,05	0,89	0,52	0,74	0,26	0,56	0,04	1,76	0,01	0,74	0,45	1,71	0,16
261244		0,89	0,42	0,66	0,02	0,54	0,01	0,69	0,00	0,26	0,00	0,25	0,00	0,67	0,00	0,84	0,01	0,48	0,00	1,87	0,00	1,04	0,70	0,95	0,77	1,76	0,00	1,11	0,53	1,23	0,20
262545		0,88	0,00	0,78	0,01	0,64	0,00	0,77	0,01	0,48	0,00	0,45	0,00	0,80	0,07	0,92	0,33	0,64	0,00	1,25	0,00	0,87	0,10	0,88	0,26	1,14	0,11	1,00	0,96	0,96	0,64
265686		1,35	0,06	1,68	0,02	1,86	0,04	1,27	0,12	1,79	0,00	2,34	0,00	2,97	0,01	2,54	0,00	3,37	0,00	0,87	0,07	1,10	0,61	0,98	0,84	0,75	0,04	1,16	0,35	0,90	0,64
266132		1,37	0,00	1,48	0,00	1,56	0,00	1,17	0,18	1,33	0,01	1,81	0,00	1,98	0,00	1,85	0,00	2,13	0,00	0,91	0,29	0,96	0,56	0,96	0,41	0,83	0,00	1,24	0,02	0,90	0,50
267419		1,14	0,66	1,23	0,50	0,99	0,98	0,68	0,41	0,71	0,46	0,84	0,68	2,36	0,00	1,69	0,00	2,15	0,00	0,92	0,69	0,84	0,45	0,90	0,61	0,56	0,05	1,31	0,28	0,89	0,67
268600		0,98	0,94	1,84	0,01	1,73	0,02	1,26	0,28	1,14	0,50	1,06	0,76	0,36	0,00	0,89	0,00	0,40	0,00	0,93	0,71	0,72	0,15	0,45	0,00	0,94	0,14	0,45	0,00	0,45	0,00
269855		1,80	0,02	1,87	0,01	2,48	0,00	1,25	0,19	1,04	0,79	1,04	0,81	0,83	0,02	1,34	0,00	0,88	0,08	1,00	0,98	0,69	0,04	0,80	0,16	1,06	0,62	0,60	0,00	0,53	0,00
274688	ENPL	1,37	0,00	1,52	0,00	1,85	0,00	1,36	0,00	1,06	0,46	1,09	0,35	0,77	0,00	1,09	0,02	0,83	0,00	0,89	0,09	0,62	0,00	0,62	0,00	0,79	0,00	0,53	0,00	0,49	0,00
278067	SPRC	0,99	0,44	0,98	0,42	1,07	0,03	0,78	0,00	0,71	0,00	0,64	0,00	0,62	0,00	0,72	0,00	0,53	0,00	0,76	0,00	0,72	0,00	0,70	0,00	0,84	0,00	0,58	0,00	0,57	0,00
279386	SET	1,04	0,19	0,96	0,20	0,71	0,00	1,05	0,03	0,86	0,00	0,80	0,00	0,81	0,00	0,92	0,01	0,75	0,00	0,93	0,01	0,74	0,00	0,82	0,00	0,91	0,01	1,04	0,27	1,01	0,80
280677	RCN1	1,47	0,00	1,76	0,00	2,03	0,00	1,33	0,00	1,14	0,01	1,16	0,01	0,88	0,05	1,07	0,31	0,90	0,04	1,02	0,51	0,89	0,02	0,73	0,00	0,90	0,09	0,64	0,00	0,66	0,00
281947		1,44	0,00	1,64	0,00	1,25	0,00	1,15	0,07	1,18	0,01	1,15	0,07	1,06	0,33	1,26	0,02	0,94	0,45	0,69	0,00	0,69	0,00	0,62	0,00	0,72	0,01	0,79	0,01	0,70	0,00
283779		0,90	0,01	0,86	0,00	0,70	0,00	0,75	0,00	0,79	0,00	0,59	0,00	0,53	0,00	0,77	0,03	0,58	0,01	0,76	0,00	0,63	0,00	0,83	0,00	0,95	0,48	0,76	0,00	0,88	0,45
288865		1,18	0,00	1,17	0,02	1,14	0,11	1,32	0,05	1,03	0,83	0,96	0,78	1,03	0,67	1,65	0,00	1,25	0,02	1,29	0,01	0,94	0,11	1,00	0,94	1,50	0,00	0,97	0,64	1,17	0,08
290046		0,74	0,00	0,82	0,01	0,69	0,01	0,95	0,54	1,04	0,37	1,13	0,05	0,71	0,00	0,88	0,00	0,67	0,00	1,19	0,08	1,22	0,00	1,17	0,03	1,38	0,00	1,51	0,01	1,25	0,09
294218		0,86	0,17	0,88	0,08	0,68	0,01	0,83	0,00	0,71	0,00	0,59	0,00	1,00	0,85	0,79	0,00	0,82	0,00	1,11	0,27	0,98	0,84	0,92	0,02	0,75	0,00	1,00	0,94	1,02	0,73
295167	COPE,ANXA5	0,83	0,00	0,98	0,22	0,81	0,00	0,89	0,00	0,83	0,00	0,72	0,00	0,70	0,00	0,87	0,00	0,68	0,00	0,98	0,08	0,82	0,00	0,78	0,00	0,86	0,00	0,82	0,00	0,82	0,00
296837		0,90	0,39	1,35	0,01	0,99	0,91	0,96	0,81	0,84	0,29	0,74	0,10	0,58	0,00	0,86	0,00	0,75	0,00	1,06	0,63	0,73	0,04	0,62	0,00	0,72	0,01	0,74	0,02	0,86	0,14
299459		1,57	0,00	1,72	0,00	2,53	0,00	1,49	0,00	1,20	0,04	1,38	0,01	2,57	0,00	2,79	0,00	2,74	0,00	0,97	0,62	0,84	0,05	0,71	0,00	0,83	0,03	0,56	0,01	0,56	0,01
300154		0,50	0,00	0,47	0,00	0,23	0,00	0,55	0,02	0,45	0,01	0,24	0,00	0,73	0,01	0,61	0,00	0,42	0,00	1,13	0,45	0,88	0,37	0,96	0,83	0,77	0,14	1,07	0,15	1,09	0,52
302071	ATPB	1,77	0,00	1,99	0,00	3,68	0,00	1,42	0,00	1,06	0,31	1,14	0,04	0,85	0,01	0,99	0,72	0,74	0,00	0,76	0,00	0,54	0,00	0,51	0,00	0,56	0,00	0,30	0,00	0,23	0,00
302775	DCTN2,PABP2,PRS6A	0,83	0,00	0,85	0,01	0,71	0,00	0,92	0,00	0,81	0,00	0,71	0,00	0,78	0,00	0,92	0,00	0,74	0,00	0,99	0,61	0,84	0,00	0,86	0,01	0,96	0,41	0,90	0,04	0,93	0,10
303936		1,13	0,25	1,12	0,40	1,46	0,04	0,94	0,52	0,80	0,02	1,17	0,12	0,77	0,00	1,19	0,01	0,87	0,14	0,90	0,27	0,88	0,13	0,78	0,05	1,39	0,01	0,86	0,25	0,78	0,10
305540	HNRPC,STRAP	0,83	0,12	1,00	0,97	1,21	0,06	0,70	0,00	0,76	0,01	0,62	0,00	1,44	0,06	1,51	0,15	1,35	0,13	0,80	0,09	0,92	0,44	0,71	0,02	0,80	0,23	0,49	0,00	0,60	0,00
306795	SET,HDGF	0,66	0,00	0,56	0,00	0,21	0,00	0,56	0,00	0,50	0,00	0,46	0,00	0,67	0,09	0,55	0,03	0,59	0,04	1,04	0,72	0,78	0,02	1,09	0,40	0,75	0,02	2,68	0,00	2,15	0,00
309937	GRAP1,GANAB	0,64	0,00	0,63	0,00	0,34	0,00	0,85	0,19	0,77	0,07	0,54	0,01	0,67	0,00	0,81	0,00	0,65	0,00	0,94	0,46	0,72	0,01	0,86	0,11	0,87	0,07	1,12	0,36	1,30	0,02
311244		0,94	0,03	0,68	0,00	0,49	0,00	1,10	0,20	0,86	0,09	0,92	0,25	0,66	0,00	0,95	0,27	0,62	0,00	0,92	0,07	0,81	0,00	0,98	0,87	1,60	0,00	1,45	0,00	1,45	0,00
311457		0,89	0,01	0,91	0,01	0,78	0,00	0,93	0,01	0,90	0,02	0,87	0,00	0,78	0,00	1,01	0,69	0,81	0,00	0,95	0,05	0,81	0,00	0,91	0,02	1,04	0,05	1,02	0,70	0,97	0,36
313877		0,72	0,00	0,78	0,01	0,30	0,00	0,66	0,00	0,60	0,00	0,60	0,00	0,43	0,17	0,58	0,30	0,51	0,23	1,07	0,47	0,93	0,34	0,90	0,16	1,15	0,10	2,31	0,00	2,61	0,00
315205		0,71	0,00	0,62	0,00	0,65	0,00	0,67	0,00	0,62	0,00	0,61	0,00	0,77	0,02	0,89	0,22	0,68	0,01	1,05	0,37	1,05	0,19	1,11	0,14	1,40	0,00	1,04	0,52	1,01	0,84
315424		0,81	0,00	0,58	0,00	0,62	0,00	0,69	0,00	0,63	0,00	0,66	0,00	0,66	0,15	0,81	0,41	0,62	0,12	0,97	0,23	0,96	0,26	1,24	0,15	1,66	0,00	1,22	0,07	1,18	0,02
317853		0,95	0,39	0,79	0,16	0,89	0,25	0,75	0,05	0,62	0,01	0,70	0,03	0,62	0,05	0,83	0,31	0,60	0,04	1,00	0,98	1,00	0,99	0,99	0,94	1,59	0,01	1,00	0,98	1,02	0,80
319168	OXRP	0,89	0,08	0,92	0,12	0,89	0,10	0,90	0,03	0,96	0,35	0,78	0,00	0,87	0,03	1,01	0,75	0,71	0,00	0,88	0,02	0,80	0,00	0,92	0,05	0,90	0,02	0,77	0,01	0,65	0,00
320497		0,83	0,00	0,72	0,00	0,58	0,00	0,84	0,08	0,62	0,00	0,58	0,00	0,48	0,00	0,59	0,01	0,37	0,00	1,14	0,03	0,80	0,00	0,96	0,61	1,14	0,16	1,11	0,25	0,90	0,20
320752		0,78	0,00	0,67	0,00	0,59	0,00	0,87	0,00	0,79	0,00	0,74	0,00	0,81	0,00	0,90	0,01	0,77	0,00	1,04	0,19	0,89	0,01	1,08	0,21	1,16	0,00	1,15	0,09	1,11	0,06
323167		0,77	0,00	0,71	0,00	0,61	0,00	0,80	0,00	0,67	0,00	0,63	0,00	0,73	0,00	0,84	0,00	0,64	0,00	1,12	0,00	0,98	0,27	1,03	0,51	1,22	0,00	1,10	0,05	1,07	0,12
324496		0,78	0,01	0,75	0,00	0,69	0,00	0,81	0,00	0,76	0,00	0,72	0,00	0,77	0,00	0,91	0,01	0,79	0,00	1,09	0,06	0,89	0,03	1,07	0,18	1,10	0,03	1,10	0,16	1,04	0,45
327583	DC1I2	0,95	0,15	1,01	0,89	1,02	0,84	0,93	0,05	0,75	0,00	0,73	0,00	0,74	0,00	0,92	0,00	0,67	0,00	1,05	0,09	0,89	0,00	0,81	0,01	1,05	0,33	0,78	0,02	0,75	0,01
328907		0,50	0,00	0,39	0,00	0,49	0,00	0,73	0,03	0,65	0,02	0,57	0,00	0,58	0,00	0,72	0,00	0,46	0,00	1,16	0,15	1,56	0,00	1,34	0,16	2,50	0,00	0,94	0,66	1,27	0,11
332534		1,27	0,00	1,32	0,00	1,23	0,00	1,05	0,07	0,89	0,02	1,14	0,02	0,80	0,00	1,09	0,01	0,89	0,00	0,79	0,00	0,69	0,00	0,64	0,00	0,91	0,07	0,88	0,05	0,80	0,00
333871		1,19	0,01	1,31	0,03	1,30	0,00	1,11	0,00	0,97	0,17	1,13	0,00	0,97	0,34	1,15	0,01	1,03	0,43	0,83	0,01	0,78	0,00	0,66	0,01	0,84	0,10	0,78	0,00	0,76	0,00
335202	HSP7C	0,68	0,01	0,44	0,00	0,28	0,00	0,77	0,00	0,63	0,00	0,60	0,00	0,39	0,00	0,75	0,10	0,35	0,00	1,22	0,13	1,14	0,37	1,54	0,08	3,38	0,00	2,32	0,02	2,46	0,01
336540		0,87	0,11	1,45	0,00	1,04	0,71	0,96	0,26	0,92	0,24	0,84	0,00	0,75	0,00	1,61	0,00	0,68	0,00	1,08	0,16	1,05	0,33	0,62	0,00	1,37	0,00	0,80	0,02	0,81	0,04
337908		1,15	0,23	1,22	0,04	2,33	0,00	1,93	0,00	2,37	0,00	2,98	0,00	1,14	0,46	1,26	0,18	1,71	0,02	0,72	0,01	0,63	0,00	0,83	0,01	0,65	0,00	0,55	0,01	0,47	0,01
338842		0,94	0,48	0,84	0,17	0,87	0,46	0,91	0,06	0,85	0,01	0,97	0,64	0,89	0,01	0,93	0,02	0,75	0,00	0,94	0,37	0,82	0,02	0,99	0,90	0,96	0,73	1,09	0,66	0,75	0,21
340352		0,72	0,02	0,93	0,59	0,71	0,20	0,82	0,10	0,90	0,26	0,77	0,07	0,80	0,02	0,92	0,18	0,67	0,01	1,22	0,07	1,06	0,54	1,04	0,71	0,95	0,68	1,15	0,60	0,90	0,72
341707	APT	1,83	0,29	0,22	0,03	0,19	0,05	1,07	0,57	0,76	0,00	0,81	0,01	1,11	0,23	0,74	0,03	0,95	0,55	0,15	0,06	0,14	0,05	0,89	0,77	0,77	0,56	1,11	0,71	1,16	0,59
343050		0,82	0,33	0,87	0,55	0,58	0,07	1,23	0,17	0,92	0,59	0,93	0,62	2,04	0,00	1,19	0,31	1,20	0,28	1,39	0,04	1,75	0,00	0,97	0,87	0,96	0,82	1,48	0,05	1,46	0,06
344430		1,63	0,00	1,15	0,33	1,42	0,01	1,05	0,51	1,08	0,31	0,81	0,05	1,22	0,01	1,03	0,62	0,92	0,10	0,82	0,04	0,87	0,05	1,19	0,18	1,04	0,72	0,72	0,02	0,76	0,00
345986		1,30	0,01	1,11	0,35	1,20	0,03	1,35	0,00	0,90	0,18	1,07	0,07	0,81	0,02	1,23	0,00	0,77	0,01	1,08	0,29	0,89	0,14	0,84	0,18	1,57	0,00	0,93	0,16	0,91	0,22
347052		1,00	0,88	1,18	0,00	0,98	0,36	0,94	0,03	0,89	0,00	0,86	0,00	0,76	0,00	0,98	0,16	0,72	0,00	0,96	0,06	0,93	0,00	0,76	0,00	1,01	0,75	0,90	0,01	0,90	0,01
349706	CLIC4	0,79	0,00	1,14	0,03	1,06	0,12	0,86	0,03	0,96	0,46	0,84	0,01	0,75	0,00	0,86	0,00	0,77	0,00	1,27	0,00	1,28	0,00	0,98	0,66	1,01	0,89	0,92	0,09	0,98	0,03
351055		1,07	0,24	1,02	0,73	1,79	0,01	1,18	0,00	0,95	0,34	1,00	0,97	0,74	0,00	0,93	0,08	0,65	0,00	1,01	0,74	0,87	0,03	0,86	0,01	1,14	0,00	0,51	0,00	0,46	0,00
352255		1,46	0,00	1,71	0,00	1,73	0,00	1,59	0,00	1,94	0,00	2,47	0,00	1,57	0,01	1,95	0,00	2,13	0,00	0,81	0,00	0,81	0,00	0,85	0,00	0,86	0,01	1,07	0,19	0,93	0,11
353686		0,81	0,00	0,61	0,03	0,46	0,02	1,04	0,54	0,67	0,00	0,77	0,01	0,77	0,00	0,89	0,02	0,51	0,00	1,19	0,01	1,19	0,02	1,03	0,91	1,81	0,01	1,57	0,16	1,38	0,34
355063		0,97	0,48	0,93	0,24	1,07	0,51	0,96	0,48	0,92	0,16	0,83	0,03	0,86	0,01	1,15	0,03	0,90	0,03	0,81	0,00	0,62	0,00	0,81	0,00	0,87	0,05	0,64	0,01	0,59	0,01
356244		0,96	0,80	0,93	0,40	1,02	0,87	1,20	0,04	0,98	0,79	0,99	0,87	0,92	0,25	1,02	0,62	0,86	0,02	1,02	0,89	0,84	0,33	0,86	0,07	0,96	0,54	0,79	0,12	0,73	0,06
357613	SAE1,ACTG,TMOD3	0,78	0,00	0,89	0,01	0,67	0,00	0,90	0,02	0,78	0,00	0,70	0,00	0,82	0,00	0,91	0,00	0,71	0,00	1,11	0,01	0,98	0,52	0,84	0,00	0,95	0,15	1,00	0,97	0,98	0,58
360270	ACTG,ACTB	1,19	0,03	1,03	0,78	1,00	0,97	1,00	0,98	0,77	0,08	0,74	0,04	0,73	0,00	0,94	0,39	0,87	0,06	0,73	0,01	0,45	0,00	0,65	0,01	0,68	0,01	0,65	0,00	0,64	0,00
362224	TXND5,ACL6A	0,80	0,00	1,00	0,97	0,88	0,03	0,73	0,00	0,84	0,01	0,70	0,00	0,80	0,00	0,93	0,10	0,84	0,00	0,87	0,00	0,79	0,00	0,80	0,00	0,74	0,00	0,76	0,00	0,76	0,00
362785		0,75	0,00	0,70	0,04	0,96	0,90	0,87	0,11	0,90	0,26	0,79	0,04	0,74	0,00	0,85	0,00	0,76	0,00	0,92	0,05	0,83	0,00	1,01	0,96	1,02	0,89	0,65	0,35	0,67	0,37
363557	HS90A	0,77	0,01	0,78	0,02	0,91	0,39	0,83	0,14	0,81	0,28	0,54	0,00	0,54	0,00	0,82	0,02	0,48	0,00	0,86	0,10	0,87	0,18	0,83	0,30	1,32	0,01	0,47	0,00	0,66	0,01
364919		0,63	0,00	0,81	0,03	0,72	0,01	0,88	0,02	0,59	0,00	0,58	0,00	0,64	0,00	0,76	0,00	0,49	0,00	1,42	0,00	1,10	0,22	0,74	0,02	1,02	0,79	0,81	0,03	0,74	0,01
366771	GRP78	1,21	0,08	0,94	0,59	0,60	0,01	0,70	0,00	0,61	0,00	0,59	0,00	0,35	0,00	0,49	0,00	0,23	0,00	0,71	0,00	0,74	0,01	0,80	0,06	1,37	0,02	1,21	0,10	0,99	0,94
369021		0,94	0,06	0,92	0,28	0,80	0,01	0,84	0,05	0,67	0,00	0,60	0,00	0,70	0,00	0,81	0,02	0,68	0,00	0,89	0,03	0,77	0,00	0,73	0,02	0,91	0,32	0,74	0,02	0,87	0,11
370380	PDIA4	1,08	0,02	1,24	0,00	1,17	0,01	1,07	0,00	1,08	0,01	1,42	0,00	1,14	0,18	1,38	0,01	1,59	0,00	0,90	0,00	0,82	0,00	0,79	0,00	0,88	0,02	1,10	0,03	1,06	0,07
371745	PLST,AMPM2,CPNE1,COR1B	0,81	0,00	0,96	0,18	0,88	0,01	0,93	0,01	0,89	0,00	1,22	0,00	0,68	0,00	0,85	0,00	0,84	0,00	1,06	0,04	0,97	0,31	0,87	0,00	1,04	0,17	1,28	0,00	1,11	0,00
373121	QCR1	0,80	0,00	0,84	0,02	0,56	0,00	0,77	0,00	0,69	0,00	0,60	0,00	0,64	0,00	0,81	0,00	0,65	0,00	0,93	0,06	0,81	0,00	0,79	0,01	0,97	0,67	1,02	0,72	1,16	0,01
373664		0,84	0,01	0,62	0,01	0,58	0,01	0,83	0,03	0,60	0,00	0,55	0,00	0,77	0,00	0,85	0,00	0,62	0,00	0,99	0,87	0,95	0,26	0,97	0,86	1,41	0,03	0,96	0,84	1,11	0,52
375839		0,93	0,12	1,03	0,46	0,99	0,77	0,94	0,48	0,82	0,06	1,02	0,79	1,15	0,14	1,40	0,02	1,21	0,05	1,13	0,01	0,98	0,36	0,89	0,02	1,08	0,36	1,16	0,03	0,97	0,36
376379		1,07	0,32	1,05	0,70	1,23	0,24	0,85	0,00	0,88	0,01	1,02	0,60	0,74	0,00	0,84	0,00	0,83	0,01	0,74	0,00	0,71	0,00	0,77	0,10	0,81	0,16	0,77	0,15	0,69	0,07
377849	SYHC,VATB2,PDIA3,RUVB2	0,81	0,00	0,84	0,00	0,69	0,00	0,86	0,00	0,77	0,00	0,65	0,00	0,74	0,00	0,88	0,00	0,61	0,00	1,08	0,02	1,05	0,10	0,94	0,04	1,22	0,00	0,96	0,07	1,03	0,31
379418	MYG1,SHLB1	0,67	0,00	0,66	0,00	0,41	0,00	0,74	0,00	0,71	0,00	0,56	0,00	0,74	0,00	0,66	0,00	0,63	0,00	1,03	0,71	1,01	0,95	1,00	0,97	0,91	0,04	1,28	0,02	1,42	0,00
380472	DCTN1	2,49	0,00	3,06	0,00	4,41	0,00	3,01	0,00	3,81	0,00	6,92	0,00	4,13	0,00	4,96	0,00	6,78	0,00	0,73	0,00	0,80	0,00	0,75	0,02	0,78	0,03	0,95	0,46	0,74	0,01
381843		1,63	0,00	1,99	0,00	2,50	0,00	1,39	0,00	1,74	0,00	2,56	0,00	1,19	0,02	1,47	0,00	1,54	0,00	0,74	0,00	0,80	0,00	0,76	0,00	0,81	0,00	0,89	0,09	0,67	0,00
384077	PLST,AMPM2,CPNE1,COR1B	0,79	0,00	0,83	0,00	0,70	0,00	0,86	0,00	0,90	0,00	0,95	0,22	0,65	0,00	0,87	0,00	0,82	0,00	0,96	0,03	0,87	0,00	0,94	0,13	1,11	0,01	1,19	0,01	1,23	0,01
386066		0,89	0,00	0,95	0,48	0,98	0,84	0,92	0,02	0,86	0,00	0,76	0,00	0,66	0,00	0,82	0,00	0,65	0,00	0,87	0,00	0,74	0,00	0,76	0,02	0,86	0,10	0,65	0,01	0,66	0,01
387435	FKB10	0,95	0,18	0,90	0,10	0,65	0,00	0,96	0,08	0,72	0,00	0,69	0,00	0,53	0,01	0,74	0,11	0,47	0,01	0,97	0,19	0,84	0,00	0,77	0,01	1,23	0,06	1,02	0,60	1,10	0,07

388879	TBB5	0,92	0,21	0,90	0,13	0,94	0,23	0,94	0,11	0,78	0,00	0,69	0,00	0,65	0,00	0,69	0,00	0,55	0,00	1,09	0,20	0,97	0,68	0,93	0,32	1,06	0,37	0,78	0,00	0,81	0,00
389942		0,98	0,80	0,99	0,88	0,98	0,89	0,98	0,86	0,81	0,13	0,76	0,02	0,77	0,02	1,10	0,30	0,90	0,30	0,97	0,66	0,77	0,01	0,79	0,07	1,09	0,22	0,74	0,12	0,90	0,53
393893		0,39	0,00	0,43	0,00	0,24	0,00	0,59	0,00	0,42	0,00	0,28	0,00	0,44	0,00	0,58	0,00	0,29	0,00	1,53	0,00	1,48	0,00	0,98	0,88	1,77	0,00	1,20	0,54	1,60	0,02
396816		1,18	0,02	1,26	0,03	1,24	0,08	0,99	0,86	0,99	0,67	1,08	0,12	0,85	0,02	1,10	0,13	0,88	0,04	0,88	0,05	0,82	0,00	0,82	0,03	0,99	0,86	0,91	0,36	0,80	0,07
399832	PAK2,PDIA3	0,89	0,05	1,03	0,60	1,01	0,74	1,01	0,51	0,89	0,00	0,99	0,73	0,93	0,10	1,04	0,29	0,76	0,00	1,09	0,12	1,06	0,34	0,82	0,02	1,02	0,74	0,93	0,13	0,76	0,00
401246		0,87	0,57	1,38	0,05	1,50	0,05	1,44	0,00	1,12	0,20	1,50	0,02	0,90	0,10	1,03	0,56	0,84	0,01	1,27	0,26	1,17	0,46	0,63	0,00	0,85	0,06	0,77	0,10	0,63	0,01
402687	TBA1B	0,38	0,00	0,64	0,00	0,51	0,00	0,71	0,00	0,52	0,00	0,37	0,00	0,60	0,00	0,66	0,00	0,42	0,00	1,83	0,00	1,72	0,00	0,79	0,01	1,12	0,08	0,71	0,03	0,91	0,38
404170		N,A,	N,A,	1.000,00	N,A,	1.000,00	N,A,	0,81	0,31	9,21	0,00	0,00	0,01	0,39	0,02	0,64	0,15	0,73	0,34	1.000,00	N,A,	1.000,00	N,A,	7,89	0,01	0,85	0,79	0,00	0,01	10,70	0,08
405373	TCPE,FKBP4,CPNE1	1,03	0,35	1,10	0,02	0,79	0,00	0,96	0,09	0,94	0,04	0,88	0,00	0,83	0,00	1,12	0,00	0,81	0,00	0,86	0,00	0,86	0,00	0,79	0,00	1,09	0,01	1,03	0,54	1,09	0,07
407629	SYK	0,78	0,08	1,00	0,98	1,29	0,29	1,26	0,49	2,91	0,04	4,41	0,04	1,56	0,02	1,18	0,05	2,07	0,02	1,03	0,86	1,92	0,01	1,84	0,10	1,12	0,52	2,18	0,18	1,54	0,18
408351	SHLB2,AL9A1	0,83	0,01	1,04	0,06	0,64	0,00	0,87	0,02	0,81	0,01	0,60	0,00	0,59	0,00	0,79	0,00	0,58	0,00	0,94	0,31	0,78	0,01	0,70	0,00	0,84	0,00	0,84	0,02	0,99	0,74
409787		0,68	0,07	0,85	0,01	0,55	0,00	0,84	0,01	0,77	0,02	0,56	0,00	0,61	0,00	0,85	0,00	0,51	0,00	0,98	0,94	0,77	0,29	0,73	0,01	0,86	0,01	0,82	0,00	0,79	0,15
411894		0,58	0,03	0,79	0,03	0,57	0,01	1,11	0,12	0,63	0,00	0,85	0,03	0,92	0,07	1,14	0,01	0,79	0,00	1,56	0,05	1,61	0,04	0,66	0,00	1,48	0,00	1,23	0,03	1,42	0,00
412768		0,57	0,00	0,73	0,00	0,46	0,00	0,85	0,00	0,69	0,00	0,68	0,00	0,62	0,00	0,74	0,00	0,52	0,00	1,31	0,02	1,29	0,02	0,83	0,00	1,22	0,00	1,30	0,00	1,37	0,00
414193	THOC3	0,79	0,09	0,79	0,00	0,58	0,00	0,87	0,01	0,79	0,00	0,71	0,00	0,72	0,00	0,76	0,00	0,62	0,00	1,07	0,63	1,04	0,76	0,97	0,19	1,09	0,01	1,18	0,00	1,22	0,01
417583	LDHB,THOC3	0,79	0,05	0,99	0,93	1,16	0,34	0,93	0,44	0,88	0,21	0,96	0,61	0,88	0,14	1,29	0,07	0,87	0,14	1,11	0,37	1,08	0,54	0,83	0,10	1,25	0,13	0,77	0,14	0,72	0,10
418384	SCMC1,TADBP,PPME1,ACTG	0,80	0,00	1,00	1,00	0,88	0,02	0,74	0,02	0,67	0,00	0,50	0,00	0,58	0,00	0,52	0,00	0,54	0,00	1,13	0,23	0,96	0,16	0,82	0,03	0,68	0,00	0,70	0,00	0,81	0,00
421481		1,18	0,14	1,02	0,78	1,07	0,41	0,95	0,29	0,71	0,00	0,72	0,00	0,90	0,01	0,99	0,66	0,63	0,00	1,11	0,26	1,18	0,09	0,97	0,60	1,51	0,00	0,93	0,24	0,92	0,23
422947	GSTO1,PSB7	0,77	0,00	0,88	0,00	0,63	0,00	0,78	0,00	0,88	0,00	0,61	0,00	0,77	0,00	0,75	0,00	0,70	0,00	0,80	0,00	0,75	0,00	0,79	0,00	0,63	0,00	0,77	0,00	0,83	0,00
424315		0,92	0,06	0,99	0,74	0,86	0,06	0,90	0,01	0,80	0,00	0,81	0,00	0,79	0,00	0,84	0,00	0,82	0,00	0,95	0,14	0,82	0,00	0,79	0,00	0,82	0,00	0,92	0,27	0,92	0,27
425876		1,00	0,99	1,13	0,24	0,77	0,18	1,16	0,04	1,09	0,18	0,95	0,34	0,90	0,03	1,20	0,00	1,00	0,89	0,97	0,84	0,71	0,10	0,80	0,06	0,84	0,10	1,03	0,88	1,02	0,90
427625		0,72	0,02	1,13	0,50	1,00	0,99	0,85	0,34	0,86	0,34	0,63	0,04	1,08	0,11	1,03	0,15	0,81	0,00	1,09	0,50	1,01	0,92	0,70	0,10	0,62	0,05	0,58	0,15	0,55	0,12
429069		0,92	0,65	0,91	0,60	1,01	0,99	0,82	0,29	0,97	0,85	0,84	0,35	0,77	0,15	1,04	0,81	0,81	0,22	0,71	0,00	0,65	0,00	0,85	0,13	0,90	0,24	0,67	0,07	0,63	0,05
430224		0,70	0,00	0,68	0,00	0,46	0,00	0,74	0,00	0,75	0,00	0,57	0,00	0,67	0,00	0,72	0,00	0,58	0,00	0,91	0,14	0,82	0,04	0,94	0,22	0,89	0,07	1,07	0,28	1,08	0,12
431675		1,90	0,00	2,01	0,00	4,10	0,00	1,43	0,00	1,31	0,00	1,64	0,00	1,32	0,00	1,41	0,00	1,46	0,00	0,64	0,00	0,60	0,00	0,55	0,01	0,60	0,01	0,34	0,00	0,31	0,00
433407		1,07	0,05	0,95	0,19	0,93	0,03	1,04	0,20	0,93	0,17	0,83	0,00	1,04	0,03	1,23	0,00	0,84	0,00	0,91	0,02	0,94	0,02	0,92	0,20	1,25	0,00	0,84	0,00	0,87	0,00
434362		0,67	0,04	0,89	0,24	0,77	0,08	0,86	0,00	0,95	0,08	0,78	0,00	0,82	0,06	0,95	0,66	0,89	0,18	1,21	0,31	1,19	0,36	1,00	1,00	1,04	0,78	0,95	0,74	1,13	0,39
435879	DNJC9,PSME3	0,92	0,20	1,03	0,16	0,86	0,00	0,97	0,45	0,98	0,34	0,81	0,00	0,98	0,28	1,03	0,16	0,91	0,00	0,92	0,28	0,84	0,04	0,83	0,00	0,80	0,00	0,82	0,00	0,84	0,00
437747		0,97	0,79	1,15	0,04	1,15	0,04	1,14	0,01	1,07	0,04	1,08	0,10	1,37	0,00	1,03	0,14	1,24	0,00	1,16	0,21	1,22	0,10	0,92	0,16	0,78	0,00	0,94	0,34	0,94	0,25
438739		1,09	0,43	1,48	0,01	0,76	0,11	1,16	0,37	1,03	0,82	1,03	0,86	0,54	0,00	0,68	0,00	0,59	0,00	0,86	0,11	0,71	0,01	0,57	0,00	0,67	0,00	1,10	0,41	1,12	0,21
440482		2,20	0,39	0,90	0,63	0,56	0,02	2,20	0,00	1,69	0,05	2,13	0,10	1,92	0,00	1,68	0,00	1,76	0,06	1,04	0,95	1,05	0,94	1,97	0,05	2,26	0,00	3,97	0,07	3,78	0,03
443236	6PGL,PSME1	0,99	0,76	1,11	0,00	1,08	0,00	1,02	0,46	0,92	0,01	0,92	0,01	0,89	0,00	1,01	0,53	0,82	0,00	1,00	0,92	0,95	0,34	0,79	0,00	0,96	0,06	0,81	0,00	0,80	0,00
444662		2,13	0,00	2,13	0,00	3,44	0,00	1,59	0,00	1,20	0,00	1,39	0,00	1,76	0,00	1,40	0,00	2,00	0,01	0,71	0,03	0,62	0,01	0,53	0,00	0,50	0,00	0,38	0,00	0,44	0,00
445851	DNJA2,APMAP,SNX6	0,86	0,00	0,93	0,15	0,74	0,01	0,87	0,00	0,98	0,31	0,81	0,00	0,77	0,00	0,95	0,38	0,76	0,01	0,88	0,00	0,82	0,00	0,91	0,09	0,92	0,19	0,95	0,55	0,93	0,48
447263	HNRH1,CNDP2	0,69	0,04	0,79	0,06	0,65	0,02	0,64	0,00	0,60	0,00	0,51	0,00	0,84	0,00	0,84	0,01	0,68	0,00	1,05	0,69	1,23	0,12	0,87	0,03	1,08	0,21	0,88	0,10	1,05	0,26
449534		0,80	0,00	0,71	0,00	0,67	0,00	0,88	0,03	0,72	0,00	0,62	0,00	0,80	0,01	0,75	0,00	0,69	0,00	1,11	0,08	1,07	0,32	1,02	0,73	1,12	0,15	0,94	0,22	1,10	0,15
450085	PEPD,PDIA3,TBA1B	0,99	0,83	1,08	0,11	1,03	0,63	1,10	0,01	0,97	0,30	1,09	0,02	0,94	0,00	1,29	0,00	0,81	0,00	1,03	0,63	1,08	0,15	0,83	0,00	1,37	0,00	0,97	0,55	0,90	0,05
451640	PDIA3	0,88	0,30	1,29	0,01	1,50	0,00	1,09	0,02	0,92	0,05	1,00	0,98	0,80	0,00	0,87	0,05	0,73	0,00	1,28	0,06	1,19	0,17	0,74	0,01	0,88	0,13	0,69	0,01	0,63	0,00
452477	PPCE	1,16	0,08	1,61	0,01	1,55	0,01	1,25	0,02	1,86	0,00	2,65	0,00	1,34	0,00	2,54	0,00	1,87	0,00	0,84	0,04	1,02	0,79	0,90	0,36	1,39	0,01	1,34	0,02	1,07	0,47
457100		0,78	0,00	0,75	0,00	0,67	0,00	0,72	0,00	0,68	0,00	0,63	0,00	0,62	0,00	0,75	0,00	0,60	0,00	0,91	0,06	0,87	0,01	0,89	0,02	1,09	0,06	0,93	0,17	0,98	0,81
458579	PUR4	0,68	0,00	0,64	0,00	0,48	0,00	0,68	0,00	0,69	0,00	0,58	0,00	0,68	0,00	0,77	0,00	0,62	0,00	0,91	0,05	0,86	0,01	0,98	0,51	1,04	0,27	1,09	0,15	1,12	0,12
460138	GANAB	0,76	0,00	0,51	0,00	0,24	0,00	0,74	0,00	0,58	0,00	0,48	0,00	0,59	0,00	0,76	0,00	0,47	0,00	0,99	0,91	0,90	0,09	1,15	0,32	1,73	0,00	1,99	0,00	2,24	0,00
461679		0,74	0,02	0,66	0,01	0,73	0,04	0,80	0,00	0,54	0,00	0,54	0,00	0,56	0,00	0,74	0,01	0,41	0,00	1,17	0,00	1,08	0,09	0,89	0,28	1,60	0,01	0,80	0,01	0,80	0,03
463195		0,70	0,00	0,24	0,00	0,13	0,00	0,65	0,00	0,62	0,07	0,42	0,00	0,64	0,00	0,71	0,00	0,50	0,00	1,20	0,02	1,07	0,16	3,32	0,07	3,42	0,01	4,38	0,01	4,72	0,00
464690		0,81	0,01	0,41	0,01	0,25	0,00	0,78	0,00	0,72	0,01	0,58	0,00	0,61	0,00	0,72	0,00	0,56	0,00	0,95	0,17	0,91	0,03	1,74	0,13	2,13	0,03	2,25	0,07	2,70	0,03
466049		0,54	0,00	0,25	0,00	0,00	0,00	0,59	0,00	0,62	0,10	0,38	0,00	0,40	0,00	0,58	0,00	0,34	0,00	1,15	0,07	0,90	0,24	2,61	0,13	2,84	0,00	1.000,00	0,00	1.000,00	0,00
467907	AGM1	0,87	0,07	0,59	0,09	0,68	0,33	1,03	0,65	1,35	0,00	1,07	0,18	0,67	0,00	0,53	0,00	0,68	0,00	0,85	0,07	0,85	0,08	1,63	0,12	0,98	0,96	1,12	0,80	1,10	0,82
469498	PRS4,SYWC	0,93	0,19	1,01	0,90	1,08	0,25	0,92	0,05	0,91	0,03	0,91	0,02	0,79	0,01	1,00	0,99	0,86	0,02	0,96	0,35	0,90	0,06	0,89	0,13	1,04	0,58	0,83	0,00	0,84	0,01
471051	HNRH1,TIF1B	0,69	0,00	0,32	0,00	0,20	0,00	0,79	0,00	0,75	0,00	0,64	0,00	0,78	0,00	0,81	0,00	0,66	0,00	1,01	0,75	1,02	0,60	2,04	0,04	2,29	0,02	2,81	0,01	2,98	0,01
473357		1,02	0,62	0,57	0,10	0,15	0,00	1,10	0,03	1,19	0,00	1,30	0,00	1,08	0,14	1,29	0,00	1,33	0,00	0,92	0,10	0,90	0,07	1,78	0,09	1,92	0,05	7,44	0,00	7,62	0,00
474852		1,23	0,02	0,99	0,86	0,93	0,34	1,02	0,62	1,58	0,00	1,55	0,00	0,76	0,00	0,75	0,00	0,79	0,00	0,66	0,00	0,78	0,00	1,26	0,00	0,95	0,43	1,31	0,01	1,07	0,30
479360	CAP2	0,99	0,74	0,39	0,05	0,21	0,01	0,93	0,01	0,90	0,01	0,83	0,00	0,91	0,01	1,01	0,57	0,87	0,00	1,00	0,94	0,98	0,54	2,44	0,07	2,76	0,04	4,15	0,01	4,40	0,01
479881		1,00	0,97	1,22	0,63	2,61	0,34	0,95	0,06	1,10	0,01	1,10	0,01	0,97	0,57	1,11	0,07	1,06	0,34	0,87	0,05	0,87	0,07	0,83	0,65	0,83	0,64	0,39	0,34	0,37	0,33
482337		0,94	0,10	1,36	0,55	3,11	0,01	0,93	0,01	0,90	0,01	0,90	0,01	0,78	0,00	0,91	0,05	0,83	0,01	0,91	0,00	0,85	0,00	0,61	0,38	0,69	0,48	0,27	0,01	0,27	0,01
483852		0,79	0,00	0,86	0,03	0,85	0,02	0,88	0,01	1,12	0,02	1,45	0,00	0,89	0,02	1,00	0,97	1,06	0,03	0,97	0,43	1,10	0,11	1,12	0,04	1,13	0,05	1,47	0,00	1,21	0,00
485355	VINC	0,84	0,01	0,92	0,17	0,80	0,01	0,87	0,00	0,88	0,00	0,99	0,73	0,78	0,00	0,93	0,09	0,93	0,09	0,97	0,35	0,94	0,17	0,90	0,06	1,03	0,60	1,17	0,00	1,19	0,00
485827		0,81	0,00	0,88	0,02	0,83	0,03	0,81	0,00	0,95	0,05	0,88	0,00	0,81	0,00	0,94	0,24	0,96	0,43	0,90	0,03	0,87	0,02	0,97	0,53	0,95	0,39	0,95	0,52	1,02	0,79
487316		0,95	0,12	0,96	0,48	0,95	0,61	0,95	0,14	0,97	0,34	0,93	0,05	0,79	0,01	0,91	0,11	0,81	0,01	0,89	0,01	0,84	0,01	0,89	0,13	0,96	0,53	0,87	0,24	0,86	0,25
488759	CALD1	0,55	0,00	0,21	0,01	5,76	0,25	0,52	0,00	0,47	0,00	0,42	0,00	0,63	0,00	0,73	0,00	0,56	0,00	0,99	0,84	1,05	0,22	2,41	0,15	3,23	0,04	0,08	0,21	0,09	0,21
490428	LMNA,AGM1	1,01	0,66	0,40	0,06	0,10	0,00	1,05	0,63	0,98	0,82	0,85	0,09	0,96	0,67	1,13	0,21	0,99	0,91	1,02	0,73	0,84	0,01	2,46	0,07	2,55	0,05	8,80	0,00	9,21	0,00
492895		0,78	0,00	1,07	0,87	1,91	0,35	0,73	0,00	0,84	0,00	0,72	0,00	0,67	0,00	0,86	0,01	0,77	0,00	0,84	0,00	0,92	0,01	0,70	0,49	0,86	0,73	0,34	0,21	0,43	0,27
494430		1,87	0,00	2,17	0,00	2,56	0,00	1,62	0,00	2,72	0,00	4,48	0,00	2,07	0,00	2,73	0,00	2,54	0,00	0,75	0,00	0,86	0,06	1,09	0,28	0,98	0,78	1,51	0,00	0,77	0,01
495366	CAPG	0,81	0,00	0,67	0,00	0,51	0,00	0,88	0,00	0,71	0,00	0,70	0,00	0,67	0,00	0,98	0,72	0,59	0,00	1,09	0,01	0,97	0,54	1,05	0,32	1,70	0,00	1,37	0,00	1,35	0,00
496907	CAPG	0,68	0,00	0,35	0,00	0,41	0,00	0,74	0,00	0,56	0,00	0,55	0,00	0,58	0,00	0,66	0,00	0,41	0,00	0,98	0,63	0,95	0,30	1,44	0,02	2,09	0,00	1,22	0,10	1,11	0,10
502104		1,58	0,00	1,07	0,55	1,02	0,88	1,14	0,01	0,93	0,05	0,73	0,00	1,19	0,01	0,88	0,13	1,10	0,27	0,43	0,00	0,30	0,00	0,51	0,00	0,33	0,00	0,43	0,00	0,43	0,00
503644		0,93	0,10	0,67	0,22	0,19	0,00	0,92	0,03	0,86	0,00	0,82	0,00	0,77	0,00	0,84	0,00	0,75	0,00	0,96	0,25	0,91	0,03	1,23	0,54	1,38	0,33	4,13	0,00	4,26	0,00
504850		0,93	0,13	0,89	0,11	0,91	0,51	0,89	0,01	0,82	0,00	0,70	0,00	0,87	0,01	0,95	0,04	0,72	0,00	1,05	0,22	0,95	0,32	1,01	0,92	1,09	0,18	0,84	0,31	0,80	0,23
506742	SFRS3	0,68	0,01	0,76	0,05	0,72	0,09	0,85	0,01	0,69	0,00	0,56	0,00	0,72	0,00	0,82	0,01	0,54	0,00	1,24	0,00	1,05	0,45	0,90	0,29	1,09	0,31	0,78	0,16	0,75	0,20
508072	SODC	0,57	0,00	0,68	0,09	0,47	0,13	0,77	0,00	0,86	0,00	0,57	0,00	0,65	0,00	0,82	0,00	0,49	0,00	1,24	0,01	1,11	0,16	1,17	0,45	1,19	0,42	1,13	0,84	1,03	0,96
509563		0,64	0,14	0,17	0,01	0,00	0,01	1,09	0,56	0,56	0,01	0,43	0,00	1,23	0,29	0,77	0,03	0,95	0,64	0,82	0,21	0,68	0,07	1,64	0,43	1,65	0,43	69,37	0,00	111,59	0,00
511439		0,88	0,27	0,96	0,87	0,86	0,62	0,85	0,02	0,79	0,01	0,62	0,00	0,89	0,06	0,69	0,00	0,75	0,00	0,84	0,16	0,73	0,03	0,71	0,28	0,52	0,09	0,62	0,28	0,64	0,29
520493		1,41	0,00	0,66	0,45	0,03	0,00	1,43	0,00	2,14	0,00	2,60	0,00	1,99	0,00	1,98	0,00	2,34	0,00	0,85	0,01	1,01	0,83	2,69	0,03	2,14	0,11	78,47	0,00	60,74	0,00
523641		1,06	0,63	0,69	0,21	0,41	0,03	0,97	0,74	0,87	0,18	1,17	0,23	0,85	0,15	1,10	0,15	0,95	0,53	0,87	0,19	0,78	0,08	1,19	0,56	1,54	0,12	2,68	0,02	2,24	0,04
525068		1,12	0,09	0,69	0,34	0,51	0,19	1,00	0,92	0,80	0,00	0,85	0,00	0,79	0,00	0,86	0,00	0,67	0,00	0,93	0,18	0,91	0,11	1,20	0,66	1,59	0,22	1,74	0,30	1,69	0,32
526716	RFC2	1,07	0,12	0,30	0,04	2,02	0,57	0,98	0,54	0,88	0,00	0,83	0,00	0,97	0,46	0,98	0,50	0,92	0,06	0,82	0,00	0,79	0,00	2,60	0,13	2,82	0,09	0,36	0,48	0,40	0,50
528317	EI2BL,ROAA,CAPG	0,80	0,14	0,64	0,00	0,47	0,00	0,86	0,00	0,70	0,00	0,64	0,00	0,69	0,00	0,81	0,00	0,51	0,00	1,25	0,11	1,19	0,20	1,27	0,00	1,74	0,00	1,60	0,01	1,51	0,01
529901		0,46	0,01	0,35	0,00	0,15	0,00	0,53	0,04	0,65	0,04	0,39	0,00	0,98	0,79	0,83	0,00	0,81	0,02	1,43	0,35	1,93	0,03	2,31	0,00	2,15	0,00	3,21	0,00	4,86	0,00
531704	TSNAX	0,86	0,00	0,39	0,06	0,31	0,02	0,86	0,01	0,78	0,00	0,68	0,00	0,94	0,18	0,84	0,00	0,70	0,00	1,05	0,15	1,06	0,24	2,06	0,16	2,08	0,15	2,34	0,08	2,24	0,10
534785	PP1A	0,88	0,00	0,85	0,00	0,68	0,00	0,90	0,00	0,86	0,00	0,77	0,00	0,80	0,00	0,88	0,00	0,77	0,00	0,94	0,01	0,89	0,00	0,94	0,10	1,02	0,63	1,04	0,08	1,10	0,01
535941		0,98	0,78	1,22	0,53	2,01	0,21	0,91	0,06	1,09	0,16	1,30	0,03	0,89	0,01	0,93	0,04	1,06	0,24	1,05	0,38	1,13	0,05	1,02	0,95	0,94	0,84	0,74	0,49	0,66	0,36
537545	LMNB1,TKT	1,28	0,03	1,18	0,06	0,82	0,09	0,80	0,00	0,75	0,00	0,69	0,00	0,54	0,00	0,67	0,00	0,37	0,00	0,75	0,00	0,75	0,00	0,76	0,00	1,00	0,96	1,00	0,92	0,79	0,00
540509	MVP	0,68	0,00	0,70	0,00	0,51	0,00	0,77	0,00	0,68	0,00	0,64	0,00	0,73	0,00	0,87	0,00	0,63	0,00	1,19	0,00	1,07	0,02	1,01	0,81	1,22	0,00	1,30	0,00	1,21	0,01
543455		1,11	0,01	1,34	0,00	1,69	0,00	0,95	0,00	0,76	0,00	0,80	0,00	0,84	0,00	0,93	0,02	0,64	0,00	1,03	0,26	0,94	0,10	0,68	0,00	0,86	0,02	0,57	0,00	0,47	0,00
543746		0,79	0,00	0,68	0,00	0,60	0,00	0,87	0,01	0,77	0,00	0,72	0,00	0,76	0,00	0,86	0,00	0,73	0,00	1,06	0,25	0,86	0,01	1,10	0,11	1,14	0,00	1,16	0,04	1,09	0,15
545274		0,60	0,00	0,49	0,00	0,16	0,00	0,80	0,00	0,66	0,00	0,55	0,00	0,44	0,00	0,69	0,00	0,45	0,00	1,29	0,01	0,86	0,15	1,32	0,00	1,69	0,00	3,30	0,00	3,28	0,00
546917		1,10	0,10	1,37	0,00	1,58	0,00	0,93	0,07	0,97	0,55	1,05	0,37	0,67	0,00	0,92	0,25	0,69	0,00	0,92	0,12	0,87	0,04	0,77	0,00	0,95	0,22	0,72	0,00	0,62	0,00
549951	UBA1	1,06	0,14	1,29	0,00	1,24	0,00	0,85	0,01	0,94	0,18	1,06	0,08	0,68	0,00	0,95	0,08	0,75	0,00	1,00	0,92	0,99	0,76	0,91	0,02	1,15	0,00	1,06	0,08	0,95	0,22
550244		0,67	0,00	0,70	0,00	0,55	0,00	0,79	0,00	0,68	0,00	0,63	0,00	0,74	0,00	0,90	0,00	0,69	0,00	1,26	0,00	1,11	0,00	1,05	0,20	1,28	0,00	1,23	0,00	1,25	0,00
551845		1,22	0,00	1,43	0,00	1,49	0,00	1,06	0,33	1,20	0,01	1,47	0,00	1,07	0,32	1,34	0,00	1,27	0,01	0,87	0,01	0,84	0,00	0,84	0,01	0,90	0,04	0,99	0,93	0,82	0,04
554833	UBE1	0,51	0,00	0,48	0,00	0,33	0,00	0,70	0,00	0,56	0,00	0,48	0,00	0,67	0,00	0,78	0,00	0,57	0,00	1,35	0,00	1,12	0,07	1,13	0,01	1,37	0,00	1,43	0,00	1,48	0,00
556498	KBP	0,97	0,63	1,61	0,02	1,19	0,05	1,02	0,59	1,04	0,69	1,11	0,12	0,39	0,00	1,01	0,55	0,43	0,00	0,79	0,00	0,75	0,00	0,48	0,00	1,15	0,21	0,70	0,00	0,66	0,00
558160		0,60	0,05	0,68	0,05	0,41	0,01	0,82	0,30	0,98	0,93	0,96	0,81	0,58	0,00	0,56	0,00	0,58	0,00	0,95	0,81	1,05	0,80	1,01	0,96	0,88	0,09	1,63	0,00	1,51	0,02
559703		0,83	0,01	1,01	0,77	1,18	0,03	0,83	0,01	0,90	0,04	0,98	0,57	0,90	0,03	1,01	0,75	0,92	0,20	1,01	0,90	1,07	0,19	0,89	0,04	0,98	0,68	0,84	0,01	0,77	0,02
562964		0,89	0,22	0,95	0,50	1,16	0,12	0,93	0,36	1,06	0,37	1,30	0,00	0,76	0,00	0,95	0,11	0,84	0,00	0,86	0,15	0,90	0,22	0,91	0,21	1,05	0,34	0,91	0,19	0,77	0,01
563145		0,79	0,00	0,62	0,04	1,02	0,95	0,94	0,57	1,97	0,23	1,20	0,75	0,19	0,23	0,22	0,25	0,24	0,25	0,96	0,56	0,56	0,02	2,56	0,16	0,79	0,71	0,94	0,94	0,52	0,27
566208		0,93	0,17	0,79	0,00	0,51	0,00	0,91	0,03	0,80	0,00	0,70	0,00	0,70	0,00	0,82	0,08	0,66	0,01	0,97	0,61	0,76	0,00	1,00	0,98	1,05	0,42	1,37	0,01	1,31	0,05
568037		0,67	0,00	0,64	0,00	0,64	0,00	0,73	0,02	0,62	0,01	0,60	0,01	0,98	0,78	1,15	0,08	0,87	0,14	1,17	0,00	1,07	0,16	1,05	0,69	1,33	0,00	1,00	0,99	1,00	0,96
569671		0,71	0,00	0,74	0,00	0,76	0,01	0,77	0,12	0,72	0,06	0,96	0,85	1,10	0,14	1,22	0,01	0,93	0,34	1,02	0,86	1,11	0,03	0,92	0,37	1,20	0,01	1,20	0,52	0,89	0,32
574393		0,66	0,00	0,66	0,00	0,57	0,00	0,70	0,00	0,65	0,00	0,58	0,00	0,59	0,00	0,73	0,00	0,54	0,00	1,00	0,94	0,95	0,38	0,93	0,21	1,17	0,03	0,95	0,72	1,00	0,97
574397		0,70	0,00	0,76	0,13	0,76	0,30	0,74	0,07	0,67	0,02	1,02	0,86	0,60	0,00	0,78	0,03	0,67	0,01	0,94	0,57	0,94	0,20	0,78	0,29	1,13	0,51	1,19	0,52	0,97	0,93
574883		1,10	0,02	0,99	0,74	0,90	0,07	0,79	0,02	0,97	0,59	0,70	0,00	0,66	0,00	0,78	0,02	0,85	0,11	0,58	0,00	0,51	0,00	0,79	0,00	0,67	0,00	0,62	0,00	0,80	0,04

576672		0,58	0,01	0,54	0,00	0,35	0,00	0,58	0,00	0,71	0,00	0,50	0,00	0,99	0,90	1,32	0,01	1,00	0,98
580959		0,73	0,00	0,75	0,05	0,77	0,20	0,77	0,05	0,71	0,04	0,98	0,83	0,61	0,00	0,80	0,01	0,68	0,00
582682		0,77	0,03	0,56	0,00	0,31	0,00	0,60	0,00	0,73	0,00	0,51	0,00	0,68	0,00	0,74	0,00	0,86	0,00
584346	PRDX3,HSPB1	1,00	0,97	0,80	0,45	0,62	0,34	0,88	0,00	0,85	0,00	0,72	0,00	0,83	0,00	0,93	0,01	0,71	0,00
586647	DC1L2,TCPB	0,81	0,00	0,42	0,02	0,28	0,00	0,82	0,02	0,79	0,00	0,68	0,00	0,88	0,01	0,80	0,00	0,76	0,00
589226		1,13	0,08	1,31	0,25	1,75	0,20	1,21	0,01	1,43	0,00	1,72	0,00	1,08	0,14	1,17	0,01	1,32	0,00
589630		0,76	0,00	0,37	0,01	0,00	N,A,	0,69	0,00	0,56	0,00	0,59	0,00	0,68	0,00	0,76	0,00	0,54	0,00
594301		1,11	0,03	1,28	0,01	1,75	0,00	1,07	0,05	1,08	0,32	1,00	0,87	1,06	0,49	1,28	0,03	1,12	0,15
596190	HS90A	1,61	0,00	2,09	0,00	1,89	0,00	1,46	0,00	0,99	0,93	1,27	0,01	0,47	0,01	1,04	0,83	0,61	0,03
1763758		0,05	0,00	0,44	0,01	0,33	0,01	0,38	0,00	9,66	0,17	0,05	0,00	1,14	0,87	0,32	0,01	0,17	0,00
1763762		0,27	0,00	0,16	0,00	0,00	0,00	0,26	0,43	1,15	0,89	0,34	0,50	0,70	0,17	0,97	0,87	0,67	0,23
1763803		0,15	0,00	0,32	0,00	0,04	0,00	0,20	0,04	0,61	0,26	0,04	0,02	0,04	0,00	0,59	0,00	0,11	0,00
1764738		0,26	0,04	0,33	0,12	0,00	0,02	0,31	0,03	1,31	0,55	1,49	0,43	0,88	0,74	0,75	0,28	1,50	0,52
1764993		0,40	0,18	0,63	0,34	0,40	0,21	0,15	0,00	1,59	0,49	0,05	0,00	0,17	0,11	0,05	0,06	82,14	0,35
1765003		0,31	0,14	0,55	0,36	0,35	0,17	0,01	0,00	0,66	0,24	0,00	0,00	0,01	0,02	0,11	0,03	0,00	0,10
1780185		0,01	0,01	1,70	0,59	0,07	0,02	0,13	0,03	0,56	0,23	0,15	0,04	0,00	0,00	0,41	0,00	0,12	0,00
1785181		0,16	0,00	0,34	0,00	0,35	0,04	0,29	0,00	0,65	0,03	0,35	0,03	0,00	0,00	0,61	0,00	0,10	0,00
1785527		0,20	0,00	0,34	0,00	0,07	0,00	0,29	0,00	0,37	0,00	1,43	0,69	0,00	0,00	0,58	0,00	0,13	0,00
1787194		0,03	0,00	0,54	0,05	0,10	0,00	0,20	0,00	0,42	0,02	1,28	0,81	0,00	0,00	0,41	0,00	0,09	0,00
1788919		0,24	0,00	0,47	0,00	0,18	0,00	0,37	0,00	0,55	0,01	0,34	0,00	0,00	0,00	0,52	0,00	0,23	0,00
1792282		0,39	0,00	0,40	0,00	0,43	0,02	0,57	0,03	0,50	0,00	0,33	0,00	0,00	0,00	0,66	0,00	0,24	0,00
1793947		0,64	0,19	0,36	0,00	0,39	0,01	0,64	0,10	0,67	0,04	0,61	0,08	0,19	0,00	0,81	0,00	0,49	0,00
1797353		0,41	0,00	0,49	0,02	0,34	0,02	0,46	0,00	0,81	0,08	0,24	0,00	0,11	0,00	0,82	0,05	0,33	0,00
1799192		0,75	0,02	1,86	0,02	2,39	0,01	0,67	0,00	1,79	0,00	1,95	0,00	0,39	0,05	1,11	0,23	0,83	0,27
1800806		0,21	0,00	0,27	0,01	0,01	0,00	0,13	0,13	0,83	0,79	0,02	0,16	0,13	0,00	0,77	0,01	0,19	0,00
1802534		0,63	0,00	1,82	0,20	2,22	0,16	0,67	0,03	1,72	0,01	1,89	0,03	0,36	0,01	1,42	0,00	0,67	0,05
1804168		1,98	0,01	1,82	0,27	1,64	0,11	1,50	0,03	1,72	0,01	2,35	0,00	1,34	0,39	1,71	0,12	2,97	0,00
1809464		0,35	0,12	0,28	0,12	0,00	0,06	0,25	0,17	1,59	0,58	0,35	0,22	0,06	0,24	0,08	0,33	0,09	0,25
1811207		0,90	0,39	1,35	0,07	1,69	0,19	3,42	0,00	7,28	0,00	9,70	0,00	1,72	0,03	3,25	0,00	2,24	0,02
1814025		0,42	0,07	0,96	0,93	2,41	0,13	0,25	0,20	2,36	0,15	0,00	0,26	0,08	0,01	1,03	0,93	0,38	0,06
1814733		0,62	0,16	0,71	0,39	4,80	0,26	0,88	0,64	1,46	0,29	0,80	0,27	0,27	0,01	0,91	0,66	0,40	0,02
1814782		2,27	0,00	2,72	0,00	3,43	0,00	1,69	0,00	2,34	0,00	3,25	0,00	2,03	0,00	2,90	0,00	2,68	0,00
1816422		0,43	0,01	0,84	0,54	1,40	0,43	0,60	0,02	1,75	0,43	0,23	0,00	0,09	0,00	0,87	0,28	0,27	0,00
1819121		0,98	0,88	1,14	0,59	2,60	0,06	1,31	0,03	1,56	0,04	1,52	0,02	0,51	0,00	1,03	0,76	0,86	0,42
1819877		0,48	0,00	0,90	0,64	1,15	0,73	0,73	0,26	1,35	0,51	0,37	0,04	0,23	0,00	0,94	0,52	0,42	0,00
1821608		0,77	0,24	1,02	0,92	1,58	0,11	1,33	0,19	1,90	0,07	1,37	0,23	0,60	0,03	0,99	0,92	0,89	0,53
1825144	RCN2	0,82	0,01	1,01	0,86	0,84	0,37	0,93	0,24	0,90	0,16	0,76	0,01	0,98	0,74	1,05	0,57	0,76	0,01
1828633	ALDOA	1,95	0,00	2,75	0,00	3,24	0,00	1,54	0,00	2,45	0,00	3,23	0,00	1,83	0,00	2,32	0,00	2,38	0,00
1837103		1,37	0,04	1,77	0,01	2,02	0,01	1,05	0,75	1,68	0,02	1,78	0,00	2,38	0,15	2,60	0,02	8,41	0,19
1840030		1,56	0,01	1,76	0,01	1,92	0,02	1,33	0,03	1,97	0,00	2,14	0,01	2,91	0,01	2,96	0,00	8,81	0,01
1841741		2,18	0,01	1,58	0,10	1,52	0,32	1,48	0,01	2,63	0,00	2,90	0,00	5,38	0,04	3,69	0,00	8,46	0,06
1843456		0,43	0,23	0,32	0,16	0,27	0,20	1,28	0,17	2,12	0,03	1,79	0,02	60,61	0,14	4,85	0,00	10,88	0,12
1844173		1,76	0,00	1,94	0,00	2,20	0,00	1,60	0,00	1,83	0,00	2,69	0,00	1,44	0,00	2,06	0,00	2,29	0,00
1846930		1,62	0,09	2,92	0,01	2,14	0,06	1,36	0,01	0,51	0,01	0,85	0,40	1,06	0,91	1,76	0,22	1,23	0,67
1847793		0,57	0,01	0,71	0,07	0,51	0,01	0,49	0,00	0,44	0,00	0,20	0,00	0,30	0,00	0,38	0,00	0,29	0,00
1864524		0,95	0,90	1,72	0,20	1,54	0,33	0,86	0,00	0,83	0,00	0,80	0,01	0,51	0,08	0,86	0,63	0,43	0,05
1864541		0,63	0,00	0,71	0,00	0,42	0,00	0,36	0,00	0,59	0,06	0,15	0,00	0,30	0,14	0,30	0,14	0,20	0,10
1866181		0,57	0,00	0,40	0,00	0,35	0,00	0,11	0,02	0,24	0,01	0,04	0,00	0,16	0,01	0,41	0,02	0,00	0,00
1867746		1,34	0,00	1,54	0,07	1,09	0,55	1,41	0,04	2,62	0,00	3,07	0,00	1,74	0,02	2,40	0,00	1,67	0,16
1871260		0,57	0,13	0,55	0,21	0,40	0,06	0,49	0,08	1,12	0,65	1,39	0,08	1,18	0,79	1,72	0,30	0,63	0,58
1911469		1,30	0,01	1,27	0,22	1,19	0,16	1,22	0,06	1,25	0,06	1,21	0,10	1,24	0,03	1,88	0,00	1,71	0,01
1913428	ELOB	0,48	0,17	0,28	0,04	0,15	0,04	0,94	0,95	0,52	0,33	0,26	0,14	0,48	0,20	0,66	0,37	0,30	0,11
1915149		0,90	0,81	1,38	0,68	0,45	0,14	0,26	0,40	7,46	0,03	11,88	0,13	0,03	0,05	0,82	0,68	1,22	0,75
1931678		1,12	0,09	0,98	0,73	1,01	0,92	0,85	0,12	0,81	0,04	0,78	0,02	1,75	0,03	0,95	0,75	1,99	0,00
1933424		1,26	0,02	1,29	0,08	1,03	0,86	0,95	0,47	0,73	0,01	0,74	0,01	2,66	0,03	1,13	0,30	2,81	0,00
1935188		1,31	0,21	1,61	0,06	1,12	0,70	0,92	0,34	0,84	0,10	0,81	0,01	2,39	0,04	0,94	0,77	2,35	0,00
1936868	MRLC2	0,10	0,06	0,18	0,09	0,08	0,11	0,50	0,07	0,80	0,50	0,29	0,03	0,02	0,15	0,50	0,48	0,03	0,15
1953484	MLRN	0,57	0,07	0,64	0,14	0,49	0,09	0,67	0,00	0,79	0,00	0,46	0,00	0,84	0,01	0,80	0,00	0,63	0,00
1960958		1,11	0,39	1,91	0,37	2,06	0,35	1,03	0,72	1,13	0,25	0,86	0,19	0,77	0,55	1,52	0,15	0,88	0,66
1962730	LEG1	0,75	0,00	0,84	0,00	0,56	0,00	0,98	0,59	0,75	0,00	0,66	0,00	1,20	0,10	1,01	0,61	1,08	0,36
1964508		1,05	0,79	0,77	0,19	0,80	0,30	0,92	0,22	0,83	0,16	0,89	0,36	2,49	0,00	1,58	0,00	3,01	0,00
1978481		1,20	0,12	1,18	0,42	1,69	0,03	1,59	0,05	1,43	0,16	1,54	0,09	0,96	0,81	2,35	0,00	1,16	0,52
1980208		0,57	0,05	0,55	0,03	0,18	0,00	0,86	0,73	1,31	0,55	0,96	0,92	0,44	0,33	0,36	0,28	0,32	0,27
1980598		0,09	0,01	0,31	0,04	0,05	0,02	0,07	0,15	0,60	0,58	0,01	0,20	0,01	0,25	0,05	0,27	0,00	0,25
1982157		0,37	0,00	0,73	0,01	0,36	0,00	0,46	0,00	0,69	0,01	0,27	0,00	0,36	0,00	0,40	0,00	0,26	0,00
1983973		0,81	0,24	1,63	0,35	1,46	0,54	0,96	0,75	0,79	0,08	0,73	0,03	8,36	0,36	1,16	0,27	0,44	0,01
1985723		0,30	0,04	0,44	0,05	0,27	0,04	0,67	0,16	0,80	0,37	0,47	0,03	0,62	0,03	0,69	0,06	0,54	0,03
1987569		0,20	0,24	0,83	0,74	0,46	0,40	0,63	0,05	0,64	0,06	0,53	0,02	2,94	0,00	1,11	0,50	3,13	0,03
1989367		1,19	0,12	1,55	0,01	1,57	0,01	0,81	0,04	0,89	0,33	0,98	0,80	1,54	0,05	1,19	0,33	1,84	0,02
1991205		0,77	0,50	0,34	0,08	0,21	0,08	1,35	0,26	0,00	N,A,	0,00	N,A,	4,05	0,01	0,80	0,15	5,23	0,02
1993029		1,01	0,98	1,43	0,13	1,38	0,14	1,29	N,A,	0,00	N,A,	0,00	N,A,	2,36	N,A,	1,17	0,71	0,00	N,A,
1994828		0,94	0,83	0,56	0,12	0,47	0,12	1,33	0,17	0,96	N,A,	1,23	N,A,	6,92	0,00	1,33	0,00	8,80	0,01
1996662		1,00	0,99	1,05	0,92	0,87	0,59	N,A,	N,A,	N,A,	N,A,	N,A,	N,A,	0,18	N,A,	0,74	0,60	0,00	N,A,
1996674		0,90	0,86	0,41	0,36	0,75	0,68	N,A,	N,A,	N,A,	N,A,	N,A,	N,A,	0,21	N,A,	0,47	N,A,	0,00	N,A,
1998321		0,49	0,00	0,68	0,01	0,24	0,00	0,65	0,03	0,73	0,13	0,48	0,01	0,38	0,00	0,60	0,01	0,32	0,00
2000013	TCTP	1,37	0,06	1,17	0,30	1,01	0,95	0,75	0,34	1,87	0,05	1,16	0,64	0,77	0,30	1,28	0,37	1,45	0,27
2001699	CPNS1	1,05	0,61	1,09	0,18	0,80	0,03	1,03	0,76	1,00	0,99	0,84	0,15	0,80	0,00	0,88	0,02	0,76	0,00
2003479	AN32A	0,73	0,00	1,29	0,02	1,08	0,02	0,77	0,00	0,71	0,00	0,53	0,00	1,19	0,15	0,77	0,13	0,94	0,58
2005865		0,70	0,00	0,99	0,94	0,72	0,02	0,79	0,18	0,72	0,04	0,54	0,00	0,76	0,00	0,86	0,02	0,85	0,11
2006762	CALU	0,44	0,00	0,77	0,52	0,25	0,00	0,15	0,24	0,18	0,25	0,01	0,17	0,31	0,01	0,62	0,00	0,28	0,01
2008587		0,62	0,00	0,80	0,10	0,57	0,01	0,39	0,00	0,43	0,01	0,28	0,00	0,29	0,00	0,74	0,00	0,63	0,28
2010414	BCCIP,ACTG	0,74	0,01	0,81	0,06	0,69	0,05	0,58	0,05	0,51	0,02	0,66	0,08	0,35	0,00	0,73	0,00	0,40	0,00
2016883		0,94	0,58	0,97	0,77	0,82	0,20	0,97	0,87	0,63	0,05	0,94	0,73	0,34	0,00	0,77	0,04	0,46	0,00
2019749		0,94	0,38	0,99	0,84	0,91	0,15	0,99	0,85	0,79	0,00	0,89	0,09	0,79	0,00	0,82	0,00	0,78	0,00
2021577		0,79	0,00	1,12	0,38	1,00	0,99	0,86	0,00	0,75	0,00	0,66	0,00	0,70	0,00	0,82	0,00	0,63	0,00
2023391		0,80	0,00	0,99	0,90	0,72	0,00	0,88	0,00	0,77	0,00	0,69	0,00	0,69	0,00	0,80	0,00	0,61	0,00
2024619		0,14	0,02	0,47	0,13	0,16	0,04	0,36	0,01	0,24	0,01	0,02	0,00	0,43	0,00	0,62	0,00	0,18	0,00
2027812		0,02	0,03	0,11	0,04	0,03	0,06	0,01	0,20	0,10	0,23	0,00	0,19	0,71	0,53	0,55	0,00	0,13	0,00
2028269		0,48	0,00	0,44	0,00	0,19	0,00	0,67	0,09	0,63	0,07	0,39	0,01	0,56	0,00	0,71	0,00	0,35	0,00
2030105		0,45	0,00	0,71	0,03	0,47	0,00	0,80	0,24	0,48	0,01	0,25	0,00	0,65	0,00	0,80	0,00	0,49	0,00
2031934		0,64	0,06	0,86	0,44	0,59	0,02	0,82	0,51	0,52	0,05	0,31	0,01	0,54	0,00	0,68	0,01	0,37	0,00
2033833	ID1737021	1,18	0,24	0,82	0,31	2,51	0,01	2,34	0,00	2,11	0,01	2,22	0,00	1,28	0,47	4,04	0,01	0,75	0,52
2035512		0,58	0,12	0,86	0,56	0,83	0,53	1,98	0,00	1,04	0,86	1,54	0,04	0,29	0,00	0,77	0,03	0,37	0,00
2037342		0,84	0,38	1,27	0,24	1,08	0,70	1,48	0,06	0,92	0,71	1,21	0,34	0,32	0,00	0,80	0,01	0,45	0,00
2039001		0,77	0,00	0,72	0,00	0,61	0,00	1,36	0,00	1,03	0,74	1,06	0,64	1,23	0,06	0,97	0,81	0,68	0,00
2040831		0,64	0,00	0,54	0,00	0,48	0,00	1,14	0,10	0,90	0,40	0,93	0,69	1,07	0,39	1,02	0,89	0,59	0,00
2042668		0,59	0,00	0,37	0,00	0,28	0,00	1,00	0,96	0,70	0,02	0,69	0,05	0,55	0,26	0,99	0,99	0,30	0,10
2044505		0,54	0,00	0,36	0,00	0,26	0,00	0,75	0,05	0,55	0,01	0,50	0,01	0,20	0,32	0,30	0,38	0,12	0,28
2048120		0,69	0,04	1,53	0,15	2,01	0,02	0,12	0,37	0,06	0,47	0,06	0,47	0,30	0,12	0,42	0,18	0,37	0,15
2051821		0,53	0,00	0,90	0,55	1,08	0,59	0,64	0,07	0,86	0,57	0,64	0,06	0,56	0,01	0,69	0,04	0,60	0,01
2055531		0,51	0,00	0,90	0,38	0,90	0,38	0,70	0,00	1,26	0,37	0,87	0,43	0,53	0,06	0,48	0,09	0,60	0,10

576672		0,97	0,90	0,81	0,33	1,26	0,01	1,15	0,02	1,39	0,05	1,37	0,06
580959		0,93	0,38	0,90	0,11	0,83	0,34	1,16	0,29	1,12	0,63	0,95	0,83
582682		0,87	0,27	0,99	0,94	1,46	0,00	1,47	0,00	1,85	0,00	3,11	0,00
584346	PRDX3,HSPB1	0,91	0,02	0,92	0,03	1,10	0,76	1,29	0,38	1,21	0,73	1,27	0,65
586647	DC1L2,TCPB	0,98	0,67	0,96	0,32	1,84	0,09	1,71	0,14	2,32	0,04	2,37	0,03
589226		0,97	0,54	1,03	0,47	0,99	0,97	0,97	0,86	0,90	0,74	0,82	0,55
589630		0,91	0,01	0,96	0,20	1,52	0,15	2,22	0,01	1.000,00	N,A,	1.000,00	N,A,
594301		0,87	0,02	0,82	0,01	0,77	0,02	0,86	0,11	0,52	0,00	0,55	0,00
596190	HS90A	0,95	0,36	0,73	0,00	0,50	0,00	1,23	0,17	0,70	0,00	0,79	0,01
1763758		5,71	0,00	15,83	0,22	16,01	0,15	0,48	0,17	0,12	0,15	0,33	0,27
1763762		4,26	0,14	1,47	0,17	31,87	0,14	3,36	0,05	1.000,00	0,39	1.000,00	0,04
1763803		1,49	0,41	0,21	0,04	2,06	0,11	1,52	0,27	1,19	0,84	2,23	0,45
1764738		0,55	0,37	1,23	0,70	1,83	0,45	0,83	0,85	1,380,322	0,07	1,099,346	0,13
1764993		0,46	0,34	0,41	0,38	3,15	0,22	0,08	0,00	0,15	0,02	196,84	0,43
1765003		0,08	0,34	0,01	0,22	3,24	0,10	0,17	0,17	0,00	0,01	0,00	0,06
1780185		25,66	0,03	0,00	0,54	0,47	0,48	0,29	0,35	3,32	0,33	2,27	0,29
1785181		1,42	0,46	0,00	0,01	1,56	0,28	1,49	0,25	0,81	0,84	0,23	0,31
1785527		1,48	0,25	0,00	0,01	1,13	0,78	1,52	0,25	21,78	0,32	1,66	0,69
1787194		7,20	0,02	0,00	0,24	0,74	0,52	0,73	0,43	12,46	0,40	0,87	0,85
1788919		1,53	0,01	0,00	0,00	1,13	0,54	1,01	0,96	1,87	0,19	1,21	0,77
1792282		1,50	0,24	0,00	0,00	1,34	0,28	1,49	0,10	0,79	0,66	0,49	0,40
1793947		0,87	0,79	0,27	0,10	1,60	0,07	1,97	0,00	1,34	0,50	1,11	0,73
1797353		1,00	0,99	0,18	0,00	1,45	0,12	1,11	0,63	0,63	0,48	0,65	0,47
1799192		0,77	0,01	0,44	0,10	0,83	0,26	0,51	0,01	0,70	0,15	0,30	0,01
1800806		1,05	0,92	0,41	0,21	5,42	0,01	1,93	0,21	3,22	0,52	12,15	0,08
1802534		0,91	0,30	0,37	0,02	0,80	0,55	0,50	0,16	0,72	0,47	0,19	0,06
1804168		0,44	0,00	0,57	0,00	0,55	0,24	0,79	0,59	0,83	0,22	1,52	0,02
1809464		0,23	0,05	0,91	0,78	1,82	0,54	1,41	0,76	51,50	0,13	220,91	0,10
1811207		0,54	0,01	0,79	0,13	0,76	0,18	0,98	0,90	0,81	0,57	0,54	0,17
1814025		0,53	0,18	0,12	0,01	2,18	0,09	0,62	0,34	0,00	0,12	0,09	0,03
1814733		0,90	0,65	0,35	0,00	1,31	0,51	1,03	0,92	0,11	0,21	0,07	0,19
1814782		0,80	0,00	0,80	0,00	0,93	0,36	0,95	0,52	1,02	0,55	0,70	0,00
1816422		0,91	0,43	0,14	0,00	1,35	0,64	0,70	0,26	0,11	0,04	0,13	0,04
1819121		0,79	0,02	0,51	0,00	0,81	0,40	0,90	0,61	0,34	0,05	0,33	0,05
1819877		0,97	0,91	0,34	0,02	0,95	0,91	0,74	0,28	0,21	0,08	0,26	0,09
1821608		0,81	0,18	0,67	0,00	0,87	0,60	0,83	0,33	0,41	0,02	0,48	0,02
1825144	RCN2	1,14	0,01	0,95	0,26	0,89	0,21	0,82	0,08	0,91	0,66	0,71	0,21
1828633	ALDOA	0,77	0,00	0,82	0,02	0,87	0,10	0,74	0,01	0,97	0,75	0,65	0,02
1837103		0,82	0,10	1,01	0,98	1,01	0,95	0,85	0,15	0,94	0,64	2,40	0,44
1840030		0,83	0,12	0,90	0,56	1,09	0,50	0,81	0,10	1,08	0,70	2,22	0,11
1841741		0,53	0,00	0,87	0,67	1,30	0,15	0,83	0,12	1,49	0,18	1,97	0,34
1843456		0,80	0,41	8,49	0,18	1,79	0,06	0,92	0,57	1,78	0,05	2,43	0,37
1844173		0,72	0,00	0,63	0,00	0,74	0,00	0,82	0,01	0,96	0,59	0,80	0,01
1846930		2,14	0,00	1,19	0,32	0,45	0,03	1,09	0,67	1,02	0,96	1,05	0,85
1847793		0,95	0,75	0,48	0,00	0,70	0,13	0,50	0,01	0,44	0,00	0,53	0,00
1864524		1,24	0,05	1,20	0,16	0,66	0,07	1,11	0,65	0,71	0,00	0,62	0,01
1864541		0,68	0,12	0,66	0,01	1,00	0,99	0,59	0,00	0,42	0,05	0,64	0,21
1866181		1,77	0,36	0,44	0,16	5,48	0,03	1,57	0,34	1,15	0,81	0,02	0,01
1867746		0,78	0,01	0,78	0,02	1,26	0,16	0,94	0,72	2,11	0,00	0,93	0,80
1871260		0,44	0,08	0,79	0,49	1,05	0,92	1,20	0,69	1,78	0,00	0,60	0,28
1911469		0,98	0,65	0,72	0,00	1,03	0,84	1,12	0,45	1,06	0,58	1,09	0,56
1913428	ELOB	3,22	0,44	2,26	0,11	3,09	0,04	5,37	0,00	2,86	0,02	4,52	0,25
1915149		0,12	0,03	0,03	0,02	2,15	0,23	0,49	0,44	10,53	0,20	2,25	0,33
1931678		1,14	0,22	2,24	0,01	1,25	0,05	1,41	0,01	1,17	0,16	2,84	0,00
1933424		1,84	0,00	4,25	0,02	1,38	0,03	1,76	0,01	1,77	0,01	5,50	0,00
1935188		1,35	0,05	2,61	0,03	1,00	0,99	0,84	0,34	1,38	0,11	3,01	0,00
1936868	MRLC2	1,77	0,12	1,56	0,46	1,54	0,42	17,31	0,19	1,20	0,77	1,93	0,08
1953484	MLRN	0,85	0,23	0,86	0,27	0,88	0,47	0,71	0,12	0,67	0,16	0,74	0,26
1960958		0,87	0,19	0,51	0,04	0,55	0,40	0,58	0,43	0,39	0,28	0,31	0,23
1962730	LEG1	1,38	0,00	1,50	0,01	0,94	0,10	1,13	0,01	1,24	0,00	1,79	0,00
1964508		0,96	0,65	1,49	0,00	1,17	0,30	1,28	0,03	1,21	0,27	2,36	0,00
1978481		1,22	0,06	1,02	0,73	1,12	0,57	2,54	0,00	0,84	0,34	0,88	0,54
1980208		1,91	0,02	1,53	0,12	3,03	0,03	1,32	0,48	6,96	0,01	3,61	0,30
1980598		1,16	0,86	0,28	0,25	2,99	0,08	0,63	0,52	0,16	0,31	0,01	0,15
1982157		1,05	0,56	0,76	0,01	0,81	0,14	0,43	0,00	0,65	0,11	0,57	0,06
1983973		1,49	0,04	17,66	0,33	0,61	0,34	1,21	0,61	0,62	0,46	0,51	0,37
1985723		1,58	0,41	1,48	0,48	1,28	0,37	1,12	0,68	1,22	0,54	1,42	0,43
1987569		3,16	0,07	6,29	0,01	0,79	0,40	0,58	0,10	1,17	0,67	2,95	0,08
1989367		1,49	0,01	2,37	0,00	1,27	0,10	1,41	0,03	1,37	0,04	2,15	0,02
1991205		0,56	0,06	2,67	0,01	0,00	N,A,	1,19	0,58	0,00	N,A,	12,42	0,04
1993029		4,22	N,A,	3,61	N,A,	0,00	N,A,	1,27	0,40	0,00	N,A,	0,00	N,A,
1994828		1,01	0,95	5,04	0,00	1,22	N,A,	1,63	0,00	1,89	N,A,	12,96	0,02
1996662		0,00	N,A,	0,27	N,A,	0,00	N,A,	1,10	0,88	0,00	N,A,	0,00	N,A,
1996674		0,00	N,A,	0,18	N,A,	0,00	N,A,	0,88	N,A,	0,00	N,A,	0,00	N,A,
1998321		1,14	0,19	0,95	0,65	0,94	0,70	1,09	0,45	1,74	0,12	1,61	0,18
2000013	TCTP	0,34	0,00	0,21	0,00	1,00	0,98	0,42	0,00	0,72	0,13	0,55	0,02
2001699	CPNS1	0,95	0,46	0,92	0,27	0,88	0,12	0,96	0,40	1,01	0,89	1,13	0,01
2003479	AN32A	1,34	0,00	1,19	0,01	0,70	0,01	0,44	0,00	0,62	0,00	0,63	0,00
2005865		1,66	0,02	1,21	0,00	1,07	0,73	0,98	0,91	1,10	0,36	1,33	0,08
2006762	CALU	2,21	0,11	0,95	0,92	1,42	0,59	1,07	0,87	0,27	0,08	1,47	0,69
2008587		1,38	0,20	0,70	0,33	1,20	0,57	1,38	0,03	1,11	0,69	1,67	0,52
2010414	BCCIP,ACTG	1,06	0,78	0,71	0,25	0,86	0,32	1,38	0,01	1,29	0,28	0,89	0,70
2016883		1,03	0,86	0,59	0,02	0,65	0,00	1,31	0,03	1,14	0,31	0,92	0,61
2019749		1,04	0,58	0,70	0,00	0,79	0,00	0,69	0,00	0,97	0,63	0,72	0,00
2021577		1,04	0,07	0,87	0,00	0,64	0,02	0,73	0,05	0,63	0,05	0,63	0,06
2023391		1,02	0,31	0,89	0,00	0,73	0,01	0,83	0,07	0,90	0,07	0,87	0,07
2024619		3,16	0,00	1,25	0,49	0,62	0,36	0,52	0,21	0,12	0,00	0,46	0,09
2027812		1,57	0,57	8,88	0,21	1,71	0,33	0,92	0,45	0,00	0,01	0,87	0,83
2028269		0,91	0,43	1,19	0,41	0,94	0,68	1,66	0,00	1,36	0,06	1,93	0,00
2030105		1,96	0,02	1,43	0,14	0,75	0,17	1,12	0,39	0,58	0,01	1,03	0,88
2031934		1,62	0,18	1,48	0,08	0,76	0,29	1,38	0,08	0,67	0,13	1,10	0,67
2033833	ID1737021	1,07	0,51	1,18	0,12	1,39	0,17	5,32	0,00	0,48	0,01	0,32	0,00
2035512		1,50	0,02	1,34	0,10	0,54	0,00	2,45	0,00	0,83	0,11	1,24	0,06
2037342		1,23	0,02	0,96	0,55	0,51	0,00	1,62	0,00	0,78	0,01	1,06	0,32
2039001		1,55	0,00	1,74	0,00	1,24	0,05	1,46	0,03	1,52	0,04	1,21	0,05
2040831		1,74	0,00	2,00	0,00	1,63	0,03	2,28	0,02	1,90	0,09	1,48	0,05
2042668		1,82	0,00	2,02	0,00	2,02	0,02	5,79	0,11	2,64	0,03	2,28	0,00
2044505		1,63	0,00	1,54	0,01	1,80	0,01	3,45	0,03	2,33	0,01	2,00	0,00
2048120		2,26	0,16	0,89	0,35	0,52	0,20	0,55	0,08	0,39	0,07	0,37	0,01
2051821		1,07	0,73	0,80	0,08	0,83	0,53	0,57	0,05	0,52	0,03	0,41	0,01
2055531		1,00	0,98	0,81	0,14	1,02	0,93	0,42	0,02	0,71	0,18	0,53	0,01

2057395		0,62	0,00	0,65	0,00	0,63	0,00	0,74	0,00	0,68	0,00	0,57	0,00	0,52	0,00	0,71	0,04	0,41	0,00	1,10	0,12	1,09	0,19	0,95	0,39	1,42	0,00	0,84	0,02	0,86	0,03
2060826		0,62	0,00	0,78	0,06	0,68	0,00	0,76	0,00	0,81	0,03	0,61	0,00	0,47	0,02	0,62	0,07	0,36	0,01	1,01	0,82	1,07	0,32	0,86	0,32	1,12	0,35	0,75	0,00	0,74	0,00
2062951		0,35	0,00	0,31	0,00	0,15	0,00	0,44	0,00	0,71	0,46	0,24	0,00	0,34	0,00	0,23	0,00	0,18	0,00	1,10	0,50	1,19	0,30	2,00	0,38	0,92	0,72	1,37	0,35	1,39	0,27
2064822		0,44	0,00	0,52	0,00	0,37	0,00	0,51	0,00	0,59	0,12	0,32	0,00	0,52	0,00	0,50	0,00	0,36	0,00	1,03	0,76	1,07	0,54	1,01	0,98	0,87	0,49	0,76	0,26	0,87	0,49
2068313		0,49	0,00	0,50	0,00	0,24	0,00	0,58	0,00	0,46	0,00	0,40	0,00	0,43	0,00	0,48	0,00	0,33	0,00	1,31	0,00	1,19	0,05	1,00	0,99	1,27	0,08	1,80	0,00	1,83	0,00
2068580		0,62	0,00	0,90	0,43	1,22	0,16	0,70	0,00	1,05	0,89	1,03	0,94	0,40	0,01	0,54	0,03	0,50	0,02	0,95	0,08	0,63	0,00	0,99	0,98	0,58	0,04	0,72	0,40	0,40	0,00
2070460		0,41	0,00	0,17	0,00	0,04	0,00	0,58	0,01	0,67	0,26	0,17	0,00	0,07	0,06	0,20	0,10	0,05	0,06	1,18	0,28	0,50	0,00	3,20	0,13	3,48	0,00	4,02	0,07	4,47	0,10
2072345		0,56	0,00	0,27	0,00	0,05	0,00	0,70	0,00	0,71	0,04	0,45	0,00	0,17	0,00	0,39	0,00	0,22	0,00	1,16	0,06	0,55	0,00	2,48	0,02	2,64	0,01	7,73	0,00	7,29	0,01
2074209		0,86	0,02	1,02	0,78	1,11	0,48	0,88	0,00	0,92	0,40	1,07	0,60	0,93	0,58	0,96	0,78	0,79	0,11	1,05	0,28	0,98	0,78	0,93	0,54	0,86	0,15	1,00	0,98	0,65	0,05
2076081		0,79	0,00	1,01	0,84	1,12	0,30	0,86	0,03	0,83	0,13	1,10	0,66	1,19	0,24	1,28	0,10	1,14	0,50	1,13	0,05	1,06	0,46	0,85	0,21	0,90	0,25	1,01	0,97	0,72	0,12
2081733		0,55	0,00	0,38	0,00	0,15	0,00	0,75	0,00	0,51	0,00	0,48	0,00	0,42	0,00	0,54	0,00	0,29	0,00	1,39	0,00	1,08	0,36	1,36	0,10	2,00	0,00	3,23	0,00	2,69	0,00
2083630		0,50	0,00	0,39	0,00	0,15	0,00	0,73	0,00	0,46	0,00	0,47	0,00	0,50	0,00	0,62	0,00	0,34	0,00	1,52	0,00	1,22	0,03	1,23	0,16	1,97	0,00	3,28	0,00	2,78	0,00
2085530		0,61	0,00	0,49	0,00	0,18	0,00	0,75	0,04	0,65	0,00	0,56	0,00	0,37	0,00	0,44	0,00	0,27	0,00	1,15	0,27	0,86	0,07	1,25	0,12	1,28	0,21	2,89	0,01	2,06	0,03
2087348		0,44	0,00	0,37	0,00	0,23	0,00	0,55	0,00	0,54	0,05	0,33	0,00	0,98	0,83	1,16	0,24	0,59	0,02	1,23	0,09	1,60	0,00	1,45	0,39	2,26	0,00	1,40	0,07	1,83	0,03
2089255		0,80	0,00	0,80	0,02	0,84	0,32	0,76	0,00	0,79	0,07	0,78	0,05	1,21	0,01	1,34	0,01	1,05	0,60	0,96	0,40	0,97	0,40	1,00	0,99	1,07	0,49	0,93	0,77	0,80	0,31
2091139		0,84	0,06	0,81	0,26	0,85	0,55	0,86	0,23	1,15	0,63	1,00	0,99	1,88	0,01	2,20	0,00	1,68	0,02	0,93	0,36	0,80	0,06	1,27	0,48	0,97	0,85	1,06	0,88	0,70	0,32
2093028		0,83	0,06	0,82	0,25	0,93	0,76	0,89	0,26	1,54	0,28	1,45	0,33	1,47	0,02	2,01	0,00	1,45	0,02	0,97	0,65	0,82	0,01	1,72	0,22	1,15	0,41	1,42	0,47	0,72	0,26
2094921		0,83	0,01	0,86	0,32	1,20	0,50	0,91	0,31	1,26	0,29	1,11	0,56	1,15	0,51	1,64	0,02	1,06	0,79	0,94	0,36	0,81	0,05	1,26	0,36	1,12	0,47	0,79	0,48	0,52	0,09
2096810		0,83	0,04	0,87	0,35	1,25	0,34	0,81	0,05	0,91	0,55	0,81	0,17	1,10	0,76	1,48	0,24	0,99	0,97	0,96	0,56	0,83	0,05	1,02	0,92	1,05	0,79	0,64	0,14	0,49	0,04
2099065		0,74	0,22	0,65	0,03	0,68	0,08	0,91	0,08	1,18	0,03	1,37	0,01	1,09	0,12	1,01	0,85	1,18	0,02	0,86	0,51	0,90	0,62	1,26	0,02	0,94	0,36	1,40	0,03	1,05	0,41
2100931		8,61	0,10	7,28	0,03	8,92	0,07	1,31	0,28	2,74	0,00	3,31	0,00	0,95	0,79	0,93	0,33	1,33	0,12	0,44	0,28	0,53	0,36	1,10	0,75	0,62	0,23	1,08	0,83	0,72	0,50
2102478		1,75	0,09	1,84	0,06	2,18	0,06	0,78	0,13	0,67	0,11	0,51	0,01	0,80	0,13	1,00	0,99	0,69	0,05	0,93	0,59	0,84	0,22	0,76	0,21	1,01	0,93	0,49	0,03	0,59	0,04
2105992		0,32	0,00	0,40	0,00	0,18	0,00	0,66	0,00	0,38	0,00	0,25	0,00	0,38	0,01	0,49	0,03	0,20	0,00	1,95	0,00	1,52	0,04	0,88	0,61	1,57	0,03	1,30	0,58	1,47	0,30
2106278		0,25	0,00	0,30	0,00	0,10	0,00	0,67	0,01	0,37	0,00	0,08	0,00	0,39	0,00	0,49	0,01	0,14	0,00	1,97	0,00	1,33	0,11	0,93	0,75	1,40	0,04	0,64	0,38	1,25	0,39
2108161		0,08	0,18	0,33	0,31	0,30	0,37	0,75	0,19	2,72	0,37	0,07	0,00	0,79	0,15	0,86	0,26	0,27	0,01	5,04	0,00	5,04	0,01	4,59	0,28	1,39	0,29	0,12	0,01	0,47	0,20
2110071		0,31	0,00	0,32	0,00	0,12	0,00	0,64	0,01	0,59	0,03	0,13	0,00	0,46	0,00	0,52	0,00	0,16	0,00	1,52	0,01	1,25	0,02	1,40	0,27	1,40	0,03	0,81	0,67	1,17	0,60
2113599		0,79	0,03	0,70	0,01	0,45	0,00	0,72	0,01	0,49	0,00	0,45	0,00	0,52	0,00	0,65	0,00	0,40	0,00	1,01	0,95	0,99	0,93	0,78	0,12	1,41	0,01	1,11	0,66	1,33	0,17
2115505		0,63	0,00	0,55	0,01	0,58	0,01	0,94	0,61	0,87	0,31	0,77	0,09	0,45	0,00	0,65	0,00	0,35	0,00	1,39	0,02	1,40	0,01	1,48	0,08	2,32	0,00	1,23	0,22	1,16	0,40
2117397	VINC	0,07	0,02	0,30	0,05	0,16	0,05	0,70	0,00	0,05	0,00	0,03	0,00	0,44	0,00	0,68	0,00	0,08	0,00	6,17	0,00	5,19	0,00	0,11	0,00	1,90	0,00	0,14	0,00	0,42	0,01
2138752		1,18	0,67	4,22	0,41	17,57	0,21	2,17	0,00	0,96	0,88	1,68	0,13	1,37	0,41	1,89	0,12	0,89	0,80	1,18	0,52	1,26	0,41	0,15	0,36	0,49	0,57	0,06	0,21	0,06	0,20
2140633		0,46	0,04	0,46	0,04	0,07	0,01	0,47	0,06	1,31	0,59	0,60	0,32	0,77	0,59	0,75	0,56	0,05	0,04	1,03	0,88	1,82	0,07	2,85	0,14	1,78	0,07	9,15	0,18	0,75	0,64
2142601		1,55	0,41	3,96	0,36	1,82	0,24	1,74	0,24	1,14	0,82	1,19	0,74	1,14	0,82	5,59	0,00	0,08	0,17	0,56	0,28	1,15	0,69	0,14	0,30	2,21	0,17	0,33	0,07	0,07	0,02
2148299		1,44	0,28	0,70	0,32	0,79	0,35	1,43	0,12	1,22	0,43	1,69	0,04	0,45	0,00	1,51	0,00	0,28	0,00	0,42	0,05	0,48	0,07	0,74	0,49	3,29	0,00	0,90	0,64	0,55	0,08
2150207		1,28	0,39	0,56	0,03	0,68	0,03	1,47	0,00	1,73	0,00	2,29	0,00	0,40	0,00	1,10	0,15	0,33	0,00	0,40	0,04	0,49	0,06	1,07	0,78	3,06	0,00	1,18	0,10	0,77	0,30
2152149		1,05	0,78	1,07	0,65	0,82	0,34	0,76	0,01	0,96	0,50	0,92	0,33	0,78	0,00	0,85	0,05	0,71	0,00	0,67	0,01	0,72	0,01	0,83	0,02	0,77	0,01	1,05	0,59	0,84	0,09
2158110		1,35	0,01	1,17	0,05	1,02	0,88	1,07	0,40	1,27	0,00	0,94	0,42	1,02	0,72	0,80	0,04	0,79	0,02	0,83	0,04	0,70	0,00	1,14	0,00	0,62	0,00	0,96	0,76	0,71	0,04
2190755		0,40	0,04	0,46	0,00	0,26	0,00	0,06	0,13	0,17	0,18	0,08	0,14	0,45	0,00	0,55	0,00	0,31	0,00	0,34	0,32	0,95	0,93	0,93	0,40	1,00	0,97	0,74	0,17	1,00	0,99
2194613		0,16	0,00	0,40	0,04	0,17	0,01	0,37	0,01	0,35	0,01	0,12	0,00	0,61	0,00	0,73	0,00	0,31	0,00	1,88	0,23	2,28	0,02	0,71	0,55	1,10	0,82	0,58	0,42	1,14	0,87
2198480	PPT1	0,44	0,17	0,11	0,01	0,00	0,02	0,05	0,26	0,16	0,51	0,00	0,43	0,17	0,14	0,74	0,54	0,01	0,16	1,21	0,77	0,53	0,60	14,88	0,00	8,80	0,00	0,00	0,50	66,39	0,28
2201032		0,48	0,06	0,18	0,01	0,54	0,40	0,04	0,01	0,38	0,12	0,00	0,00	0,00	0,34	0,02	0,34	0,00	0,34	0,28	0,11	0,00	0,03	6,88	0,15	1,54	0,60	0,00	0,37	0,00	0,49
2202941		0,68	0,06	1,46	0,19	0,91	0,86	0,73	0,36	1,03	0,91	1,04	0,88	0,48	0,05	0,03	0,00	0,46	0,04	0,80	0,30	0,60	0,04	0,52	0,06	0,02	0,00	0,85	0,79	0,43	0,31
2204207		0,00	0,25	0,06	0,28	0,06	0,36	0,48	N,A,	1,63	N,A,	2,51	N,A,	0,35	0,02	0,53	0,04	0,70	0,50	420,85	N,A,	63,78	0,05	21,61	N,A,	1,47	0,34	34,79	N,A,	2,01	0,50
2209869		1,00	0,99	1,22	0,25	1,21	0,38	0,70	0,00	0,85	0,09	0,72	0,00	1,06	0,65	1,07	0,73	0,84	0,34	0,72	0,01	0,63	0,00	0,72	0,04	0,52	0,01	0,61	0,04	0,41	0,01
2212082	CHP1	0,64	0,00	0,65	0,00	0,15	0,00	0,84	0,02	0,70	0,00	0,64	0,00	0,64	0,00	0,71	0,00	0,43	0,00	1,23	0,03	1,03	0,61	1,02	0,84	1,14	0,26	4,04	0,00	2,98	0,00
2214019		0,09	0,32	0,02	0,28	0,00	0,36	0,10	0,12	0,18	0,21	0,00	0,14	0,02	0,00	0,55	0,10	0,02	0,00	0,44	0,50	0,03	0,32	3,08	0,21	2,89	0,17	0,00	0,37	7,89	0,19
2215889		0,59	0,06	0,76	0,13	0,49	0,01	0,78	0,08	0,88	0,45	0,49	0,00	0,58	0,02	0,77	0,09	0,45	0,01	1,16	0,57	1,06	0,83	1,00	0,99	1,08	0,57	0,88	0,54	0,99	0,98
2218268		0,02	0,14	0,29	0,24	0,00	0,13	0,00	0,30	0,78	0,83	0,00	0,16	178,35	0,36	0,00	0,25	0,00	0,44	0,06	0,52	6,090,258	0,44	7,78	0,23	0,00	0,30	0,00	0,16	0,02	0,28
2221628		0,53	0,00	0,53	0,01	0,28	0,01	0,72	0,06	0,64	0,02	0,57	0,01	0,74	0,01	1,10	0,30	0,42	0,00	1,37	0,07	1,43	0,00	1,23	0,29	2,16	0,00	2,08	0,09	1,55	0,27
2275539		0,82	0,28	0,49	0,19	0,00	0,01	0,90	0,08	0,94	0,29	0,72	0,00	0,72	0,01	0,85	0,17	0,57	0,01	0,91	0,47	0,75	0,08	1,57	0,39	1,47	0,48	1,853,653	0,00	1,508,842	0,02
2287478	SFRS3	0,67	0,00	0,70	0,02	0,27	0,00	0,67	0,00	0,72	0,01	0,44	0,00	0,60	0,00	0,81	0,00	0,46	0,00	0,85	0,13	0,70	0,03	0,87	0,44	0,91	0,48	1,40	0,17	1,33	0,45
2288948		0,07	0,08	0,67	0,65	0,00	N,A,	0,38	0,01	0,75	0,30	0,40	0,01	0,56	0,01	0,87	0,13	0,01	0,00	5,27	0,06	6,30	0,01	1,06	0,90	1,01	0,95	1.000,00	N,A,	1.000,00	N,A,
2290995		0,33	0,11	1,09	0,89	0,42	0,23	0,20	0,02	0,50	0,12	0,09	0,01	0,00	0,34	0,00	0,34	0,00	0,34	0,41	0,08	0,03	0,01	0,31	0,22	0,00	0,09	0,15	0,06	0,05	0,04
2292906		0,62	0,02	1,04	0,90	0,70	0,11	0,73	0,00	0,78	0,09	0,49	0,00	0,61	0,11	0,42	0,04	0,57	0,10	0,79	0,01	0,57	0,00	0,50	0,09	0,23	0,02	0,47	0,00	0,46	0,01
2296834		0,07	0,05	0,30	0,19	0,00	0,14	0,00	0,08	1,56	0,64	0,00	0,08	0,00	0,17	0,08	0,20	0,01	0,24	0,00	0,11	0,00	0,11	4,58	0,36	0,18	0,15	ð	ð	1.000,00	0,49
2298466	ERP29,IF4E	0,26	0,02	0,42	0,03	0,40	0,06	0,91	0,85	1,81	0,34	0,57	0,45	0,99	0,98	1,13	0,50	6,17	0,30	2,01	0,04	0,84	0,75	2,48	0,14	0,59	0,01	0,82	0,66	3,39	0,46
2300514		2,95	0,09	1,61	0,39	3,26	0,12	2,82	0,21	3,49	0,01	5,03	0,07	3,38	0,05	5,68	0,00	6,85	0,04	0,69	0,47	0,43	0,11	1,56	0,15	1,34	0,33	1,11	0,86	0,80	0,66
2302886		0,37	0,03	0,84	0,47	0,47	0,07	1,03	0,94	1,48	0,09	0,09	0,00	1,42	0,51	4,23	0,00	1,65	0,45	2,25	0,19	2,91	0,15	1,43	0,11	3,84	0,00	0,17	0,00	2,71	0,31
2304779		0,63	0,12	0,88	0,08	0,75	0,01	0,81	0,12	0,83	0,15	0,87	0,34	0,72	0,00	0,95	0,07	0,78	0,00	1,21	0,53	0,93	0,82	0,89	0,09	0,87	0,07	1,10	0,46	0,85	0,17
2305694		0,74	0,22	0,93	0,02	0,85	0,03	0,82	0,14	0,94	0,59	0,87	0,27	0,81	0,01	0,79	0,01	0,82	0,02	1,10	0,72	0,99	0,96	0,99	0,81	0,76	0,00	1,00	0,99	0,86	0,10
2308652		0,40	0,00	0,41	0,00	0,30	0,00	0,40	0,00	0,37	0,00	0,36	0,00	0,48	0,00	0,60	0,00	0,39	0,00	1,24	0,42	1,23	0,42	1,13	0,35	1,52	0,01	1,48	0,06	1,34	0,13
2314456		7,19	0,02	1,14	0,76	3,56	0,04	1,29	0,70	0,73	0,38	0,86	0,64	1,54	0,16	1,37	0,05	1,88	0,00	0,33	0,08	0,26	0,03	1,19	0,55	1,45	0,20	0,45	0,07	0,64	0,19
2315592		1,03	0,42	0,88	0,11	0,70	0,01	1,13	0,09	1,00	0,99	0,86	0,03	1,02	0,79	1,15	0,05	0,98	0,76	0,91	0,05	0,78	0,00	0,94	0,43	1,02	0,77	1,01	0,89	1,10	0,36
2318312		0,90	0,44	0,99	0,91	0,77	0,10	0,88	0,23	0,74	0,00	0,58	0,00	0,45	0,00	0,51	0,00	0,39	0,00	1,22	0,19	1,03	0,74	0,92	0,11	1,07	0,41	0,94	0,45	1,04	0,62
2329037		0,90	0,01	1,11	0,02	1,01	0,78	1,00	0,87	0,86	0,00	0,73	0,00	0,64	0,00	0,82	0,00	0,64	0,00	1,14	0,00	0,98	0,36	0,79	0,00	1,01	0,80	0,75	0,00	0,86	0,00
2340727		0,71	0,11	0,92	0,77	0,49	0,03	1,10	0,57	1,80	0,00	1,29	0,07	0,99	0,95	1,35	0,16	1,18	0,18	1,02	0,90	1,02	0,88	1,29	0,35	1,07	0,82	1,74	0,02	1,76	0,01
2350362		0,95	0,33	1,29	0,00	1,21	0,01	1,00	0,99	0,87	0,00	0,74	0,00	0,58	0,00	0,73	0,00	0,55	0,00	1,24	0,00	1,15	0,01	0,79	0,00	1,05	0,23	0,72	0,00	0,85	0,01
2352301		0,88	0,01	1,17	0,01	1,10	0,05	1,07	0,17	0,95	0,14	0,79	0,00	0,72	0,00	0,93	0,05	0,67	0,00	1,35	0,00	1,13	0,04	0,89	0,03	1,09	0,05	0,79	0,00	0,84	0,01
2354041		1,44	0,00	1,43	0,00	1,71	0,00	1,32	0,00	0,89	0,00	1,00	0,89	0,98	0,57	1,33	0,00	0,98	0,64	1,17	0,00	0,98	0,24	0,80	0,00	1,33	0,00	0,75	0,00	0,82	0,00
2382844	PPM1G	0,79	0,00	0,78	0,00	0,75	0,01	0,76	0,00	0,64	0,00	0,63	0,00	0,71	0,00	0,83	0,01	0,55	0,00	1,03	0,39	0,87	0,09	0,88	0,04	1,04	0,45	0,89	0,25	0,72	0,02
2404447	NP1L4	1,46	0,00	1,86	0,00	2,11	0,00	1,38	0,00	1,66	0,00	2,33	0,00	1,64	0,00	1,94	0,00	2,01	0,00	0,89	0,00	0,93	0,01	0,83	0,00	0,86	0,00	1,03	0,23	0,79	0,00
2439918		0,85	0,09	1,04	0,92	1,44	0,53	0,81	0,04	0,71	0,01	0,65	0,00	0,97	0,92	1,17	0,53	0,76	0,38	1,07	0,19	1,07	0,10	0,77	0,46	1,06	0,84	0,51	0,32	0,49	0,31
2447768		1,44	0,01	1,57	0,08	1,34	0,35	1,23	0,03	1,00	0,98	1,09	0,39	0,49	0,00	1,45	0,06	0,51	0,00	1,07	0,29	0,97	0,68	0,80	0,34	2,62	0,00	1,02	0,93	1,08	0,74
2549343		0,66	0,01	0,56	0,02	0,56	0,09	0,70	0,03	0,72	0,02	0,72	0,19	1,15	0,56	0,90	0,63	0,96	0,93	1,92	0,00	3,15	0,00	2,35	0,00	2,91	0,00	2,34	0,13	3,12	0,26
2555205		0,49	0,01	0,43	0,01	0,42	0,03	0,71	0,31	0,47	0,04	0,27	0,02	0,24	0,09	0,57	0,31	0,19	0,08	2,98	0,03	0,79	0,30	2,24	0,01	2,16	0,01	1,32	0,63	0,75	0,41
2588249	ANXA2,NACA	1,00	0,99	1,06	0,88	0,58	0,23	0,74	0,32	1,33	0,12	0,70	0,32	0,36	0,15	0,67	0,45	0,61	0,45	0,73	0,34	0,56	0,12	1,23	0,48	1,00	1,00	1,18	0,75	1,66	0,55
2592168		0,03	0,02	0,57	0,36	0,32	0,06	0,21	0,07	1,21	0,67	0,00	0,09	0,00	0,34	0,79	0,83	0,00	0,34	7,51	0,48	0,14	0,44	2,10	0,27	22,25	0,15	0,00	0,10	0,00	0,04
2596060		1,49	0,00	1,27	0,08	0,99	0,93	1,37	0,00	1,27	0,00	1,13	0,19	1,11	0,03	1,40	0,00	1,25	0,02	0,78	0,02	0,55	0,00	0,85	0,18	0,82	0,12	0,97	0,82	0,94	0,65
2617667		1,04	0,67	1,17	0,06	0,88	0,13	0,76	0,29	0,74	0,16	0,48	0,02	0,96	0,88	0,76	0,13	0,91	0,61	1,11	0,59	0,87	0,54	0,95	0,56	0,61	0,00	0,82	0,13	0,96	0,83
2649893		0,84	0,00	1,04	0,56	0,87	0,06	0,81	0,00	0,78	0,00	0,75	0,00	0,65	0,01	0,83	0,22	0,60	0,00	0,80	0,00	0,78	0,00	0,62	0,00	0,80	0,11	0,72	0,01	0,69	0,00
2662678	RCN1	1,24	0,00	1,36	0,00	1,57	0,00	1,15	0,01	0,86	0,01	1,07	0,17	0,91	0,02	1,21	0,00	0,93	0,01	1,04	0,20	0,91	0,03	0,72	0,00	1,11	0,03	0,77	0,00	0,73	0,00
2666660	SPRC	0,93	0,02	1,44	0,00	1,35	0,00	1,03	0,50	0,88	0,01	0,83	0,00	0,60	0,00	0,72	0,00	0,60	0,00	1,34	0,00	1,20	0,00	0,74	0,00	0,92	0,14	0,74	0,00	0,82	0,00
2680471	RCN1,SPRC	1,12	0,04	1,31	0,01	1,59	0,00	1,21	0,00	0,91	0,03	0,93	0,12	0,83	0,00	0,89	0,07	0,77	0,00	1,13	0,02	0,98	0,66	0,73	0,00	0,91	0,25	0,61	0,00	0,65	0,00
2687300	RCN1	1,52	0,00	1,87	0,00	2,30	0,00	1,55	0,00	1,16	0,04	1,36	0,00	0,92	0,33	1,05	0,53	0,95	0,53	1,27	0,00	1,12	0,06	0,77	0,05	1,05	0,64	0,74	0,00	0,77	0,00
2722863		0,20	0,10	0,22	0,12	0,05	0,10	0,24	0,02	1,02	0,96	1,33	0,43	0,29	0,00	0,93	0,45	0,15	0,00	0,27	0,12	1,15	0,78	1,02	0,98	3,40	0,01	5,30	0,03	2,12	0,28
2728784		0,43	0,14	1,69	0,49	1,41	0,40	1,41	0,12	1,46	0,47	2,53	0,00	0,19	0,34	0,96	0,97	0,43	0,50	1,54	0,10	0,80	0,54	0,41	0,32	1,06	0,93	0,85	0,42	0,57	0,08
2729506		3,12	0,00	4,19	0,00	6,68	0,00	2,01	0,00	3,42	0,00	5,69	0,00	2,39	0,00	2,08	0,00	3,22	0,00	0,66	0,00	0,88	0,07	0,83	0,00	0,57	0,00	0,87	0,02	0,55	0,00
2737054		0,43	0,00	0,26	0,00	0,07	0,00	0,34	0,06	0,94	0,86	0,72	0,39	0,21	0,00	0,71	0,00	0,14	0,00	0,22	0,00	0,53	0,01	1,03	0,93	3,09	0,00	2,77	0,06	2,19	0,37
2740977		1,54	0,00	1,73	0,00	2,06	0,00	1,42	0,00	1,14	0,02	1,34	0,00	0,96	0,12	1,25	0,00	1,03	0,29	0,91	0,00	0,78	0,00	0,65	0,00	0,91	0,01	0,64	0,00	0,63	0,00
2742902		1,26	0,00	1,24	0,00	1,39	0,00	1,25	0,00	1,04	0,29	1,15	0,00	0,98	0,83	1,21	0,03	0,91	0,31	0,94	0,03	0,89	0,00	0,79	0,00	1,12	0,01	0,78	0,00	0,75	0,00
2784333		0,66	0,00	1,07	0,55	1,39	0,13	0,74	0,00	1,08	0,85	0,80	0,29	0,66	0,03	1,05	0,66	0,74	0,03	1,04	0,76	0,84	0,30	0,92	0,84	0,82	0,14	0,53	0,07	0,45	0,02
2788379		0,72	0,00	0,76	0,00	0,63	0,00	0,76	0,00	0,73	0,00	0,62	0,00	0,65	0,00	0,78	0,00	0,61	0,00	1,05	0,02	0,89	0,00	0,94	0,46	1,00	0,94	0,97	0,57	0,95	0,21
2816455	HS90A,HS90B	0,92	0,12	0,83	0,06	0,81	0,01	0,92	0,00	0,92	0,01	0,77	0,00	1,15	0,01	0,87	0,12	1,10	0,05	1,03	0,28	0,93	0,05	1,15	0,12	0,78	0,07	0,98	0,48	1,01	0,59
2818369	P3H1	0,87	0,01	0,88	0,04	0,76	0,00	0,91	0,04	0,82	0,00	0,70	0,00	0,93	0,03	1,06	0,03	0,89	0,00	1,00	1,00	0,82	0,00	0,90	0,12	0,93	0,19	0,89	0,01	0,90	0,01
2830369		0,83	0,00	0,96	0,52	0,69	0,00	0,90	0,02	0,92	0,04	0,71	0,00	0,93	0,06	0,97	0,47	0,97	0,17	0,92	0,00	0,75	0,00	0,80	0,01	0,67	0,00	0,87	0,00	0,93	0,04
2838327		0,76	0,02	0,81	0,04	0,57	0,00	0,82	0,00	0,90	0,23	0,64	0,00	0,95	0,27	0,96	0,38	0,94	0,14	0,83	0,08	0,74	0,02	0,86	0,20	0,71	0,01	0,86	0,04	0,98	0,38
2845794	GRP78	1,39	0,00	1,40	0,00	1,49	0,00	1,09	0,00	1,03	0,46	0,97	0,40	1,00	0,87	1,02	0,52	0,86	0,00	0,78	0,00	0,79	0,00	0,73	0,00	0,80	0,00	0,65	0,00	0,63	0,00
2846419	GRP78	0,92	0,24	0,94	0,17	1,04	0,49	0,98	0,72	0,75	0,01	0,72	0,00	0,76	0,00	0,92	0,09	0,56	0,00	1,25	0,01	1,15	0,04	0,94	0,33	1,36	0,00	0,82	0,03	0,75	0,02
2880272		1,50	0,01	1,33	0,15	1,52	0,02	0,71	0,10	0,69	0,05	0,82	0,24	0,51	0,00	0,68	0,00	0,42	0,00	0,60	0,00	0,63	0,00	0,66	0,03	0,94	0,68	0,69	0,01	0,51	0,00
2889441		1,20	0,12	1,11	0,50	1,45	0,01	1,36	0,00	1,21	0,00	1,57	0,00	0,69	0,03	1,75	0,00	0,78	0,13	0,80	0,01	0,69	0,00	0,77	0,08	1,89	0,00	0,76	0,00	0,65	0,00
2906459	SET,HDGF	0,81	0,00	0,73	0,00	0,45	0,00	0,96	0,10	0,80	0,00	0,73	0,00	0,78	0,00	0,90	0,09	0,69	0,00	1,03	0,30	0,85	0,00	0,95	0,50	1,08	0,29	1,40	0,00	1,34	0,01
2912567		0,78	0,45	0,83	0,57	0,89	0,78	1,14	0,33	0,98	0,85	1,05	0,52	1,16	0,10	1,19	0,18	1,78	0,07	1,37	0,06	1,10	0,32	1,10	0,59	1,07	0,68	1,10	0,64	1,48	0,28

2917416		1,03	0,68	0,76	0,10	0,32	0,00	1,16	0,25	1,01	0,86	0,90	0,34	1,01	0,90	1,15	0,23	1,06	0,51	1,03	0,82	0,76	0,01	1,23	0,21	1,18	0,38	2,57	0,01	2,55	0,01
2924537		0,95	0,24	1,06	0,15	0,78	0,00	0,91	0,01	1,10	0,08	0,83	0,00	1,21	0,03	1,11	0,23	1,39	0,01	0,82	0,00	0,76	0,00	0,88	0,03	0,63	0,00	0,90	0,05	1,06	0,53
2926520		0,67	0,14	0,74	0,17	0,84	0,48	1,09	0,63	1,44	0,13	1,09	0,65	2,18	0,00	2,30	0,03	3,17	0,01	0,97	0,89	1,49	0,03	1,17	0,38	1,42	0,17	0,78	0,23	1,74	0,13
2944561		0,83	0,31	0,78	0,33	0,70	0,17	0,94	0,65	1,60	0,00	1,22	0,19	1,35	0,14	1,59	0,03	1,08	0,70	0,72	0,04	0,65	0,01	1,30	0,19	0,81	0,39	1,12	0,56	0,63	0,08
2964442	HNRPC,STRAP	0,74	0,03	0,70	0,00	0,60	0,00	0,73	0,00	1,00	0,96	0,76	0,00	1,28	0,15	1,22	0,30	1,36	0,11	0,79	0,09	0,86	0,23	1,14	0,08	0,87	0,11	1,00	0,95	1,12	0,23
2970570		0,88	0,12	1,12	0,44	1,01	0,95	1,16	0,66	0,71	0,41	0,84	0,65	0,84	0,26	1,03	0,70	0,90	0,70	1,86	0,01	1,04	0,81	0,90	0,49	1,00	0,99	1,17	0,52	0,97	0,94
2974603		0,82	0,00	0,86	0,00	0,65	0,00	0,94	0,38	0,77	0,01	0,82	0,04	0,84	0,00	0,98	0,54	0,67	0,00	1,14	0,00	1,15	0,00	0,88	0,00	1,29	0,00	1,24	0,03	1,16	0,01
2977290		0,39	0,00	0,82	0,29	0,27	0,00	0,20	0,00	0,92	0,79	0,51	0,10	0,11	0,00	0,41	0,06	0,27	0,01	0,26	0,00	0,17	0,00	0,57	0,07	0,30	0,01	0,97	0,94	0,61	0,06
2990527		0,00	0,14	0,10	0,17	0,02	0,22	0,00	0,18	0,51	0,25	0,01	0,03	0,16	0,02	0,55	0,16	0,00	0,01	0,00	0,47	11,70	0,40	2,08	0,23	0,81	0,77	0,09	0,33	0,00	0,37
2995227	SFRS1	1,00	0,96	0,72	0,01	0,44	0,00	0,63	0,08	0,44	0,01	0,64	0,11	1,02	0,84	0,86	0,25	0,72	0,06	0,91	0,48	0,86	0,08	0,87	0,20	1,01	0,93	2,07	0,04	1,38	0,20
2997219		0,74	0,00	0,78	0,01	0,52	0,00	0,75	0,13	1,21	0,43	1,03	0,90	0,27	0,03	0,89	0,73	0,59	0,18	0,46	0,00	0,19	0,00	0,71	0,06	0,60	0,02	0,91	0,72	0,59	0,03
3019322		0,64	0,03	0,57	0,07	0,35	0,01	1,08	0,79	0,80	0,32	0,74	0,19	0,40	0,01	0,93	0,82	0,29	0,01	1,74	0,13	1,23	0,15	1,44	0,25	3,26	0,03	2,20	0,04	1,69	0,16
3022983		0,50	0,00	1,15	0,08	0,68	0,01	0,41	0,00	0,81	0,01	0,23	0,00	0,33	0,00	0,42	0,00	0,24	0,00	0,81	0,08	0,67	0,00	0,69	0,00	0,37	0,00	0,33	0,00	0,37	0,00
3040913		0,84	0,14	0,84	0,23	0,61	0,03	1,25	0,03	1,11	0,55	0,69	0,08	4,15	0,00	2,23	0,01	3,24	0,00	1,05	0,66	0,78	0,03	0,94	0,75	0,42	0,01	0,80	0,50	0,84	0,43
3046761		0,94	0,08	0,96	0,32	0,75	0,00	0,80	0,07	0,79	0,03	0,54	0,00	0,68	0,05	0,81	0,17	0,65	0,04	0,77	0,03	0,49	0,00	0,74	0,01	0,58	0,00	0,65	0,00	0,59	0,00
3056834	PRDX4	1,41	0,01	1,23	0,01	1,46	0,04	1,22	0,01	1,08	0,32	1,12	0,17	2,04	0,00	2,22	0,00	1,97	0,00	0,91	0,24	0,90	0,24	0,93	0,23	1,12	0,12	0,80	0,14	0,84	0,18
3058865	PSB6	0,88	0,13	1,07	0,37	0,62	0,02	0,86	0,02	0,83	0,00	0,72	0,00	0,70	0,00	0,89	0,01	0,55	0,00	1,04	0,54	0,98	0,77	0,83	0,01	1,05	0,37	1,24	0,15	1,10	0,50
3062814		0,85	0,27	1,28	0,08	1,12	0,63	0,86	0,38	0,85	0,38	0,65	0,07	0,57	0,00	0,74	0,00	0,58	0,00	1,24	0,11	0,79	0,19	0,80	0,14	0,68	0,01	0,70	0,22	0,61	0,12
3068832		1,16	0,14	1,15	0,09	0,72	0,01	1,16	0,17	0,90	0,34	0,91	0,39	0,75	0,01	1,05	0,52	0,82	0,06	0,75	0,01	0,56	0,00	0,59	0,00	0,79	0,01	0,95	0,37	0,99	0,85
3078712		1,31	0,49	1,39	0,24	1,21	0,55	1,27	0,56	1,47	0,30	1,33	0,37	1,13	0,69	0,77	0,55	1,59	0,22	0,58	0,21	0,83	0,56	0,63	0,07	0,54	0,05	0,65	0,09	1,26	0,46
3085623	TMOD3	0,71	0,09	1,01	0,94	0,99	0,93	0,72	0,37	0,68	0,30	0,93	0,82	0,67	0,03	0,58	0,04	0,76	0,15	1,11	0,67	0,72	0,19	0,74	0,06	0,44	0,00	1,03	0,92	0,59	0,06
3091679		1,25	0,49	2,02	0,03	1,65	0,25	0,89	0,75	1,57	0,12	1,11	0,71	0,84	0,27	0,75	0,09	0,77	0,26	0,60	0,13	1,02	0,92	0,65	0,06	0,56	0,02	0,56	0,16	0,71	0,37
3102815	PR56A,TXND5	0,86	0,00	0,97	0,38	0,89	0,07	0,89	0,01	0,78	0,00	0,68	0,00	0,69	0,00	0,81	0,00	0,60	0,00	1,02	0,59	0,87	0,00	0,80	0,00	0,91	0,10	0,75	0,01	0,74	0,01
3111980	TBB2C	0,77	0,00	0,86	0,01	0,67	0,00	0,78	0,00	0,76	0,00	0,66	0,00	0,64	0,00	0,77	0,00	0,61	0,00	0,99	0,41	0,85	0,00	0,85	0,01	0,91	0,05	0,96	0,22	0,93	0,08
3128108	RD23B	1,95	0,01	2,35	0,00	3,17	0,00	1,42	0,06	2,08	0,00	3,42	0,00	1,64	0,07	1,72	0,05	2,28	0,00	0,65	0,00	0,88	0,08	0,80	0,04	0,77	0,02	0,97	0,77	0,75	0,00
3130260		0,53	0,00	0,49	0,00	0,31	0,00	0,56	0,00	0,54	0,00	0,41	0,00	0,52	0,00	0,60	0,00	0,55	0,00	1,01	0,91	0,84	0,05	1,06	0,30	1,05	0,67	1,27	0,02	1,52	0,00
3163353		1,22	0,01	1,19	0,01	0,86	0,07	0,99	0,66	0,81	0,00	0,74	0,00	0,91	0,15	0,98	0,77	0,67	0,00	0,99	0,88	1,01	0,85	0,84	0,00	1,12	0,08	1,06	0,22	1,05	0,48
3170569		1,39	0,00	1,28	0,03	1,12	0,07	1,21	0,00	0,77	0,00	0,85	0,01	1,04	0,36	1,06	0,09	0,85	0,00	1,05	0,36	1,06	0,32	0,73	0,01	1,19	0,05	0,91	0,15	1,08	0,07
3184881		0,87	0,02	1,03	0,58	1,01	0,87	1,04	0,81	1,54	0,00	1,90	0,01	0,85	0,20	1,15	0,24	1,09	0,48	0,80	0,05	0,84	0,01	1,01	0,84	0,95	0,42	1,27	0,16	0,92	0,35
3228910	SAKS1,SAE1	1,04	0,42	1,08	0,32	1,11	0,29	1,02	0,77	0,94	0,33	0,97	0,62	0,96	0,40	1,11	0,08	1,08	0,26	0,91	0,04	0,84	0,01	0,80	0,03	0,93	0,37	0,81	0,06	0,89	0,28
3231778		0,86	0,01	0,75	0,00	0,54	0,00	0,95	0,09	0,82	0,00	0,67	0,00	0,73	0,00	0,92	0,10	0,72	0,00	0,96	0,34	0,79	0,01	0,94	0,07	1,14	0,02	1,06	0,41	1,24	0,02
3248968		0,95	0,55	1,03	0,74	1,07	0,66	1,09	0,31	0,87	0,13	1,05	0,58	1,28	0,00	1,55	0,00	1,35	0,00	1,14	0,12	1,12	0,12	0,84	0,07	1,26	0,02	0,97	0,86	1,05	0,76
3255127	ACTG	1,08	0,16	1,15	0,05	1,09	0,23	1,15	0,00	1,13	0,09	0,97	0,44	0,88	0,00	1,29	0,00	1,01	0,74	0,81	0,00	0,72	0,00	0,75	0,01	0,98	0,81	0,68	0,00	0,82	0,03
3259231		0,96	0,17	1,12	0,09	0,70	0,01	1,04	0,08	1,06	0,03	0,93	0,14	0,81	0,01	1,11	0,04	0,92	0,12	0,98	0,39	0,79	0,00	0,85	0,02	0,93	0,23	1,19	0,12	1,22	0,08
3261240		0,76	0,01	0,81	0,03	0,54	0,00	0,86	0,09	0,70	0,00	0,71	0,00	0,78	0,01	0,94	0,31	0,65	0,00	1,22	0,01	1,18	0,04	0,92	0,16	1,32	0,01	1,42	0,02	1,37	0,03
3270147	CBX5,CATB	0,99	0,93	1,17	0,32	1,28	0,39	1,37	0,05	1,12	0,13	1,59	0,00	0,90	0,03	1,29	0,00	0,88	0,15	1,56	0,02	1,53	0,00	1,09	0,54	1,87	0,00	1,41	0,16	1,17	0,54
3275754		0,32	0,00	0,69	0,11	0,18	0,00	0,49	0,00	0,79	0,23	0,37	0,00	0,29	0,00	0,45	0,00	0,17	0,00	1,34	0,20	1,12	0,42	1,01	0,97	0,80	0,42	1,81	0,17	1,20	0,76
3296127	GMFB	0,76	0,00	0,89	0,02	0,61	0,00	1,22	0,00	0,89	0,00	0,98	0,69	1,12	0,00	1,31	0,00	1,07	0,01	1,48	0,00	1,63	0,00	0,94	0,14	1,65	0,00	1,49	0,00	1,94	0,00
3313934	PSME2,1433E	1,44	0,00	1,33	0,01	1,76	0,00	1,36	0,00	1,04	0,23	1,17	0,00	1,13	0,01	1,31	0,00	1,13	0,10	0,93	0,19	0,81	0,01	0,77	0,01	1,02	0,81	0,65	0,01	0,66	0,02
3329884	ARP3,TADBP	1,28	0,41	1,64	0,00	1,55	0,02	1,30	0,01	1,29	0,01	1,53	0,00	1,22	0,00	1,17	0,03	1,26	0,00	0,82	0,47	0,84	0,52	0,63	0,00	0,63	0,00	0,80	0,00	0,72	0,00
3348523	DCUP	0,84	0,06	0,76	0,00	0,50	0,00	1,05	0,37	0,86	0,03	0,84	0,01	0,83	0,00	1,14	0,01	0,67	0,00	1,00	0,99	1,02	0,84	0,90	0,03	1,53	0,00	1,34	0,00	1,37	0,00
3352574		0,65	0,08	0,89	0,31	0,64	0,02	0,88	0,02	0,78	0,00	0,65	0,00	0,75	0,00	0,84	0,00	0,68	0,00	1,26	0,32	1,16	0,52	0,82	0,09	0,96	0,63	0,94	0,58	1,07	0,52
3369954	TADBP,PPME1	0,76	0,72	0,56	0,30	0,23	0,09	0,00	0,13	0,00	N,A,	0,00	N,A,	0,96	0,93	0,67	0,01	0,00	N,A,	0,04	0,43	1,57	0,60	0,00	N,A,	1,49	0,21	0,00	N,A,	0,00	N,A,
3392731	CNDP2,PEPD	0,74	0,01	0,85	0,02	0,69	0,00	0,92	0,01	0,85	0,00	0,80	0,00	0,81	0,00	0,99	0,78	0,68	0,00	1,15	0,14	1,18	0,08	0,92	0,20	1,26	0,00	1,06	0,23	1,06	0,11
3395583		0,81	0,01	0,83	0,19	1,14	0,58	0,60	0,15	0,54	0,07	0,49	0,05	0,91	0,66	0,89	0,34	0,85	0,13	0,93	0,70	0,99	0,97	0,81	0,23	0,95	0,75	0,53	0,08	0,66	0,18
3403522		13,17	0,38	0,16	0,21	0,10	0,26	1,12	0,20	1,25	0,11	1,07	0,71	0,92	0,48	1,15	0,01	1,20	0,17	0,01	0,35	0,01	0,35	0,61	0,01	0,53	0,00	0,81	0,29	0,86	0,25
3413662		0,54	0,00	0,81	0,10	1,01	0,81	0,72	0,00	0,77	0,00	0,85	0,00	0,49	0,00	0,78	0,06	0,45	0,00	1,48	0,00	1,61	0,00	1,06	0,64	1,73	0,01	0,94	0,12	0,80	0,00
3432480		0,96	0,24	1,58	0,42	3,16	0,17	0,97	0,33	1,59	0,00	2,11	0,00	1,44	0,00	1,27	0,01	1,85	0,00	0,88	0,01	1,09	0,08	0,87	0,78	0,58	0,36	0,58	0,37	0,42	0,23
3435858	QCR1	0,86	0,41	0,65	0,02	0,56	0,00	0,26	0,24	2,59	0,48	17,97	0,13	0,37	0,01	0,93	0,66	2,85	0,33	0,28	0,04	0,31	0,02	3,66	0,32	1,04	0,88	29,60	0,12	3,68	0,37
3464756		0,65	0,03	0,54	0,04	0,42	0,04	0,88	0,54	0,83	0,49	0,43	0,01	0,90	0,67	0,15	0,00	1,36	0,01	1,43	0,18	0,82	0,39	1,63	0,25	0,17	0,04	1,10	0,85	1,94	0,07
3477702		2,20	0,00	2,87	0,00	3,83	0,00	1,99	0,00	2,87	0,00	4,54	0,00	1,70	0,00	2,60	0,00	2,59	0,00	0,70	0,00	0,69	0,00	0,78	0,00	0,81	0,00	0,92	0,02	0,61	0,00
3479720		2,36	0,00	3,22	0,00	4,49	0,00	2,52	0,00	4,49	0,00	7,39	0,00	2,16	0,00	2,90	0,00	2,72	0,00	0,56	0,00	0,84	0,01	0,73	0,01	0,83	0,04	0,86	0,30	0,56	0,01
3483954		1,52	0,00	2,02	0,00	2,57	0,00	1,23	0,05	1,67	0,00	2,25	0,00	1,57	0,00	1,90	0,00	1,90	0,00	0,83	0,00	0,93	0,01	0,84	0,00	0,84	0,00	0,89	0,05	0,66	0,00
3485928		1,58	0,00	2,20	0,00	3,54	0,00	1,37	0,00	1,26	0,02	1,60	0,00	1,00	0,98	1,61	0,00	1,23	0,01	0,83	0,01	0,76	0,00	0,55	0,00	0,88	0,04	0,43	0,00	0,42	0,00
3497145		0,35	0,00	1,15	0,27	0,35	0,00	0,30	0,00	0,83	0,04	0,21	0,00	0,18	0,00	0,21	0,00	0,06	0,00	1,01	0,92	0,52	0,00	0,85	0,24	0,18	0,00	0,72	0,26	0,16	0,01
3499167		0,06	0,03	0,21	0,07	0,00	0,05	0,00	0,01	0,43	0,08	0,00	0,01	0,00	0,05	0,00	0,24	0,00	0,05	0,00	0,33	0,01	0,34	1,48	0,48	0,00	0,26	1.000,00	0,44	1.000,00	0,44
3543802		0,78	0,00	0,95	0,21	0,74	0,00	0,98	0,81	0,97	0,28	0,73	0,00	0,85	0,01	1,01	0,76	0,86	0,02	1,00	1,00	0,77	0,00	0,81	0,00	0,76	0,00	0,79	0,01	0,83	0,03
3549612		2,58	0,00	3,10	0,00	4,54	0,00	2,33	0,00	2,47	0,00	4,23	0,00	2,37	0,00	3,58	0,00	3,59	0,00	0,96	0,59	0,82	0,04	0,84	0,02	1,04	0,56	0,98	0,69	0,71	0,00
3557801		0,86	0,19	0,65	0,01	0,77	0,02	0,87	0,22	0,68	0,02	0,69	0,02	0,98	0,30	1,10	0,01	0,83	0,00	1,07	0,54	0,85	0,16	1,10	0,51	1,25	0,05	0,94	0,23	0,79	0,00
3591281	IF1AX,SORCN	0,68	0,04	0,95	0,83	0,47	0,09	0,98	0,81	0,79	0,02	0,89	0,28	0,43	0,01	1,36	0,12	0,58	0,01	1,75	0,01	0,84	0,63	1,00	0,99	1,87	0,05	2,28	0,07	1,61	0,38
3591287		0,08	0,18	0,37	0,35	0,04	0,24	3,54	0,36	0,20	0,06	0,95	0,90	0,69	0,58	5,77	0,28	0,41	0,26	232,67	0,21	31,60	0,04	2,78	0,08	55,60	0,20	122,32	0,01	37,06	0,01
3597623		0,98	0,62	1,09	0,48	0,86	0,24	1,14	0,02	0,91	0,05	0,72	0,00	1,08	0,06	1,02	0,60	0,94	0,13	1,06	0,13	0,97	0,36	0,76	0,06	0,82	0,12	0,76	0,10	0,96	0,76
3661699	ANXA1,DCPS	0,70	0,00	0,89	0,08	0,69	0,00	0,88	0,00	0,81	0,00	0,70	0,00	0,72	0,00	0,82	0,00	0,59	0,00	1,01	0,76	0,92	0,13	0,73	0,00	0,83	0,01	0,81	0,03	0,76	0,02
3668269		0,78	0,00	1,13	0,06	0,62	0,00	0,86	0,00	0,88	0,01	0,58	0,00	0,52	0,00	0,68	0,00	0,35	0,00	1,21	0,00	0,94	0,47	0,85	0,03	0,84	0,06	1,04	0,71	0,79	0,06
3711681		0,86	0,05	0,82	0,02	0,49	0,00	0,86	0,00	0,88	0,00	0,55	0,00	0,94	0,02	0,69	0,00	1,04	0,11	0,71	0,00	0,50	0,00	0,76	0,00	0,39	0,00	0,81	0,04	0,97	0,68
3761682		1,04	0,77	1,10	0,71	1,18	0,47	1,11	0,51	1,13	0,42	1,45	0,03	0,74	0,02	1,02	0,90	0,62	0,01	0,97	0,79	1,08	0,44	0,93	0,78	1,40	0,18	1,12	0,55	0,80	0,38
3761869		0,50	0,00	0,86	0,57	1,20	0,55	0,43	0,00	0,74	0,63	0,49	0,16	0,39	0,02	0,48	0,05	0,35	0,01	1,14	0,34	0,96	0,73	1,13	0,88	0,68	0,31	0,53	0,38	0,36	0,05
3762259		0,68	0,00	0,74	0,01	0,52	0,00	0,72	0,00	0,71	0,00	0,58	0,00	0,68	0,00	0,84	0,01	0,64	0,00	1,00	0,97	0,87	0,16	0,90	0,18	1,00	0,99	1,06	0,07	1,09	0,01
3762281	KU86	0,66	0,00	0,64	0,00	0,49	0,00	0,79	0,00	0,69	0,00	0,67	0,00	0,81	0,00	0,88	0,01	0,68	0,00	1,09	0,03	1,02	0,54	0,99	0,74	1,15	0,01	1,25	0,00	1,14	0,00
3762560	SF3A3	0,86	0,00	0,83	0,00	0,69	0,00	0,85	0,00	0,71	0,00	0,66	0,00	0,73	0,00	0,81	0,00	0,65	0,00	1,00	0,89	0,86	0,00	0,87	0,01	1,00	0,89	0,96	0,27	0,95	0,34
3763668		0,55	0,00	0,63	0,00	0,40	0,00	0,71	0,06	0,69	0,04	0,47	0,01	0,59	0,00	0,77	0,00	0,56	0,00	1,03	0,82	0,75	0,03	0,88	0,08	0,87	0,02	0,95	0,54	1,00	1,00
3763685		0,80	0,00	0,78	0,00	0,57	0,00	0,84	0,00	0,71	0,00	0,64	0,00	0,70	0,00	0,86	0,02	0,70	0,00	1,01	0,69	0,75	0,00	0,87	0,09	0,94	0,40	1,09	0,06	1,07	0,32
3763695		1,01	0,97	0,95	0,76	1,44	0,08	1,15	0,22	1,13	0,14	0,98	0,84	1,28	0,04	1,49	0,00	0,75	0,14	1,05	0,67	1,09	0,32	1,08	0,33	1,33	0,01	0,62	0,02	0,45	0,01
3763705		0,94	0,59	0,75	0,08	0,74	0,08	1,24	0,00	1,30	0,00	1,44	0,00	0,49	0,00	1,29	0,03	0,56	0,01	0,68	0,01	0,57	0,00	0,88	0,28	1,86	0,00	1,00	0,94	0,83	0,19
3763878		0,39	0,27	0,71	0,68	1,12	0,90	0,63	0,18	0,00	N,A,	0,00	N,A,	0,58	0,03	0,49	0,01	0,67	0,16	0,70	0,49	0,82	0,57	0,00	N,A,	0,38	0,36	0,00	N,A,	0,34	0,37
3764268		0,67	0,00	0,69	0,00	0,79	0,01	0,69	0,00	0,69	0,00	0,57	0,00	0,68	0,00	0,79	0,00	0,68	0,00	1,02	0,86	0,91	0,29	0,98	0,77	1,03	0,71	0,71	0,00	0,76	0,00
3764287		1,17	0,18	1,96	0,00	1,49	0,00	1,03	0,63	1,15	0,23	1,01	0,87	0,78	0,00	1,10	0,01	0,72	0,00	0,80	0,06	0,73	0,02	0,53	0,00	0,61	0,00	0,62	0,00	0,53	0,00
3764290		0,71	0,00	0,97	0,79	1,21	0,00	0,93	0,06	0,98	0,50	1,14	0,00	1,00	0,99	1,12	0,06	1,03	0,61	1,13	0,01	1,07	0,07	0,87	0,27	0,87	0,28	0,81	0,00	0,64	0,00
3764305		1,63	0,00	1,94	0,00	2,49	0,00	1,53	0,00	1,89	0,00	2,65	0,00	1,48	0,00	1,69	0,00	1,86	0,00	0,85	0,00	0,89	0,01	0,89	0,06	0,86	0,02	0,97	0,67	0,74	0,01
3764569		0,80	0,00	0,89	0,06	0,70	0,00	0,68	0,00	0,72	0,00	0,53	0,00	0,52	0,00	0,76	0,01	0,76	0,02	0,76	0,00	0,52	0,00	0,73	0,01	0,69	0,00	0,69	0,01	0,86	0,10
3764601		1,34	0,00	1,07	0,17	0,92	0,10	1,16	0,00	0,89	0,00	0,90	0,00	1,04	0,50	1,17	0,11	0,82	0,02	0,94	0,06	1,02	0,51	0,90	0,04	1,43	0,00	1,06	0,17	1,18	0,02
3764826		0,86	0,05	1,19	0,02	1,08	0,39	0,93	0,33	1,20	0,10	1,36	0,01	0,66	0,17	1,46	0,08	0,70	0,24	1,02	0,81	0,76	0,05	0,96	0,62	1,20	0,03	1,19	0,15	0,64	0,04
3765009	CHM4B,RPB3	0,92	0,03	1,03	0,49	0,79	0,00	0,91	0,08	0,79	0,00	0,72	0,00	0,93	0,05	0,99	0,76	0,77	0,00	1,08	0,09	0,93	0,04	0,83	0,00	0,88	0,02	1,00	0,97	0,90	0,05
3765082		0,92	0,54	1,07	0,76	0,93	0,85	0,85	0,23	0,87	0,18	0,96	0,91	0,83	0,36	0,91	0,57	0,50	0,03	1,22	0,26	1,12	0,43	1,08	0,72	1,06	0,78	1,38	0,59	0,67	0,46
3765346		0,16	0,01	0,36	0,03	0,13	0,02	0,70	0,13	0,92	0,78	0,03	0,00	0,14	0,01	0,35	0,02	0,20	0,02	2,18	0,05	0,69	0,48	1,29	0,47	0,75	0,29	0,13	0,00	1,21	0,86
3765482		0,94	0,56	0,84	0,70	0,62	0,00	1,49	0,02	1,73	0,00	1,41	0,07	1,15	0,67	1,83	0,02	0,81	0,50	1,77	0,01	1,49	0,29	2,30	0,04	2,64	0,03	2,55	0,01	1,60	0,34
3765624		0,88	0,00	1,04	0,42	1,23	0,02	0,96	0,30	0,93	0,18	1,27	0,03	0,87	0,45	1,05	0,76	0,99	0,95	1,00	0,95	0,88	0,15	0,82	0,02	0,90	0,05	0,95	0,63	0,72	0,01
3765667		0,65	0,00	0,73	0,00	0,52	0,00	0,75	0,00	0,68	0,00	0,53	0,00	0,69	0,00	0,79	0,00	0,56	0,00	1,22	0,00	1,23	0,00	0,98	0,77	1,24	0,01	1,07	0,32	1,24	0,01
3765671		0,19	0,00	0,57	0,06	0,17	0,01	0,18	0,11	1,15	0,76	0,46	0,27	0,22	0,00	0,79	0,05	0,25	0,00	0,45	0,12	1,21	0,68	0,90	0,61	1,40	0,07	1,24	0,67	1,48	0,46
3765677		0,68	0,00	0,56	0,00	0,42	0,00	0,79	0,03	0,72	0,01	0,47	0,00	0,65	0,00	0,88	0,00	0,37	0,00	0,92	0,20	0,62	0,00	1,02	0,79	1,01	0,81	0,88	0,62	0,57	0,07
3765683		1,17	0,11	1,31	0,11	1,96	0,15	1,11	0,14	1,29	0,11	1,31	0,00	0,72	0,01	1,03	0,77	0,81	0,09	0,72	0,00	0,72	0,00	0,75	0,13	0,92	0,53	0,50	0,14	0,49	0,14
3765685		0,99	0,70	0,89	0,15	0,94	0,53	0,98	0,37	0,76	0,00	0,78	0,00	0,70	0,00	0,92	0,01	0,61	0,00	1,09	0,01	0,97	0,44	0,94	0,45	1,42	0,00	0,91	0,36	0,90	0,31
3765686		0,83	0,36	0,81	0,28	0,73	0,19	1,05	0,62	0,85	0,16	0,84	0,14	0,97	0,74	1,29	0,03	1,12	0,07	1,20	0,14	0,77	0,09	1,00	0,97	1,04	0,67	1,10	0,47	1,01	0,87
3765692	GRP75	0,86	0,01	0,87	0,07	0,88	0,13	0,81	0,00	0,69	0,00	0,70	0,00	0,83	0,08	1,20	0,04	0,84	0,03	1,01	0,79	0,86	0,07	0,85	0,04	1,22	0,04	0,86	0,06	0,84	0,05
3765696		0,67	0,53	0,46	0,31	2,09	0,22	1,27	0,49	1,61	0,17	1,31	0,42	3,77	0,00	13,71	0,04	2,66	0,01	1,07	0,81	1,47	0,06	1,98	0,05	7,79	0,05	0,35	0,06	0,33	0,05
3765727		0,88	0,10	0,86	0,05	0,79	0,03	0,85	0,00	0,71	0,00	0,86	0,00	0,83	0,00	0,78	0,00	0,74	0,00	0,97	0,52	0,89	0,02	0,83	0,00	0,86	0,00	1,09	0,14	0,87	0,03
3765751		0,96	0,72	1,45	0,01	0,81	0,13	0,80	0,25	1,14	0,42	0,69	0,08	0,73	0,01	0,72	0,02	0,75	0,02	0,74	0,07	0,76	0,04	0,70	0,00	0,50	0,00	0,75	0,06	0,92	0,45
3765802		0,43	0,04	0,35	0,03	0,07	0,02	0,45	0,07	0,57	0,13	0,31	0,03	0,22	0,08	0,36	0,14	0,07	0,04	2,17	0,14	1,27	0,68	3,38	0,03	2,53	0,21	8,68	0,17	2,27	0,19

Lendeckel et al.:
Proteomic changes of tissue-tolerable plasma (TTP) treated airway epithelial cells and their relation to wound healing

Supplementary Table S-2

ID	Protein Labels	Co_24h dose-dep.			Co_48h			Co_72h			Co_24h time-dep.		Pl30s_24h		Pl60s_24h		Pl120s_24h	
		ratio 'PL-30s-24h'	ratio 'PL-1min-24h'	ratio 'PL-120s-24h'	ratio 'PL-30sec-48h'	ratio 'PL-1min-48h'	ratio 'PL-2min-48h'	ratio 'PL-30sec-72h'	ratio 'Pl-1min-72h'	ratio 'PL-2min-72h'	ratio 'Co-48h'	ratio 'Co-72h'	ratio 'PL-30sec-48h'	ratio 'PL-30sec-72h'	ratio 'PL-1min-48h'	ratio 'Pl-1min-72h'	ratio 'PL-2min-48h'	ratio 'PL-2min-72h'
1449	1433B_HUMAN	0.86	0.92	0.74	0.90	0.77	0.68	0.67	0.85	0.58	1.02	1.15	1.07	0.90	0.85	1.05	0.95	0.91
1373	1433E_HUMAN	1.21	1.42	1.82	0.96	0.87	0.78	0.77	0.82	0.72	1.08	1.17	0.85	0.75	0.65	0.67	0.46	0.46
1403	1433E_HUMAN	0.87	0.83	0.68	0.93	0.68	0.64	0.71	0.88	0.59	1.04	1.13	1.11	0.92	0.85	1.20	0.97	0.98
3313934	1433E_HUMAN	1.44	1.33	1.76	1.36	1.04	1.17	1.13	1.31	1.13	0.98	1.03	0.93	0.81	0.77	1.02	0.65	0.66
1449	1433G_HUMAN	0.86	0.92	0.74	0.90	0.77	0.68	0.67	0.85	0.58	1.02	1.15	1.07	0.90	0.85	1.05	0.95	0.91
1438	1433S_HUMAN	0.86	0.87	0.67	0.88	0.73	0.65	0.82	0.85	0.64	1.01	1.00	1.02	0.95	0.84	0.98	0.97	0.96
1429	1433T_HUMAN	0.81	0.78	0.61	0.86	0.66	0.59	0.68	0.85	0.62	1.01	1.04	1.07	0.87	0.86	1.13	0.99	1.06
1429	1433Z_HUMAN	0.81	0.78	0.61	0.86	0.66	0.59	0.68	0.85	0.62	1.01	1.04	1.07	0.87	0.86	1.13	0.99	1.06
768	2AAA_HUMAN	2.41	2.86	3.89	1.87	2.52	3.98	2.28	2.50	2.92	0.92	0.92	0.71	0.87	0.81	0.80	0.94	0.69
828	2AAA_HUMAN	1.77	2.23	2.89	1.42	1.70	2.40	1.66	1.96	2.07	1.02	0.92	0.82	0.87	0.78	0.81	0.85	0.66
3316	2AAA_HUMAN	2.26	2.79	3.86	1.79	2.34	3.56	2.09	2.33	2.66	0.97	0.96	0.77	0.89	0.82	0.80	0.90	0.66
767	2AAB_HUMAN	2.46	3.02	4.17	1.78	2.33	3.84	2.15	2.46	2.92	1.02	1.03	0.74	0.90	0.78	0.84	0.94	0.72
971	2ABA_HUMAN	0.78	0.82	0.59	0.78	0.77	0.69	0.70	0.85	0.68	0.95	0.98	0.96	0.89	0.89	1.02	1.11	1.14
1358	3HIDH_HUMAN	0.67	0.69	0.50	0.62	0.53	0.47	0.69	0.64	0.47	1.12	1.07	1.04	1.09	0.87	1.00	1.04	1.01
1399	3HIDH_HUMAN	0.87	0.86	0.76	0.86	0.69	0.65	0.78	0.78	0.58	1.21	1.29	1.21	1.16	0.97	1.17	1.03	0.98
1399	3HIDH_HUMAN	0.87	0.86	0.76	0.86	0.69	0.65	0.78	0.78	0.58	1.21	1.29	1.21	1.16	0.97	1.17	1.03	0.98
1433	6PGL_HUMAN	1.04	1.28	1.52	1.13	0.98	0.96	0.86	0.97	0.81	0.99	1.13	1.07	0.93	0.76	0.86	0.62	0.60
992	ACL6A_HUMAN	0.60	0.93	0.59	0.75	0.82	0.52	0.83	0.96	0.66	0.95	0.71	1.18	0.98	0.83	0.73	0.83	0.80
203683	ACL6A_HUMAN	1.58	2.39	2.80	1.19	1.28	1.38	1.27	1.80	1.69	0.99	0.86	0.75	0.69	0.53	0.65	0.49	0.52
651	ACPH_HUMAN	0.75	0.79	0.61	0.84	0.71	0.64	0.73	0.85	0.60	1.03	1.07	1.16	1.03	0.92	1.14	1.10	1.05
1058	ACTB_HUMAN	0.51	0.62	0.46	0.69	0.57	0.47	0.74	0.84	0.55	0.92	0.84	1.23	1.21	0.84	1.14	0.93	1.00
1064	ACTB_HUMAN	0.95	0.72	0.80	0.78	0.87	0.73	1.28	0.79	1.23	1.48	0.97	1.22	1.31	1.81	1.06	1.35	1.48
1073	ACTB_HUMAN	1.00	1.28	1.04	0.92	0.91	0.84	0.60	0.94	0.64	0.85	1.16	0.78	0.69	0.60	0.85	0.68	0.71
1078	ACTB_HUMAN	0.69	0.77	0.57	0.84	0.78	0.64	0.69	0.80	0.76	0.87	0.83	1.06	0.83	0.88	0.86	0.97	1.11
1080	ACTB_HUMAN	0.77	0.78	0.62	0.81	0.73	0.59	0.70	0.83	0.65	0.90	0.91	0.95	0.83	0.84	0.97	0.86	0.96
1114	ACTB_HUMAN	1.31	1.70	1.96	1.29	1.55	1.71	1.35	1.53	1.67	0.85	0.78	0.84	0.81	0.78	0.70	0.74	0.67
209100	ACTB_HUMAN	0.76	0.99	0.87	0.88	0.86	0.74	0.48	0.73	0.63	0.87	1.02	1.01	0.65	0.76	0.75	0.75	0.74
360270	ACTB_HUMAN	1.19	1.03	1.00	1.00	0.77	0.74	0.73	0.94	0.87	0.88	0.74	0.73	0.45	0.65	0.68	0.65	0.64
420	ACTG_HUMAN	0.93	0.97	1.28	0.86	0.84	0.77	0.88	0.93	0.69	1.07	1.16	0.99	1.10	0.93	1.11	0.65	0.63
1008	ACTG_HUMAN	0.93	0.98	0.76	0.94	0.95	0.77	0.65	0.91	0.63	0.84	1.10	0.85	0.77	0.81	1.02	0.85	0.90
1008	ACTG_HUMAN	0.93	0.98	0.76	0.94	0.95	0.77	0.65	0.91	0.63	0.84	1.10	0.85	0.77	0.81	1.02	0.85	0.90
1027	ACTG_HUMAN	1.09	1.30	1.47	1.01	0.89	0.84	0.69	0.83	0.68	1.16	1.59	1.07	1.01	0.79	1.02	0.66	0.73
1047	ACTG_HUMAN	0.78	0.95	0.66	0.83	0.85	0.64	0.81	0.85	0.76	0.90	0.79	0.96	0.83	0.80	0.71	0.87	0.91
1055	ACTG_HUMAN	0.83	0.99	0.99	1.03	1.30	1.31	1.33	1.13	1.28	0.63	0.54	0.79	0.87	0.84	0.62	0.84	0.70
1058	ACTG_HUMAN	0.51	0.62	0.46	0.69	0.57	0.47	0.74	0.84	0.55	0.92	0.84	1.23	1.21	0.84	1.14	0.93	1.00
1064	ACTG_HUMAN	0.95	0.72	0.80	0.78	0.87	0.73	1.28	0.79	1.23	1.48	0.97	1.22	1.31	1.81	1.06	1.35	1.48
1067	ACTG_HUMAN	0.95	0.51	0.47	0.76	0.84	0.72	1.36	0.82	1.30	1.52	0.96	1.21	1.37	2.48	1.54	2.30	2.62
1073	ACTG_HUMAN	1.00	1.28	1.04	0.92	0.91	0.84	0.60	0.94	0.64	0.85	1.16	0.78	0.69	0.60	0.85	0.68	0.71
1080	ACTG_HUMAN	0.77	0.78	0.62	0.81	0.73	0.59	0.70	0.83	0.65	0.90	0.91	0.95	0.83	0.84	0.97	0.86	0.96
1119	ACTG_HUMAN	1.51	1.56	2.05	1.33	1.12	1.19	1.18	1.21	1.06	0.98	1.01	0.86	0.79	0.71	0.78	0.57	0.52
1478	ACTG_HUMAN	1.73	1.93	2.47	1.35	1.19	1.11	0.97	1.08	0.90	1.13	1.31	0.89	0.74	0.69	0.73	0.51	0.48
15097	ACTG_HUMAN	0.93	1.05	0.66	0.99	0.90	0.80	0.78	0.98	0.81	0.95	1.05	1.01	0.88	0.81	0.98	1.14	1.30
209100	ACTG_HUMAN	0.76	0.99	0.87	0.88	0.86	0.74	0.48	0.73	0.63	0.87	1.02	1.01	0.65	0.76	0.75	0.75	0.74
360270	ACTG_HUMAN	1.19	1.03	1.00	1.00	0.77	0.74	0.73	0.94	0.87	0.88	0.74	0.73	0.45	0.65	0.68	0.65	0.64
418384	ACTG_HUMAN	0.80	1.00	0.88	0.74	0.67	0.50	0.58	0.52	0.54	1.23	1.32	1.13	0.96	0.82	0.68	0.70	0.81
2010414	ACTG_HUMAN	0.74	0.81	0.69	0.58	0.51	0.66	0.35	0.73	0.40	1.36	1.53	1.06	0.71	0.86	1.38	1.29	0.89
507	ACTN4_HUMAN	0.64	0.62	0.44	0.67	0.62	0.48	0.73	0.79	0.68	1.01	0.80	1.06	0.92	1.01	1.04	1.12	1.25
517	ACTN4_HUMAN	0.80	0.76	0.58	0.76	0.69	0.60	0.63	0.81	0.65	1.01	1.05	0.96	0.83	0.91	1.11	1.05	1.18
518	ACTN4_HUMAN	0.69	0.64	0.47	0.71	0.66	0.53	0.69	0.80	0.69	0.98	0.85	1.00	0.85	1.01	1.06	1.11	1.25
8247	ACTN4_HUMAN	0.59	0.53	0.35	0.71	0.57	0.49	0.71	0.80	0.56	1.09	0.95	1.30	1.12	1.17	1.42	1.52	1.50
8700	ACTN4_HUMAN	0.41	0.56	0.41	1.06	0.71	0.70	0.69	1.02	0.57	0.87	1.59	2.22	2.64	1.11	2.91	1.48	2.24
31063	ACTY_HUMAN	0.63	0.43	0.27	0.69	0.69	0.56	0.69	0.74	0.61	1.06	1.08	1.16	1.19	1.71	1.85	2.20	2.45
1047	ADRM1_HUMAN	0.78	0.95	0.66	0.83	0.85	0.64	0.81	0.85	0.76	0.90	0.79	0.96	0.83	0.80	0.71	0.87	0.91
1052	ADRM1_HUMAN	0.81	0.87	0.55	0.83	0.70	0.63	0.74	0.87	0.70	1.06	0.98	1.08	0.90	0.85	0.98	1.21	1.25

697	AGM1_HUMAN	1.60	1.44	1.82	1.63	2.22	2.57	1.55	1.08	1.80	0.89	1.11	0.91	1.08	1.37	0.84	1.26	1.11
467907	AGM1_HUMAN	0.87	0.59	0.68	1.03	1.35	1.07	0.67	0.53	0.68	0.71	1.11	0.85	0.85	1.63	0.98	1.12	1.10
490428	AGM1_HUMAN	1.01	0.40	0.10	1.05	0.98	0.85	0.96	1.13	0.99	0.99	0.89	1.02	0.84	2.46	2.55	8.80	9.21
894	AL1B1_HUMAN	1.01	0.36	0.09	1.03	0.93	0.87	0.92	1.05	0.91	0.90	0.92	0.92	0.84	2.34	2.70	8.98	9.64
950	AL9A1_HUMAN	0.74	1.02	0.58	0.91	0.97	0.69	0.84	0.95	0.90	0.80	0.72	0.99	0.82	0.76	0.67	0.96	1.12
408351	AL9A1_HUMAN	0.83	1.04	0.64	0.87	0.81	0.60	0.59	0.79	0.58	0.89	1.10	0.94	0.78	0.70	0.84	0.84	0.99
1386	ALBU_HUMAN	0.75	0.72	0.61	0.78	0.82	0.65	0.73	0.85	0.66	0.96	0.91	1.00	0.89	1.09	1.07	1.04	0.99
1828633	ALDOA_HUMAN	1.95	2.75	3.24	1.54	2.45	3.23	1.83	2.32	2.38	0.97	0.88	0.77	0.82	0.87	0.74	0.97	0.65
384077	AMPM2_HUMAN	0.79	0.83	0.70	0.86	0.90	0.95	0.65	0.87	0.82	0.88	1.06	0.96	0.87	0.94	1.11	1.19	1.23
2003479	AN32A_HUMAN	0.73	1.29	1.08	0.77	0.71	0.53	1.19	0.77	0.94	1.27	0.73	1.34	1.19	0.70	0.44	0.62	0.63
3661699	ANXA1_HUMAN	0.70	0.89	0.69	0.88	0.81	0.70	0.72	0.82	0.59	0.81	0.90	1.01	0.92	0.73	0.83	0.81	0.76
1173	ANXA2_HUMAN	0.80	0.90	0.93	0.72	0.72	0.64	0.59	0.73	0.63	0.94	0.97	0.84	0.70	0.75	0.78	0.64	0.65
1254	ANXA2_HUMAN	1.46	1.40	1.13	1.32	1.16	0.98	1.17	1.30	1.17	0.88	0.74	0.80	0.59	0.74	0.69	0.77	0.77
825	ANXA3_HUMAN	1.23	1.50	1.84	1.08	1.23	1.54	1.17	1.27	1.32	1.02	0.97	0.90	0.92	0.84	0.82	0.86	0.70
1351	ANXA3_HUMAN	0.77	0.81	0.59	0.78	0.78	0.63	0.81	0.79	0.74	0.98	0.90	1.00	0.95	0.93	0.88	1.04	1.13
17069	ANXA3_HUMAN	0.81	0.65	0.41	0.78	0.64	0.58	0.81	0.71	0.63	1.27	1.17	1.22	1.16	1.26	1.27	1.83	1.81
1343	ANXA4_HUMAN	0.91	1.06	1.16	0.87	0.83	0.76	0.88	0.86	0.81	0.98	0.90	0.92	0.87	0.76	0.73	0.64	0.63
1325	ANXA5_HUMAN	0.71	0.89	0.70	0.74	0.78	0.64	0.69	0.78	0.61	0.96	0.94	0.99	0.91	0.84	0.82	0.87	0.82
1345	ANXA5_HUMAN	1.51	1.54	1.93	1.40	1.05	1.33	0.86	1.31	0.84	1.12	1.54	1.04	0.88	0.77	1.31	0.77	0.67
213992	ANXA5_HUMAN	0.76	0.84	0.57	0.79	0.73	0.63	0.65	0.66	0.56	0.96	1.02	0.99	0.86	0.82	0.80	1.06	0.99
22159	ANXA6_HUMAN	1.47	2.16	2.40	1.47	1.91	2.97	1.38	1.80	2.07	0.81	0.96	0.81	0.90	0.72	0.80	1.00	0.83
983	APMAP_HUMAN	0.83	0.84	0.65	0.79	0.79	0.68	0.74	0.88	0.69	0.95	0.94	0.91	0.84	0.88	0.98	0.99	0.99
341707	APT_HUMAN	1.83	0.22	0.19	1.07	0.76	0.81	1.11	0.74	0.95	0.26	0.23	0.15	0.14	0.89	0.77	1.11	1.16
964	ARP3_HUMAN	0.80	0.80	0.62	0.83	0.65	0.64	0.74	0.87	0.65	1.06	1.03	1.10	0.96	0.86	1.13	1.09	1.08
965	ARP3_HUMAN	0.61	0.70	0.42	0.68	0.68	0.51	0.65	0.74	0.60	0.95	0.89	1.05	0.94	0.92	0.94	1.16	1.28
1046	ARP3_HUMAN	0.81	1.00	0.80	0.85	0.77	0.69	0.64	0.73	0.57	1.03	1.27	1.08	1.01	0.79	0.92	0.89	0.90
1078	ARP3_HUMAN	0.69	0.77	0.57	0.84	0.78	0.64	0.69	0.80	0.76	0.87	0.83	1.06	0.83	0.88	0.86	0.97	1.11
3329884	ARP3_HUMAN	1.28	1.64	1.55	1.30	1.29	1.53	1.22	1.17	1.26	0.81	0.88	0.82	0.84	0.63	0.63	0.80	0.72
1121	ASNA_HUMAN	0.75	0.73	0.53	0.77	0.76	0.58	0.75	0.85	0.69	0.98	0.92	1.01	0.92	1.02	1.07	1.07	1.20
1152	ASNA_HUMAN	0.86	0.78	0.66	0.86	0.71	0.70	0.78	0.84	0.69	1.08	1.07	1.09	0.98	0.98	1.17	1.14	1.11
927	ATPB_HUMAN	1.18	1.30	1.25	1.14	0.94	0.92	0.92	1.11	0.81	0.95	1.02	0.92	0.80	0.69	0.87	0.70	0.66
1047	ATPB_HUMAN	0.78	0.95	0.66	0.83	0.85	0.64	0.81	0.85	0.76	0.90	0.79	0.96	0.83	0.80	0.71	0.87	0.91
3737	ATPB_HUMAN	1.41	1.33	1.62	1.30	0.90	1.02	1.00	1.30	0.96	1.00	1.04	0.92	0.74	0.68	1.02	0.63	0.61
302071	ATPB_HUMAN	1.77	1.99	3.68	1.42	1.06	1.14	0.85	0.99	0.74	0.95	1.13	0.76	0.54	0.51	0.56	0.30	0.23
936	BASP1_HUMAN	0.85	0.68	0.55	0.97	0.60	0.51	1.06	0.95	0.65	1.21	0.97	1.37	1.20	1.07	1.35	1.12	1.14
48368	BASP1_HUMAN	0.81	0.45	0.35	0.90	0.55	0.48	0.54	0.68	0.34	1.05	1.32	1.16	0.86	1.29	2.01	1.46	1.28
2010414	BCCIP_HUMAN	0.74	0.81	0.69	0.58	0.51	0.66	0.35	0.73	0.40	1.36	1.53	1.06	0.71	0.86	1.38	1.29	0.89
1596	BID_HUMAN	0.69	0.69	0.49	0.79	0.81	0.65	0.68	0.71	0.50	0.83	0.72	0.97	0.72	0.98	0.75	1.12	0.74
934	BLMH_HUMAN	0.77	0.32	0.02	0.81	0.71	0.61	0.70	0.81	0.61	0.97	1.10	1.02	1.00	2.19	2.79	68.19	76.90
951	BLMH_HUMAN	0.74	0.69	0.59	0.80	0.79	0.73	0.71	0.86	0.71	0.94	1.01	1.01	0.97	1.08	1.26	1.16	1.22
624	CALD1_HUMAN	0.89	0.27	0.00	0.84	0.76	0.70	0.72	0.87	0.69	0.99	1.08	0.94	0.88	2.83	3.50	°	°
644	CALD1_HUMAN	0.92	1.40	2.13	0.84	0.91	0.80	0.64	0.89	0.86	0.83	0.92	0.75	0.64	0.54	0.59	0.31	0.37
28319	CALD1_HUMAN	0.68	0.60	6.91	0.76	0.74	0.62	0.79	0.87	0.86	0.89	0.85	0.99	0.98	1.09	1.23	0.08	0.11
488759	CALD1_HUMAN	0.55	0.21	5.76	0.52	0.47	0.42	0.63	0.73	0.56	1.05	0.91	0.99	1.05	2.41	3.23	0.08	0.09
745	CALR_HUMAN	2.02	2.75	3.59	1.80	2.49	3.64	1.96	2.46	2.60	0.88	0.91	0.78	0.88	0.80	0.81	0.89	0.66
1027	CALU_HUMAN	1.09	1.30	1.47	1.01	0.89	0.84	0.69	0.83	0.68	1.16	1.59	1.07	1.01	0.79	1.02	0.66	0.73
53317	CALU_HUMAN	1.06	1.23	1.37	1.03	0.88	0.83	0.62	0.77	0.63	1.08	1.55	1.05	0.90	0.77	0.97	0.66	0.72
54018	CALU_HUMAN	1.05	1.21	1.20	0.84	0.74	0.69	0.48	0.64	0.51	1.04	1.43	0.83	0.65	0.64	0.76	0.60	0.61
2006762	CALU_HUMAN	0.44	0.77	0.25	0.15	0.18	0.01	0.31	0.62	0.28	6.28	1.33	2.21	0.95	1.42	1.07	0.27	1.47
479360	CAP2_HUMAN	0.99	0.39	0.21	0.93	0.90	0.83	0.91	1.01	0.87	1.06	1.06	1.00	0.98	2.44	2.76	4.15	4.40
1128	CAPG_HUMAN	0.71	0.70	0.53	0.81	0.67	0.64	0.84	0.92	0.70	1.02	1.06	1.15	1.25	0.97	1.39	1.21	1.39
495366	CAPG_HUMAN	0.81	0.67	0.51	0.88	0.71	0.70	0.67	0.98	0.59	1.00	1.17	1.09	0.97	1.05	1.70	1.37	1.35
496907	CAPG_HUMAN	0.68	0.35	0.41	0.74	0.56	0.55	0.58	0.66	0.41	0.90	1.11	0.98	0.95	1.44	2.09	1.22	1.11
528317	CAPG_HUMAN	0.80	0.64	0.47	0.86	0.70	0.64	0.69	0.81	0.51	1.16	1.39	1.25	1.19	1.27	1.74	1.60	1.51
477	CAPR1_HUMAN	0.78	0.80	0.58	0.84	0.83	0.65	0.74	0.89	0.77	0.85	0.78	0.92	0.75	0.89	0.88	0.96	1.06
1351	CAPZB_HUMAN	0.77	0.81	0.59	0.78	0.78	0.63	0.81	0.79	0.74	0.98	0.90	1.00	0.95	0.93	0.88	1.04	1.13
1440	CAPZB_HUMAN	0.78	0.95	0.77	0.87	0.75	0.68	0.81	0.88	0.62	1.02	1.10	1.15	1.15	0.81	1.01	0.90	0.88
17069	CAPZB_HUMAN	0.81	0.65	0.41	0.78	0.64	0.58	0.81	0.71	0.63	1.27	1.17	1.22	1.16	1.26	1.27	1.83	1.81

1512	CATB_HUMAN	1.13	1.30	1.99	1.00	1.26	1.56	1.03	1.08	1.14	0.90	0.97	0.80	0.88	0.88	0.80	0.71	0.55
1530	CATB_HUMAN	0.67	0.78	0.58	0.78	0.76	0.66	0.67	0.70	0.59	0.99	1.06	1.14	1.05	0.96	0.95	1.13	1.07
3270147	CATB_HUMAN	0.99	1.17	1.28	1.37	1.12	1.59	0.90	1.29	0.88	1.13	1.69	1.56	1.53	1.09	1.87	1.41	1.17
152055	CATD_HUMAN	0.81	0.99	0.93	1.02	0.79	0.82	1.10	0.96	0.71	1.47	1.67	1.86	2.26	1.18	1.62	1.30	1.27
17216	CAZA1_HUMAN	0.44	0.59	0.27	0.50	0.55	0.36	0.41	0.55	0.32	1.11	1.16	1.25	1.08	1.03	1.09	1.51	1.42
1296	CAZA2_HUMAN	0.70	0.84	0.67	0.82	0.76	0.58	0.66	0.89	0.61	1.01	1.09	1.18	1.01	0.91	1.15	0.87	0.98
693	CBR4_HUMAN	1.19	1.07	1.41	1.75	2.21	2.37	1.58	1.13	1.79	0.70	0.94	1.04	1.25	1.45	0.99	1.18	1.20
163898	CBX1_HUMAN	0.86	0.85	0.70	0.92	0.76	0.71	0.74	0.81	0.64	0.87	0.89	0.92	0.76	0.78	0.85	0.88	0.82
1618	CBX3_HUMAN	0.34	0.65	0.14	0.03	0.73	0.36	4.54	0.73	0.17	0.92	1.15	0.08	15.15	1.02	1.28	2.46	1.43
1530	CBX5_HUMAN	0.67	0.78	0.58	0.78	0.76	0.66	0.67	0.70	0.59	0.99	1.06	1.14	1.05	0.96	0.95	1.13	1.07
3270147	CBX5_HUMAN	0.99	1.17	1.28	1.37	1.12	1.59	0.90	1.29	0.88	1.13	1.69	1.56	1.53	1.09	1.87	1.41	1.17
941	CD2B2_HUMAN	0.91	0.93	0.69	0.92	0.64	0.50	0.74	0.92	0.56	1.13	0.99	1.15	0.80	0.77	0.97	0.82	0.80
958	CDC37_HUMAN	0.81	0.84	0.66	0.90	0.80	0.71	0.77	0.93	0.73	0.94	0.93	1.05	0.87	0.89	1.02	1.01	1.03
1399	CDV3_HUMAN	0.87	0.86	0.76	0.86	0.69	0.65	0.78	0.78	0.58	1.21	1.29	1.21	1.16	0.97	1.17	1.03	0.98
826	CH60_HUMAN	1.30	1.69	1.35	1.14	1.14	1.17	0.80	1.16	0.91	0.95	1.09	0.83	0.68	0.64	0.75	0.82	0.73
827	CH60_HUMAN	1.63	1.71	1.46	1.41	1.17	1.26	1.08	1.38	1.14	1.00	1.07	0.86	0.71	0.68	0.86	0.86	0.83
2212082	CHP1_HUMAN	0.64	0.65	0.15	0.84	0.70	0.64	0.64	0.71	0.43	0.94	1.03	1.23	1.03	1.02	1.14	4.04	2.98
152055	CLIC1_HUMAN	0.81	0.99	0.93	1.02	0.79	0.82	1.10	0.96	0.71	1.47	1.67	1.86	2.26	1.18	1.62	1.30	1.27
1409	CLIC4_HUMAN	0.81	0.77	0.64	0.79	0.74	0.61	0.80	0.84	0.68	1.06	1.05	1.04	1.04	1.02	1.15	1.03	1.13
1478	CLIC4_HUMAN	1.73	1.93	2.47	1.35	1.19	1.11	0.97	1.08	0.90	1.13	1.31	0.89	0.74	0.69	0.73	0.51	0.48
909	CNDP2_HUMAN	0.66	0.65	0.49	0.70	0.59	0.48	0.74	0.76	0.56	1.02	0.96	1.08	1.08	0.93	1.13	1.00	1.10
918	CNDP2_HUMAN	0.63	0.66	0.51	0.79	0.69	0.61	0.77	0.86	0.62	0.95	0.96	1.19	1.19	0.98	1.25	1.13	1.18
923	CNDP2_HUMAN	0.69	0.76	0.56	0.79	0.57	0.51	0.72	0.86	0.51	1.03	1.16	1.17	1.21	0.77	1.30	0.95	1.06
447263	CNDP2_HUMAN	0.69	0.79	0.65	0.64	0.60	0.51	0.84	0.84	0.68	1.13	1.01	1.05	1.23	0.87	1.08	0.88	1.05
1154	CNN3_HUMAN	0.87	0.79	0.52	1.06	0.89	0.78	0.93	1.04	0.76	0.84	0.87	1.02	0.93	0.95	1.15	1.24	1.26
1165	CNN3_HUMAN	0.87	0.79	0.64	0.83	0.76	0.63	0.80	0.93	0.81	0.93	0.80	0.88	0.74	0.90	0.95	0.92	1.02
1641	COF1_HUMAN	0.59	0.69	0.55	0.60	0.60	0.42	0.63	0.67	0.45	0.95	0.96	0.96	1.03	0.82	0.93	0.72	0.80
693	COR1B_HUMAN	1.19	1.07	1.41	1.75	2.21	2.37	1.58	1.13	1.79	0.70	0.94	1.04	1.25	1.45	0.99	1.18	1.20
235608	COR1B_HUMAN	0.90	1.01	1.02	0.84	0.82	1.09	0.65	0.79	0.75	1.10	1.29	1.03	0.94	0.90	1.02	1.18	0.96
384077	COR1B_HUMAN	0.79	0.83	0.70	0.86	0.90	0.95	0.65	0.87	0.82	0.88	1.06	0.96	0.87	0.94	1.11	1.19	1.23
1821	COX5A_HUMAN	1.39	1.50	1.69	1.19	0.86	0.97	1.75	1.21	2.08	1.42	1.08	1.22	1.36	0.81	0.87	0.82	1.33
1167	CPIN1_HUMAN	0.82	0.77	0.61	0.92	0.80	0.70	0.82	0.92	0.75	0.90	0.91	1.01	0.91	0.93	1.08	1.03	1.12
791	CPNE1_HUMAN	0.73	0.74	0.59	0.79	0.73	0.71	0.71	0.83	0.66	0.96	1.01	1.04	0.97	0.94	1.13	1.17	1.13
384077	CPNE1_HUMAN	0.79	0.83	0.70	0.86	0.90	0.95	0.65	0.87	0.82	0.88	1.06	0.96	0.87	0.94	1.11	1.19	1.23
813	CPNE2_HUMAN	0.99	1.01	1.27	1.02	1.01	1.22	0.97	1.15	0.92	0.89	0.87	0.91	0.85	0.89	0.99	0.86	0.64
1401	CPNS1_HUMAN	0.75	0.93	0.67	0.84	0.82	0.62	0.71	0.81	0.62	0.94	0.97	1.06	0.93	0.83	0.85	0.87	0.91
1502	CRK_HUMAN	1.16	1.53	1.86	0.97	1.49	1.48	1.21	1.23	1.41	0.77	0.64	0.65	0.67	0.76	0.52	0.61	0.49
565	CSDE1_HUMAN	0.57	0.18	0.03	0.74	0.69	0.49	0.84	0.85	0.55	0.76	0.60	0.99	0.89	2.94	2.87	13.70	12.04
1282	CSN6_HUMAN	0.60	0.69	0.41	0.63	0.59	0.48	0.61	0.78	0.50	1.08	1.06	1.13	1.07	0.92	1.19	1.27	1.30
17216	CSN6_HUMAN	0.44	0.59	0.27	0.50	0.55	0.36	0.41	0.55	0.32	1.11	1.16	1.25	1.08	1.03	1.09	1.51	1.42
26959	CTNA1_HUMAN	0.51	0.07	0.01	0.53	0.36	0.28	0.34	0.65	0.37	0.87	0.92	0.91	0.61	4.53	8.61	°	°
697	CWC27_HUMAN	1.60	1.44	1.82	1.63	2.22	2.57	1.55	1.08	1.80	0.89	1.11	0.91	1.08	1.37	0.84	1.26	1.11
650	DC1I2_HUMAN	0.57	0.56	0.48	0.69	0.56	0.52	0.65	0.74	0.55	0.92	0.93	1.10	1.04	0.93	1.23	1.02	1.07
586647	DC1L2_HUMAN	0.81	0.42	0.28	0.82	0.79	0.68	0.88	0.80	0.76	0.97	0.88	0.98	0.96	1.84	1.71	2.32	2.37
3661699	DCPS_HUMAN	0.70	0.89	0.69	0.88	0.81	0.70	0.72	0.82	0.59	0.81	0.90	1.01	0.92	0.73	0.83	0.81	0.76
380472	DCTN1_HUMAN	2.49	3.06	4.41	3.01	3.81	6.92	4.13	4.96	6.78	0.60	0.48	0.73	0.80	0.75	0.78	0.95	0.74
958	DCTN2_HUMAN	0.81	0.84	0.66	0.90	0.80	0.71	0.77	0.93	0.73	0.94	0.93	1.05	0.87	0.89	1.02	1.01	1.03
3348523	DCUP_HUMAN	0.84	0.76	0.50	1.05	0.86	0.84	0.83	1.14	0.67	0.80	1.03	1.00	1.02	0.90	1.53	1.34	1.37
893	DD19A_HUMAN	0.84	0.38	0.14	0.86	0.85	0.72	0.85	0.87	0.83	0.92	0.89	0.95	0.90	2.06	2.01	4.73	5.26
902	DD19A_HUMAN	0.74	0.31	0.23	0.73	0.68	0.53	0.73	0.77	0.56	0.94	0.94	0.93	0.92	2.03	2.33	2.22	2.34
436	DDB1_HUMAN	0.72	0.66	0.61	0.78	0.67	0.65	0.79	0.86	0.69	0.99	0.88	1.07	0.97	1.00	1.14	1.05	1.00
1087	DJB11_HUMAN	1.02	0.60	0.22	1.06	1.02	0.92	0.94	1.01	0.84	0.95	1.00	0.98	0.91	1.61	1.68	4.04	3.86
1137	DJB11_HUMAN	0.70	0.28	0.84	0.82	0.73	0.61	0.70	0.78	0.58	0.96	1.10	1.13	1.10	2.46	3.02	0.70	0.75
983	DNJA2_HUMAN	0.83	0.84	0.65	0.79	0.79	0.68	0.74	0.88	0.69	0.95	0.94	0.91	0.84	0.88	0.98	0.99	0.99
1319	DNJC9_HUMAN	0.61	0.76	0.53	0.83	0.75	0.71	0.82	0.86	0.68	0.86	0.89	1.17	1.19	0.85	1.01	1.15	1.14
559	DP13A_HUMAN	0.67	0.85	0.59	0.76	0.67	0.54	0.87	0.92	0.75	0.99	0.73	1.13	0.94	0.78	0.79	0.91	0.92
122740	DP13B_HUMAN	1.17	2.37	4.00	1.20	1.05	1.58	0.74	1.33	1.36	0.69	1.27	0.70	0.80	0.31	0.71	0.27	0.43
410	DPOD2_HUMAN	0.43	0.36	0.20	0.59	0.46	0.31	0.49	0.62	0.39	0.95	1.03	1.31	1.18	1.20	1.75	1.44	1.97

13335	DPOD2_HUMAN	0.66	0.69	0.40	0.69	0.71	0.50	0.68	0.65	0.46	1.22	1.35	1.28	1.40	1.26	1.26	1.55	1.57
244116	DPOD2_HUMAN	0.36	0.20	0.02	0.42	0.29	0.07	0.36	0.44	0.17	1.11	1.16	1.32	1.17	1.60	2.52	3.48	8.63
624	DPYL2_HUMAN	0.89	0.27	0.01	0.84	0.76	0.70	0.72	0.87	0.69	0.99	1.08	0.94	0.88	2.83	3.50	°	°
644	DPYL2_HUMAN	0.92	1.40	2.13	0.84	0.91	0.80	0.64	0.89	0.86	0.83	0.92	0.75	0.64	0.54	0.59	0.31	0.37
778	DPYL2_HUMAN	0.79	0.39	0.07	0.83	0.92	0.74	0.82	0.92	0.85	0.89	0.83	0.93	0.87	2.11	1.96	8.99	9.57
35820	DPYL2_HUMAN	1.65	0.75	0.11	1.55	2.35	3.28	1.23	1.44	1.53	0.80	1.18	0.75	0.88	2.53	2.28	23.27	15.97
435	DREB_HUMAN	0.70	0.67	0.48	0.85	0.79	0.67	0.74	0.91	0.69	0.86	0.85	1.05	0.90	1.01	1.16	1.20	1.21
943	DX39A_HUMAN	0.80	0.70	0.50	0.82	0.67	0.62	0.75	0.87	0.59	1.05	1.00	1.07	0.93	0.99	1.23	1.29	1.18
1352	ECH1_HUMAN	0.81	0.73	0.63	0.79	0.72	0.72	0.77	0.74	0.52	1.17	1.36	1.15	1.29	1.16	1.38	1.32	1.10
1435	ECHM_HUMAN	0.77	0.69	0.50	0.79	0.70	0.62	0.83	0.85	0.56	1.05	1.07	1.08	1.15	1.06	1.32	1.30	1.20
1453	ECHM_HUMAN	0.87	0.61	0.16	0.92	0.85	0.82	0.81	0.92	0.77	0.90	0.91	0.95	0.85	1.25	1.36	4.72	4.44
1473	ECHM_HUMAN	1.07	0.93	0.47	1.17	0.97	1.06	0.98	1.13	0.91	0.95	1.02	1.04	0.93	0.99	1.23	2.17	1.99
1225	EF1D_HUMAN	1.04	0.95	0.86	1.10	1.15	0.96	1.23	1.66	1.59	0.71	0.49	0.75	0.57	0.85	0.85	0.79	0.90
1251	EF1D_HUMAN	1.08	1.10	0.96	1.14	1.10	0.88	1.21	1.22	1.27	0.70	0.50	0.74	0.56	0.70	0.56	0.64	0.66
1055	EIF3G_HUMAN	0.83	0.99	0.99	1.03	1.30	1.31	1.33	1.13	1.28	0.63	0.54	0.79	0.87	0.84	0.62	0.84	0.70
1058	EIF3G_HUMAN	0.51	0.62	0.46	0.69	0.57	0.47	0.74	0.84	0.55	0.92	0.84	1.23	1.21	0.84	1.14	0.93	1.00
1078	EIF3G_HUMAN	0.69	0.77	0.57	0.84	0.78	0.64	0.69	0.80	0.76	0.87	0.83	1.06	0.83	0.88	0.86	0.97	1.11
1913428	ELOB_HUMAN	0.48	0.28	0.15	0.94	0.52	0.26	0.48	0.66	0.30	1.64	2.23	3.22	2.26	3.09	5.37	2.86	4.52
1015	ENOA_HUMAN	1.92	2.91	3.48	1.66	2.54	3.38	1.86	2.29	2.52	0.92	0.92	0.80	0.90	0.80	0.73	0.90	0.67
953	ENOG_HUMAN	0.76	0.81	0.54	0.86	0.75	0.64	0.74	0.85	0.62	0.92	0.87	1.04	0.86	0.84	0.91	1.09	1.00
1001	ENOG_HUMAN	0.79	0.68	0.55	0.85	0.66	0.64	0.82	0.83	0.63	1.06	1.10	1.15	1.15	1.04	1.36	1.22	1.25
15652	ENOG_HUMAN	0.99	0.84	0.68	0.92	0.69	0.70	0.83	0.87	0.66	1.04	1.07	0.97	0.90	0.85	1.10	1.07	1.05
93790	ENOG_HUMAN	0.60	0.57	0.44	0.76	0.61	0.44	0.71	0.67	0.51	0.86	0.74	1.07	0.87	0.93	0.87	0.86	0.85
132924	ENPL_HUMAN	0.75	0.86	0.76	0.90	0.64	0.71	0.73	0.89	0.67	0.92	0.97	1.10	0.94	0.68	1.00	0.86	0.86
274688	ENPL_HUMAN	1.37	1.52	1.85	1.36	1.06	1.09	0.77	1.09	0.83	0.90	1.11	0.89	0.62	0.62	0.79	0.53	0.49
622	ERF3A_HUMAN	0.76	0.78	0.51	0.82	0.80	0.64	0.74	0.89	0.79	0.88	0.80	0.95	0.77	0.89	0.90	1.10	1.23
622	ERF3B_HUMAN	0.76	0.78	0.51	0.82	0.80	0.64	0.74	0.89	0.79	0.88	0.80	0.95	0.77	0.89	0.90	1.10	1.23
518	ERO1A_HUMAN	0.69	0.64	0.47	0.71	0.66	0.53	0.69	0.80	0.69	0.98	0.85	1.00	0.85	1.01	1.06	1.11	1.25
1408	ERP29_HUMAN	0.92	0.56	0.25	0.95	0.84	0.75	0.86	0.93	0.67	1.01	1.05	1.05	0.98	1.53	1.74	3.07	2.84
1433	ERP29_HUMAN	1.04	1.28	1.52	1.13	0.98	0.96	0.86	0.97	0.81	0.99	1.13	1.07	0.93	0.76	0.86	0.62	0.60
2298466	ERP29_HUMAN	0.26	0.42	0.40	0.91	1.81	0.57	0.99	1.13	6.17	0.58	0.22	2.01	0.84	2.48	0.59	0.82	3.39
1458	EXOS4_HUMAN	0.84	0.89	0.77	0.85	0.66	0.63	0.81	0.81	0.59	1.19	1.22	1.20	1.18	0.88	1.11	0.96	0.93
624	EZRI_HUMAN	0.89	0.27	0.01	0.84	0.76	0.70	0.72	0.87	0.69	0.99	1.08	0.94	0.88	2.83	3.50	°	°
637	EZRI_HUMAN	0.71	0.89	1.04	0.80	0.83	0.73	0.87	0.87	0.87	0.90	0.77	1.01	0.95	0.84	0.76	0.63	0.65
644	EZRI_HUMAN	0.92	1.40	2.13	0.84	0.91	0.80	0.64	0.89	0.86	0.83	0.92	0.75	0.64	0.54	0.59	0.31	0.37
627	FETA_HUMAN	1.14	1.40	1.56	1.21	1.43	1.87	1.07	1.15	1.33	0.82	0.98	0.87	0.91	0.84	0.80	0.99	0.83
191574	FKB10_HUMAN	0.91	1.09	0.76	0.94	0.78	0.67	0.68	0.81	0.73	1.14	1.21	1.18	0.90	0.81	0.90	1.00	1.17
387435	FKB10_HUMAN	0.95	0.90	0.65	0.96	0.72	0.69	0.53	0.74	0.47	0.97	1.50	0.97	0.84	0.77	1.23	1.02	1.10
843	FKBP4_HUMAN	0.81	0.80	0.66	0.82	0.74	0.62	0.75	0.86	0.72	0.96	0.88	0.97	0.81	0.89	0.94	0.90	0.97
692	FKBP9_HUMAN	1.02	0.83	0.94	0.95	0.86	0.90	0.54	0.83	0.39	0.81	1.52	0.75	0.81	0.84	1.53	0.77	0.64
1173	FSTL1_HUMAN	0.80	0.90	0.93	0.72	0.72	0.64	0.59	0.73	0.63	0.94	0.97	0.84	0.70	0.75	0.78	0.64	0.65
1137	GALK1_HUMAN	0.70	0.28	0.84	0.82	0.73	0.61	0.70	0.78	0.58	0.96	1.10	1.13	1.10	2.46	3.02	0.70	0.75
496	GANAB_HUMAN	0.68	0.75	0.71	0.72	0.63	0.57	0.62	0.75	0.48	1.05	1.21	1.11	1.11	0.88	1.21	0.85	0.82
499	GANAB_HUMAN	0.74	0.71	0.69	0.80	0.62	0.60	0.63	0.78	0.49	1.09	1.32	1.18	1.12	0.96	1.47	0.95	0.94
508	GANAB_HUMAN	0.66	0.57	0.48	0.70	0.58	0.54	0.68	0.75	0.52	1.06	1.03	1.11	1.05	1.09	1.36	1.18	1.11
510	GANAB_HUMAN	0.57	0.54	0.42	0.68	0.58	0.52	0.55	0.72	0.45	1.00	1.26	1.19	1.19	1.08	1.69	1.25	1.35
4596	GANAB_HUMAN	0.67	0.74	0.49	0.73	0.63	0.54	0.54	0.76	0.56	0.93	1.06	1.01	0.87	0.79	1.09	1.02	1.23
309937	GANAB_HUMAN	0.64	0.63	0.34	0.85	0.77	0.54	0.67	0.81	0.65	0.71	0.68	0.94	0.72	0.86	0.87	1.12	1.30
460138	GANAB_HUMAN	0.76	0.51	0.24	0.74	0.58	0.48	0.59	0.76	0.47	1.02	1.16	0.99	0.90	1.15	1.73	1.99	2.24
1282	GBB1_HUMAN	0.60	0.69	0.41	0.63	0.59	0.48	0.61	0.78	0.50	1.08	1.06	1.13	1.07	0.92	1.19	1.27	1.30
1282	GBB2_HUMAN	0.60	0.69	0.41	0.63	0.59	0.48	0.61	0.78	0.50	1.08	1.06	1.13	1.07	0.92	1.19	1.27	1.30
17229	GBB2_HUMAN	0.46	0.58	0.31	0.53	0.55	0.39	0.45	0.55	0.37	1.05	1.17	1.22	1.16	0.99	1.11	1.35	1.40
825	GDIA_HUMAN	1.23	1.50	1.84	1.08	1.23	1.54	1.17	1.27	1.32	1.02	0.97	0.90	0.92	0.84	0.82	0.86	0.70
828	GDIA_HUMAN	1.77	2.23	2.89	1.42	1.70	2.40	1.66	1.96	2.07	1.02	0.92	0.82	0.87	0.78	0.81	0.85	0.66
940	GDIB_HUMAN	0.67	0.65	0.49	0.69	0.67	0.54	0.70	0.75	0.63	0.99	0.93	1.03	0.98	1.03	1.08	1.08	1.18
971	GDIB_HUMAN	0.78	0.82	0.59	0.78	0.77	0.69	0.70	0.85	0.68	0.95	0.98	0.96	0.89	0.89	1.02	1.11	1.14
1401	GDIR1_HUMAN	0.75	0.93	0.67	0.84	0.82	0.62	0.71	0.81	0.62	0.94	0.97	1.06	0.93	0.83	0.85	0.87	0.91
584	GELS_HUMAN	0.63	0.66	0.40	0.76	0.61	0.69	0.67	0.86	0.60	1.06	1.15	1.29	1.23	0.99	1.51	1.83	1.73

585	GELS_HUMAN	0.47	0.51	0.38	0.70	0.56	0.58	0.75	0.85	0.62	0.99	0.93	1.48	1.48	1.08	1.56	1.52	1.52
17482	GELS_HUMAN	1.04	1.42	1.75	0.91	1.10	1.48	0.66	0.85	0.67	1.31	2.06	1.16	1.31	1.02	1.24	1.11	0.79
1166	GIPC1_HUMAN	0.91	0.51	0.30	1.01	0.95	0.86	1.00	1.04	0.95	0.89	0.89	0.98	0.98	1.66	1.82	2.59	2.88
1167	GLRX3_HUMAN	0.82	0.77	0.61	0.92	0.80	0.70	0.82	0.92	0.75	0.90	0.91	1.01	0.91	0.93	1.08	1.03	1.12
1167	GLRX3_HUMAN	0.82	0.77	0.61	0.92	0.80	0.70	0.82	0.92	0.75	0.90	0.91	1.01	0.91	0.93	1.08	1.03	1.12
535	GLU2B_HUMAN	1.02	1.20	1.36	1.07	0.86	0.84	0.84	1.02	0.72	1.06	1.12	1.11	0.92	0.76	0.95	0.65	0.59
3296127	GMFB_HUMAN	0.76	0.89	0.61	1.22	0.89	0.98	1.12	1.31	1.07	0.93	1.11	1.48	1.63	0.94	1.65	1.49	1.94
309937	GRAP1_HUMAN	0.64	0.63	0.34	0.85	0.77	0.54	0.67	0.81	0.65	0.71	0.68	0.94	0.72	0.86	0.87	1.12	1.30
22159	GRP75_HUMAN	1.47	2.16	2.40	1.47	1.91	2.97	1.38	1.80	2.07	0.81	0.96	0.81	0.90	0.72	0.80	1.00	0.83
318	GRP78_HUMAN	0.45	0.33	0.19	0.04	0.05	0.05	0.09	0.12	0.56	8.50	1.24	0.84	0.26	1.38	0.46	2.28	3.66
658	GRP78_HUMAN	1.04	1.08	1.41	1.10	1.00	0.94	1.11	0.83	1.01	0.99	0.89	1.05	0.95	0.92	0.69	0.66	0.64
661	GRP78_HUMAN	1.69	2.06	2.89	1.55	1.39	1.74	1.19	1.79	1.64	0.89	0.97	0.82	0.68	0.60	0.84	0.54	0.55
662	GRP78_HUMAN	1.32	1.42	1.91	1.26	1.08	1.23	1.10	1.26	1.26	0.95	0.92	0.91	0.77	0.72	0.82	0.61	0.60
3760	GRP78_HUMAN	2.45	3.63	5.67	2.16	2.03	2.84	1.47	3.16	2.74	0.84	1.07	0.74	0.64	0.47	0.93	0.42	0.52
10719	GRP78_HUMAN	1.27	1.46	2.18	0.98	0.90	0.88	1.57	1.80	1.31	1.66	0.94	1.28	1.16	1.02	1.16	0.67	0.57
12067	GRP78_HUMAN	1.17	1.04	1.03	1.06	1.00	1.08	0.66	1.11	0.61	0.79	1.17	0.72	0.66	0.76	1.24	0.83	0.69
120878	GRP78_HUMAN	0.95	0.99	0.83	1.10	0.89	0.91	0.79	0.95	0.58	0.83	1.12	0.96	0.93	0.75	1.08	0.91	0.78
366771	GRP78_HUMAN	1.21	0.94	0.60	0.70	0.61	0.59	0.35	0.49	0.23	1.24	2.61	0.71	0.74	0.80	1.37	1.21	0.99
2845794	GRP78_HUMAN	1.39	1.40	1.49	1.09	1.03	0.97	1.00	1.02	0.86	0.99	1.10	0.78	0.79	0.73	0.80	0.65	0.63
2846419	GRP78_HUMAN	0.92	0.94	1.04	0.98	0.75	0.72	0.76	0.92	0.56	1.17	1.38	1.25	1.15	0.94	1.36	0.82	0.75
943	GSHB_HUMAN	0.80	0.70	0.50	0.82	0.67	0.62	0.75	0.87	0.59	1.05	1.00	1.07	0.93	0.99	1.23	1.29	1.18
1393	GSTO1_HUMAN	0.90	0.28	0.01	0.96	0.90	0.76	0.98	0.97	0.84	1.04	1.04	1.11	1.13	3.41	3.67	°	°
1408	GSTO1_HUMAN	0.92	0.56	0.25	0.95	0.84	0.75	0.86	0.93	0.67	1.01	1.05	1.05	0.98	1.53	1.74	3.07	2.84
422947	GSTO1_HUMAN	0.77	0.88	0.63	0.78	0.88	0.61	0.77	0.75	0.70	0.79	0.75	0.80	0.75	0.79	0.63	0.77	0.83
1541	GSTP1_HUMAN	0.71	0.77	0.59	0.85	0.71	0.65	0.74	0.84	0.60	1.04	1.14	1.24	1.19	0.95	1.25	1.14	1.17
843	HDAC2_HUMAN	0.81	0.80	0.66	0.82	0.74	0.62	0.75	0.86	0.72	0.96	0.88	0.97	0.81	0.89	0.94	0.90	0.97
1194	HDGF_HUMAN	0.76	0.76	0.55	0.86	0.70	0.67	0.70	0.85	0.59	1.02	1.14	1.15	1.05	0.94	1.28	1.25	1.24
1231	HDGF_HUMAN	0.91	0.66	0.56	1.04	0.83	0.84	0.98	1.00	0.88	0.80	0.77	0.91	0.83	1.00	1.16	1.18	1.20
88373	HDGF_HUMAN	0.72	0.67	0.47	0.86	0.69	0.67	0.71	0.80	0.56	0.92	1.05	1.10	1.03	0.95	1.25	1.32	1.24
306795	HDGF_HUMAN	0.66	0.56	0.21	0.56	0.50	0.46	0.67	0.55	0.59	1.23	0.77	1.04	0.78	1.09	0.75	2.68	2.15
2906459	HDGF_HUMAN	0.81	0.73	0.45	0.96	0.80	0.73	0.78	0.90	0.69	0.87	0.88	1.03	0.85	0.95	1.08	1.40	1.34
217409	HEXB_HUMAN	0.97	0.97	0.92	0.92	0.81	0.71	0.89	0.83	0.66	1.07	1.16	1.02	1.07	0.89	0.99	0.82	0.84
879	HMCS1_HUMAN	0.81	0.79	0.60	0.75	0.67	0.55	0.51	0.65	0.53	1.01	1.18	0.93	0.75	0.86	0.96	0.93	1.04
191012	HMCS1_HUMAN	0.89	0.92	0.71	0.90	0.78	0.69	0.75	0.93	0.64	0.96	1.03	0.98	0.88	0.82	1.04	0.93	0.93
464	HNRH1_HUMAN	0.87	0.66	0.70	0.73	0.66	0.88	0.67	0.79	0.75	1.08	1.21	0.90	0.94	1.07	1.44	1.35	1.29
909	HNRH1_HUMAN	0.66	0.65	0.49	0.70	0.59	0.48	0.74	0.76	0.56	1.02	0.96	1.08	1.08	0.93	1.13	1.00	1.10
918	HNRH1_HUMAN	0.63	0.66	0.51	0.79	0.69	0.61	0.77	0.86	0.62	0.95	0.96	1.19	1.19	0.98	1.25	1.13	1.18
921	HNRH1_HUMAN	0.67	0.66	0.54	0.72	0.61	0.54	0.75	0.79	0.60	1.05	1.05	1.12	1.16	0.96	1.24	1.05	1.18
923	HNRH1_HUMAN	0.69	0.76	0.56	0.79	0.57	0.51	0.72	0.86	0.51	1.03	1.16	1.17	1.21	0.77	1.30	0.95	1.06
934	HNRH1_HUMAN	0.77	0.32	0.02	0.81	0.71	0.61	0.70	0.81	0.61	0.97	1.10	1.02	1.00	2.19	2.79	68.19	76.90
956	HNRH1_HUMAN	0.68	0.70	0.50	0.78	0.71	0.60	0.80	0.84	0.67	0.98	0.97	1.13	1.13	0.99	1.16	1.18	1.32
971	HNRH1_HUMAN	0.78	0.82	0.59	0.78	0.77	0.69	0.70	0.85	0.68	0.95	0.98	0.96	0.89	0.89	1.02	1.11	1.14
29521	HNRH1_HUMAN	0.66	0.65	0.49	0.76	0.68	0.60	0.74	0.83	0.60	0.93	0.97	1.07	1.10	0.97	1.23	1.15	1.20
447263	HNRH1_HUMAN	0.69	0.79	0.65	0.64	0.60	0.51	0.84	0.84	0.68	1.13	1.01	1.05	1.23	0.87	1.08	0.88	1.05
471051	HNRH1_HUMAN	0.69	0.32	0.20	0.79	0.75	0.64	0.78	0.81	0.66	0.88	0.91	1.01	1.02	2.04	2.29	2.81	2.98
922	HNRH2_HUMAN	0.67	0.40	0.20	0.71	0.69	0.58	0.70	0.75	0.61	0.90	0.95	0.96	0.99	1.57	1.78	2.65	2.94
922	HNRH2_HUMAN	0.67	0.40	0.20	0.71	0.69	0.58	0.70	0.75	0.61	0.90	0.95	0.96	0.99	1.57	1.78	2.65	2.94
29521	HNRH2_HUMAN	0.66	0.65	0.49	0.76	0.68	0.60	0.74	0.83	0.60	0.93	0.97	1.07	1.10	0.97	1.23	1.15	1.20
441	HNRL2_HUMAN	0.58	0.54	0.35	0.63	0.52	0.48	0.60	0.71	0.48	1.05	0.99	1.13	1.02	1.01	1.30	1.45	1.36
457	HNRL2_HUMAN	0.57	0.52	0.31	0.59	0.45	0.45	0.55	0.66	0.45	1.18	1.23	1.23	1.19	1.02	1.54	1.73	1.77
458	HNRL2_HUMAN	0.61	0.53	0.34	0.65	0.54	0.50	0.60	0.73	0.50	1.06	1.06	1.14	1.05	1.08	1.48	1.59	1.58
494	HNRL2_HUMAN	0.70	0.49	0.35	0.83	0.76	0.65	0.87	0.99	0.80	0.87	0.71	1.02	0.88	1.34	1.43	1.64	1.65
1203	HNRPC_HUMAN	0.75	0.63	0.71	0.90	0.86	0.73	0.79	0.85	0.66	0.76	1.01	0.91	1.07	1.03	1.36	0.77	0.94
305540	HNRPC_HUMAN	0.83	1.00	1.21	0.70	0.76	0.62	1.44	1.51	1.35	0.94	0.53	0.80	0.92	0.71	0.80	0.49	0.60
2964442	HNRPC_HUMAN	0.74	0.70	0.60	0.73	1.00	0.76	1.28	1.22	1.36	0.80	0.50	0.79	0.86	1.14	0.87	1.00	1.12
854	HNRPF_HUMAN	1.12	1.30	1.58	1.12	0.90	0.94	0.85	1.04	0.78	1.06	1.24	1.05	0.94	0.73	1.00	0.63	0.61
977	HNRPF_HUMAN	0.91	1.04	1.15	1.02	0.84	0.72	0.87	0.96	0.65	0.97	1.05	1.08	1.00	0.78	0.97	0.61	0.59
13616	HNRPF_HUMAN	0.89	0.93	0.76	0.94	0.75	0.72	0.80	0.90	0.65	0.90	0.93	0.94	0.84	0.72	0.90	0.85	0.80

770	HNRPK_HUMAN	2.31	2.77	3.79	1.91	2.64	3.85	2.34	2.47	2.85	0.89	0.86	0.74	0.87	0.85	0.76	0.90	0.64
796	HNRPK_HUMAN	0.84	0.71	0.64	0.94	0.70	0.73	0.96	0.96	0.69	0.98	0.99	1.11	1.14	0.97	1.34	1.13	1.08
879	HNRPK_HUMAN	0.81	0.79	0.60	0.75	0.67	0.55	0.51	0.65	0.53	1.01	1.18	0.93	0.75	0.86	0.96	0.93	1.04
191012	HNRPK_HUMAN	0.89	0.92	0.71	0.90	0.78	0.69	0.75	0.93	0.64	0.96	1.03	0.98	0.88	0.82	1.04	0.93	0.93
201269	HNRPK_HUMAN	0.63	0.56	0.55	0.55	0.58	0.41	0.49	0.70	0.48	1.01	0.95	0.88	0.73	1.05	1.19	0.76	0.84
446	HNRPU_HUMAN	0.40	0.61	0.36	0.72	0.45	0.38	0.59	0.76	0.38	1.00	1.12	1.80	1.66	0.73	1.40	1.05	1.21
447	HNRPU_HUMAN	0.83	0.70	0.42	0.80	0.68	0.62	0.57	0.74	0.50	1.01	1.23	0.97	0.85	0.98	1.32	1.49	1.46
464	HNRPU_HUMAN	0.87	0.66	0.70	0.73	0.66	0.88	0.67	0.79	0.75	1.08	1.21	0.90	0.94	1.07	1.44	1.35	1.29
479	HNRPU_HUMAN	0.47	0.30	0.20	0.65	0.41	0.45	0.73	0.73	0.62	0.99	0.79	1.37	1.23	1.35	1.91	2.27	2.49
10099	HOOK3_HUMAN	1.11	1.27	1.31	1.61	1.59	1.37	1.14	1.20	1.26	1.03	1.36	1.49	1.40	1.29	1.29	1.08	1.31
477	HS105_HUMAN	0.78	0.80	0.58	0.84	0.83	0.65	0.74	0.89	0.77	0.85	0.78	0.92	0.75	0.89	0.88	0.96	1.06
486	HS105_HUMAN	0.79	0.78	0.54	0.87	0.84	0.67	0.80	0.94	0.83	0.85	0.75	0.93	0.76	0.92	0.91	1.06	1.16
507	HS105_HUMAN	0.64	0.62	0.44	0.67	0.62	0.48	0.73	0.79	0.68	1.01	0.80	1.06	0.92	1.01	1.04	1.12	1.25
553	HS90A_HUMAN	0.95	0.79	0.87	0.98	1.09	0.97	1.18	0.89	1.14	1.15	0.94	1.18	1.16	1.59	1.06	1.29	1.23
559	HS90A_HUMAN	0.67	0.85	0.59	0.76	0.67	0.54	0.87	0.92	0.75	0.99	0.73	1.13	0.94	0.78	0.79	0.91	0.92
571	HS90A_HUMAN	0.76	0.92	0.60	0.79	0.80	0.58	0.88	0.95	0.75	0.98	0.66	1.02	0.76	0.85	0.69	0.94	0.83
573	HS90A_HUMAN	0.74	0.88	0.62	0.86	0.90	0.65	0.92	0.97	0.94	0.77	0.60	0.89	0.74	0.78	0.66	0.81	0.91
574	HS90A_HUMAN	0.88	0.96	0.68	0.90	0.90	0.66	0.90	0.95	0.92	0.91	0.76	0.93	0.78	0.85	0.75	0.89	1.02
575	HS90A_HUMAN	0.82	0.93	0.65	0.86	0.82	0.61	0.83	0.97	0.85	0.92	0.76	0.96	0.77	0.81	0.78	0.87	0.99
584	HS90A_HUMAN	0.63	0.66	0.40	0.76	0.61	0.69	0.67	0.86	0.60	1.06	1.15	1.29	1.23	0.99	1.51	1.83	1.73
618	HS90A_HUMAN	0.73	0.66	0.53	0.75	0.63	0.62	0.68	0.76	0.57	1.05	1.06	1.08	0.98	1.02	1.23	1.23	1.13
622	HS90A_HUMAN	0.76	0.78	0.51	0.82	0.80	0.64	0.74	0.89	0.79	0.88	0.80	0.95	0.77	0.89	0.90	1.10	1.23
623	HS90A_HUMAN	0.76	0.78	0.63	0.80	0.71	0.71	0.60	0.80	0.60	0.94	1.10	0.98	0.87	0.86	1.14	1.07	1.05
627	HS90A_HUMAN	1.14	1.40	1.56	1.21	1.43	1.87	1.07	1.15	1.33	0.82	0.98	0.87	0.91	0.84	0.80	0.99	0.83
628	HS90A_HUMAN	1.58	1.95	2.46	1.48	1.84	2.60	1.32	1.46	1.65	0.89	1.10	0.84	0.92	0.84	0.82	0.94	0.74
629	HS90A_HUMAN	0.70	0.69	0.58	0.72	0.65	0.63	0.75	0.80	0.63	1.07	0.98	1.10	1.04	1.02	1.14	1.16	1.05
4106	HS90A_HUMAN	0.96	0.72	0.97	0.92	1.01	0.88	1.33	0.83	1.29	1.36	1.00	1.30	1.40	1.91	1.15	1.22	1.33
10099	HS90A_HUMAN	1.11	1.27	1.31	1.61	1.59	1.37	1.14	1.20	1.26	1.03	1.36	1.49	1.40	1.29	1.29	1.08	1.31
10923	HS90A_HUMAN	0.65	0.77	0.58	0.81	0.67	0.55	0.72	0.91	0.75	0.85	0.76	1.05	0.84	0.73	0.89	0.81	0.97
20414	HS90A_HUMAN	1.24	1.44	1.61	1.22	1.48	1.96	1.09	1.24	1.53	0.84	0.94	0.82	0.82	0.86	0.81	1.02	0.89
363557	HS90A_HUMAN	0.77	0.78	0.91	0.83	0.81	0.54	0.54	0.82	0.48	0.80	1.25	0.86	0.87	0.83	1.32	0.47	0.66
596190	HS90A_HUMAN	1.61	2.09	1.89	1.46	0.99	1.27	0.47	1.04	0.61	1.04	2.48	0.95	0.73	0.50	1.23	0.70	0.79
574	HS90B_HUMAN	0.88	0.96	0.68	0.90	0.90	0.66	0.90	0.95	0.92	0.91	0.76	0.93	0.78	0.85	0.75	0.89	1.02
577	HS90B_HUMAN	0.90	0.85	0.61	0.88	0.72	0.69	0.88	0.93	0.69	1.06	1.04	1.04	1.01	0.90	1.14	1.20	1.18
4106	HS90B_HUMAN	0.96	0.72	0.97	0.92	1.01	0.88	1.33	0.83	1.29	1.36	1.00	1.30	1.40	1.91	1.15	1.22	1.33
490	HSP74_HUMAN	0.84	0.82	0.57	0.87	0.80	0.72	0.73	0.92	0.76	0.97	1.01	1.00	0.87	0.93	1.12	1.22	1.36
491	HSP74_HUMAN	0.72	0.71	0.49	0.80	0.75	0.63	0.81	0.86	0.81	0.93	0.81	1.03	0.91	0.98	0.98	1.20	1.32
694	HSP7C_HUMAN	1.11	1.33	1.17	1.03	0.98	1.05	0.68	0.99	0.75	0.87	1.14	0.80	0.69	0.64	0.85	0.78	0.73
92002	HSP7C_HUMAN	0.85	0.96	0.67	0.78	0.58	0.61	0.78	0.86	0.76	1.37	0.86	1.26	0.78	0.83	0.77	1.26	0.97
335202	HSP7C_HUMAN	0.68	0.44	0.28	0.77	0.63	0.60	0.39	0.75	0.35	1.09	1.99	1.22	1.14	1.54	3.38	2.32	2.46
717	HSPA8_HUMAN	1.99	2.81	3.40	1.80	2.45	3.72	1.61	2.01	2.46	0.84	1.04	0.76	0.84	0.73	0.75	0.92	0.76
22159	HSPA8_HUMAN	1.47	2.16	2.40	1.47	1.91	2.97	1.38	1.80	2.07	0.81	0.96	0.81	0.90	0.72	0.80	1.00	0.83
1453	HSPB1_HUMAN	0.87	0.61	0.16	0.92	0.85	0.82	0.81	0.92	0.77	0.90	0.91	0.95	0.85	1.25	1.36	4.72	4.44
1468	HSPB1_HUMAN	0.97	1.02	0.79	0.97	0.82	0.71	0.77	0.88	0.58	0.96	1.05	0.95	0.84	0.77	0.91	0.86	0.77
1492	HSPB1_HUMAN	0.88	0.41	0.60	0.96	0.80	0.76	0.87	1.00	0.73	0.98	1.07	1.07	1.07	1.91	2.59	1.25	1.32
364	HYOU1_HUMAN	1.08	1.19	1.38	1.03	0.90	0.86	0.85	0.97	0.78	1.01	1.06	0.96	0.84	0.77	0.87	0.63	0.60
364	HYOU1_HUMAN	1.08	1.19	1.38	1.03	0.90	0.86	0.85	0.97	0.78	1.01	1.06	0.96	0.84	0.77	0.87	0.63	0.60
365	HYOU1_HUMAN	0.89	0.96	1.02	0.92	0.83	0.71	0.85	0.92	0.73	0.96	0.90	0.99	0.86	0.83	0.87	0.68	0.65
365	HYOU1_HUMAN	0.89	0.96	1.02	0.92	0.83	0.71	0.85	0.92	0.73	0.96	0.90	0.99	0.86	0.83	0.87	0.68	0.65
376	HYOU1_HUMAN	1.31	1.48	1.90	1.16	1.01	1.07	0.85	1.03	0.85	1.02	1.21	0.90	0.79	0.70	0.84	0.58	0.54
436	HYOU1_HUMAN	0.72	0.66	0.61	0.78	0.67	0.65	0.79	0.86	0.69	0.99	0.88	1.07	0.97	1.00	1.14	1.05	1.00
5403	HYOU1_HUMAN	0.72	0.76	0.39	0.68	0.60	0.57	0.66	0.84	0.70	1.14	1.03	1.09	0.95	0.90	1.14	1.67	1.84
5526	HYOU1_HUMAN	0.68	0.62	0.34	0.69	0.60	0.54	0.70	0.81	0.66	1.11	0.93	1.14	0.97	1.07	1.22	1.74	1.81
319168	HYOU1_HUMAN	0.89	0.92	0.89	0.90	0.96	0.78	0.87	1.01	0.71	0.87	0.81	0.88	0.80	0.92	0.90	0.77	0.65
427	ICAL_HUMAN	0.75	0.73	0.56	0.93	0.70	0.72	0.77	0.91	0.64	0.99	1.05	1.23	1.08	0.96	1.32	1.28	1.22
430	ICAL_HUMAN	0.72	0.65	0.59	0.93	0.72	0.75	0.80	0.90	0.66	0.94	0.92	1.22	1.03	1.04	1.27	1.20	1.03
475	ICAL_HUMAN	0.66	0.70	0.41	0.89	0.70	0.64	0.72	0.88	0.63	0.92	1.02	1.24	1.12	0.92	1.29	1.42	1.57
126195	ICAL_HUMAN	0.81	0.80	0.66	0.91	0.71	0.73	0.73	0.87	0.61	1.05	1.21	1.18	1.08	0.94	1.32	1.16	1.13

1154	IDH3A_HUMAN	0.87	0.79	0.52	1.06	0.89	0.78	0.93	1.04	0.76	0.84	0.87	1.02	0.93	0.95	1.15	1.24	1.26
1174	IDH3A_HUMAN	0.99	0.87	0.61	1.10	0.95	0.92	0.88	1.07	0.76	0.80	0.95	0.88	0.84	0.88	1.16	1.21	1.18
1458	IDI1_HUMAN	0.84	0.89	0.77	0.85	0.66	0.63	0.81	0.81	0.59	1.19	1.22	1.20	1.18	0.88	1.11	0.96	0.93
3591281	IF1AX_HUMAN	0.68	0.95	0.47	0.98	0.79	0.89	0.43	1.36	0.58	1.20	1.31	1.75	0.84	1.00	1.87	2.28	1.61
1196	IF2A_HUMAN	0.75	0.83	0.60	0.84	0.86	0.68	0.77	0.84	0.75	0.87	0.85	0.98	0.87	0.91	0.87	0.99	1.07
964	IF2B_HUMAN	0.80	0.80	0.62	0.83	0.65	0.64	0.74	0.87	0.65	1.06	1.03	1.10	0.96	0.86	1.13	1.09	1.08
1030	IF4A1_HUMAN	0.78	0.70	0.53	0.81	0.66	0.56	0.76	0.74	0.58	1.09	1.06	1.14	1.04	1.02	1.12	1.15	1.16
3670	IF4A1_HUMAN	0.83	0.56	0.48	0.92	0.66	0.57	0.83	0.83	0.65	1.03	0.93	1.15	0.93	1.21	1.37	1.24	1.26
13655	IF4A1_HUMAN	1.00	1.00	0.72	0.87	0.80	0.70	0.61	0.90	0.58	1.04	1.34	0.90	0.82	0.83	1.20	1.00	1.08
2298466	IF4E_HUMAN	0.26	0.42	0.40	0.91	1.81	0.57	0.99	1.13	6.17	0.58	0.22	2.01	0.84	2.48	0.59	0.82	3.39
1697	IF5A1_HUMAN	0.74	0.86	0.54	0.83	0.86	0.62	0.82	0.87	0.79	0.90	0.82	1.01	0.91	0.90	0.83	1.03	1.21
1032	ILEU_HUMAN	0.66	0.32	0.01	0.68	0.75	0.54	0.69	0.75	0.67	0.98	0.92	1.01	0.95	2.29	2.15	2,240,386	2,605,427
1087	ILEU_HUMAN	1.02	0.60	0.22	1.06	1.02	0.92	0.94	1.01	0.84	0.95	1.00	0.98	0.91	1.61	1.68	4.04	3.86
1093	ILEU_HUMAN	0.70	0.38	0.06	0.79	0.65	0.63	0.86	0.83	0.63	1.02	1.01	1.16	1.25	1.74	2.18	10.97	10.76
1137	ILEU_HUMAN	0.70	0.28	0.84	0.82	0.73	0.61	0.70	0.78	0.58	0.96	1.10	1.13	1.10	2.46	3.02	0.70	0.75
1139	ILEU_HUMAN	0.80	0.42	0.15	0.90	0.78	0.79	0.75	0.84	0.65	0.93	1.13	1.04	1.05	1.70	2.23	5.03	5.04
499	IMMT_HUMAN	0.74	0.71	0.69	0.80	0.62	0.60	0.63	0.78	0.49	1.09	1.32	1.18	1.12	0.96	1.47	0.95	0.94
510	IMMT_HUMAN	0.57	0.54	0.42	0.68	0.58	0.52	0.55	0.72	0.45	1.00	1.26	1.19	1.19	1.08	1.69	1.25	1.35
577	IMMT_HUMAN	0.90	0.85	0.61	0.88	0.72	0.69	0.88	0.93	0.69	1.06	1.04	1.04	1.01	0.90	1.14	1.20	1.18
624	IMMT_HUMAN	0.89	0.27	0.01	0.84	0.76	0.70	0.72	0.87	0.69	0.99	1.08	0.94	0.88	2.83	3.50	°	°
644	IMMT_HUMAN	0.92	1.40	2.13	0.84	0.91	0.80	0.64	0.89	0.86	0.83	0.92	0.75	0.64	0.54	0.59	0.31	0.37
1351	IPYR_HUMAN	0.77	0.81	0.59	0.78	0.78	0.63	0.81	0.79	0.74	0.98	0.90	1.00	0.95	0.93	0.88	1.04	1.13
17069	IPYR_HUMAN	0.81	0.65	0.41	0.78	0.64	0.58	0.81	0.71	0.63	1.27	1.17	1.22	1.16	1.26	1.27	1.83	1.81
767	IRGQ_HUMAN	2.46	3.02	4.17	1.78	2.33	3.84	2.15	2.46	2.92	1.02	1.03	0.74	0.90	0.78	0.84	0.94	0.72
393	ITA3_HUMAN	0.84	0.84	0.95	0.76	0.72	0.64	0.71	0.81	0.62	1.08	1.17	0.99	1.00	0.94	1.13	0.73	0.76
408	ITA3_HUMAN	0.87	0.95	1.18	0.87	0.78	0.80	0.91	1.03	0.77	1.06	0.96	1.06	1.01	0.87	1.04	0.71	0.63
420	ITA3_HUMAN	0.93	0.97	1.28	0.86	0.84	0.77	0.88	0.93	0.69	1.07	1.16	0.99	1.10	0.93	1.11	0.65	0.63
195754	ITA3_HUMAN	0.94	1.05	1.40	0.92	0.75	0.74	0.91	1.03	0.67	1.16	1.15	1.14	1.12	0.83	1.12	0.61	0.55
927	K1C17_HUMAN	1.18	1.30	1.25	1.14	0.94	0.92	0.92	1.11	0.81	0.95	1.02	0.92	0.80	0.69	0.87	0.70	0.66
981	K1C17_HUMAN	1.04	0.81	0.68	0.74	0.78	0.62	0.67	0.71	0.76	0.78	0.72	0.56	0.47	0.75	0.63	0.72	0.81
1080	K1C18_HUMAN	0.77	0.78	0.62	0.81	0.73	0.59	0.70	0.83	0.65	0.90	0.91	0.95	0.83	0.84	0.97	0.86	0.96
417	K1C9_HUMAN	1.58	1.86	2.76	1.57	2.34	3.08	1.43	1.57	1.54	0.85	0.98	0.84	0.88	1.07	0.83	0.95	0.54
943	K2C8_HUMAN	0.80	0.70	0.50	0.82	0.67	0.62	0.75	0.87	0.59	1.05	1.00	1.07	0.93	0.99	1.23	1.29	1.18
202423	K2C8_HUMAN	1.22	1.14	0.84	1.10	1.34	0.91	1.04	0.55	0.70	1.10	1.99	0.99	1.69	1.30	0.96	1.19	1.67
745	KAP0_HUMAN	2.02	2.75	3.59	1.80	2.49	3.64	1.96	2.46	2.60	0.88	0.91	0.78	0.88	0.80	0.81	0.89	0.66
959	KAP0_HUMAN	0.71	0.70	0.55	0.78	0.62	0.54	0.72	0.76	0.52	0.97	0.97	1.07	0.98	0.86	1.06	0.95	0.91
989	KAP0_HUMAN	1.07	1.23	1.41	1.00	0.91	0.83	0.92	1.02	0.79	1.02	1.07	0.95	0.92	0.76	0.88	0.60	0.60
3670	KAP2_HUMAN	0.83	0.56	0.48	0.92	0.66	0.57	0.83	0.83	0.65	1.03	0.93	1.15	0.93	1.21	1.37	1.24	1.26
556498	KBP_HUMAN	0.97	1.61	1.19	1.02	1.04	1.11	0.39	1.01	0.43	0.75	1.84	0.79	0.75	0.48	1.15	0.70	0.66
207376	KCRB_HUMAN	0.77	0.79	0.68	0.73	0.54	0.59	0.54	0.62	0.36	0.84	0.98	0.80	0.68	0.58	0.77	0.73	0.52
951	LA_HUMAN	0.74	0.69	0.59	0.80	0.79	0.73	0.71	0.86	0.71	0.94	1.01	1.01	0.97	1.08	1.26	1.16	1.22
1821	LEG1_HUMAN	1.39	1.50	1.69	1.19	0.86	0.97	1.75	1.21	2.08	1.42	1.08	1.22	1.36	0.81	0.87	0.82	1.33
1962730	LEG1_HUMAN	0.75	0.84	0.56	0.98	0.75	0.66	1.20	1.01	1.08	1.06	0.94	1.38	1.50	0.94	1.13	1.24	1.79
10509	LG3BP_HUMAN	1.45	1.65	1.80	1.29	1.27	1.28	0.90	1.26	1.01	1.06	1.20	0.94	0.74	0.81	0.92	0.75	0.67
778	LKHA4_HUMAN	0.79	0.39	0.07	0.83	0.92	0.74	0.82	0.92	0.85	0.89	0.83	0.93	0.87	2.11	1.96	8.99	9.57
765	LMNA_HUMAN	0.81	0.47	0.36	0.90	0.95	0.67	0.62	0.73	0.51	0.93	1.26	1.03	0.96	1.86	1.95	1.72	1.76
490428	LMNA_HUMAN	1.01	0.40	0.10	1.05	0.98	0.85	0.96	1.13	0.99	0.99	0.89	1.02	0.84	2.46	2.55	8.80	9.21
694	LMNB1_HUMAN	1.11	1.33	1.17	1.03	0.98	1.05	0.68	0.99	0.75	0.87	1.14	0.80	0.69	0.64	0.85	0.78	0.73
708	LMNB1_HUMAN	1.16	1.19	0.85	0.83	0.73	0.71	0.66	0.73	0.46	1.28	1.57	0.91	0.89	0.79	0.96	1.07	0.84
709	LMNB1_HUMAN	0.68	0.69	0.74	0.84	0.68	0.72	0.78	0.88	0.55	0.90	0.99	1.11	1.14	0.89	1.26	0.88	0.73
712	LMNB1_HUMAN	0.95	1.01	0.67	0.90	0.74	0.78	0.65	0.74	0.47	1.12	1.52	1.06	1.04	0.82	1.11	1.31	1.06
537545	LMNB1_HUMAN	1.28	1.18	0.82	0.80	0.75	0.69	0.54	0.67	0.37	1.19	1.76	0.75	0.75	0.76	1.00	1.00	0.79
395	LPPRC_HUMAN	0.68	0.68	0.72	0.67	0.67	0.56	0.69	0.77	0.62	0.99	0.95	0.97	0.96	0.97	1.08	0.77	0.82
1308	LZTL1_HUMAN	0.73	0.86	0.51	0.81	0.89	0.61	0.69	0.83	0.69	0.78	0.76	0.86	0.71	0.81	0.73	0.94	1.03
691	MAOX_HUMAN	1.20	0.91	1.12	1.48	1.88	1.99	1.66	1.16	1.84	0.86	0.93	1.06	1.29	1.76	1.17	1.53	1.53
44539	MARCS_HUMAN	0.95	0.92	1.18	1.11	0.85	0.88	0.58	0.75	1.18	0.97	1.42	1.13	0.87	0.90	1.16	0.72	1.42
409	MATR3_HUMAN	0.76	0.76	0.88	0.76	0.69	0.63	0.65	0.75	0.54	0.98	1.13	0.97	0.96	0.89	1.11	0.71	0.69
768	MCM6_HUMAN	2.41	2.86	3.89	1.87	2.52	3.98	2.28	2.50	2.92	0.92	0.92	0.71	0.87	0.81	0.80	0.94	0.69

3316	MCM6_HUMAN	2.26	2.79	3.86	1.79	2.34	3.56	2.09	2.33	2.66	0.97	0.96	0.77	0.89	0.82	0.80	0.90	0.66
585	MD1L1_HUMAN	0.47	0.51	0.38	0.70	0.56	0.58	0.75	0.85	0.62	0.99	0.93	1.48	1.48	1.08	1.56	1.52	1.52
15097	MEP50_HUMAN	0.93	1.05	0.66	0.99	0.90	0.80	0.78	0.98	0.81	0.95	1.05	1.01	0.88	0.81	0.98	1.14	1.30
1647	ML12A_HUMAN	0.59	0.78	0.52	0.72	0.70	0.54	0.65	0.70	0.57	0.88	0.88	1.07	0.96	0.79	0.79	0.91	0.96
1936868	ML12B_HUMAN	0.10	0.18	0.08	0.50	0.80	0.29	0.02	0.50	0.03	0.34	6.18	1.77	1.56	1.54	17.31	1.20	1.93
1041	MP2K2_HUMAN	0.75	0.70	0.20	0.78	0.79	0.65	0.80	0.81	0.69	1.05	1.03	1.10	1.09	1.18	1.19	3.37	3.51
902	MPPA_HUMAN	0.74	0.31	0.23	0.73	0.68	0.53	0.73	0.77	0.56	0.94	0.94	0.93	0.92	2.03	2.33	2.22	2.34
973	MPPB_HUMAN	0.74	0.79	0.53	0.84	0.83	0.72	0.72	0.93	0.69	0.88	0.92	0.99	0.89	0.92	1.08	1.19	1.21
998	MPPB_HUMAN	0.71	0.73	0.59	0.78	0.74	0.60	0.70	0.89	0.60	0.94	0.96	1.04	0.95	0.96	1.17	0.96	0.98
627	MRE11_HUMAN	1.14	1.40	1.56	1.21	1.43	1.87	1.07	1.15	1.33	0.82	0.98	0.87	0.91	0.84	0.80	0.99	0.83
508	MSH2_HUMAN	0.66	0.57	0.48	0.70	0.58	0.54	0.68	0.75	0.52	1.06	1.03	1.11	1.05	1.09	1.36	1.18	1.11
1128	MTNA_HUMAN	0.71	0.70	0.53	0.81	0.67	0.64	0.84	0.92	0.70	1.02	1.06	1.15	1.25	0.97	1.39	1.21	1.39
1145	MTNA_HUMAN	0.59	0.64	0.47	0.76	0.62	0.59	0.63	0.76	0.50	0.97	1.29	1.27	1.38	0.94	1.53	1.21	1.37
528317	MTNA_HUMAN	0.80	0.64	0.47	0.86	0.70	0.64	0.69	0.81	0.51	1.16	1.39	1.25	1.19	1.27	1.74	1.60	1.51
223745	MTND_HUMAN	0.82	1.10	0.87	0.79	0.86	0.63	1.05	0.82	0.96	1.06	0.73	1.01	0.93	0.82	0.54	0.77	0.81
460	MVP_HUMAN	0.64	0.60	0.39	0.73	0.61	0.53	0.70	0.78	0.59	1.07	1.07	1.24	1.17	1.08	1.38	1.43	1.60
466	MVP_HUMAN	0.60	0.56	0.38	0.76	0.62	0.55	0.70	0.82	0.64	0.95	0.87	1.20	1.01	1.06	1.29	1.38	1.47
487	MVP_HUMAN	0.76	0.74	0.51	0.80	0.67	0.59	0.65	0.81	0.63	1.09	1.17	1.15	1.01	1.00	1.28	1.26	1.44
7894	MVP_HUMAN	0.85	0.75	0.54	0.87	0.70	0.68	0.73	0.84	0.65	1.05	1.19	1.08	1.02	0.97	1.33	1.32	1.43
8700	MVP_HUMAN	0.41	0.56	0.41	1.06	0.71	0.70	0.69	1.02	0.57	0.87	1.59	2.22	2.64	1.11	2.91	1.48	2.24
197182	MVP_HUMAN	0.69	0.69	0.46	0.73	0.61	0.59	0.64	0.77	0.59	1.13	1.20	1.20	1.12	1.00	1.34	1.45	1.54
540509	MVP_HUMAN	0.68	0.70	0.51	0.77	0.68	0.64	0.73	0.87	0.63	1.04	0.99	1.19	1.07	1.01	1.22	1.30	1.21
584	MX1_HUMAN	0.63	0.66	0.40	0.76	0.61	0.69	0.67	0.86	0.60	1.06	1.15	1.29	1.23	0.99	1.51	1.83	1.73
17545	MX1_HUMAN	0.85	1.09	1.29	0.76	0.86	1.28	0.57	0.71	0.53	1.30	2.12	1.16	1.40	1.03	1.37	1.29	0.88
379418	MYG1_HUMAN	0.67	0.66	0.41	0.74	0.71	0.56	0.74	0.66	0.63	0.93	0.91	1.03	1.01	1.00	0.91	1.28	1.42
31063	MYH13_HUMAN	0.63	0.43	0.27	0.69	0.69	0.56	0.69	0.74	0.61	1.06	1.08	1.16	1.19	1.71	1.85	2.20	2.45
1052	MYL3_HUMAN	0.81	0.87	0.55	0.83	0.70	0.63	0.74	0.87	0.70	1.06	0.98	1.08	0.90	0.85	0.98	1.21	1.25
1755	MYL6_HUMAN	0.84	0.96	0.74	0.86	0.76	0.64	0.70	0.80	0.67	0.92	0.95	0.94	0.79	0.74	0.79	0.81	0.86
72360	MYL9_HUMAN	0.89	0.81	0.62	1.10	0.86	0.73	1.15	1.13	0.86	0.95	0.86	1.17	1.10	1.00	1.19	1.13	1.20
1953484	MYL9_HUMAN	0.57	0.64	0.49	0.67	0.79	0.46	0.84	0.80	0.63	0.72	0.58	0.85	0.86	0.88	0.71	0.67	0.74
1254	NACA_HUMAN	1.46	1.40	1.13	1.32	1.16	0.98	1.17	1.30	1.17	0.88	0.74	0.80	0.59	0.74	0.69	0.77	0.77
13655	NDRG3_HUMAN	1.00	1.00	0.72	0.87	0.80	0.70	0.61	0.90	0.58	1.04	1.34	0.90	0.82	0.83	1.20	1.00	1.08
659	NDUS1_HUMAN	0.88	0.88	0.59	0.77	0.60	0.57	0.70	0.83	0.48	1.12	1.17	0.98	0.93	0.76	1.10	1.08	0.94
697	NDUS1_HUMAN	1.60	1.44	1.82	1.63	2.22	2.57	1.55	1.08	1.80	0.89	1.11	0.91	1.08	1.37	0.84	1.26	1.11
1432	NNMT_HUMAN	0.75	0.79	0.68	0.81	0.75	0.63	0.87	0.92	0.67	1.04	0.94	1.13	1.08	1.00	1.09	0.95	0.91
1478	NNMT_HUMAN	1.73	1.93	2.47	1.35	1.19	1.11	0.97	1.08	0.90	1.13	1.31	0.89	0.74	0.69	0.73	0.51	0.48
845	NP1L1_HUMAN	1.76	2.29	2.82	1.48	1.76	2.59	1.46	1.81	1.80	1.00	1.04	0.84	0.86	0.77	0.82	0.92	0.67
926	NP1L1_HUMAN	1.04	1.00	0.75	1.16	0.79	0.65	0.92	1.16	0.85	0.94	0.81	1.05	0.71	0.74	0.94	0.82	0.93
929	NP1L1_HUMAN	1.09	1.00	0.75	1.20	0.92	0.74	0.91	1.13	0.96	0.84	0.76	0.93	0.63	0.77	0.86	0.83	0.97
842	NP1L4_HUMAN	1.86	2.45	3.01	1.65	2.18	3.20	1.85	2.30	2.43	0.89	0.87	0.79	0.87	0.80	0.82	0.95	0.70
846	NP1L4_HUMAN	1.72	2.27	2.81	1.55	1.98	2.86	1.74	2.09	2.19	0.92	0.90	0.82	0.91	0.80	0.83	0.93	0.70
2404447	NP1L4_HUMAN	1.46	1.86	2.11	1.38	1.66	2.33	1.64	1.94	2.01	0.93	0.83	0.89	0.93	0.83	0.86	1.03	0.79
1225	NPM_HUMAN	1.04	0.95	0.86	1.10	1.15	0.96	1.23	1.66	1.59	0.71	0.49	0.75	0.57	0.85	0.85	0.79	0.90
144537	NUBP1_HUMAN	0.71	0.70	0.53	0.79	0.74	0.55	0.77	0.78	0.62	0.86	0.79	0.95	0.86	0.91	0.89	0.90	0.93
825	NUCB1_HUMAN	1.23	1.50	1.84	1.08	1.23	1.54	1.17	1.27	1.32	1.02	0.97	0.90	0.92	0.84	0.82	0.86	0.70
3227	NUCB1_HUMAN	1.42	1.78	2.14	1.27	1.43	1.75	1.35	1.50	1.52	1.01	0.95	0.90	0.90	0.81	0.80	0.82	0.67
520	NUCL_HUMAN	0.76	0.63	0.50	0.75	0.60	0.62	0.63	0.76	0.52	1.03	1.14	1.02	0.94	0.98	1.36	1.27	1.19
31063	OAT_HUMAN	0.63	0.43	0.27	0.69	0.69	0.56	0.69	0.74	0.61	1.06	1.08	1.16	1.19	1.71	1.85	2.20	2.45
1255	ODPB_HUMAN	0.77	0.76	0.67	0.82	0.71	0.64	0.80	0.89	0.66	0.99	0.97	1.05	1.01	0.93	1.14	0.94	0.95
1282	ODPB_HUMAN	0.60	0.69	0.41	0.63	0.59	0.48	0.61	0.78	0.50	1.08	1.06	1.13	1.07	0.92	1.19	1.27	1.30
1312	OTUB1_HUMAN	0.81	0.76	0.60	0.81	0.72	0.63	0.83	0.87	0.75	1.07	0.94	1.06	0.95	1.02	1.07	1.13	1.17
550	P3H1_HUMAN	0.67	0.67	0.48	0.78	0.67	0.59	0.70	0.79	0.62	0.95	0.91	1.09	0.94	0.95	1.09	1.17	1.18
558	P3H3_HUMAN	0.83	0.39	0.13	0.92	0.88	0.62	0.77	0.85	0.62	1.12	1.22	1.23	1.13	2.56	2.69	5.28	5.71
813	P4HA1_HUMAN	0.99	1.01	1.27	1.02	1.01	1.22	0.97	1.15	0.92	0.89	0.87	0.91	0.85	0.89	0.99	0.86	0.64
951	PA2G4_HUMAN	0.74	0.69	0.59	0.80	0.79	0.73	0.71	0.86	0.71	0.94	1.01	1.01	0.97	1.08	1.26	1.16	1.22
1032	PA2G4_HUMAN	0.66	0.32	0.01	0.68	0.75	0.54	0.69	0.75	0.67	0.98	0.92	1.01	0.95	2.29	2.15	2,240,386	2,605,427
1304	PCNA_HUMAN	0.82	0.79	0.68	0.88	0.66	0.65	0.79	0.88	0.58	1.11	1.09	1.19	1.06	0.93	1.23	1.06	0.93
61862	PCNA_HUMAN	0.83	0.83	0.61	0.81	0.68	0.56	0.70	0.83	0.55	1.08	1.00	1.06	0.84	0.88	1.00	0.99	0.90

824	PDIA1_HUMAN	1.32	1.49	1.90	1.18	0.93	1.03	0.96	1.16	0.89	1.11	1.19	1.00	0.87	0.69	0.93	0.60	0.56
2239	PDIA1_HUMAN	1.57	2.02	3.04	1.36	1.15	1.40	1.05	1.38	1.15	1.02	1.18	0.89	0.79	0.58	0.81	0.47	0.45
691	PDIA3_HUMAN	1.20	0.91	1.12	1.48	1.88	1.99	1.66	1.16	1.84	0.86	0.93	1.06	1.29	1.76	1.17	1.53	1.53
813	PDIA3_HUMAN	0.99	1.01	1.27	1.02	1.01	1.22	0.97	1.15	0.92	0.89	0.87	0.91	0.85	0.89	0.99	0.86	0.64
836	PDIA3_HUMAN	1.31	1.58	1.97	1.30	1.24	1.75	1.28	1.29	1.29	1.01	1.11	1.00	1.08	0.79	0.91	0.90	0.73
854	PDIA3_HUMAN	1.12	1.30	1.58	1.12	0.90	0.94	0.85	1.04	0.78	1.06	1.24	1.05	0.94	0.73	1.00	0.63	0.61
864	PDIA3_HUMAN	1.07	1.28	1.45	1.02	1.00	0.91	0.82	0.88	0.70	1.00	1.24	0.95	0.95	0.78	0.86	0.63	0.60
964	PDIA3_HUMAN	0.80	0.80	0.62	0.83	0.65	0.64	0.74	0.87	0.65	1.06	1.03	1.10	0.96	0.86	1.13	1.09	1.08
234358	PDIA3_HUMAN	1.24	1.64	2.09	1.25	1.50	2.07	1.16	1.54	1.44	0.88	0.91	0.89	0.85	0.80	0.85	0.88	0.63
377849	PDIA3_HUMAN	0.81	0.84	0.69	0.86	0.77	0.65	0.74	0.88	0.61	1.02	1.16	1.08	1.05	0.94	1.22	0.96	1.03
451640	PDIA3_HUMAN	0.88	1.29	1.50	1.09	0.92	1.00	0.80	0.87	0.73	1.04	1.30	1.28	1.19	0.74	0.88	0.69	0.63
660	PDIA4_HUMAN	1.33	1.46	1.68	1.52	2.06	2.87	1.02	1.52	1.44	0.71	1.25	0.81	0.95	0.99	1.29	1.20	1.07
717	PDIA4_HUMAN	1.99	2.81	3.40	1.80	2.45	3.72	1.61	2.01	2.46	0.84	1.04	0.76	0.84	0.73	0.75	0.92	0.76
22159	PDIA4_HUMAN	1.47	2.16	2.40	1.47	1.91	2.97	1.38	1.80	2.07	0.81	0.96	0.81	0.90	0.72	0.80	1.00	0.83
120878	PDIA4_HUMAN	0.95	0.99	0.83	1.10	0.89	0.91	0.79	0.95	0.58	0.83	1.12	0.96	0.93	0.75	1.08	0.91	0.78
370380	PDIA4_HUMAN	1.08	1.24	1.17	1.07	1.08	1.42	1.14	1.38	1.59	0.91	0.79	0.90	0.82	0.79	0.88	1.10	1.06
927	PDIA6_HUMAN	1.18	1.30	1.25	1.14	0.94	0.92	0.92	1.11	0.81	0.95	1.02	0.92	0.80	0.69	0.87	0.70	0.66
958	PDIA6_HUMAN	0.81	0.84	0.66	0.90	0.80	0.71	0.77	0.93	0.73	0.94	0.93	1.05	0.87	0.89	1.02	1.01	1.03
982	PDIA6_HUMAN	1.28	1.36	1.60	1.20	0.91	1.01	0.99	1.15	0.98	1.04	1.14	0.97	0.88	0.70	0.96	0.65	0.69
13655	PDIA6_HUMAN	1.00	1.00	0.72	0.87	0.80	0.70	0.61	0.90	0.58	1.04	1.34	0.90	0.82	0.83	1.20	1.00	1.08
1784	PEA15_HUMAN	0.80	0.83	0.56	0.82	0.69	0.59	0.65	0.74	0.60	0.99	1.00	1.02	0.80	0.82	0.88	1.03	1.06
234358	PEPD_HUMAN	1.24	1.64	2.09	1.25	1.50	2.07	1.16	1.54	1.44	0.88	0.91	0.89	0.85	0.80	0.85	0.88	0.63
1296	PGP_HUMAN	0.70	0.84	0.67	0.82	0.76	0.58	0.66	0.89	0.61	1.01	1.09	1.18	1.01	0.91	1.15	0.87	0.98
1468	PHB_HUMAN	0.97	1.02	0.79	0.97	0.82	0.71	0.77	0.88	0.58	0.96	1.05	0.95	0.84	0.77	0.91	0.86	0.77
16655	PHP14_HUMAN	0.70	0.82	0.60	0.83	0.66	0.64	1.91	0.89	1.56	1.76	1.45	2.10	3.96	1.41	1.56	1.87	3.76
1492	PIMT_HUMAN	0.88	0.41	0.60	0.96	0.80	0.76	0.87	1.00	0.73	0.98	1.07	1.07	1.07	1.91	2.59	1.25	1.32
577	PLOD3_HUMAN	0.90	0.85	0.61	0.88	0.72	0.69	0.88	0.93	0.69	1.06	1.04	1.04	1.01	0.90	1.14	1.20	1.18
25674	PLOD3_HUMAN	1.46	1.33	1.45	1.12	0.98	0.84	0.84	0.96	0.74	1.03	1.26	0.79	0.73	0.75	0.91	0.59	0.64
384077	PLST_HUMAN	0.79	0.83	0.70	0.86	0.90	0.95	0.65	0.87	0.82	0.88	1.06	0.96	0.87	0.94	1.11	1.19	1.23
843	PNCB_HUMAN	0.81	0.80	0.66	0.82	0.74	0.62	0.75	0.86	0.72	0.96	0.88	0.97	0.81	0.89	0.94	0.90	0.97
1443	PNPO_HUMAN	0.84	0.67	0.16	0.91	0.85	0.75	0.84	0.93	0.77	0.93	0.95	1.01	0.95	1.19	1.31	4.42	4.60
1272	PP1A_HUMAN	0.88	0.61	0.27	1.04	0.98	0.80	0.72	0.90	0.74	0.71	0.80	0.85	0.66	1.14	1.17	2.09	2.19
1030	PP1R7_HUMAN	0.78	0.70	0.53	0.81	0.66	0.56	0.76	0.74	0.58	1.09	1.06	1.14	1.04	1.02	1.12	1.15	1.16
1279	PP2AB_HUMAN	0.66	0.82	0.49	0.73	0.74	0.60	0.76	0.84	0.64	1.03	0.95	1.15	1.09	0.93	0.98	1.25	1.24
446	PPCE_HUMAN	0.40	0.61	0.36	0.72	0.45	0.38	0.59	0.76	0.38	1.00	1.12	1.80	1.66	0.73	1.40	1.05	1.21
452477	PPCE_HUMAN	1.16	1.61	1.55	1.25	1.86	2.65	1.34	2.54	1.87	0.78	0.88	0.84	1.02	0.90	1.39	1.34	1.07
2382844	PPM1G_HUMAN	0.79	0.78	0.75	0.76	0.64	0.63	0.71	0.83	0.55	1.06	0.97	1.03	0.87	0.88	1.04	0.89	0.72
1060	PPME1_HUMAN	0.80	0.80	0.57	0.72	0.75	0.59	0.80	0.81	0.67	0.90	0.87	0.81	0.87	0.85	0.88	0.94	1.01
1084	PPME1_HUMAN	0.69	0.72	0.47	0.64	0.70	0.51	0.56	0.71	0.65	1.11	1.15	1.02	0.92	1.08	1.13	1.19	1.57
418384	PPME1_HUMAN	0.80	1.00	0.88	0.74	0.67	0.50	0.58	0.52	0.54	1.23	1.32	1.13	0.96	0.82	0.68	0.70	0.81
3369954	PPME1_HUMAN	0.76	0.56	0.23	0.01	0.00	0.00	0.96	0.67	0.00	6.26	1.25	0.04	1.57	0.00	1.49	0.00	0.00
893	PPP5_HUMAN	0.84	0.38	0.14	0.86	0.85	0.72	0.85	0.87	0.83	0.92	0.89	0.95	0.90	2.06	2.01	4.73	5.26
1296	PPP6_HUMAN	0.70	0.84	0.67	0.82	0.76	0.58	0.66	0.89	0.61	1.01	1.09	1.18	1.01	0.91	1.15	0.87	0.98
2198480	PPT1_HUMAN	0.44	0.11	0.01	0.05	0.16	0.00	0.17	0.74	0.02	10.34	1.36	1.21	0.53	14.88	8.80	0.00	66.39
1541	PRDX2_HUMAN	0.71	0.77	0.59	0.85	0.71	0.65	0.74	0.84	0.60	1.04	1.14	1.24	1.19	0.95	1.25	1.14	1.17
1510	PRDX3_HUMAN	0.69	0.28	0.05	0.79	0.65	0.53	0.79	0.85	0.55	1.02	1.07	1.18	1.23	2.38	3.27	11.47	12.58
1471	PRDX4_HUMAN	0.90	1.11	1.21	1.08	0.98	0.91	0.99	1.16	0.92	0.87	0.81	1.04	0.89	0.77	0.85	0.65	0.62
3056834	PRDX4_HUMAN	1.41	1.23	1.46	1.22	1.08	1.12	2.04	2.22	1.97	1.06	0.62	0.91	0.90	0.93	1.12	0.80	0.84
1802	PROF2_HUMAN	0.76	0.90	0.64	0.80	0.66	0.53	1.01	0.84	0.95	1.35	1.04	1.42	1.39	0.98	0.97	1.12	1.55
177384	PRS6A_HUMAN	0.87	0.86	0.69	0.88	0.75	0.66	0.83	0.89	0.72	0.95	0.84	0.95	0.80	0.83	0.88	0.91	0.88
3102815	PRS6A_HUMAN	0.86	0.97	0.89	0.89	0.78	0.68	0.69	0.81	0.60	0.99	1.09	1.02	0.87	0.80	0.91	0.75	0.74
958	PRS6B_HUMAN	0.81	0.84	0.66	0.90	0.80	0.71	0.77	0.93	0.73	0.94	0.93	1.05	0.87	0.89	1.02	1.01	1.03
997	PRS7_HUMAN	0.82	0.86	0.64	0.83	0.88	0.68	0.78	0.86	0.72	0.87	0.85	0.88	0.81	0.89	0.86	0.93	0.97
1446	PSA3_HUMAN	0.72	0.81	0.59	0.83	0.70	0.59	0.67	0.77	0.57	0.96	1.04	1.10	0.96	0.83	0.98	0.96	1.00
1458	PSB10_HUMAN	0.84	0.89	0.77	0.85	0.66	0.63	0.81	0.81	0.59	1.19	1.22	1.20	1.18	0.88	1.11	0.96	0.93
16938	PSB4_HUMAN	1.06	1.05	1.07	0.96	1.01	0.86	0.82	1.07	0.83	0.85	0.85	0.76	0.65	0.82	0.87	0.67	0.66
3058865	PSB6_HUMAN	0.88	1.07	0.62	0.86	0.83	0.72	0.70	0.89	0.55	1.07	1.25	1.04	0.98	0.83	1.05	1.24	1.10
1402	PSB7_HUMAN	0.84	0.85	0.62	0.93	0.90	0.74	0.90	0.95	0.85	0.85	0.81	0.93	0.86	0.89	0.90	1.00	1.11

422947	PSB7_HUMAN	0.77	0.88	0.63	0.78	0.88	0.61	0.77	0.75	0.70	0.79	0.75	0.80	0.75	0.79	0.63	0.77	0.83
1553	PSB9_HUMAN	0.99	1.10	0.86	0.90	0.93	0.69	0.71	0.92	0.70	1.04	1.03	0.94	0.74	0.87	0.86	0.84	0.84
1352	PSDE_HUMAN	0.81	0.73	0.63	0.79	0.72	0.72	0.77	0.74	0.52	1.17	1.36	1.15	1.29	1.16	1.38	1.32	1.10
954	PSMD5_HUMAN	0.77	0.71	0.51	0.75	0.71	0.59	0.75	0.84	0.67	1.08	0.97	1.04	0.94	1.08	1.15	1.25	1.27
1433	PSME1_HUMAN	1.04	1.28	1.52	1.13	0.98	0.96	0.86	0.97	0.81	0.99	1.13	1.07	0.93	0.76	0.86	0.62	0.60
1373	PSME2_HUMAN	1.21	1.42	1.82	0.96	0.87	0.78	0.77	0.82	0.72	1.08	1.17	0.85	0.75	0.65	0.67	0.46	0.46
1392	PSME2_HUMAN	0.87	0.82	0.71	0.81	0.67	0.63	0.70	0.79	0.57	1.19	1.25	1.11	1.01	0.98	1.21	1.05	1.01
1409	PSME2_HUMAN	0.81	0.77	0.64	0.79	0.74	0.61	0.80	0.84	0.68	1.06	1.05	1.04	1.04	1.02	1.15	1.03	1.13
3313934	PSME2_HUMAN	1.44	1.33	1.76	1.36	1.04	1.17	1.13	1.31	1.13	0.98	1.03	0.93	0.81	0.77	1.02	0.65	0.66
934	PTN1_HUMAN	0.77	0.32	0.02	0.81	0.71	0.61	0.70	0.81	0.61	0.97	1.10	1.02	1.00	2.19	2.79	68.19	76.90
1684	PTN1_HUMAN	0.96	1.38	1.02	0.95	0.97	0.86	0.78	1.01	0.93	0.93	0.87	0.93	0.71	0.66	0.63	0.78	0.79
906	PTRF_HUMAN	0.73	0.67	0.58	0.81	0.75	0.62	0.75	0.87	0.71	0.86	0.78	0.95	0.79	0.95	1.01	0.93	0.95
458579	PUR4_HUMAN	0.68	0.64	0.48	0.68	0.69	0.58	0.68	0.77	0.62	0.91	0.87	0.91	0.86	0.98	1.04	1.09	1.12
201269	QCR1_HUMAN	0.63	0.56	0.55	0.55	0.58	0.41	0.49	0.70	0.48	1.01	0.95	0.88	0.73	1.05	1.19	0.76	0.84
964	QCR1_HUMAN	0.80	0.80	0.62	0.83	0.65	0.64	0.74	0.87	0.65	1.06	1.03	1.10	0.96	0.86	1.13	1.09	1.08
373121	QCR1_HUMAN	0.80	0.84	0.56	0.77	0.69	0.60	0.64	0.81	0.65	0.97	1.01	0.93	0.81	0.79	0.97	1.02	1.16
3435858	QCR1_HUMAN	0.86	0.65	0.56	0.26	2.59	17.97	0.37	0.93	2.85	0.92	0.72	0.28	0.31	3.66	1.04	29.60	3.68
714	RANB3_HUMAN	2.14	2.56	3.02	1.52	1.93	2.77	1.90	2.13	2.38	1.05	0.88	0.75	0.78	0.79	0.73	0.96	0.70
1446	RANG_HUMAN	0.72	0.81	0.59	0.83	0.70	0.59	0.67	0.77	0.57	0.96	1.04	1.10	0.96	0.83	0.98	0.96	1.00
953	RBBP7_HUMAN	0.76	0.81	0.54	0.86	0.75	0.64	0.74	0.85	0.62	0.92	0.87	1.04	0.86	0.84	0.91	1.09	1.00
13655	RBBP7_HUMAN	1.00	1.00	0.72	0.87	0.80	0.70	0.61	0.90	0.58	1.04	1.34	0.90	0.82	0.83	1.20	1.00	1.08
1586	RBM8A_HUMAN	0.83	0.84	0.60	0.98	0.78	0.74	0.84	0.90	0.67	0.98	1.02	1.16	1.03	0.91	1.09	1.21	1.14
1596	RBM8A_HUMAN	0.69	0.69	0.49	0.79	0.81	0.65	0.68	0.71	0.50	0.83	0.72	0.97	0.72	0.98	0.75	1.12	0.74
1604	RBM8A_HUMAN	0.68	0.65	0.40	0.70	0.74	0.54	0.59	0.80	0.26	0.91	0.88	0.93	0.77	1.03	1.09	1.23	0.58
1127	RCN1_HUMAN	0.96	1.19	1.22	0.95	0.90	0.74	0.70	0.78	0.61	1.05	1.23	1.05	0.90	0.79	0.81	0.64	0.62
280677	RCN1_HUMAN	1.47	1.76	2.03	1.33	1.14	1.16	0.88	1.07	0.90	1.13	1.48	1.02	0.89	0.73	0.90	0.64	0.66
2662678	RCN1_HUMAN	1.24	1.36	1.57	1.15	0.86	1.07	0.91	1.21	0.93	1.13	1.24	1.04	0.91	0.72	1.11	0.77	0.73
2680471	RCN1_HUMAN	1.12	1.31	1.59	1.21	0.91	0.93	0.83	0.89	0.77	1.04	1.33	1.13	0.98	0.73	0.91	0.61	0.65
2687300	RCN1_HUMAN	1.52	1.87	2.30	1.55	1.16	1.36	0.92	1.05	0.95	1.24	1.86	1.27	1.12	0.77	1.05	0.74	0.77
935	RD23A_HUMAN	0.87	0.73	0.55	0.88	0.55	0.46	0.93	0.87	0.54	1.29	1.00	1.30	1.06	0.97	1.20	1.07	0.99
94683	RD23B_HUMAN	1.02	1.14	1.30	0.96	0.88	0.90	0.80	0.77	0.67	1.05	1.21	0.98	0.94	0.81	0.82	0.72	0.63
3128108	RD23B_HUMAN	1.95	2.35	3.17	1.42	2.08	3.42	1.64	1.72	2.28	0.90	1.04	0.65	0.88	0.80	0.77	0.97	0.75
1367	RFA2_HUMAN	1.02	0.22	0.16	0.99	0.83	0.89	0.99	0.97	0.79	1.12	1.20	1.08	1.16	4.19	5.28	6.14	5.80
1375	RFA2_HUMAN	0.66	0.51	0.41	0.83	0.63	0.50	0.90	0.87	0.51	0.95	0.96	1.19	1.30	1.18	1.64	1.14	1.19
1821	RFA3_HUMAN	1.39	1.50	1.69	1.19	0.86	0.97	1.75	1.21	2.08	1.42	1.08	1.22	1.36	0.81	0.87	0.82	1.33
1146	RFC2_HUMAN	0.77	0.36	0.01	0.93	0.79	0.75	0.77	0.82	0.60	0.97	1.06	1.18	1.07	2.12	2.42	2,783,752	2,465,027
526716	RFC2_HUMAN	1.07	0.30	2.02	0.98	0.88	0.83	0.97	0.98	0.92	0.89	0.87	0.82	0.79	2.60	2.82	0.36	0.40
1005	RINI_HUMAN	0.66	0.68	0.38	0.69	0.67	0.50	0.69	0.78	0.69	1.01	0.86	1.04	0.89	1.00	1.00	1.30	1.54
1128	ROAA_HUMAN	0.71	0.70	0.53	0.81	0.67	0.64	0.84	0.92	0.70	1.02	1.06	1.15	1.25	0.97	1.39	1.21	1.39
1137	ROAA_HUMAN	0.70	0.28	0.84	0.82	0.73	0.61	0.70	0.78	0.58	0.96	1.10	1.13	1.10	2.46	3.02	0.70	0.75
1139	ROAA_HUMAN	0.80	0.42	0.15	0.90	0.78	0.79	0.75	0.84	0.65	0.93	1.13	1.04	1.05	1.70	2.23	5.03	5.04
528317	ROAA_HUMAN	0.80	0.64	0.47	0.86	0.70	0.64	0.69	0.81	0.51	1.16	1.39	1.25	1.19	1.27	1.74	1.60	1.51
1723	RPR1B_HUMAN	0.62	1.32	0.30	0.55	0.68	0.59	1.06	0.88	0.73	1.23	0.97	1.10	1.68	0.64	0.65	2.45	2.40
17216	RPR1B_HUMAN	0.44	0.59	0.27	0.50	0.55	0.36	0.41	0.55	0.32	1.11	1.16	1.25	1.08	1.03	1.09	1.51	1.42
1156	RSSA_HUMAN	0.92	1.19	0.89	0.91	0.79	0.73	0.69	0.77	0.59	1.01	1.16	1.00	0.88	0.67	0.75	0.83	0.78
92002	RSSA_HUMAN	0.85	0.96	0.67	0.78	0.58	0.61	0.78	0.86	0.76	1.37	0.86	1.26	0.78	0.83	0.77	1.26	0.97
888	RUVB1_HUMAN	0.87	0.62	0.46	0.93	0.86	0.73	0.88	0.97	0.81	0.93	0.89	0.98	0.89	1.28	1.38	1.47	1.56
201269	RUVB2_HUMAN	0.63	0.56	0.55	0.55	0.58	0.41	0.49	0.70	0.48	1.01	0.95	0.88	0.73	1.05	1.19	0.76	0.84
377849	RUVB2_HUMAN	0.81	0.84	0.69	0.86	0.77	0.65	0.74	0.88	0.61	1.02	1.16	1.08	1.05	0.94	1.22	0.96	1.03
1032	SAHH_HUMAN	0.66	0.32	0.01	0.68	0.75	0.54	0.69	0.75	0.67	0.98	0.92	1.01	0.95	2.29	2.15	2,240,386	2,605,427
1087	SAHH_HUMAN	1.02	0.60	0.22	1.06	1.02	0.92	0.94	1.01	0.84	0.95	1.00	0.98	0.91	1.61	1.68	4.04	3.86
1093	SCMC1_HUMAN	0.70	0.38	0.06	0.79	0.65	0.63	0.86	0.83	0.63	1.02	1.01	1.16	1.25	1.74	2.18	10.97	10.76
1139	SCMC1_HUMAN	0.80	0.42	0.15	0.90	0.78	0.79	0.75	0.84	0.65	0.93	1.13	1.04	1.05	1.70	2.23	5.03	5.04
418384	SCMC1_HUMAN	0.80	1.00	0.88	0.74	0.67	0.50	0.58	0.52	0.54	1.23	1.32	1.13	0.96	0.82	0.68	0.70	0.81
937	SCRN1_HUMAN	0.69	0.66	0.39	0.75	0.62	0.53	0.68	0.76	0.60	1.05	1.06	1.15	1.05	0.99	1.21	1.44	1.65
31063	SEP15_HUMAN	0.63	0.43	0.27	0.69	0.69	0.56	0.69	0.74	0.61	1.06	1.08	1.16	1.19	1.71	1.85	2.20	2.45
921	SEPT8_HUMAN	0.67	0.66	0.54	0.72	0.61	0.54	0.75	0.79	0.60	1.05	1.05	1.12	1.16	0.96	1.24	1.05	1.18
1052	SET_HUMAN	0.81	0.87	0.55	0.83	0.70	0.63	0.74	0.87	0.70	1.06	0.98	1.08	0.90	0.85	0.98	1.21	1.25

1156	SET_HUMAN	0.92	1.19	0.89	0.91	0.79	0.73	0.69	0.77	0.59	1.01	1.16	1.00	0.88	0.67	0.75	0.83	0.78
1157	SET_HUMAN	0.83	0.89	0.61	0.90	0.78	0.61	0.67	0.84	0.61	0.93	0.90	1.01	0.72	0.81	0.85	0.93	0.89
90190	SET_HUMAN	1.19	0.77	0.56	1.10	0.69	0.86	0.80	0.81	0.69	1.13	1.29	1.05	0.87	1.03	1.35	1.74	1.58
92002	SET_HUMAN	0.85	0.96	0.67	0.78	0.58	0.61	0.78	0.86	0.76	1.37	0.86	1.26	0.78	0.83	0.77	1.26	0.97
306795	SET_HUMAN	0.66	0.56	0.21	0.56	0.50	0.46	0.67	0.55	0.59	1.23	0.77	1.04	0.78	1.09	0.75	2.68	2.15
2906459	SET_HUMAN	0.81	0.73	0.45	0.96	0.80	0.73	0.78	0.90	0.69	0.87	0.88	1.03	0.85	0.95	1.08	1.40	1.34
3762560	SF3A3_HUMAN	0.86	0.83	0.69	0.85	0.71	0.66	0.73	0.81	0.65	1.01	1.02	1.00	0.86	0.87	1.00	0.96	0.95
354	SF3B2_HUMAN	0.55	0.47	0.16	0.81	0.66	0.59	0.58	0.70	0.51	0.91	1.04	1.35	1.10	1.29	1.57	3.33	3.27
355	SF3B2_HUMAN	0.50	0.43	0.21	0.77	0.58	0.53	0.64	0.73	0.49	0.91	0.93	1.40	1.19	1.23	1.59	2.30	2.16
9421	SF3B2_HUMAN	1.22	1.53	1.93	1.05	0.95	0.99	0.81	0.94	0.73	1.22	1.46	1.05	0.97	0.76	0.90	0.62	0.55
954	SH3G1_HUMAN	0.77	0.71	0.51	0.75	0.71	0.59	0.75	0.84	0.67	1.08	0.97	1.04	0.94	1.08	1.15	1.25	1.27
13083	SH3G1_HUMAN	0.77	0.80	0.57	0.86	0.58	0.56	0.71	0.82	0.59	1.09	1.08	1.21	0.99	0.80	1.11	1.08	1.12
379418	SHLB1_HUMAN	0.67	0.66	0.41	0.74	0.71	0.56	0.74	0.66	0.63	0.93	0.91	1.03	1.01	1.00	0.91	1.28	1.42
950	SHLB2_HUMAN	0.74	1.02	0.58	0.91	0.97	0.69	0.84	0.95	0.90	0.80	0.72	0.99	0.82	0.76	0.67	0.96	1.12
972	SHLB2_HUMAN	0.62	0.72	0.48	0.75	0.61	0.56	0.69	0.77	0.54	1.08	1.11	1.30	1.24	0.91	1.19	1.25	1.25
408351	SHLB2_HUMAN	0.83	1.04	0.64	0.87	0.81	0.60	0.59	0.79	0.58	0.89	1.10	0.94	0.78	0.70	0.84	0.84	0.99
1628	SKP1_HUMAN	0.98	1.10	0.94	1.00	0.80	0.72	0.64	1.00	0.62	1.07	1.11	1.09	0.73	0.78	1.01	0.82	0.73
3104	SMCE1_HUMAN	0.92	0.86	0.65	0.86	0.86	0.80	0.73	0.90	0.75	0.94	1.01	0.88	0.81	0.93	1.05	1.15	1.16
1305	SNAA_HUMAN	0.73	0.76	0.60	0.71	0.66	0.54	0.72	0.72	0.61	1.05	0.92	1.02	0.92	0.91	0.88	0.95	0.93
1167	SNAG_HUMAN	0.82	0.77	0.61	0.92	0.80	0.70	0.82	0.92	0.75	0.90	0.91	1.01	0.91	0.93	1.08	1.03	1.12
918	SNX6_HUMAN	0.63	0.66	0.51	0.79	0.69	0.61	0.77	0.86	0.62	0.95	0.96	1.19	1.19	0.98	1.25	1.13	1.18
956	SNX6_HUMAN	0.68	0.70	0.50	0.78	0.71	0.60	0.80	0.84	0.67	0.98	0.97	1.13	1.13	0.99	1.16	1.18	1.32
983	SNX6_HUMAN	0.83	0.84	0.65	0.79	0.79	0.68	0.74	0.88	0.69	0.95	0.94	0.91	0.84	0.88	0.98	0.99	0.99
1651	SODC_HUMAN	0.87	0.90	0.75	1.09	0.83	0.84	0.76	0.99	0.66	0.93	1.23	1.16	1.07	0.86	1.35	1.04	1.09
30752	SODC_HUMAN	0.71	0.63	0.53	0.70	0.68	0.54	0.68	0.70	0.56	0.86	0.82	0.84	0.78	0.93	0.91	0.87	0.86
508072	SODC_HUMAN	0.57	0.68	0.47	0.77	0.86	0.57	0.65	0.82	0.49	0.92	0.98	1.24	1.11	1.17	1.19	1.13	1.03
1589	SORCN_HUMAN	0.72	0.72	0.47	0.84	0.79	0.61	0.74	0.84	0.60	0.91	0.92	1.06	0.94	1.00	1.07	1.17	1.17
3591281	SORCN_HUMAN	0.68	0.95	0.47	0.98	0.79	0.89	0.43	1.36	0.58	1.20	1.31	1.75	0.84	1.00	1.87	2.28	1.61
1308	SPEE_HUMAN	0.73	0.86	0.51	0.81	0.89	0.61	0.69	0.83	0.69	0.78	0.76	0.86	0.71	0.81	0.73	0.94	1.03
1127	SPRC_HUMAN	0.96	1.19	1.22	0.95	0.90	0.74	0.70	0.78	0.61	1.05	1.23	1.05	0.90	0.79	0.81	0.64	0.62
278067	SPRC_HUMAN	0.99	0.98	1.07	0.78	0.71	0.64	0.62	0.72	0.53	0.97	1.15	0.76	0.72	0.70	0.84	0.58	0.57
2666660	SPRC_HUMAN	0.93	1.44	1.35	1.03	0.88	0.83	0.60	0.72	0.60	1.21	1.84	1.34	1.20	0.74	0.92	0.74	0.82
2680471	SPRC_HUMAN	1.12	1.31	1.59	1.21	0.91	0.93	0.83	0.89	0.77	1.04	1.33	1.13	0.98	0.73	0.91	0.61	0.65
1083	SPS1_HUMAN	0.70	0.85	0.60	0.83	0.77	0.68	0.77	0.77	0.64	0.91	1.02	1.08	1.13	0.82	0.93	1.02	1.08
1121	SPSY_HUMAN	0.75	0.73	0.53	0.77	0.76	0.58	0.75	0.85	0.69	0.98	0.92	1.01	0.92	1.02	1.07	1.07	1.20
92002	SPSY_HUMAN	0.85	0.96	0.67	0.78	0.58	0.61	0.78	0.86	0.76	1.37	0.86	1.26	0.78	0.83	0.77	1.26	0.97
557	SRC8_HUMAN	0.70	0.72	0.49	0.79	0.69	0.58	0.65	0.89	0.63	1.03	1.06	1.16	0.99	0.98	1.32	1.22	1.35
559	SRC8_HUMAN	0.67	0.85	0.59	0.76	0.67	0.54	0.87	0.92	0.75	0.99	0.73	1.13	0.94	0.78	0.79	0.91	0.92
1305	SRSF1_HUMAN	0.73	0.76	0.60	0.71	0.66	0.54	0.72	0.72	0.61	1.05	0.92	1.02	0.92	0.91	0.88	0.95	0.93
1308	SRSF1_HUMAN	0.73	0.86	0.51	0.81	0.89	0.61	0.69	0.83	0.69	0.78	0.76	0.86	0.71	0.81	0.73	0.94	1.03
2995227	SRSF1_HUMAN	1.00	0.72	0.44	0.63	0.44	0.64	1.02	0.86	0.72	1.43	0.84	0.91	0.86	0.87	1.01	2.07	1.38
1345	SRSF2_HUMAN	1.51	1.54	1.93	1.40	1.05	1.33	0.86	1.31	0.84	1.12	1.54	1.04	0.88	0.77	1.31	0.77	0.67
1536	SRSF3_HUMAN	0.76	0.83	0.81	0.79	0.72	0.59	0.87	0.83	0.66	1.17	1.07	1.21	1.22	1.01	1.06	0.86	0.88
1565	SRSF3_HUMAN	0.76	0.80	0.63	0.71	0.71	0.57	0.68	0.84	0.58	1.17	1.03	1.09	0.92	1.04	1.08	1.06	0.94
1572	SRSF3_HUMAN	0.55	0.40	0.03	0.73	0.62	0.43	0.64	0.79	0.31	0.84	0.84	1.11	0.98	1.30	1.66	10.80	7.63
1578	SRSF3_HUMAN	0.79	0.73	0.75	0.75	0.55	0.44	0.71	0.69	0.55	1.15	0.95	1.10	0.85	0.87	0.90	0.67	0.70
506742	SRSF3_HUMAN	0.68	0.76	0.72	0.85	0.69	0.56	0.72	0.82	0.54	0.99	1.00	1.24	1.05	0.90	1.09	0.78	0.75
2287478	SRSF3_HUMAN	0.67	0.70	0.27	0.67	0.72	0.44	0.60	0.81	0.46	0.85	0.78	0.85	0.70	0.87	0.91	1.40	1.33
1638	SSRD_HUMAN	0.68	0.79	0.52	0.74	0.87	0.57	0.97	0.50	1.01	0.97	0.65	1.06	0.93	1.07	0.41	1.05	1.26
853	STABP_HUMAN	1.77	2.47	3.03	1.59	2.29	3.13	1.87	2.17	2.40	0.88	0.84	0.78	0.89	0.81	0.74	0.91	0.67
1553	STAM1_HUMAN	0.99	1.10	0.86	0.90	0.93	0.69	0.71	0.92	0.70	1.04	1.03	0.94	0.74	0.87	0.86	0.84	0.84
826	STK3_HUMAN	1.30	1.69	1.35	1.14	1.14	1.17	0.80	1.16	0.91	0.95	1.09	0.83	0.68	0.64	0.75	0.82	0.73
1119	STML2_HUMAN	1.51	1.56	2.05	1.33	1.12	1.19	1.18	1.21	1.06	0.98	1.01	0.86	0.79	0.71	0.78	0.57	0.52
1684	STMN1_HUMAN	0.96	1.38	1.02	0.95	0.97	0.86	0.78	1.01	0.93	0.93	0.87	0.93	0.71	0.66	0.63	0.78	0.79
1686	STMN1_HUMAN	1.02	1.56	1.14	1.04	1.16	0.94	0.93	1.27	1.13	0.92	0.78	0.93	0.71	0.68	0.63	0.76	0.77
1689	STMN1_HUMAN	0.64	0.64	0.48	0.82	0.58	0.54	0.66	0.73	0.44	1.03	1.22	1.33	1.27	0.93	1.39	1.16	1.12
1695	STMN1_HUMAN	0.89	0.79	0.65	1.06	0.65	0.73	0.78	0.92	0.56	1.05	1.30	1.25	1.13	0.86	1.50	1.19	1.13
1161	STRAP_HUMAN	0.77	0.88	0.54	0.82	0.91	0.65	0.76	0.89	0.77	0.86	0.79	0.92	0.78	0.89	0.79	1.03	1.13

305540	STRAP_HUMAN	0.83	1.00	1.21	0.70	0.76	0.62	1.44	1.51	1.35	0.94	0.53	0.80	0.92	0.71	0.80	0.49	0.60
2964442	STRAP_HUMAN	0.74	0.70	0.60	0.73	1.00	0.76	1.28	1.22	1.36	0.80	0.50	0.79	0.86	1.14	0.87	1.00	1.12
1058	SUCB1_HUMAN	0.51	0.62	0.46	0.69	0.57	0.47	0.74	0.84	0.55	0.92	0.84	1.23	1.21	0.84	1.14	0.93	1.00
144537	SUGT1_HUMAN	0.71	0.70	0.53	0.79	0.74	0.55	0.77	0.78	0.62	0.86	0.79	0.95	0.86	0.91	0.89	0.90	0.93
691	SWP70_HUMAN	1.20	0.91	1.12	1.48	1.88	1.99	1.66	1.16	1.84	0.86	0.93	1.06	1.29	1.76	1.17	1.53	1.53
460	SYAC_HUMAN	0.64	0.60	0.39	0.73	0.61	0.53	0.70	0.78	0.59	1.07	1.07	1.24	1.17	1.08	1.38	1.43	1.60
929	SYAP1_HUMAN	1.09	1.00	0.75	1.20	0.92	0.74	0.91	1.13	0.96	0.84	0.76	0.93	0.63	0.77	0.86	0.83	0.97
934	SYDC_HUMAN	0.77	0.32	0.02	0.81	0.71	0.61	0.70	0.81	0.61	0.97	1.10	1.02	1.00	2.19	2.79	68.19	76.90
974	SYDC_HUMAN	0.86	0.84	0.65	0.96	0.93	0.80	0.99	1.13	0.97	0.86	0.71	0.95	0.82	0.95	0.96	1.05	1.05
28319	SYG_HUMAN	0.68	0.60	6.91	0.76	0.74	0.62	0.79	0.87	0.86	0.89	0.85	0.99	0.98	1.09	1.23	0.08	0.11
908	SYHC_HUMAN	0.67	0.70	0.45	0.78	0.73	0.57	0.74	0.82	0.66	0.95	0.94	1.12	1.05	0.99	1.10	1.20	1.36
377849	SYHC_HUMAN	0.81	0.84	0.69	0.86	0.77	0.65	0.74	0.88	0.61	1.02	1.16	1.08	1.05	0.94	1.22	0.96	1.03
683	SYK_HUMAN	1.18	1.26	2.39	1.25	1.65	2.16	1.28	1.47	1.65	0.84	0.91	0.89	0.98	1.10	1.06	0.76	0.63
407629	SYK_HUMAN	0.78	1.00	1.29	1.26	2.91	4.41	1.56	1.18	2.07	0.64	0.96	1.03	1.92	1.84	1.12	2.18	1.54
885	SYWC_HUMAN	0.78	0.73	0.52	0.83	0.79	0.67	0.76	0.87	0.70	1.06	1.19	1.13	1.17	1.15	1.42	1.36	1.60
1046	TADBP_HUMAN	0.81	1.00	0.80	0.85	0.77	0.69	0.64	0.73	0.57	1.03	1.27	1.08	1.01	0.79	0.92	0.89	0.90
1078	TADBP_HUMAN	0.69	0.77	0.57	0.84	0.78	0.64	0.69	0.80	0.76	0.87	0.83	1.06	0.83	0.88	0.86	0.97	1.11
1084	TADBP_HUMAN	0.69	0.72	0.47	0.64	0.70	0.51	0.56	0.71	0.65	1.11	1.15	1.02	0.92	1.08	1.13	1.19	1.57
29100	TADBP_HUMAN	0.61	0.74	0.48	0.65	0.60	0.43	0.55	0.61	0.51	1.11	1.09	1.19	0.98	0.89	0.89	0.99	1.17
418384	TADBP_HUMAN	0.80	1.00	0.88	0.74	0.67	0.50	0.58	0.52	0.54	1.23	1.32	1.13	0.96	0.82	0.68	0.70	0.81
3329884	TADBP_HUMAN	1.28	1.64	1.55	1.30	1.29	1.53	1.22	1.17	1.26	0.81	0.88	0.82	0.84	0.63	0.63	0.80	0.72
3369954	TADBP_HUMAN	0.76	0.56	0.23	0.01	0.00	0.00	0.96	0.67	0.00	6.26	1.25	0.04	1.57	0.00	1.49	0.00	0.00
1270	TALDO_HUMAN	1.06	0.66	0.25	1.18	1.17	1.01	0.75	1.09	0.82	0.77	0.85	0.85	0.60	1.36	1.41	3.04	2.74
879	TBA1A_HUMAN	0.81	0.79	0.60	0.75	0.67	0.55	0.51	0.65	0.53	1.01	1.18	0.93	0.75	0.86	0.96	0.93	1.04
2200	TBA1A_HUMAN	1.66	2.22	2.82	1.50	2.11	2.96	1.77	2.07	2.25	0.88	0.87	0.80	0.92	0.84	0.81	0.93	0.69
191012	TBA1A_HUMAN	0.89	0.92	0.71	0.90	0.78	0.69	0.75	0.93	0.64	0.96	1.03	0.98	0.88	0.82	1.04	0.93	0.93
836	TBA1B_HUMAN	1.31	1.58	1.97	1.30	1.24	1.75	1.28	1.29	1.29	1.01	1.11	1.00	1.08	0.79	0.91	0.90	0.73
876	TBA1B_HUMAN	0.76	0.85	0.64	0.77	0.70	0.57	0.61	0.83	0.57	0.98	1.00	0.99	0.81	0.81	0.98	0.88	0.89
890	TBA1B_HUMAN	0.80	0.78	0.59	0.81	0.65	0.58	0.75	0.84	0.63	1.14	1.29	1.15	1.20	0.94	1.38	1.11	1.38
977	TBA1B_HUMAN	0.91	1.04	1.15	1.02	0.84	0.72	0.87	0.96	0.65	0.97	1.05	1.08	1.00	0.78	0.97	0.61	0.59
15904	TBA1B_HUMAN	1.17	1.21	0.98	0.90	0.74	0.73	0.56	0.72	0.58	1.20	1.42	0.92	0.68	0.74	0.85	0.89	0.84
402687	TBA1B_HUMAN	0.38	0.64	0.51	0.71	0.52	0.37	0.60	0.66	0.42	0.97	1.08	1.83	1.72	0.79	1.12	0.71	0.91
906	TBA1C_HUMAN	0.73	0.67	0.58	0.81	0.75	0.62	0.75	0.87	0.71	0.86	0.78	0.95	0.79	0.95	1.01	0.93	0.95
906	TBA4A_HUMAN	0.73	0.67	0.58	0.81	0.75	0.62	0.75	0.87	0.71	0.86	0.78	0.95	0.79	0.95	1.01	0.93	0.95
3104	TBB2A_HUMAN	0.92	0.86	0.65	0.86	0.86	0.80	0.73	0.90	0.75	0.94	1.01	0.88	0.81	0.93	1.05	1.15	1.16
875	TBB3_HUMAN	0.78	0.77	0.55	0.77	0.75	0.66	0.82	0.85	0.72	1.04	0.89	1.02	0.93	1.00	0.99	1.23	1.16
875	TBB4B_HUMAN	0.78	0.77	0.55	0.77	0.75	0.66	0.82	0.85	0.72	1.04	0.89	1.02	0.93	1.00	0.99	1.23	1.16
3104	TBB4B_HUMAN	0.92	0.86	0.65	0.86	0.86	0.80	0.73	0.90	0.75	0.94	1.01	0.88	0.81	0.93	1.05	1.15	1.16
93790	TBB4B_HUMAN	0.60	0.57	0.44	0.76	0.61	0.44	0.71	0.67	0.51	0.86	0.74	1.07	0.87	0.93	0.87	0.86	0.85
3111980	TBB4B_HUMAN	0.77	0.86	0.67	0.78	0.76	0.66	0.64	0.77	0.61	0.97	1.03	0.99	0.85	0.85	0.91	0.96	0.93
875	TBB5_HUMAN	0.78	0.77	0.55	0.77	0.75	0.66	0.82	0.85	0.72	1.04	0.89	1.02	0.93	1.00	0.99	1.23	1.16
906	TBB5_HUMAN	0.73	0.67	0.58	0.81	0.75	0.62	0.75	0.87	0.71	0.86	0.78	0.95	0.79	0.95	1.01	0.93	0.95
93790	TBB5_HUMAN	0.60	0.57	0.44	0.76	0.61	0.44	0.71	0.67	0.51	0.86	0.74	1.07	0.87	0.93	0.87	0.86	0.85
388879	TBB5_HUMAN	0.92	0.90	0.94	0.94	0.78	0.69	0.65	0.69	0.55	1.06	1.37	1.09	0.97	0.93	1.06	0.78	0.81
3104	TBB6_HUMAN	0.92	0.86	0.65	0.86	0.86	0.80	0.73	0.90	0.75	0.94	1.01	0.88	0.81	0.93	1.05	1.15	1.16
893	TBG1_HUMAN	0.84	0.38	0.14	0.86	0.85	0.72	0.85	0.87	0.83	0.92	0.89	0.95	0.90	2.06	2.01	4.73	5.26
973	TBG1_HUMAN	0.74	0.79	0.53	0.84	0.83	0.72	0.72	0.93	0.69	0.88	0.92	0.99	0.89	0.92	1.08	1.19	1.21
998	TBG1_HUMAN	0.71	0.73	0.59	0.78	0.74	0.60	0.70	0.89	0.60	0.94	0.96	1.04	0.95	0.96	1.17	0.96	0.98
879	TBL1R_HUMAN	0.81	0.79	0.60	0.75	0.67	0.55	0.51	0.65	0.53	1.01	1.18	0.93	0.75	0.86	0.96	0.93	1.04
191012	TBL1R_HUMAN	0.89	0.92	0.71	0.90	0.78	0.69	0.75	0.93	0.64	0.96	1.03	0.98	0.88	0.82	1.04	0.93	0.93
793	TCPA_HUMAN	0.83	0.79	0.61	0.89	0.83	0.71	0.92	0.98	0.86	0.90	0.80	0.97	0.89	0.96	1.00	1.06	1.14
813	TCPA_HUMAN	0.99	1.01	1.27	1.02	1.01	1.22	0.97	1.15	0.92	0.89	0.87	0.91	0.85	0.89	0.99	0.86	0.64
888	TCPB_HUMAN	0.87	0.62	0.46	0.93	0.86	0.73	0.88	0.97	0.81	0.93	0.89	0.98	0.89	1.28	1.38	1.47	1.56
586647	TCPB_HUMAN	0.81	0.42	0.28	0.82	0.79	0.68	0.88	0.80	0.76	0.97	0.88	0.98	0.96	1.84	1.71	2.32	2.37
843	TCPE_HUMAN	0.81	0.80	0.66	0.82	0.74	0.62	0.75	0.86	0.72	0.96	0.88	0.97	0.81	0.89	0.94	0.90	0.97
847	TCPE_HUMAN	0.95	0.93	0.69	0.93	0.81	0.73	0.64	0.91	0.64	0.95	1.13	0.92	0.76	0.83	1.10	1.01	1.05
760	TCPG_HUMAN	0.74	0.62	0.46	0.83	0.85	0.71	0.79	0.90	0.75	0.90	0.89	1.00	0.95	1.23	1.29	1.41	1.47
771	TCPG_HUMAN	0.91	0.40	0.10	0.92	0.91	0.83	0.83	0.99	0.84	0.96	1.01	0.96	0.92	2.17	2.49	7.87	8.50

796	TCPQ_HUMAN	0.84	0.71	0.64	0.94	0.70	0.73	0.96	0.96	0.69	0.98	0.99	1.11	1.14	0.97	1.34	1.13	1.08
843	TCPQ_HUMAN	0.81	0.80	0.66	0.82	0.74	0.62	0.75	0.86	0.72	0.96	0.88	0.97	0.81	0.89	0.94	0.90	0.97
1576	TCTP_HUMAN	0.84	0.81	0.59	0.91	0.81	0.69	0.79	0.90	0.69	0.91	0.86	0.99	0.81	0.91	0.95	1.07	0.99
2000013	TCTP_HUMAN	1.37	1.17	1.01	0.75	1.87	1.16	0.77	1.28	1.45	0.63	0.38	0.34	0.21	1.00	0.42	0.72	0.55
1610	TEBP_HUMAN	0.57	0.81	0.46	0.92	0.73	0.63	0.42	0.71	0.35	0.78	1.44	1.27	1.07	0.70	1.26	1.06	1.09
2310	TEBP_HUMAN	0.74	0.79	0.44	0.93	0.79	0.67	0.56	0.52	0.44	0.89	1.27	1.12	0.95	0.89	0.83	1.35	1.27
542	TERA_HUMAN	0.88	0.90	0.63	0.91	0.74	0.76	0.76	0.96	0.74	1.02	1.08	1.04	0.93	0.84	1.16	1.23	1.26
9875	TERA_HUMAN	0.70	0.67	0.45	0.78	0.64	0.57	0.73	0.86	0.66	0.97	0.93	1.09	0.97	0.93	1.19	1.22	1.34
22159	TF65_HUMAN	1.47	2.16	2.40	1.47	1.91	2.97	1.38	1.80	2.07	0.81	0.96	0.81	0.90	0.72	0.80	1.00	0.83
1821	THIO_HUMAN	1.39	1.50	1.69	1.19	0.86	0.97	1.75	1.21	2.08	1.42	1.08	1.22	1.36	0.81	0.87	0.82	1.33
1115	THOC3_HUMAN	0.71	0.88	0.60	0.96	0.97	0.79	0.72	0.85	0.66	0.91	1.11	1.23	1.13	1.00	1.07	1.20	1.22
414193	THOC3_HUMAN	0.79	0.79	0.58	0.87	0.79	0.71	0.72	0.76	0.62	0.97	1.14	1.07	1.04	0.97	1.09	1.18	1.22
607	THOP1_HUMAN	0.95	0.92	0.72	0.82	0.73	0.70	0.77	0.90	0.61	1.13	1.20	0.97	0.97	0.90	1.18	1.10	1.02
663	THOP1_HUMAN	0.64	0.63	0.52	0.68	0.67	0.56	0.71	0.79	0.67	1.05	0.91	1.11	1.01	1.11	1.13	1.14	1.18
663	THOP1_HUMAN	0.64	0.63	0.52	0.68	0.67	0.56	0.71	0.79	0.67	1.05	0.91	1.11	1.01	1.11	1.13	1.14	1.18
478	TIF1B_HUMAN	0.62	0.61	0.40	0.69	0.60	0.54	0.65	0.75	0.56	1.12	1.12	1.25	1.18	1.09	1.36	1.50	1.56
479	TIF1B_HUMAN	0.47	0.30	0.20	0.65	0.41	0.45	0.73	0.73	0.62	0.99	0.79	1.37	1.23	1.35	1.91	2.27	2.49
1449	TIF1B_HUMAN	0.86	0.92	0.74	0.90	0.77	0.68	0.67	0.85	0.58	1.02	1.15	1.07	0.90	0.85	1.05	0.95	0.91
7894	TIF1B_HUMAN	0.85	0.75	0.54	0.87	0.70	0.68	0.73	0.84	0.65	1.05	1.19	1.08	1.02	0.97	1.33	1.32	1.43
471051	TIF1B_HUMAN	0.69	0.32	0.20	0.79	0.75	0.64	0.78	0.81	0.66	0.88	0.91	1.01	1.02	2.04	2.29	2.81	2.98
537545	TKT_HUMAN	1.28	1.18	0.82	0.80	0.75	0.69	0.54	0.67	0.37	1.19	1.76	0.75	0.75	0.76	1.00	1.00	0.79
774	TM1L2_HUMAN	2.19	2.79	3.75	1.51	2.16	3.25	1.87	2.04	2.38	1.05	1.00	0.72	0.85	0.81	0.73	0.91	0.63
1047	TMOD3_HUMAN	0.78	0.95	0.66	0.83	0.85	0.64	0.81	0.85	0.76	0.90	0.79	0.96	0.83	0.80	0.71	0.87	0.91
3085623	TMOD3_HUMAN	0.71	1.01	0.99	0.72	0.68	0.93	0.67	0.58	0.76	1.09	0.77	1.11	0.72	0.74	0.44	1.03	0.59
1315	TPM1_HUMAN	0.76	0.82	0.65	0.59	0.62	0.47	0.49	0.54	0.52	1.09	1.16	0.85	0.75	0.83	0.76	0.79	0.93
1372	TPM1_HUMAN	0.80	0.77	0.58	0.85	0.72	0.67	0.83	0.92	0.75	1.05	0.98	1.11	1.02	0.98	1.16	1.22	1.28
55610	TPM1_HUMAN	0.76	0.61	0.50	0.49	0.47	0.36	0.42	0.46	0.42	1.14	1.16	0.74	0.64	0.89	0.87	0.84	0.98
1372	TPM2_HUMAN	0.80	0.77	0.58	0.85	0.72	0.67	0.83	0.92	0.75	1.05	0.98	1.11	1.02	0.98	1.16	1.22	1.28
55610	TPM2_HUMAN	0.76	0.61	0.50	0.49	0.47	0.36	0.42	0.46	0.42	1.14	1.16	0.74	0.64	0.89	0.87	0.84	0.98
56308	TPM2_HUMAN	0.86	0.82	0.64	0.82	0.73	0.65	0.68	0.92	0.77	0.98	1.08	0.93	0.86	0.88	1.21	1.01	1.31
87491	TPM2_HUMAN	0.98	1.04	0.89	0.91	0.93	0.69	0.83	0.94	1.02	0.93	0.76	0.87	0.64	0.83	0.69	0.72	0.88
1372	TPM3_HUMAN	0.80	0.77	0.58	0.85	0.72	0.67	0.83	0.92	0.75	1.05	0.98	1.11	1.02	0.98	1.16	1.22	1.28
1372	TPM3L_HUMAN	0.80	0.77	0.58	0.85	0.72	0.67	0.83	0.92	0.75	1.05	0.98	1.11	1.02	0.98	1.16	1.22	1.28
1369	TPM4_HUMAN	0.92	0.88	0.66	0.89	0.77	0.66	0.80	0.87	0.71	1.00	0.98	0.96	0.86	0.88	0.97	1.01	1.07
1372	TPM4_HUMAN	0.80	0.77	0.58	0.85	0.72	0.67	0.83	0.92	0.75	1.05	0.98	1.11	1.02	0.98	1.16	1.22	1.28
13616	TPP1_HUMAN	0.89	0.93	0.76	0.94	0.75	0.72	0.80	0.90	0.65	0.90	0.93	0.94	0.84	0.72	0.90	0.85	0.80
1802	TR112_HUMAN	0.76	0.90	0.64	0.80	0.66	0.53	1.01	0.84	0.95	1.35	1.04	1.42	1.39	0.98	0.97	1.12	1.55
683	TRFL_HUMAN	1.18	1.26	2.39	1.25	1.65	2.16	1.28	1.47	1.65	0.84	0.91	0.89	0.98	1.10	1.06	0.76	0.63
688	TRFL_HUMAN	1.54	1.60	1.75	1.45	1.88	2.56	1.57	1.88	1.99	0.93	0.95	0.88	0.97	1.10	1.12	1.36	1.08
837	TRXR1_HUMAN	0.79	0.37	0.10	0.94	1.03	1.00	0.94	0.99	1.12	0.86	0.80	1.02	0.95	2.40	2.14	8.38	8.66
849	TRXR1_HUMAN	0.88	0.52	0.34	0.89	0.90	0.83	0.87	0.96	0.95	0.93	0.88	0.95	0.87	1.62	1.64	2.24	2.42
751	TRXR1_HUMAN	2.00	2.51	3.35	1.65	2.32	3.51	2.17	2.30	2.73	0.91	0.83	0.75	0.91	0.84	0.76	0.95	0.68
1335	TSNAX_HUMAN	1.16	0.83	0.88	1.13	1.30	1.54	1.22	1.25	1.15	1.03	1.06	1.01	1.12	1.61	1.59	1.80	1.39
531704	TSNAX_HUMAN	0.86	0.39	0.31	0.86	0.78	0.68	0.94	0.84	0.70	1.04	0.97	1.05	1.06	2.06	2.08	2.34	2.24
16669	TXD17_HUMAN	0.82	0.88	0.69	0.97	0.73	0.75	2.44	1.24	1.99	1.66	1.20	1.96	3.59	1.38	1.69	1.78	3.44
959	TXND5_HUMAN	0.71	0.70	0.55	0.78	0.62	0.54	0.72	0.76	0.52	0.97	0.97	1.07	0.98	0.86	1.06	0.95	0.91
977	TXND5_HUMAN	0.91	1.04	1.15	1.02	0.84	0.72	0.87	0.96	0.65	0.97	1.05	1.08	1.00	0.78	0.97	0.61	0.59
989	TXND5_HUMAN	1.07	1.23	1.41	1.00	0.91	0.83	0.92	1.02	0.79	1.02	1.07	0.95	0.92	0.76	0.88	0.60	0.60
992	TXND5_HUMAN	0.60	0.93	0.59	0.75	0.82	0.52	0.83	0.96	0.66	0.95	0.71	1.18	0.98	0.83	0.73	0.83	0.80
13083	TXND5_HUMAN	0.77	0.80	0.57	0.86	0.58	0.56	0.71	0.82	0.59	1.09	1.08	1.21	0.99	0.80	1.11	1.08	1.12
177384	TXND5_HUMAN	0.87	0.86	0.69	0.88	0.75	0.66	0.83	0.89	0.72	0.95	0.84	0.95	0.80	0.83	0.88	0.91	0.88
203683	TXND5_HUMAN	1.58	2.39	2.80	1.19	1.28	1.38	1.27	1.80	1.69	0.99	0.86	0.75	0.69	0.53	0.65	0.49	0.52
3102815	TXND5_HUMAN	0.86	0.97	0.89	0.89	0.78	0.68	0.69	0.81	0.60	0.99	1.09	1.02	0.87	0.80	0.91	0.75	0.74
36428	U2AF2_HUMAN	0.83	0.27	0.01	0.91	0.94	1.04	0.66	0.76	0.67	0.91	1.27	1.00	1.02	3.20	3.61	°	°
460	UBA1_HUMAN	0.64	0.60	0.39	0.73	0.61	0.53	0.70	0.78	0.59	1.07	1.07	1.24	1.17	1.08	1.38	1.43	1.60
466	UBA1_HUMAN	0.60	0.56	0.38	0.76	0.62	0.55	0.70	0.82	0.64	0.95	0.87	1.20	1.01	1.06	1.29	1.38	1.47
466	UBA1_HUMAN	0.60	0.56	0.38	0.76	0.62	0.55	0.70	0.82	0.64	0.95	0.87	1.20	1.01	1.06	1.29	1.38	1.47
478	UBA1_HUMAN	0.62	0.61	0.40	0.69	0.60	0.54	0.65	0.75	0.56	1.12	1.12	1.25	1.18	1.09	1.36	1.50	1.56

1358	UBA1_HUMAN	0.67	0.69	0.50	0.62	0.53	0.47	0.69	0.64	0.47	1.12	1.07	1.04	1.09	0.87	1.00	1.04	1.01
197182	UBA1_HUMAN	0.69	0.69	0.46	0.73	0.61	0.59	0.64	0.77	0.59	1.13	1.20	1.20	1.12	1.00	1.34	1.45	1.54
549951	UBA1_HUMAN	1.06	1.29	1.24	0.85	0.94	1.06	0.68	0.95	0.75	1.24	1.56	1.00	0.99	0.91	1.15	1.06	0.95
554833	UBA1_HUMAN	0.51	0.48	0.33	0.70	0.56	0.48	0.67	0.78	0.57	0.97	0.85	1.35	1.12	1.13	1.37	1.43	1.48
3542	UBA5_HUMAN	0.69	0.48	0.33	0.88	0.72	0.50	0.82	0.79	0.56	0.93	0.82	1.18	0.97	1.39	1.35	1.43	1.39
1116	UBCP1_HUMAN	0.86	0.54	0.34	0.94	0.80	0.78	1.00	0.97	0.77	1.00	0.98	1.10	1.15	1.50	1.77	2.32	2.24
545	UBP5_HUMAN	0.82	0.76	0.56	0.86	0.67	0.66	0.76	0.90	0.72	1.01	0.95	1.06	0.88	0.89	1.13	1.18	1.22
550	UBP5_HUMAN	0.67	0.67	0.48	0.78	0.67	0.59	0.70	0.79	0.62	0.95	0.91	1.09	0.94	0.95	1.09	1.17	1.18
767	UBQL1_HUMAN	2.46	3.02	4.17	1.78	2.33	3.84	2.15	2.46	2.92	1.02	1.03	0.74	0.90	0.78	0.84	0.94	0.72
1491	UCHL1_HUMAN	0.83	0.88	0.63	0.95	0.89	0.77	0.84	1.00	0.88	0.89	0.89	1.01	0.90	0.90	1.01	1.08	1.25
809	ULA1_HUMAN	0.82	0.70	0.56	0.80	0.71	0.62	0.75	0.87	0.68	1.03	1.00	1.00	0.91	1.03	1.24	1.14	1.23
1031	VAT1_HUMAN	0.71	0.52	0.82	0.77	0.78	0.65	0.85	0.88	0.75	0.91	0.85	0.99	1.02	1.36	1.43	0.73	0.78
854	VATB2_HUMAN	1.12	1.30	1.58	1.12	0.90	0.94	0.85	1.04	0.78	1.06	1.24	1.05	0.94	0.73	1.00	0.63	0.61
864	VATB2_HUMAN	1.07	1.28	1.45	1.02	1.00	0.91	0.82	0.88	0.70	1.00	1.24	0.95	0.95	0.78	0.86	0.63	0.60
377849	VATB2_HUMAN	0.81	0.84	0.69	0.86	0.77	0.65	0.74	0.88	0.61	1.02	1.16	1.08	1.05	0.94	1.22	0.96	1.03
975	VIME_HUMAN	1.42	1.24	1.07	1.48	1.10	1.00	1.47	1.49	1.35	0.95	0.59	0.99	0.62	0.84	0.72	0.88	0.75
1044	VIME_HUMAN	1.69	1.43	1.21	1.69	1.38	1.33	1.71	2.09	1.55	0.93	0.58	0.93	0.58	0.90	0.84	1.03	0.74
3227	VIME_HUMAN	1.42	1.78	2.14	1.27	1.43	1.75	1.35	1.50	1.52	1.01	0.95	0.90	0.90	0.81	0.80	0.82	0.67
13655	VIME_HUMAN	1.00	1.00	0.72	0.87	0.80	0.70	0.61	0.90	0.58	1.04	1.34	0.90	0.82	0.83	1.20	1.00	1.08
2117397	VINC_HUMAN	0.07	0.30	0.16	0.70	0.05	0.03	0.44	0.68	0.08	0.63	0.84	6.17	5.19	0.11	1.90	0.14	0.42
828	VTDB_HUMAN	1.77	2.23	2.89	1.42	1.70	2.40	1.66	1.96	2.07	1.02	0.92	0.82	0.87	0.78	0.81	0.85	0.66
1325	WDR61_HUMAN	0.71	0.89	0.70	0.74	0.78	0.64	0.69	0.78	0.61	0.96	0.94	0.99	0.91	0.84	0.82	0.87	0.82
697	WDR72_HUMAN	1.60	1.44	1.82	1.63	2.22	2.57	1.55	1.08	1.80	0.89	1.11	0.91	1.08	1.37	0.84	1.26	1.11
618	XRCC5_HUMAN	0.73	0.66	0.53	0.75	0.63	0.62	0.68	0.76	0.57	1.05	1.06	1.08	0.98	1.02	1.23	1.23	1.13
629	XRCC5_HUMAN	0.70	0.69	0.58	0.72	0.65	0.63	0.75	0.80	0.63	1.07	0.98	1.10	1.04	1.02	1.14	1.16	1.05
3762281	XRCC5_HUMAN	0.66	0.64	0.49	0.79	0.69	0.67	0.81	0.88	0.68	0.92	0.83	1.09	1.02	0.99	1.15	1.25	1.14
624	ZYX_HUMAN	0.89	0.27	0.01	0.84	0.76	0.70	0.72	0.87	0.69	0.99	1.08	0.94	0.88	2.83	3.50	°	°
644	ZYX_HUMAN	0.92	1.40	2.13	0.84	0.91	0.80	0.64	0.89	0.86	0.83	0.92	0.75	0.64	0.54	0.59	0.31	0.37

Lendeckel et al.:
Proteomic changes of tissue-tolerable plasma (TTP) treated airway epithelial cells and their relation to wound healing

Supplementary Table S-3

Oxidative Stress Response - 24h

Gene Name	Gene Function	Ratio (/Co_24h)		
		Pl30s_24h	Pl60s_24h	Pl120s_24h
TXND5_HUMAN	Thioredoxin domain-containing protein	1,58	2,39	2,80
TXRX1_HUMAN	Thioredoxin reductase	2,00	2,51	3,35
THIO_HUMAN	Thioredoxin	1,39	1,50	1,69
TXRX1_HUMAN	Thioredoxin reductase	0,79	0,37	0,10
TXRX1_HUMAN	Thioredoxin reductase	0,88	0,52	0,34
SODC_HUMAN	Superoxide dismutase [Cu-Zn]	0,71	0,63	0,53
GSTO1_HUMAN	Glutathione S-transferase	0,90	0,28	0,01
GSTO1_HUMAN	Glutathione S-transferase	0,92	0,56	0,25
PRDX3_HUMAN	Thioredoxin-dependent peroxide reductase	0,69	0,28	0,05
PRDX2_HUMAN	Thioredoxin-dependent peroxide reductase	0,71	0,77	0,59
TXND5_HUMAN	Thioredoxin domain-containing protein	0,71	0,70	0,55
TXND5_HUMAN	Thioredoxin domain-containing protein	0,77	0,80	0,57
GLRX3_HUMAN	Glutaredoxin-3	0,82	0,77	0,61
GSTO1_HUMAN	Glutathione S-transferase	0,77	0,88	0,63
GSTP1_HUMAN	Glutathione S-transferase	0,71	0,77	0,59

UV Damage Repair – 24h

Gene Name	Gene Function	Ratio (/Co_24h)		
		Pl30s_24h	Pl60s_24h	Pl120s_24h
DPOD2_HUMAN	DNA polymerase subunit	0,43	0,36	0,20
DPOD2_HUMAN	DNA polymerase subunit	0,36	0,20	0,02
MSH2_HUMAN	DNA mismatch repair protein	0,66	0,57	0,48
RFA2_HUMAN	Replication protein A	0,66	0,51	0,41
RUVB2_HUMAN	RuvB-like 2	0,63	0,56	0,55
RD23B_HUMAN	UV excision repair protein	1,95	2,35	3,17
RFA2_HUMAN	Replication protein	1,02	0,22	0,16
DDB1_HUMAN	DNA damage-binding protein	0,72	0,66	0,61
RUVB1_HUMAN	RuvB-like 1	0,87	0,62	0,46
PCNA_HUMAN	Proliferating cell nuclear antigen	0,83	0,83	0,61
RD23A_HUMAN	UV excision repair protein	0,87	0,73	0,55
MRE11_HUMAN	Double-strand break repair protein	1,14	1,40	1,56

Oxidative Stress Response - 48h

Gene Name	Gene Function	Ratio (/Co_48h)		
		Pl30s_48h	Pl60s_48h	Pl120s_48h
TRXR1_HUMAN	Thioredoxin reductase	1,65	2,32	3,51
PRDX3_HUMAN	Thioredoxin-dependent peroxide reductase	0,79	0,65	0,53
TXND5_HUMAN	Thioredoxin domain-containing protein	0,78	0,62	0,54
TXND5_HUMAN	Thioredoxin domain-containing protein	0,86	0,58	0,56
GSTO1_HUMAN	Glutathione S-transferase	0,78	0,88	0,61
GSTP1_HUMAN	Glutathione S-transferase	0,85	0,71	0,65
TXND5_HUMAN	Thioredoxin domain-containing protein	0,75	0,82	0,52
TXND5_HUMAN	Thioredoxin domain-containing protein	0,88	0,75	0,66
SODC_HUMAN	Superoxide dismutase [Cu-Zn]	0,70	0,68	0,54
SODC_HUMAN	Superoxide dismutase [Cu-Zn]	0,77	0,86	0,57
PRDX2_HUMAN	Thioredoxin-dependent peroxide reductase	0,85	0,71	0,65

UV Damage Repair - 48h

Gene Name	Gene Function	Ratio (/Co_48h)		
		Pl30s_48h	Pl60s_48h	Pl120s_48h
DPOD2_HUMAN	DNA polymerase subunit	0,59	0,46	0,31
RUVB2_HUMAN	RuvB-like 2	0,55	0,58	0,41
DPOD2_HUMAN	DNA polymerase subunit	0,42	0,29	0,07
MSH2_HUMAN	DNA mismatch repair protein	0,70	0,58	0,54
PCNA_HUMAN	Proliferating cell nuclear antigen	0,88	0,66	0,65
RD23A_HUMAN	UV excision repair protein	0,88	0,55	0,46
RFA2_HUMAN	Replication protein	0,83	0,63	0,50
RD23B_HUMAN	UV excision repair protein	1,42	2,08	3,42
PCNA_HUMAN	Proliferating cell nuclear antigen	0,81	0,68	0,56
RUVB2_HUMAN	RuvB-like 2	0,86	0,77	0,65
DPOD2_HUMAN	DNA polymerase subunit	0,69	0,71	0,50
DDB1_HUMAN	DNA damage-binding protein	0,78	0,67	0,65
PPP6_HUMAN	Serine/Threonine-protein phosphatase	0,82	0,76	0,58
MRE11_HUMAN	Double-strand break repair protein	1,21	1,43	1,87

Oxidative Stress Response - 72h

Gene Name	Gene Function	Ratio (/Co_72h)		
		Pl30s_72h	Pl60s_72h	Pl120s_72h
TRXR1_HUMAN	Thioredoxin reductase	2,17	2,30	2,73
PRDX4_HUMAN	Thioredoxin-dependent peroxide reductase	2,04	2,22	1,97
TXND5_HUMAN	Thioredoxin domain-containing protein	1,27	1,80	1,69
THIO_HUMAN	Thioredoxin	1,75	1,21	2,08
SODC_HUMAN	Superoxide dismutase [Cu-Zn]	0,68	0,70	0,56
PRDX2_HUMAN	Thioredoxin-dependent peroxide reductase	0,74	0,84	0,60
PRDX3_HUMAN	Thioredoxin-dependent peroxide reductase	0,79	0,85	0,55
GSTP1_HUMAN	Glutathione S-transferase	0,74	0,84	0,60
TXND5_HUMAN	Thioredoxin domain-containing protein	0,72	0,76	0,52
TXND5_HUMAN	Thioredoxin domain-containing protein	0,87	0,96	0,65
TXND5_HUMAN	Thioredoxin domain-containing protein	0,83	0,96	0,66
TXND5_HUMAN	Thioredoxin domain-containing protein	0,71	0,82	0,59
TXND5_HUMAN	Thioredoxin domain-containing protein	0,69	0,81	0,60
SODC_HUMAN	Superoxide dismutase [Cu-Zn]	0,76	0,99	0,66

UV Damage Repair - 72h

Gene Name	Gene Function	Ratio (/Co_72h)		
		Pl30s_72h	Pl60s_72h	Pl120s_72h
DPOD2_HUMAN	DNA polymerase subunit	0,36	0,44	0,17
DPOD2_HUMAN	DNA polymerase subunit	0,49	0,62	0,39
DPOD2_HUMAN	DNA polymerase subunit	0,68	0,65	0,46
RD23B_HUMAN	UV excision repair protein	1,64	1,72	2,28
PCNA_HUMAN	Proliferating cell nuclear antigen	0,70	0,83	0,55
MSH2_HUMAN	DNA mismatch repair protein	0,68	0,75	0,52
RD23A_HUMAN	UV excision repair protein	0,93	0,87	0,54
PCNA_HUMAN	Proliferating cell nuclear antigen	0,79	0,88	0,58
RFA2_HUMAN	Replication protein	0,90	0,87	0,51
RUVB2_HUMAN	RuvB-like 2	0,74	0,88	0,61

Lendeckel et al.:
Proteomic changes of tissue-tolerable plasma (TTP) treated airway epithelial cells and their relation to wound healing

Supplementary Table S-4

Cell Death / Apoptotic Factors - 24h

**Gene Name**	**Gene Function**	**Ratio (/Co_24h)**		
		Pl30s_24h	Pl60s_24h	Pl120s_24h
MX1_HUMAN	Interferon-induced GTP-binding protein	0,63	0,66	0,40
LMNA_HUMAN	Prelamin	0,81	0,47	0,36
DP13A_HUMAN	DCC-Interacting Protein	0,67	0,85	0,59
IF5A1_HUMAN	Eukaryotic translation initiation factor	0,74	0,86	0,54
LEG1_HUMAN	Galectin-1	0,75	0,84	0,56
LMNA_HUMAN	Prelamin	1,01	0,40	0,10
BID_HUMAN	BH3-Interacting domain death agonist	0,69	0,69	0,49
NDUS1_HUMAN	NADH-ubiquinone oxidoreductase	0,88	0,88	0,59
UCHL1_HUMAN	Ubiquitin carboxyl-terminal hydrolase	0,83	0,88	0,63
CATB_HUMAN	Cathepsin B	0,67	0,78	0,58
ULA1_HUMAN	NEDD8 activating enzyme	0,82	0,70	0,56

Anti-Apoptotic Factors – 24h

**Gene Name**	**Gene Function**	**Ratio (/Co_24h)**		
		Pl30s_24h	Pl60s_24h	Pl120s_24h
ANXA5_HUMAN	Annexin A5	1,51	1,54	1,93
GDIA_HUMAN	Rab GDP dissociation inhibitor	1,77	2,23	2,89
GRP75_HUMAN	Stress-70 protein	1,47	2,16	2,40
TF65_HUMAN	Transcription factor p65	1,47	2,16	2,40
GDIA_HUMAN	Rab GDP dissociation inhibitor	1,23	1,50	1,84

Cell Proliferation / Cell Division - 24h

**Gene Name**	**Gene Function**	**Ratio (/Co_24h)**		
		Pl30s_24h	Pl60s_24h	Pl120s_24h
HDGF_HUMAN	Hepatoma-derived growth factor	0,66	0,56	0,21
PA2G4_HUMAN	Proliferation-associated protein	0,66	0,32	0,01
MD1L1_HUMAN	Mitotic spindle assembly checkpoint protein	0,47	0,51	0,38
HDGF_HUMAN	Hepatoma-derived growth factor	0,91	0,66	0,56
HDGF_HUMAN	Hepatoma-derived growth factor	0,72	0,67	0,47
GMFB_HUMAN	Glia maturation factor	0,76	0,89	0,61
HDGF_HUMAN	Hepatoma-derived growth factor	0,81	0,73	0,45
PA2G4_HUMAN	Proliferation-associated protein	0,74	0,69	0,59
PP1A_HUMAN	Serine/threonine-protein phosphatase	0,88	0,61	0,27
CPNS1_HUMAN	Calpain	0,75	0,93	0,67
DCTN2_HUMAN	Dynactin	0,81	0,84	0,66
HDGF_HUMAN	Hepatoma-derived growth factor	0,76	0,76	0,55

Cell Death / Apoptotic Factors- 48h

Gene Name	Gene Function	Ratio (/Co_48h)		
		Pl30s_48h	Pl60s_48h	Pl120s_48h
NDUS1_HUMAN	NADH-ubiquinone oxidoreductase	0,77	0,60	0,57
BID_HUMAN	BH3-Interacting domain death agonist	0,79	0,81	0,65
DP13A_HUMAN	DCC-Interacting Protein	0,76	0,67	0,54
IF5A1_HUMAN	Eukaryotic translation initiation factor	0,83	0,86	0,62
LEG1_HUMAN	Galectin-1	0,98	0,75	0,66
CATB_HUMAN	Cathepsin B	0,78	0,76	0,66
ULA1_HUMAN	NEDD8 activating enzyme	0,80	0,71	0,62

Anti-Apoptotic Factors – 48h

Gene Name	Gene Function	Ratio (/Co_48h)		
		Pl30s_48h	Pl60s_48h	Pl120s_48h
GDIA_HUMAN	Rab GDP dissociation inhibitor	1,42	1,70	2,40
GRP75_HUMAN	Stress-70 protein	1,47	1,91	2,97
TF65_HUMAN	Transcription factor p65	1,47	1,91	2,97
GDIA_HUMAN	Rab GDP dissociation inhibitor	1,08	1,23	1,54

Cell Proliferation / Cell Division - 48h

Gene Name	Gene Function	Ratio (/Co_48h)		
		Pl30s_48h	Pl60s_48h	Pl120s_48h
HDGF_HUMAN	Hepatoma-derived growth factor	0,56	0,50	0,46
EXOS4_HUMAN	Exosome complex component	0,85	0,66	0,63
MD1L1_HUMAN	Mitotic spindle assembly checkpoint protein	0,70	0,56	0,58
PA2G4_HUMAN	Proliferation-associated protein	0,68	0,75	0,54
CPNS1_HUMAN	Calpain	0,84	0,82	0,62

Cell Death / Apoptotic Factors - 72h

Gene Name	Gene Function	Ratio (/Co_72h)		
		Pl30s_72h	Pl60s_72h	Pl120s_72h
BID_HUMAN	BH3-Interacting domain detah agonist	0,68	0,71	0,50
MX1_HUMAN	Interferon-induced GTP-binding protein	0,67	0,86	0,60
NDUS1_HUMAN	NADH-ubiquinone oxidoreductase	0,70	0,83	0,48
CATB_HUMAN	Cathepsin B	0,67	0,70	0,59

Anti-Apoptotic Factors – 72h

Gene Name	Gene Function	Ratio (/Co_72h)		
		Pl30s_72h	Pl60s_72h	Pl120s_72h
GDIA_HUMAN	Rab GDP dissociation inhibitor	1,66	1,96	2,07
GRP75_HUMAN	Stress-70 protein	1,38	1,80	2,07
TF65_HUMAN	Transcription factor p65	1,38	1,80	2,07
NPM_HUMAN	Nucleophosmin	1,23	1,66	1,59

Cell Proliferation / Cell Division - 72h

Gene Name	Gene Function	Ratio (/Co_72h)		
		Pl30s_72h	Pl60s_72h	Pl120s_72h
HDGF_HUMAN	Hepatoma-derived growth factor	0,67	0,55	0,59
CPNS1_HUMAN	Calpain	0,71	0,81	0,62
HDGF_HUMAN	Hepatoma-derived growth factor	0,70	0,85	0,59
HDGF_HUMAN	Hepatoma-derived growth factor	0,71	0,80	0,56
EXOS4_HUMAN	Exosome complex component	0,81	0,81	0,59
MD1L1_HUMAN	Mitotic spindle assembly checkpoint protein	0,75	0,85	0,62
